# Epigenetic Changes in the Regulation of *Nicotiana tabacum* Response to *Cucumber Mosaic Virus* Infection and Symptom Recovery through Single-Base Resolution Methylomes

**DOI:** 10.3390/v10080402

**Published:** 2018-07-29

**Authors:** Chenguang Wang, Chaonan Wang, Wenjie Xu, Jingze Zou, Yanhong Qiu, Jun Kong, Yunshu Yang, Boyang Zhang, Shuifang Zhu

**Affiliations:** 1 College of Plant Protection, China Agricultural University, Beijing 100083, China; italy10.wang@gmail.com (C.W.); wangcn@163.com (C.W.); xuwenjieee@163.com (W.X.); kongjun@163.com (J.K.); 2 Institute of Plant Quarantine, Chinese Academy of Inspection and Quarantine, Beijing 100083, China; zoujze@outlook.com (J.Z.); qiuyanhong124@126.com (Y.Q.); 3 College of Biological Sciences, China Agricultural University, Beijing 100083, China; 4 Beijing Academy of Food Sciences, Beijing 100162, China; yangys019@hotmail.com; 5 College of Food Science and Nutritional Engineering, China Agricultural University, Beijing 100083, China; bojanchang@cau.edu.cn

**Keywords:** *Nicotiana tabacum*, *Cucumber mosaic virus*, DNA methylation, gene expression, RNAi, demethylation

## Abstract

Plants have evolved multiple mechanisms to respond to viral infection. These responses have been studied in detail at the level of host immune response and antiviral RNA silencing (RNAi). However, the possibility of epigenetic reprogramming has not been thoroughly investigated. Here, we identified the role of DNA methylation during viral infection and performed reduced representation bisulfite sequencing (RRBS) on tissues of *Cucumber mosaic virus* (CMV)-infected *Nicotiana tabacum* at various developmental stages. Differential methylated regions are enriched with CHH sequence contexts, 80% of which are located on the gene body to regulate gene expression in a temporal style. The methylated genes depressed by methyltransferase inhibition largely overlapped with methylated genes in response to viral invasion. Activation in the argonaute protein and depression in methyl donor synthase revealed the important role of dynamic methylation changes in modulating viral clearance and resistance signaling. Methylation-expression relationships were found to be required for the immune response and cellular components are necessary for the proper defense response to infection and symptom recovery.

## 1. Introduction

Plants have acquired a series of adaptations to survive under adverse environmental stress, including poor soil, low metal availability, abiotic stress, and viral infection [1,2,3]. Consequently, plants have evolved molecular, metabolic and physiological mechanisms to counter environmental challenges. These mechanisms include genome recombination, gene expression regulation, transcriptional and post-transcriptional gene silencing, epigenetic modifications, and post-translational modifications. Epigenetic changes in the DNA loci are primarily DNA methylation and histone modification and result in the repression of gene expression and corresponding heritable morphological variations without altering the concrete DNA sequence [4,5,6,7]. In plants, DNA methylation is applied in three different types, namely, CG, CHG, and CHH (where H is A, C, or T), and is enriched in transposable elements (TEs) and coding regions to affect gene transcription and expression [8,9]. In Arabidopsis, CG methylation is maintained by MET1. CHG and CHH are site-specifically methylated by CMT3 [10] and DRM2 [11], as well as CMT2 [12]. CHH sites are not symmetric, and their methylation is mainly mediated by RNA-directed DNA methylation pathways (RdDM) [13], which require formation of double-stranded RNAs (dsRNAs) by plant-specific RNA polymerase IV (Pol IV) and RNA-dependent RNA polymerase 2 (RDR2) and procession by Dicer-like 3 (DCL3) and Argonaute 4 (AGO4) to guide DNA methylation with the help of RNA polymerase [14,15,16].

DNA methylation is dynamic and has been fully characterized in embryonic stem cells, germ cells, microbial cells, as well as single tetrads in maize population using the newly developed high-throughput single-cell sequencing [7,17,18,19,20]. These high-quality sequencing data facilitated the visualization of DNA methylation at a single-nucleotide resolution and paved the way to elucidate the complex epigenetic programming through cell division and differentiation [21,22]. DNA methylation can also be reprogrammed to affect gene expression through developmental signals, external stresses and pathogen invasion, and it is involved in the evolution of plants and social insects [23], plant development [24,25,26,27], phosphate starvation responses [28], and antibiotic defense [29,30,31], as well as resistance to viruses [32]. However, plant viruses acquired invasion mechanisms that differ from other pathogens (bacteria and fungi), and although it is possible that these mechanisms are linked [33], it still remains uncertain if viral infection causes methylation reprogramming.

*Cucumber mosaic virus* (CMV) is the type virus of the genus *Cucumovirus* in the family *Bromoviridae*, and it can infect more than 1200 plant species [34]. The CMV genome is composed of three positive-stranded RNAs. RNA1 and RNA2 encode two proteins that comprise the viral RNA-dependent RNA polymerase subunits. M strain of CMV (M-CMV) is highly virulent to the tobacco plants, but the disease development process includes a transient recovery period when the newly emerged leaves appear nearly healthy [35], which presents as an obvious phenomenon in *Nicotiana tabacum* cv. Xanthi nc. RNA silencing (RNAi) is the major antiviral defense in plants and is triggered by dsRNAs, followed by DCL splicing and RNA-induced silencing complexes (RISC) activity [2]. RNAi has been shown to be responsible for viral clearance and symptom recovery through post-transcriptional gene silencing (PTGS) and transcriptional gene silencing (TGS) [36]. Moreover, metabolic programming and innate immunity processes were also significantly influenced during the symptom development stages [35]. However, several features of the epigenetic mechanisms that regulate gene expression in response to viral infection remain unknown, although some evidence suggests that genes encoded by virus or plant genome-mediated DNA methylation reinforces DNA methylation-mediated antiviral silencing [37,38,39]. 

In this study, we report the whole genome DNA methylation patterns in CMV-infected tobacco tissues through reduced representation bisulfite sequencing (RRBS) and discuss the interaction between gene expression and DNA methylation in chlorosis and the recovery stage. Our data suggest that DNA methylation plays a dynamic role in the modulation of plant response to CMV infection and suppression.

## 2. Materials and Methods

### 2.1. Plant Growth and Virus Inoculation

Tobacco plants (*Nicotiana tabacum* cv. Xanthi nc) and *Nicotiana benthamiana* were grown in a growth room with a controlled environmental climate programmed for 16 h of light at 24 °C and 8 h in the dark at 21 °C. Seedlings with up to eight fully expanded leaves were used for virus inoculations. The viral particles (20 mg/mL, stored at our laboratory) were rub-inoculated onto the top two leaves (each with 10 μL) of tobacco plants having four fully expanded leaves, as reported previously [35]. The mock inoculum was prepared from leaves of healthy plants and applied in the same way as viral inoculum. All the experiments were repeated at least four times with reproducible results. In order to eliminate the variation between individual plants, five leaves from five different plants were mixed as reported previously. Sporadic dots of systemically infected leaf at each stage (11 dpi, 13 dpi, 13 dr, 16 dpi, and mock tissue) from M-CMV infected plants were harvested for DNA and RNA extraction (see Supplemental Figure S1). The sporadic dots were chosen in the middle panel of leaves at each stage. A total of 250 dots (50 × 4 stages + healthy leaves within 11–15 dpi) were finally excised.

For 5-azacytidine treatment, 1 μM 5-azacytidine was sprayed onto the upper leaves of four-leaf-stage *N. tabacum* for constitutive four days. The viral inoculum was then rub-inoculated onto the top two leaves of tobacco plants. Leaves were collected for RNA extraction at 7 dpi.

### 2.2. VIGS Assay

VIGS assays were performed by following the protocol previously described [40]. Fragments (around 350–500 bp) of NbMET1, NbCMT3, NbDRM1, NbSAMS1 cloned and inserted into pTRV2. pTRV1, or pTRV2 derivatives were introduced into *A. tumefaciens* strain GV3101. *Agrobacterium* cultures (OD_600_ = 0.8) were mixed at a 1:1 ratio and infiltrated into the lower leaves of four-leaf-stage *Nicotiana benthamiana* using a needleless syringe. *Agrobacterium* cultures containing empty vectors were used as negative control. Ten days after infiltration, the upper leaves of the plants were infected with M-CMV by rub inoculation. Each silencing experiment was repeated at least five times, and each experiment included ten independent plants.

### 2.3. DNA and RNA Extraction

The collected leaf dots were frozen in liquid nitrogen for DNA extraction with DNeasy Plant Mini Kit (Qiagen, Hilden, Germany) according to the manufacturer’s instructions. Total RNA was extracted using Trizol Reagent (Invitrogen, Carlsbad, CA, USA) following the manufacturer’s instructions and then treated with DNase I (Invitrogen). The concentration and purity of DNA and RNA of each sample were determined using NanoDrop N2000 (Thermo Fisher, Wilmington, DE, USA) according to the OD260 and OD280 values.

### 2.4. Reduced Representation Bisulfite Sequencing (RRBS) Library Construction and Sequencing

Samples of 1–5 ng of genomic DNA were used to construct the RRBS library, as previously described with minor modifications [41]. Briefly, the isolated genomic DNAs, together with 1% unmethylated lambda DNA (Thermo Scientific, Wilmington, DE, USA), were subjected to MspI digestion (Fermentas, Waltham, MA, USA), dA-tailing with 5 units of klenow polymerase (3′ to 5′ exo-, New England Biolabs, Ipswich, MA, USA), and adapter ligation with methylated adapter (New England Biolabs, Ipswich, MA, USA). The samples were then subject to bisulfite conversion using a MethylCode Bisulfite Conversion Kit (Invitrogen, Carlsbad, CA, USA) according to the manufacturer’s instructions. After 0.8 × Agencourt Ampure XP beads (Beckman Coulter, Brea, CA, USA) purification, the beads were further collected and washed several times, and the library was generated with 25 cycles of PCR amplifications using 1 U Kapa HiFi HS DNA Polymerase (Kapa Biosystems, Wilmington, MA, USA), together with 0.15 μM Illumina Forward universal primer and 0.15 μM pre-indexed Illumina Reverse primer (Illumina, San Diego, CA, USA). Amplified libraries were purified with 0.8 × XP beads twice and were assessed using Qubit ds HS assay (Invitrogen, Carlsbad, CA, USA). The final quality-ensured libraries were pooled and sequenced on the Illumina HiSeq2000/2500 sequencer for 150 bp paired-end sequencing. Duplicate libraries were prepared for each stage.

### 2.5. Identification and Analysis of Differential Methylated Regions (DMRs)

Pair end reads generated by Illumina sequencing were aligned to the TN90 Nicotiana tabacum genome and the cl857 Sam7 Lambda genome (48,502 bases) using BS-Seeker2 (v2.1.1) with default parameters, except –m to 0.06 [42]. Clean reads for each biological replicate were processed and aligned independently as previously reported. The bulk methylation levels were analyzed for cytosine sites with ≥10× in each sample using CGmapTools [43]. Methylated and non-methylated cytosines were evaluated for the significance of treatments by the *F* test. As the RRBS DNA methylomes are fragmented in the covered region, the DMRs were identified through a dynamic fragmentation strategy in CGmapTools. DMRs for each sample were defined by comparing methylation levels to mock inoculations in 100 bp bins across the genome. Fischer’s exact test was used to identify bins that were significantly differentially methylated (Benjamini–Hochberg corrected FDR < 0.01) and absolute methylation difference (ΔmC >0.1) for CG, CHG, CHH methylation, respectively. Bins that were within 100 bp were merged. Finally, only bins that contained 10 informative cytosines (i.e., covered by ≥4 reads) were considered as DMRs. DMRs are listed in Table S9.

### 2.6. mRNA Sequencing and Analysis

The RNA-seq libraries were prepared using the TruSeq RNA Sample Preparation Kit from Illumina (San Diego, CA, USA). Libraries were sequenced on an Illumina HiSeq 2000 to generate GB paired-end reads. Duplicate libraries were prepared for each stage. Clean mRNA reads were mapped to TN90 Nicotiana tabacum genome and its annotation [44] using Bowtie allowing two mismatches, and differentially expressed genes (DEGs) were processed defined by applying two-fold and *q* < 0.01 cutoff.

### 2.7. Analysis of Correlation of DMRs and DEGs

All heat maps in this study were generated by complete linkage and using Euclidean distance as a distance measure. The mC distributions across the gene body were analyzed by CGmapTools. To determine overlap of DMRs with different genomic elements, we considered 1 bp overlap as an overlap, and examined their overlaps with the different genomic elements. The positive and negative association with both differential methylation changes and differential expression were extracted using our own Perl scripts. DMRs identified with this approach were subjected to a detailed analysis to categorized the genomic region in which the methylation changes occurred, the level and direction of differential expression change (upregulated, downregulated), and the direction of methylation change (hypermethylation, hypomethylation).

### 2.8. Functional Enrichment Analysis

Because high-quality genome sequence information is available for Arabidopsis, and because antiviral defenses are well studied in that model, for our functional analysis, we chose to compare our transcriptome results with Arabidopsis-virus responses as previously reported [45]. Gene lists generated from the analysis above were listed and blasted with Arabidopsis annotation (TAIR10) with highest protein coverage. Arabidopsis-related lists were then analyzed in AgriGO v.2 (http://systemsbiology.cau.edu.cn/agriGOv2/index.php) [46]. The enriched biological categories were selected in a cut point in a normalized frequency (NF = Relative frequency of set/Relative frequency of reference) that was greater than or equal to 1.5-fold and *p* < 0.05.

### 2.9. Small RNA-seq and Data Analysis

Small RNA-seq libraries were constructed from total RNA isolated from the same tissues as described for the mRNA libraries, using the TruSeq Small RNA Sample Prep Kit from Illumina (San Diego, CA, USA). The libraries were sequenced on the Illumina HiSeq 2000 as the mRNA-seq libraries. Bowtie-0.12.7 was used for mapping clean reads with perfect matches to the tobacco genome, and reads were categorized by their lengths for analysis [47]. The abundance of 24-nt small RNAs with fixed-size bins (500 nt) was calculated.

### 2.10. qRT-PCR

Total RNAs were extracted from indicated plant materials using Transzol reagent (Transgen, Beijing, China) and reverse transcribed using Trans-Script One-Step gDNA Removal and cDNA Synthesis SuperMix (Transgen, Beijing, China). Quantitative RT-PCR was performed with ABi 7500 real-time PCR system using SYBR Select Master Mix (Life Technologies, Carlsbad, CA, USA). EF1α was used as the internal control. Primers are listed in the Supplemental Information (Table S1).

### 2.11. Detection of Hydrogen Peroxide

Detection of ROS generation is according to that previously report [48]. Briefly, leaves were vacuum-infiltrated with H_2_DCF-DA in phosphate buffer for 10–30 s and placed in the dark for 45 min after being kept in the dark for 2 h. The fluorescence emission spectrum of H_2_O_2_ sensors was recorded at room temperature using a Hitachi F-7000 fluorescence spectrophotometer (Hitachi, Tokyo, Japan).

## 3. Results

### 3.1. Loss of Methyltransferase Expression Activates Antiviral Defense

**Figure 1:**
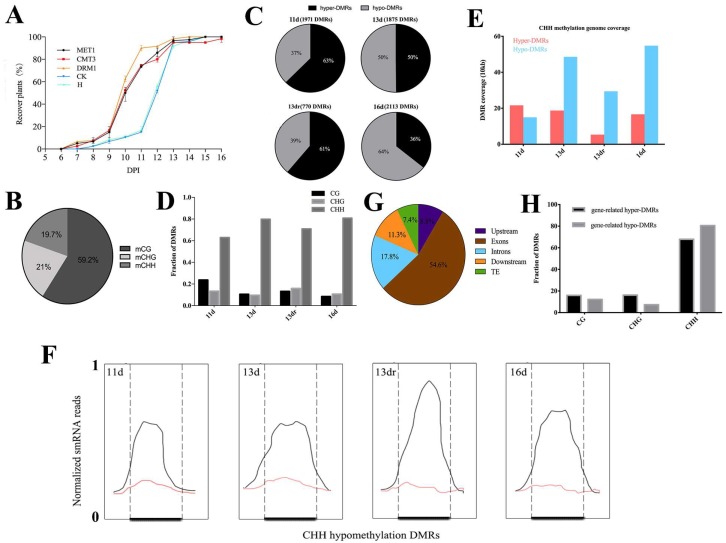
Viral infection is associated with DNA methylation and demethylation. (**A**) Developmental stages of tobacco leaves from 11 d to 16 d and their comparison with healthy leaf. Green parts of leaf at 13 d stage are marked as 13 dr. Red dots are selected tissues for DNA extraction and library construction; (**B**) Context breakdown for mCs. Values are the mean percentages of mCs in all libraries; (**C**) Total number of differentially methylated regions (DMRs) and distribution of hyper- and hypo-DMRs; (**D**) Context enrichment of DMRs; (**E**) Genome coverage of identified CHH hyper- and hypo-DMRs; (**F**) Average distribution of 24-nt siRNA reads in healthy tissues (black) and infected tissues at different stages (red) over defined CHH hypomethylation DMRs in indicated samples. The X-axis indicates CHH hypomethylated DMRs (dark lines in the middle) and their flanking regions. Flanking regions are the same length as middle regions. siRNA reads were normalized based on their frequency; (**G**) Percentages of DMRs mapping to different genomic categories; (**H**) Context enrichment of gene-related DMRs.

CMV-infected tabacum has been characterized previously [35]. Briefly, tobacco leaves above the inoculated leaves initially show vein clearing 5–7 days post inoculation (dpi), which is subsequently developed to the mosaic symptom at 8–10 dpi. At 11, 12 dpi, the newly emerging leaves show severe chlorosis, but contain normal green (or partially recovered) regions at 12, 13 dpi. Finally, the new emerging leaf at 16 dpi often shows near complete recovery. However, this recovery process is not continuous and was inhibited by another cycle of pathogenesis. The concrete stage of disease development and transient recovery induced by M-CMV is our focus. Fractions of tissues were collected for DNA extraction on 11, 13, and 16 dpi based on the previous definition of infected stages, which were marked 11 d, 13 d, and 16 d, respectively (Figure 1A). At 13 dpi, tissues that showed partial recovery were also collected and identified as 13 dr. Healthy leaves were a mixture of developmental stages. To identify possible regulators involved in DNA methylation-mediated antiviral defense, we devised a series of well-based virus-induced silencing (VIGS) system. We cloned fragments of DNA methyltransferase genes, including methyltransferase 1 (MET1), chromomethylase 3 (CMT3) and domains rearranged methylase 1 (DRM1), and then infiltrated these into tobacco plants. RNAi lines were characterized according to their level of downregulation of genes and then inoculated with CMV sap (Figure S1C). Subsequently, M-CMV was inoculated into the upper leaves. In these RNAi plants, an accelerated recovery time was observed (Figure S1A,B). 

The expression tendency during the viral infection was also evaluated through quantitative RT-PCR (Figure S1D). The expression of methyltransferase genes (*MET1, CMT3,* and *DRM1*) was significantly up-regulated at 11 d and 13 d, and returned to normal expression level at 13 dr and 16 d. In RNAi lines these genes were up-regulated earlier than wild-type plants. The DNA demethylases, repressor of silencing 1 (ROS1) and demeter-like 2 (DML2), were also increased 3-fold and 4-fold, respectively, throughout the entire developmental stage, indicating the loss of methylation in the genome context. These results indicate the evidence of DNA methylation and demethylation that involved in symptom recovery and antiviral defense.

### 3.2. Viral Infection Is Associated with CHH Methylation

To evaluate the global changes in DNA methylation induced by viral infection, we performed RRBS to show the methylation profiling of CMV-infected plants and RNAi lines. We used Illumina next-generation sequencing platforms for bisulfite-converted genomic DNA sequencing with high coverage and bisulfite conversion (Table S2 and Figure S2). The percent of methylcytosines over total cytosines in each specific sequence context was 59.2% for mCG, 21% for mCHG and 19.7% for mCHH (Figure 1B). The genome averages of three forms of methylation were similar to other plants [49,50,51,52] (Figure S2). However, the methylome of tobacco after viral infection is not typical of that observed in higher eukaryotes, where negative correlation occurred between CG and CHG methylation and the gene body (Table S3). This result suggested that methylation levels are interfered with by viral infection. 

To examine whether the dynamic regulation of DNA methylation by CMV occurred in specific genomic domains, we performed a clustering analysis to identify differentially methylated regions (DMRs) with a dynamic fragmentation strategy [43]. At 11 d and 13 dr, we observed a predominant hypermethylation tendency (61% and 63%) compared to hypomethylation (39% and 37%) (Figure 1C). Although mCG context was the most popular pattern in the methylated site, all samples were enriched with CHH DMRs (Figure 1D and Figure S3A–D) in the fraction of whole DMRs, or DMRs coverage (Figure 1E and Figure S3E,F). On average, we identified 2303 CHH hypermethylation DMRs and 2685 CHH hypomethylation DMRs in the infected tissues; the size of these regions ranged from 744 to 3 bp (Figure S4). CHH methylation is always associated with small interfering RNAs of 24-nt in length (24-nt siRNAs) [9,12]. From small RNA sequencing results, siRNAs are enriched over these hypomethylated sites in wild type, but eliminated in infected plants. Hence loss of DNA methylation is associated with loss of 24-nt siRNAs (Figure 1F). However, viral infection resulted in relative increases of 21-nt RNA populations and 22-nt siRNA populations with a concomitant reduction in 24-nt RNA populations, which produced similar populations of 24-nt siRNA as healthy plants (Figure S5). This result suggested that other mechanisms that regulated no-CG methylation may function at recovery stages, although DNA demethylation is associated with the loss of 24-nt siRNAs at DMRs. 

To assess whether DNA methylation preferentially occurs in specific genomic contexts, we classified all of the DMRs into the following categories: intergenic, upstream (2000 bp upstream from the transcription start site), gene body (including 5′ and 3′ untranslated regions, exons, and introns), exons, introns, downstream (2000 bp downstream from the transcription terminate site), and transposable element (TE). The allocation demonstrated that 54.6% of the DMRs mapped to exons, 17.8% in introns, 11.3% in downstream, 8.8% in upstream, and 7.4% in TEs (Figure 1G). The methylation contribution was enriched within the gene body, indicating the reprogramming effect of gene-related DMRs on the genic region. DMRs in the gene body were further extracted to analyze the context distribution. The result showed that CHH were the most popular context in both hypermethylated and hypomethylated gene-related DMRs (CG = 19%, CHG = 20%, CHH = 61%) (Figure 1H). These context distributions depict the general behavior of methylated regulation within genic regions, including upstream, gene body, and downstream. 

### 3.3. Methylation Contexts in Gene Body Correlate with Gene Expression

**Figure 2:**
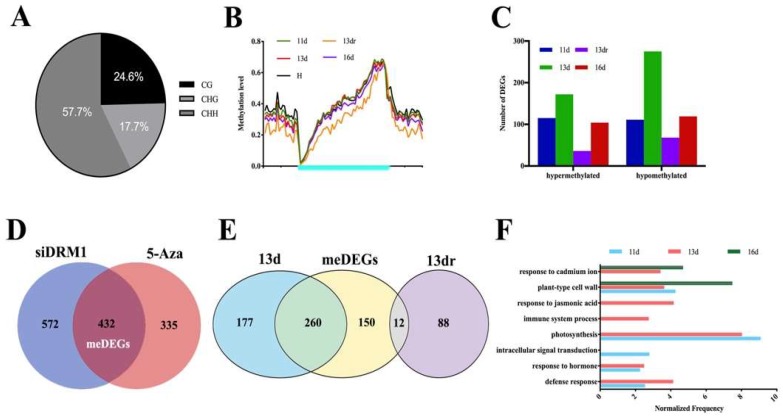
Methylation contexts in the gene body correlate with gene expression. (**A**) Context enrichment of DMR-covered genes; (**B**) Methylation level of gene body (cyan region) and flanking regions (black region); 11d, 13d, 13dr and 16d means infected plants at 11dpi, 13dpi, 13dpi (recovered sites) and 16dpi. H means healthy plants. (**C**) Number of genes affected by hyper- and hypo-DMRs mapping to the gene body that were differentially expressed (up-regulated and down-regulated); (**D**) Venn diagram showing the overlap of DEGs in siDRM1 and 5-Aza treated plants. The overlap genes were defined as meDEGs; (**E**) Venn diagram showing shared DEGs in 13 d, 13 dr, and meDEGs; (**F**) Gene ontology enrichment analysis of meDEGs at each stage. Values along the x-axis represent the normalized frequency (relative frequency of the inquiry set/relative frequency of the reference set), and the enrichment cutoff was greater than 1.5-fold. The *p*-values of all the enriched pathways were less than 0.01.

The DNA methylation in the plant genome has often been correlated with global transcriptional changes [53]. To assess this effect, we also performed RNA-seq of the same treatment analyzed in the methylation profiling. The analysis was systematically classified in terms of the methylation direction and the type of differential expression (up-regulated or down-regulated). Hypermethylated leaves in four stages (11 d, 13 d, 13 dr, and 16 d) were associated with 72, 77, 21, and 53 down-regulated genes, whereas hypomethylated leaves in four stages (11 d, 13 d, 13 dr, and 16 d) were associated with 36, 105, 13, and 40 up-regulated genes (Table S4), most of which are CHH sequence context (Figure 2A). This suggested that DMRs enriched in the gene body are also correlated positively with gene expression (Figure 2B), indicating the complex regulation of DNA methylation in the response of tobacco to viral processes. We also analyzed the correlation of differential methylations with DEGs (Figure 2C). At 11 dpi, at which virions were accumulated at a significant level in the chlorosis leaves, there was displayed a nearly equal distribution of hypermethylation (115 DEGs) and hypomethylation of DEGs (106 DEGs). However, differential methylation in the recovery stage (13 d, 13 dr, and 16 d) displayed a tendency in which hypomethylated DEGs (271, 66, and 113 DEGs) were more abundant than hypermethylated DEGs (166, 34, and 104 DEGs) (Figure S6). 

We also performed the RNA-seq of RNAi lines that target *DRM1* genes (siDRM1) and tobacco plants treated with 1 μM 5-azacytidine (5-Aza), a compound that impairs DNA methylation maintenance. Shared genes that were significantly up-regulated or down-regulated were defined as meDEGs (Figure 2D). Approximately 60% of genes became activated with viral infection (meDEGs), most of which were then down-regulated at normal expression level at the recovery stage (Figure 2E). This analysis indicates not only the DNA methylation, especially CHH methylation involvement in antiviral defense, but also its requirement for deregulation of endogenous methylated genes. 

### 3.4. Functional Analysis of DMRs Located in Coding Genes in a Temporal Regulation

By performing overlap analysis between meDEGs and DEGs at different stages, we found a cluster of activated genes with viral infection regulated by DNA methylation. Functional analysis of meDEGs showed the enriched pathways (Figure 2F, Table S5). DEGs at 11 dpi were strongly enriched in defense response (2.54-fold enrichment, *p* < 0.0001) and intracellular signal transduction (2.78-fold enrichment, *p* = 0.0015), despite the evident photosynthesis pathway (9.08-fold enrichment, *p* < 0.0001). MeDEGs at the 13 dpi stage were enriched in a similar pattern as that observed at 11 dpi. Additionally, meDEGs at 13 dpi were strong enriched in the immune system process (2.75-fold enrichment, *p* = 0.0013) and in response to jasmonic acid (4.17-fold enrichment, *p* < 0.0001). However, such functional pathways are not involved at 13 dr or 16 dpi. In addition, no biological function was enriched in meDEGs at 13 dr, indicating the minor effect of DNA methylation in the partial recovery part of tobacco leaves. DEGs at 16 dpi showed no evident biological enrichment but were enriched in plant-type cell wall (7.48-fold enrichment, *p* < 0.0001) and plasmodesma (2.33-fold enrichment, *p* = 0.0044).

Some characteristic genes were identified and regulated through methylation change (Figure S7). AGO 2, but not AGO 1, is hypomethylated with up-regulated expression at 11 d and 13 d. A fatty acid biosynthesis enzyme (LOC107774635) that is involved in the salicylic acid signaling is hypermethylated in the chlorosis tissue. Some Ser-Thr kinases (*STKs*) that were identified as being receptor-like kinase/Pelle (*RLK*) proteins functioning as transmembrane or membrane effectors were also found to be up-regulated in the process of infection, however, they were misregulated in the recovery process.

### 3.5. Stability of Methylated and Demethylated DMRs across the Developmental Stages

We also investigated whether some of the methylated and demethylated DMRs were retained through the recovery process. Less than 3% of coding genes contained hypomethylated DMRs maintained during the developmental stage (11 d to 16 d), which were mainly involved in cell periphery and plasma membrane (Table S7). However, 35% of hypomethylated DEGs were maintained from severe chlorosis to recovery, and 24% of hypomethylated DEGs at 13 d and 13 dr were retained. However, a smaller fraction (13.9%) of hypermethylated DEGs was stable across the developmental stage. These results suggested that many sites are epigenetically unstable and continue to switch states (Table S6). 

**Figure 3:**
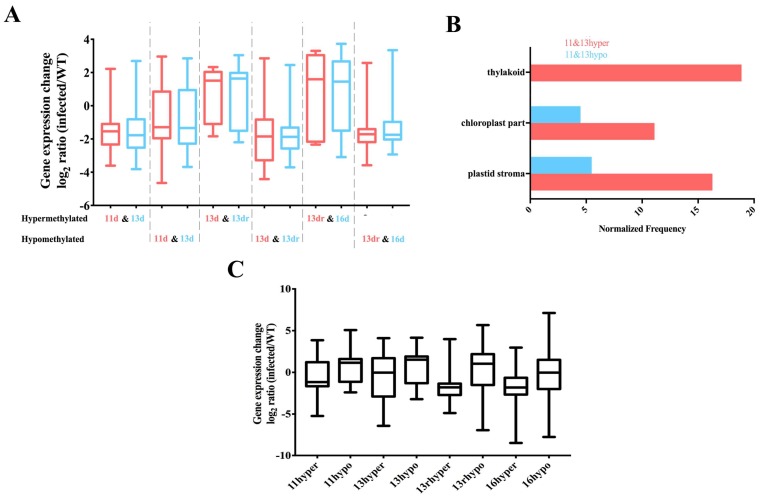
Stable DMRs across the developmental stages. (**A**) Expression levels of all protein-coding genes defined to be regulated at the nearby stages. Fold change were calculated from two biological replicates; (**B**) Gene ontology enrichment analysis of stable DMRs covered genes at 11 d and 13 d. Values along the x-axis represent the normalized frequency (relative frequency of the inquiry set/relative frequency of the reference set), and the enrichment cutoff was greater than 1.5-fold. The *p*-values of all the enriched pathways were less than 0.01; (**C**) Expression levels of all protein-coding genes found to be regulated at their promoter. Fold changes were calculated from two biological replicates.

Genes that were stably covered in DMRs did not follow the regular pattern (hypermethylated down-regulation and hypomethylated up-regulation) (Figure 3A). In the chlorosis tissue, DMRs are mainly stable in the regulation of cellular components, such as plastid stroma and chloroplasts (Figure 3B). Furthermore, genes containing DMRs from 13 d to 16 d were not enriched in the metabolic pathways but were instead involved in the ethylene-responsive transcription factor (LOC107828178), S-adenosylmethionine synthase (LOC107807060) and zinc finger protein (LOC107796498) [54]. S-adenosylmethionine synthase is an enzyme in the transformation from l-methionine (l-Met) into S-adenosyl-l-methionine (AdoMet, SAM), which is a precursor of ethylene and a methyl donor for methylation reactions and is activated in antiviral defense to clear virions.

### 3.6. DMRs in Promoter Alters Gene Expression

**Figure 4:**
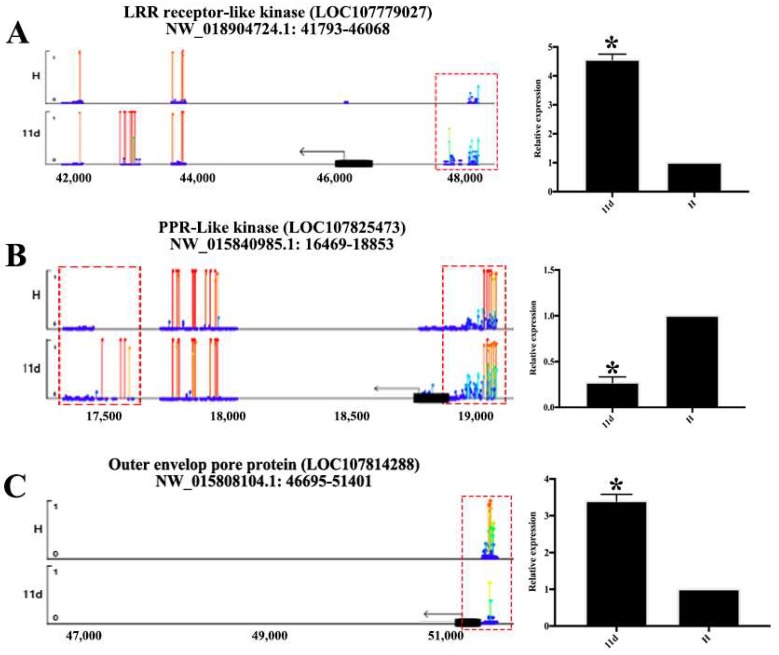
Methylation level across the nucleotides of the gene body and promoters of LRR receptor-like kinase (**A**); PPR-like kinase (**B**) and outer envelope pore protein (**C**). Dark lines and arrows showed the transcription direction and the start of gene body and to the right of them were promoter regions. Red box means significant changes in methylation level. The lollipop line (Y-axis) showed the methylation level. Expression levels are shown on the right of each figure. Expression levels are shown on the right. Significant differences are indicated (* *p* < 0.05) based on Student’s *t*-test.

Although the loss of DNA methylation in tobacco plants occurred within a relatively small proportion of the tobacco genome, DMRs concentrated in or near protein-coding gene promoters are more prone to altered gene expression [55]. To assess this effect, we also associated DMRs in the upstream region with the expression of nearby expressed genes. Hypomethylated DMRs located in the promoters of genes are associated with higher expression levels of certain genes than lower expressed genes (Figure 3C). These genes were not significantly associated with any particular biological processes. Rather, they appeared to be a random set of genes involved in different processes. Hence DNA methylation of the promoter is related to deregulated transcription of certain genes. Although some coding genes with hypermethylated or hypomethylated contexts in their promoters were not functionally characterized, there are still some characteristic genes that are regulated by this pattern (Figure 4, Table S8), such as the outer and inner envelope pore protein (LOC107814288 and LOC107764440) that promotes protein transport in both 11 d and 13 d [56,57] and proline-rich receptor-like protein kinase that promotes transmembrane signaling [58].

## 4. Discussion

DNA methylation is a common feature of eukaryotic epigenomes. Additionally, gene body methylation is occurred and strongly conserved between orthologues of plant species and affects a biased subset of long, slowly evolving genes, which shapes important features of plant genome evolution [59,60]. Many studies have improved our understanding of plant epigenome variation for defense response to geminivirus, including the *Beet severe curly top virus* (BSCTV) [61], *Tomato yellow leaf curl virus* (TYLCV) [37] and *Pea seed-borne mosaic virus* (PSbMV). Geminiviruses, like typical DNA viruses that display a double-strand viral genomic DNA [62,63], are challenged by the host DNA methylation-mediated gene silencing defense and must develop strategies to counter it [64,65]. Few studies have focused on the regulation of DNA methylation by RNA viruses [66]. In this study, we tested the effect of DNA methylation on the RNA virus invasion, and determined the role of hypomethylation in the process of antiviral defense; the most comprehensive analysis of single-base resolution methylomes in the tissues of tobacco plants during viral infection through RRBS. Tissues of *Nicotiana tabacum* during CMV infection showed patterns of methylation regulation and dynamically methylated regions regulation for viral defense. DMRs identified in the recovery stages are associated with gene expression and thereby influence biological regulation for viral clearance (Table S9). This is the first whole-genome methylation pattern of another model plant, supporting the further analysis of plant epigenetic change in response to viral pathogens. The positive effect of methyltransferase inhibition indicates the breakdown of methylation that prompts symptom recovery, partly suggesting the role of demethylation in defense gene activation and antiviral silencing. CHH methylation is usually mediated by RdDM pathway targeted in the short TEs and the edges of TEs [9,12,67]. In *Arabidopsis*, CMV infection with 2b deletion induced an enhanced population of 21-nt siRNAs and decreased the proportion of 24-nt siRNAs [68], as well as overexpressing 2b suppressed RdDM not only at the previously annotated loci directed by 24-nt siRNAs but also at a new set of loci associated with 21/22-nt siRNAs [69]. In our study, CHH hypomethylation was dominant at 16 d, together with the loss of 24-nt siRNA distribution across the gene body. Therefore, epigenetic regulation mediated by the small RNA pathway is conserved between *Nicotiana tabacum* and *Arabidopsis*.

Approximately 60% of coding genes that showed differential expression under methyltransferase inhibition were overlapped with those after CMV infection in this study. More than 70% of methylated and demethylated regions are basically dynamic, and 40% of them are related to the expression of coding genes, which affect metabolic pathways of plant defense response. This is different from *Arabidopsis* response to bacterial pathogens in which only DNA demethylation is part of the plant immune response [30]. Some defense genes were modulated through DNA methylation, and validated by the burst of reactive oxygen species (*ROS*) at 11, 13 d, and 13 dr (Figure S8). Fatty acid biosynthesis enzyme LOC107774635, which is homologous with suppressor of SA insensitive 2 (*SSI2*) in *Arabidopsis thaliana* [70], was down-regulated and hypermethylated at 11 and 13 d, but up-regulated and hypomethylated at 13 dr. This enzyme modulates *EDS5* and *PAD5* to activate salicylic acid- and jasmonic acid-mediated defense pathways [71]. Other hormone-related downstream genes, including phenylalanine lyase (*PAL*), defense with no dead (DND2), ethylene responsive factor (*ERF*)-like proteins, and pathogenesis related (*PR*)-like proteins, are also regulated at 11 d and 13 d, and deregulated at the recovery stage. This result suggests that, during viral infection, passive or active DNA demethylation releases the silencing marks of signaling genes, contributing to the transcriptional activation [72]. 

S-adenosylmethionine synthase (*SAMS*) catalyzes the conversion of l-methionine (l-Met) and ATP into SAM, which is a precursor of ethylene and a methyl donor for methylation reactions, thus possibly regulating DNA methylation and activating gene expression. It has been reported that *Rice dwarf virus* (RDV)-encoded protein Pns11 interacts with *OsSAMS1* to enhance its enzymatic activity, thus enhancing rice susceptibility to its infection [73]. Viral recovery is accelerated in the *SAMS*-silenced tobaccos compared with non-silenced control plants, with 72.5% of *SAMS*-silenced plants showing symptom recovery at 11 dpi, but only 10% of non-silenced plants recovering from viral infection. Thus, this result indicated that malfunction of *SAMS* activates DNA demethylation and downstream gene expression for antiviral defense (Figure S9).

Viral symptom recovery is also accompanied by the induction of RNA silencing. *AGO2* were found to be hypomethylated in all four stages and up-regulated only in 11 d and 13 d, however, no epigenetic modification was found in *AGO1*. This may suggest that no-mCG methylated contexts in the *AGO2* are functional in the balance of the degradation of viral genome and viral suppressors. The 2b-mediated inhibition of *AGO1* function and over-accumulated *AGO2* in hypomethylation may have a negative impact on CMV RNA and thus could be acting as a second defense layer to promote viral clearance in parts of infected leaves, as previously proposed in *Arabidopsis thaliana* [38,74].

**Figure 5:**
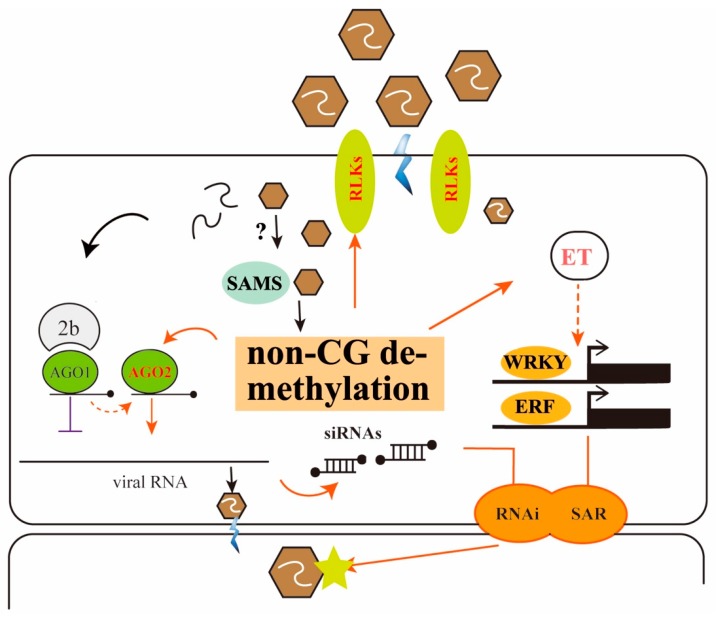
Proposed model for tobacco epigenetic change in response to CMV infection. Virions are recognized by hypomethylated plant *RLKs* that act as membrane-associated or transmembrane proteins. *SAMS* were depressed under unknown mechanisms to regulate DNA methylation. Over-accumulated *AGO2* proteins due to hypomethylation results in antiviral silencing and thus could be acting as a second defense layer. Key components of hormone signaling, such as *ERF* and *WRKY* family, are hypomethylated and significantly expressed and transmitted to developmental tissues as systematic resistance for recovery of new leaves. These two components of plant defense signals are transferred to new leaves for antiviral activity. Arrows in orange represent methylation regulation in response to viral infection. Arrows in dotted lines and question represent possible biological process. The red text indicates genes or pathways regulated by DNA methylation or demethylation.

The remodeling of DNA methylation patterns could be an active component of the tobacco response to viral infection or a consequence of viral clearance (Figure 5). *AGO2*-mediated RNAi is the most popular mechanism to degrade the genome of RNA virus for partial recovery (13 dr); however, repression of *SAMS* may result in methylation reprogramming through the plant genome, thus acting as the motivator to transmit hormone signals and initiate the immune response, in addition to viral-host interactions. Key components of hormone signaling, such as the *ERF* and *WRKY* family, are differentially expressed and transmitted to newly developed tissues as systematic resistance for recovery of new leaves (16 d). Our finding is different from a previous study that the defense response against *Pst* as a whole is negatively regulated by DNA methylation [29]. Our analysis was also based on the developmental stages and high resolution methylomes, which are exactly what the previous work suggests. The finding that global methylation patterns are drastically remodeled by viral infection could provide insight into how epigenetic changes influence transcriptional programs to adapt and battle RNA viruses. 

## Supplementary Materials

The following are available online at http://www.mdpi.com/1999-4915/10/8/402/s1, Figure S1: Methylation change during CMV infection. Figure S2 Methylomes of CMV infection on different stages. Figure S3: DMRs context enrichment and coverage. Context enrichment of DMRs was shown at 11 d (A), 13 d (B), 13 dr (C), and 16 d (D), respectively. (E) Genome coverage of identified CG hypermethylation and hypomethylation DMRs. (F) Genome coverage of identified CHG hypermethylation and hypomethylation DMRs. Figure S4: Distribution of sizes of unique CHH DMRs at infected stage (11 dpi and 13 dpi). Figure S5: Relative abundance of unique tobacco small RNAs according to their lengths. Figure S6 Venn diagram of hypomethylated DMRs covered genes (A) and hypermethylated DMRs covered genes (B). Figure S7: Lollipop diagram of meDEGs. Methylation levels across the gene body of some differentially expressed genes have been listed, such as *AGO2* (A), *SSI2* (B), *PR* (C), and *ERF* (D). DMRs in the gene body are presented with dotted lines. Figure S8: The burst of reactive oxygen species (ROS) of healthy and CMV-infected tobacco leaves at 11 dpi, 13 dpi, and 16 dpi. Figure S9: The incidences of recovered plants, which were determined by visual assessment of disease symptoms at 6–16 dpi. Table S1: Primers used in this study. Table S2: Reduced representation bisulfite sequencing summary. Table S3: Correlation between methylation level and the density of genes and TEs. Table S4: Differentially expressed genes targeted by differential methylation. Hypermethylated and Hypomethylated DEGs are listed as Upregulated or Downregulated. Table S5: Gene ontology (GO) enrichment of meDEGs at each stage. Table S6: GO enrichment analysis of shared meDEGs. Table S7: Description of hypermethylated and hypomethylated DMRs covered genes. Table S8: Differentially expressed gene descriptions of differential methylation on its promoter. Table S9: DMRs identified in this study. DMRs are separated by methylation direction (hyper-DMRs and hypo-DMRs) and developmental stages.
