# Supplementary material for: Epigenetic Changes in the Regulation of Nicotiana tabacum Response to Cucumber Mosaic Virus Infection and Symptom Recovery through Single-Base Resolution Methylomes
Source: Viruses. 2018 Jul 29;10(8):402. doi: 10.3390/v10080402 (PMC6115852; doi:10.3390/v10080402)
Supplement: Supplementary file 1 [file viruses-10-00402-s001.pdf]

Epigenetic changes in the regulation of *Nicotiana tabacum* response to *Cucumber mosaic virus* infection and symptom recovery through single-base resolution methylomes

Chenguang Wang[1, 2], Chaonan Wang[1, 2], Wenjie Xu[1, 2], Jingze Zou[2, 3], Yanhong Qiu[2], Jun Kong[1, 2], Yunshu Yang[4], Boyang Zhang[5], Pengyu Zhu[2], Wei Fu[2], Shuifang Zhu[1, 2].

[1] Colloge of Plant Protection, China Agricultural University, Bejing, 100083, China

[2] Institute of Plant Quarantine, Chinese Academy of Inspection and Quarantine, Beijing, 100083, China.

[3] Colloge of Biological Sciences, China Agricultural University, Bejing, 100083, China

[4] Beijing Academy of Food Sciences, Beijing, 100162, China

[5] Colloge of Food Science and Nurtional Engineering, China Agricultural University, Bejing, 100083, China

Running title: Epigenetic changes of Nicotiana tabacum through methylomes

Numbers of tables:0

Numbers of figures:6

Numbers of figures in color:5

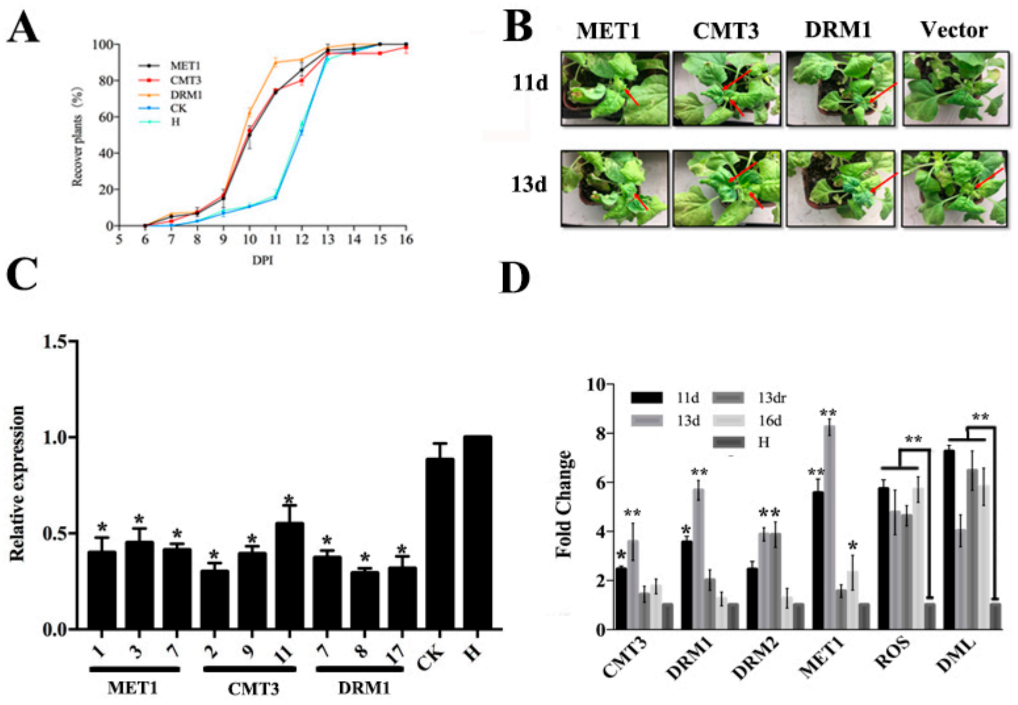

Figure S1. Methylation change during CMV infection. (A) The incidences of recovered plants in RNAi lines and WT plants, which were determined by visual assessment of disease symptoms at 6-16 dpi. Phenotypes of MET1 (black), CMT3 (red) and DRM1 (orange) RNAi lines after viral infection was observed in 40 individual plants. Mixture of pTRV1 and pTRV2 inoculation (CK) and healthy plants (H) were negative control. Means and standard deviations were obtained from three independent experiments. Y-axis indicates the rate of recovered plants on the whole evaluated plants. (B) Phenotypic comparison of plants at the recovery stage. Red arrows showed the recovery site. (C) Expression levels of MET1, CMT3 and DRM1 in WT (H), vector (CK) and RNAi lines. (10 days after inoculation). The average (± standard deviation) values from three biological replicates are shown. Significant differences are indicated (*p<0.05) based on Student's t-test. (D) Expression levels systematic tobacco tissues infected with CMV-M at 11d, 13d, 13dr and 16d. Data are the mean values ± SD of three biological replicates. Significant differences are indicated (*p<0.05, **p<0.01) based on Student's t-test.

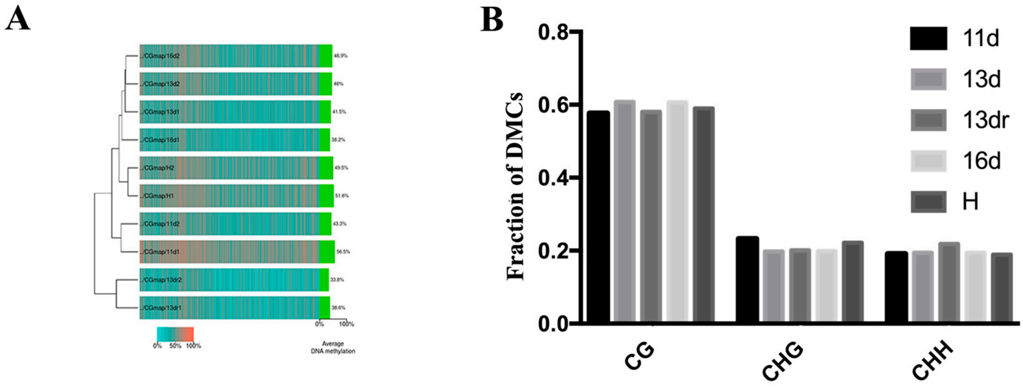

Figure S2. Methylomes of CMV infection on different stages. (A) Heatmaps of methylomes and average methylation rate in each sample. Each stage was tested in duplicate. (B) Context enrichment of DMCs at each stage.

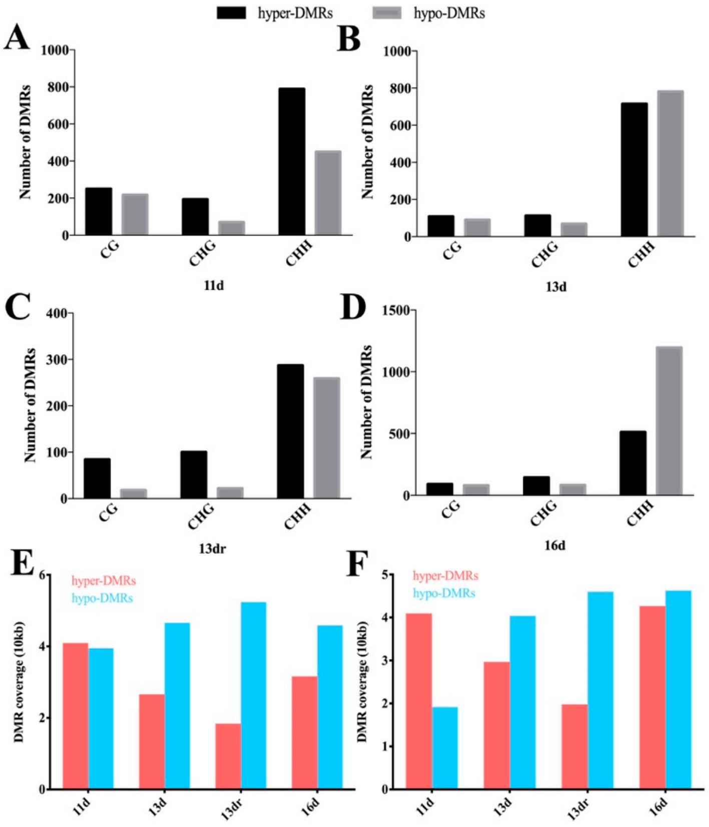

Figure S3. DMRs context enrichment and coverage. Context enrichment of DMRs were shown at 11d (A), 13d (B), 13dr (C) and 16d (D), respectively. (E) Genome coverage of identified CG hypermethylation and hypomethylation DMRs. (F) Genome coverage of identified CHG hypermethylation and hypomethylation DMRs.

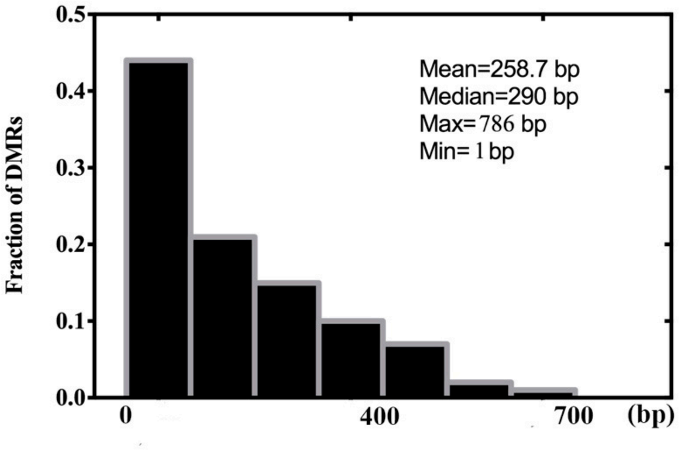

Figure S4. Distribution of sizes of unique CHH DMRs at infected stage (11dpi and 13dpi). Each lane in the X-axis indicate 100bp range of CHH DMRs. The y-axis indicates the rate of specialized DMRs in all CHH DMRs.

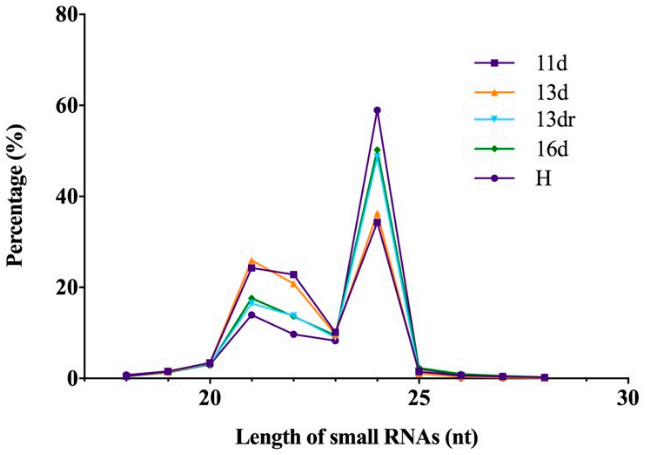

Figure S5. Relative abundance of total tobacco small RNAs according to their lengths.

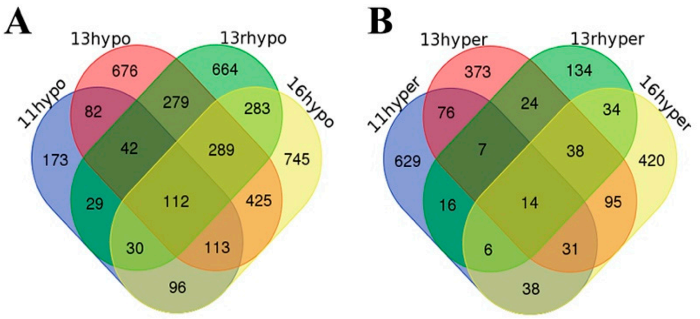

Figure S6. Venn diagram of hypomethylated DMRs covered genes (A) and hypermethylated DMRs covered genes (B).

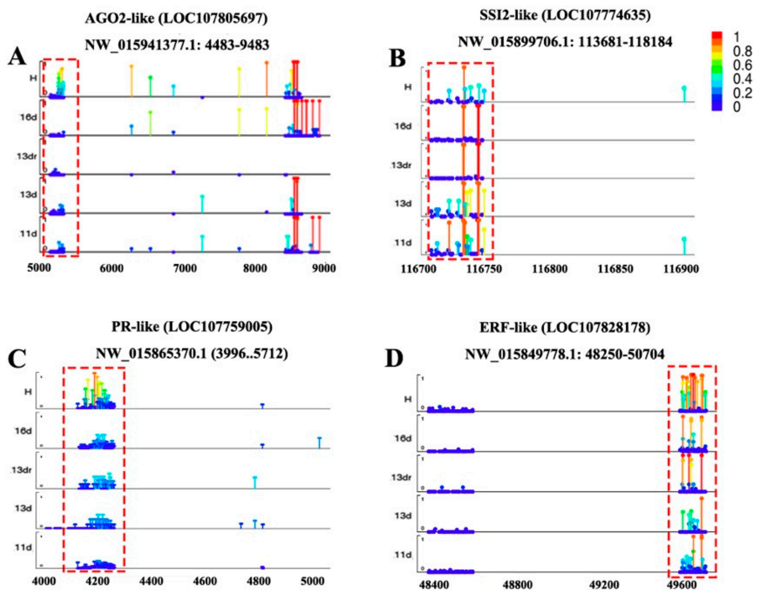

Figure S7. Lollipop diagram of meDEGs. Methylation level across the gene body of some differentially expressed genes have been listed, such as AGO2 (A), SSI2 (B), PR (C) and ERF (D). DMRs in the gene body are presented with dotted lines.

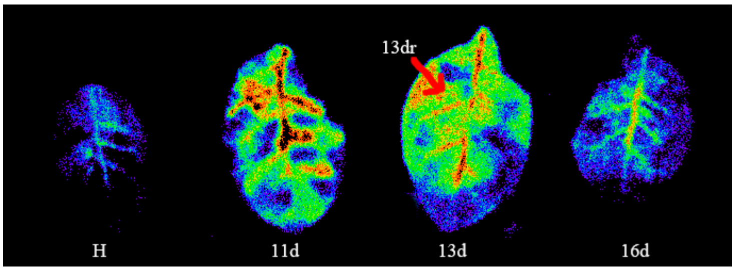

Figure S8. The burst of reactive oxygen species (ROS) of healthy and CMV-infected tobacco leaves at 11dpi, 13dpi and 16dpi. Color indicates the ROS production on the specific site of tissue.

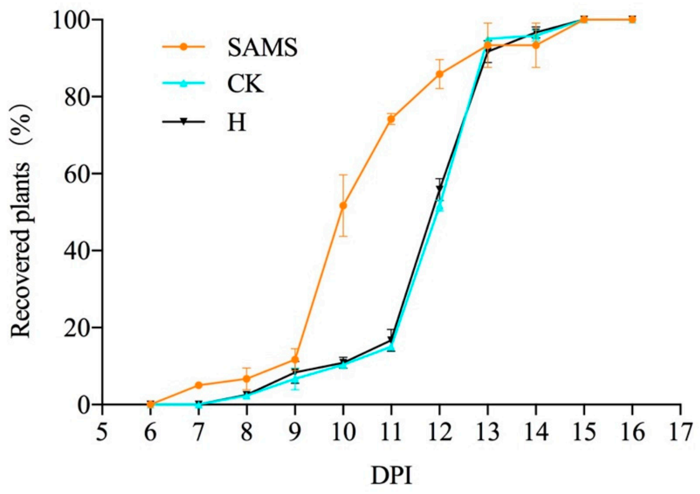

Figure S9. The incidences of recovered plants, which were determined by visual assessment of disease symptoms at 6-16 dpi. 40 individual plants were evaluated. SAMS, CK and H indicate SAMS-silenced, vector-silenced and non-silenced plant. Means and standard deviations were obtained from three independent experiments. Y-axis indicates the rate of recovered plants on the whole evaluated plants.

Table S1 Primers used in this study.

Experiment	Primer name	Sequence (5'-3')	Note
qRT-PCR	MET1-F	AGAGGAAGAGGTTGTGGCTATT	For expression mesurement in *Nicotiana tabacum*
	MET1-R	GTTGTGGTATTCCTTCCGAGTTATG	
	CMT3-F	TACAAACTGACAGGGCCAATAA	
	CMT3-R	CATTGCCAGCGCTAAAGAATAC	
	DRM1-F	ACTGCTCTCGTCATTCAAGTAG	
	DRM1-R	CTGCTTCCTGGGCACTATTAT	
	DRM2-F	CGGTGGTGAAGTTGCTCTTTA	
	DRM2-R	CCCACCAGCTTCTCACAATATC	
	ROS1-F	CCAGATGATCCATGCTCCTATC	
	ROS1-R	CCTCTTGGGAGTTGCACTTTA	
	DML2-F	AGAGAGGAGGTCAGTGTAGTT	
	DML2-R	TGCTCTGTCTAATGTGCTATCC	
	NbMET-F	GCTTGGTATGAACCAGTCCTAAAG	For expression mesurement in *RNAi* lines
	NbMET-R	TGAAACCCGTTTAATGACATCTCC	
	NbCMT-F	ACGGAGACCCAAAGGAAATAAATAAG	
	NbCMT-R	GTAGGCAAGTTAGGATGCAAAGAG	
	NbDRM-F	TCAGGCAGCAGTGTGAGATC	
	NbDRM-R	TAGGAGGATCAGAATTTGCAACAAG	
TRV-VIGS	NbMET-TRV-F	CCG**GAATTC**AGTGATTATCCTTTGCCTGCATATT	pTRV2
	NbMET-TRV-R	CGG**GGTACC**CTGGTTCATACCAAGCAGCATAC	
	NbCMT-TRV-F	GC**TCTAGA**GTTGGAACTCTTTGCATCCTAACT	
	NbCMT-TRV-R	CGG**GGTACC**ACAAGTCTGCCCAAAGCATATC	
	NbDRM-TRV-F	GC**TCTAGA**TCAGTTTCGCCGCATCTAAAG	

NbDRM-TRV-R	CGG**GGTACC**CACTGAAACCCA TAGCCAGAAG

Table S2 Reduced representation bisulfite sequencing summary.

Stages	Total Mapped bases (Gb)	Coverage width	Bisulfite conversion rate
11d rep1	9.86	74.72%	99.69%
11d rep2	7.23	70.67%	99.81%
13d rep1	6.65	75.71%	99.60%
13d rep2	8.00	72.00%	99.62%
13dr rep1	8.06	82.25%	99.57%
13dr rep2	7.25	74.83%	99.62%
16d rep1	7.48	60.36%	99.64%
16d rep2	9.45	61.57%	99.53%
H rep1	7.99	75.82%	99.49%
H rep2	7.02	71.61%	100.00%

Table S3 Correlation between methylation level and the density of genes and TEs.

Stages	Sequence context	Correlation to gene density	Correlation to TE density
11d	CG	0.32	0.11
	CHG	-0.23	0.42
	CHH	0.24	0.31
13d	CG	-0.15	-0.21
	CHG	0.27	0.38
	CHH	-0.17	-0.40
13dr	CG	-0.62	0.05
	CHG	-0.31	0.20
	CHH	0.27	-0.35
16d	CG	0.33	-0.52
	CHG	-0.31	-0.30
	CHH	-0.42	-0.24

Table S4 Differentially expressed genes targeted by differential methylation. Hypermethylated and Hypomethylated DEGs are listed as Upregulated or Downregulated.

11days post inoculation

Hypermethylated		Hypomethylated	
upregulated	downregulated	upregulated	downregulated
LOC107760225	LOC107814768	LOC107824127	LOC107830977
LOC107803921	LOC107827304	LOC107782199	LOC107767653
LOC107810686	LOC107830453	LOC107805036	LOC107774073
LOC107814147	LOC107764337	LOC107814288	LOC107785315
LOC107814853	LOC107771222	LOC107815681	LOC107803361
LOC107816085	LOC107771890	LOC107823813	LOC107811785

LOC107819773	LOC107774251	LOC107824178	LOC107814122
LOC107820748	LOC107776836	LOC107824328	LOC107816302
LOC107822407	LOC107784161	LOC107825404	LOC107817057
LOC107823987	LOC107798764	LOC107825457	LOC107817209
LOC107826438	LOC107806301	LOC107825947	LOC107817323
LOC107827090	LOC107810736	LOC107826315	LOC107817883
LOC107827494	LOC107814152	LOC107829385	LOC107818849
LOC107763110	LOC107819449	LOC107830577	LOC107820326
LOC107763560	LOC107820174	LOC107830687	LOC107820495
LOC107763928	LOC107820949	LOC107831213	LOC107820497
LOC107764219	LOC107821052	LOC107759005	LOC107821481
LOC107769268	LOC107821919	LOC107763927	LOC107822452
LOC107773125	LOC107822207	LOC107769853	LOC107822634
LOC107773793	LOC107823823	LOC107782584	LOC107826148
LOC107776309	LOC107825473	LOC107784033	LOC107828178
LOC107779906	LOC107826672	LOC107785592	LOC107830408
LOC107782668	LOC107828560	LOC107789149	LOC107763047
LOC107783939	LOC107829799	LOC107789632	LOC107765365
LOC107784363	LOC107830375	LOC107791455	LOC107768861
LOC107785312	LOC107830987	LOC107794504	LOC107770411
LOC107786177	LOC107831545	LOC107794813	LOC107771658
LOC107786749	LOC107760509	LOC107795479	LOC107773045
LOC107788198	LOC107761637	LOC107797969	LOC107773232
LOC107789908	LOC107762260	LOC107800540	LOC107773564
LOC107790583	LOC107766322	LOC107801990	LOC107773801
LOC107790852	LOC107769103	LOC107803886	LOC107773808
LOC107791466	LOC107769475	LOC107805697	LOC107775280
LOC107792081	LOC107773465	LOC107808758	LOC107776126
LOC107795121	LOC107774022	LOC107811733	LOC107776229
LOC107795779	LOC107774635	LOC107813055	LOC107777350
LOC107799728	LOC107775100		LOC107778365
LOC107801401	LOC107775219		LOC107778620
LOC107801465	LOC107779902		LOC107779140
LOC107804035	LOC107779981		LOC107780379
LOC107805431	LOC107782030		LOC107782450
LOC107811953	LOC107782384		LOC107783657
LOC107812388	LOC107784622		LOC107783891
	LOC107785376		LOC107788394
	LOC107786588		LOC107789344
	LOC107786762		LOC107790956
	LOC107786858		LOC107792197
	LOC107788304		LOC107792758
	LOC107790109		LOC107793272

	LOC107790272		LOC107797466
	LOC107790339		LOC107797839
	LOC107790581		LOC107798677
	LOC107793082		LOC107799294
	LOC107793156		LOC107799901
	LOC107793178		LOC107799995
	LOC107793358		LOC107800214
	LOC107796817		LOC107800285
	LOC107798594		LOC107801215
	LOC107799192		LOC107802063
	LOC107801464		LOC107802364
	LOC107801762		LOC107802784
	LOC107803080		LOC107803242
	LOC107804565		LOC107803843
	LOC107805241		LOC107805414
	LOC107805303		LOC107807034
	LOC107805587		LOC107807060
	LOC107805847		LOC107808474
	LOC107807043		LOC107809852
	LOC107807149		LOC107810527
	LOC107810528		LOC107810529
	LOC107811505		
	LOC107813123		

13days post inoculation

Hypermethylated		Hypomethylated	
upregulated	downregulated	upregulated	downregulated
LOC107814855	LOC107820790	LOC107818180	LOC107817253
LOC107828516	LOC107830977	LOC107821292	LOC107797025
LOC107832293	LOC107761657	LOC107785486	LOC107800556
LOC107762301	LOC107767653	LOC107790647	LOC107814768
LOC107764701	LOC107770844	LOC107795812	LOC107761618
LOC107779149	LOC107774251	LOC107805036	LOC107761677
LOC107801011	LOC107785989	LOC107810686	LOC107763187
LOC107801714	LOC107810736	LOC107814826	LOC107766483
LOC107827241	LOC107812028	LOC107815388	LOC107768720
LOC107814330	LOC107813664	LOC107815523	LOC107774020
LOC107814785	LOC107816078	LOC107815568	LOC107774216
LOC107815479	LOC107817323	LOC107815664	LOC107776829
LOC107815679	LOC107817573	LOC107816468	LOC107782038
LOC107815681	LOC107817815	LOC107817191	LOC107783615
LOC107816214	LOC107818087	LOC107819279	LOC107785315

LOC107816842	LOC107819987	LOC107821291	LOC107787480
LOC107817420	LOC107827754	LOC107821687	LOC107793232
LOC107817646	LOC107829202	LOC107821888	LOC107803361
LOC107818030	LOC107832181	LOC107823405	LOC107804496
LOC107820817	LOC107832355	LOC107823578	LOC107808445
LOC107822227	LOC107832468	LOC107823726	LOC107810756
LOC107822407	LOC107832794	LOC107823757	LOC107811785
LOC107822435	LOC107761838	LOC107823813	LOC107811850
LOC107823241	LOC107764263	LOC107823966	LOC107812546
LOC107823988	LOC107768296	LOC107824178	LOC107814048
LOC107825404	LOC107769475	LOC107826315	LOC107814122
LOC107825457	LOC107770931	LOC107827024	LOC107814720
LOC107826669	LOC107772805	LOC107827090	LOC107814796
LOC107827045	LOC107773564	LOC107827494	LOC107815199
LOC107760007	LOC107773808	LOC107827529	LOC107815457
LOC107763032	LOC107774022	LOC107829208	LOC107815751
LOC107828153	LOC107774398	LOC107829374	LOC107816302
LOC107830474	LOC107774635	LOC107829500	LOC107816351
LOC107830577	LOC107775100	LOC107829525	LOC107816530
LOC107830653	LOC107776229	LOC107830299	LOC107817057
LOC107830754	LOC107777466	LOC107830546	LOC107817209
LOC107830916	LOC107779056	LOC107830687	LOC107817883
LOC107832462	LOC107780379	LOC107831213	LOC107819136
LOC107758959	LOC107781119	LOC107831483	LOC107819377
LOC107759529	LOC107782384	LOC107831702	LOC107819784
LOC107764072	LOC107782430	LOC107832391	LOC107820490
LOC107765822	LOC107783257	LOC107758934	LOC107820616
LOC107766358	LOC107783821	LOC107759641	LOC107820682
LOC107770715	LOC107784622	LOC107759873	LOC107821406
LOC107770994	LOC107785376	LOC107762641	LOC107821919
LOC107771090	LOC107785488	LOC107763344	LOC107822634
LOC107772214	LOC107786772	LOC107763475	LOC107822770
LOC107772515	LOC107788304	LOC107763560	LOC107822911
LOC107772606	LOC107789905	LOC107763885	LOC107823398
LOC107774457	LOC107790339	LOC107764502	LOC107823775
LOC107774987	LOC107792531	LOC107766766	LOC107824273
LOC107776224	LOC107792758	LOC107769471	LOC107824395
LOC107778382	LOC107793872	LOC107769853	LOC107825176
LOC107778447	LOC107795020	LOC107770758	LOC107825332
LOC107779946	LOC107797795	LOC107772127	LOC107825949
LOC107780165	LOC107798594	LOC107773282	LOC107826148
LOC107780214	LOC107798677	LOC107774049	LOC107827041
LOC107782055	LOC107800903	LOC107775904	LOC107828123

LOC107783336	LOC107801762	LOC107780515	LOC107828178
LOC107784558	LOC107804443	LOC107780535	LOC107828669
LOC107785366	LOC107805847	LOC107782668	LOC107828826
LOC107785865	LOC107806382	LOC107783614	LOC107830375
LOC107786276	LOC107806780	LOC107784033	LOC107830408
LOC107787047	LOC107807034	LOC107785236	LOC107831056
LOC107790362	LOC107807891	LOC107785687	LOC107832235
LOC107790583	LOC107808119	LOC107786426	LOC107832321
LOC107791039	LOC107809852	LOC107787409	LOC107759633
LOC107792369	LOC107810310	LOC107787644	LOC107759735
LOC107793424	LOC107810474	LOC107789151	LOC107760984
LOC107794052	LOC107810527	LOC107789181	LOC107761087
LOC107794479	LOC107810529	LOC107789715	LOC107761637
LOC107796215	LOC107810528	LOC107790143	LOC107761678
LOC107796498	LOC107811439	LOC107790489	LOC107761969
LOC107798610	LOC107811505	LOC107790543	LOC107763092
LOC107799869	LOC107812441	LOC107790881	LOC107763257
LOC107800018	LOC107812786	LOC107791409	LOC107763893
LOC107802273	LOC107813196	LOC107794504	LOC107764974
LOC107803493		LOC107794992	LOC107765050
LOC107804454		LOC107795025	LOC107765074
LOC107804602		LOC107795365	LOC107766400
LOC107805784		LOC107797548	LOC107766680
LOC107806520		LOC107797716	LOC107767286
LOC107806705		LOC107798570	LOC107767727
LOC107807885		LOC107800100	LOC107769462
LOC107808012		LOC107800421	LOC107769616
LOC107808821		LOC107800540	LOC107770135
LOC107810155		LOC107800889	LOC107770767
LOC107812018		LOC107801999	LOC107771658
LOC107812507		LOC107803628	LOC107771747
		LOC107803671	LOC107772158
		LOC107803724	LOC107773687
		LOC107803886	LOC107774509
		LOC107804287	LOC107774570
		LOC107805697	LOC107774919
		LOC107805967	LOC107776409
		LOC107807258	LOC107777394
		LOC107808758	LOC107778137
		LOC107809847	LOC107778365
		LOC107809961	LOC107778634
		LOC107810488	LOC107778999
		LOC107810574	LOC107779140

		LOC107810787	LOC107779814
		LOC107810900	LOC107779945
		LOC107811265	LOC107779981
		LOC107812303	LOC107780410
			LOC107782079
			LOC107782318
			LOC107782344
			LOC107782408
			LOC107782450
			LOC107783657
			LOC107783891
			LOC107785829
			LOC107786565
			LOC107786858
			LOC107787062
			LOC107787165
			LOC107787460
			LOC107787712
			LOC107788857
			LOC107788890
			LOC107789344
			LOC107789676
			LOC107790378
			LOC107790598
			LOC107790955
			LOC107790956
			LOC107791321
			LOC107792480
			LOC107792668
			LOC107793178
			LOC107793358
			LOC107793365
			LOC107793983
			LOC107794375
			LOC107795036
			LOC107795380
			LOC107795677
			LOC107795798
			LOC107796817
			LOC107797139
			LOC107797466
			LOC107797595
			LOC107797839

			LOC107798109
			LOC107798399
			LOC107799294
			LOC107799374
			LOC107799995
			LOC107800300
			LOC107801215
			LOC107801464
			LOC107802364
			LOC107802630
			LOC107803038
			LOC107803242
			LOC107805027
			LOC107805404
			LOC107805587
			LOC107806344
			LOC107806565
			LOC107807060
			LOC107808408
			LOC107809034
			LOC107809161
			LOC107811551

13days post inoculation (recovery site)

Hypermethylated		Hypomethylated	
upregulated	downregulated	upregulated	downregulated
LOC107787819	LOC107814768	LOC107832293	LOC107817253
LOC107815479	LOC107827263	LOC107802932	LOC107766483
LOC107816085	LOC107774495	LOC107814975	LOC107774020
LOC107768592	LOC107783615	LOC107822227	LOC107776692
LOC107770715	LOC107812028	LOC107822960	LOC107793232
LOC107772214	LOC107816401	LOC107830577	LOC107811785
LOC107775293	LOC107817773	LOC107764219	LOC107814796
LOC107789092	LOC107820476	LOC107767222	LOC107816302
LOC107790362	LOC107821979	LOC107774049	LOC107823775
LOC107791039	LOC107831545	LOC107778840	LOC107824096
LOC107796498	LOC107771432	LOC107785062	LOC107826148
LOC107804544	LOC107773801	LOC107794052	LOC107827558
LOC107808012	LOC107777688	LOC107794504	LOC107828178
	LOC107780867	LOC107797281	LOC107830355
	LOC107790956	LOC107800540	LOC107761637
	LOC107792531	LOC107800889	LOC107761678

	LOC107798424	LOC107801990	LOC107762220
	LOC107803242	LOC107810900	LOC107763893
	LOC107805587		LOC107768131
	LOC107807034		LOC107768721
	LOC107809680		LOC107773045
			LOC107775428
			LOC107775473
			LOC107777998
			LOC107779056
			LOC107781010
			LOC107782450
			LOC107783257
			LOC107784516
			LOC107785405
			LOC107787019
			LOC107787803
			LOC107795036
			LOC107795798
			LOC107799294
			LOC107800214
			LOC107800941
			LOC107801215
			LOC107801291
			LOC107802630
			LOC107803843
			LOC107805852
			LOC107807060
			LOC107809769
			LOC107810532
			LOC107810888
			LOC107812289
			LOC107813068

16days post inoculation

Hypermethylated		Hypomethylated	
upregulated	downregulated	upregulated	downregulated
LOC107814855	LOC107813894	LOC107822727	LOC107800556
LOC107814985	LOC107820790	LOC107824127	LOC107832338
LOC107832258	LOC107830453	LOC107772802	LOC107767560
LOC107759759	LOC107768348	LOC107805036	LOC107767653
LOC107781766	LOC107774495	LOC107814266	LOC107774020
LOC107787819	LOC107798764	LOC107815568	LOC107804496

LOC107807853	LOC107810736	LOC107818832	LOC107810756
LOC107810686	LOC107813875	LOC107819666	LOC107811785
LOC107814975	LOC107816401	LOC107820326	LOC107813721
LOC107815523	LOC107817083	LOC107824178	LOC107814122
LOC107815679	LOC107820616	LOC107824328	LOC107814438
LOC107816085	LOC107820951	LOC107825601	LOC107814625
LOC107820817	LOC107822634	LOC107827090	LOC107815905
LOC107821772	LOC107824576	LOC107832837	LOC107816532
LOC107822227	LOC107824862	LOC107761664	LOC107817057
LOC107822960	LOC107831545	LOC107763475	LOC107817209
LOC107823241	LOC107832355	LOC107764502	LOC107817883
LOC107825263	LOC107759334	LOC107764582	LOC107818849
LOC107825404	LOC107767049	LOC107767437	LOC107820476
LOC107825453	LOC107772038	LOC107767998	LOC107823775
LOC107832462	LOC107772805	LOC107768386	LOC107825976
LOC107759209	LOC107773564	LOC107769471	LOC107826148
LOC107762866	LOC107775473	LOC107770758	LOC107828123
LOC107767666	LOC107777998	LOC107771462	LOC107828178
LOC107774577	LOC107780867	LOC107776184	LOC107829234
LOC107778447	LOC107781010	LOC107777196	LOC107759407
LOC107781553	LOC107782384	LOC107780515	LOC107759629
LOC107783176	LOC107783257	LOC107782584	LOC107760154
LOC107785532	LOC107783266	LOC107786005	LOC107760583
LOC107788198	LOC107784928	LOC107788674	LOC107760595
LOC107790881	LOC107785928	LOC107792369	LOC107761637
LOC107791039	LOC107786137	LOC107794504	LOC107763092
LOC107791455	LOC107787803	LOC107797281	LOC107765459
LOC107793424	LOC107790955	LOC107798610	LOC107766400
LOC107794052	LOC107793862	LOC107800421	LOC107766884
LOC107794479	LOC107794659	LOC107800889	LOC107767661
LOC107794992	LOC107796710	LOC107801990	LOC107767963
LOC107796498	LOC107797466	LOC107803724	LOC107770767
LOC107797059	LOC107799294	LOC107804602	LOC107771167
LOC107798680	LOC107799876	LOC107806624	LOC107773045
LOC107798880	LOC107800462		LOC107773801
LOC107799869	LOC107800903		LOC107774426
LOC107800100	LOC107802630		LOC107776409
LOC107802501	LOC107805241		LOC107777040
LOC107803523	LOC107805284		LOC107777394
LOC107804287	LOC107806163		LOC107778620
LOC107806072	LOC107807034		LOC107779140
LOC107808012	LOC107807885		LOC107779981
LOC107808758	LOC107807891		LOC107780047

LOC107808821	LOC107809888		LOC107780410
LOC107812145	LOC107810474		LOC107781308
	LOC107810529		LOC107782079
	LOC107813104		LOC107782318
			LOC107782450
			LOC107785578
			LOC107786611
			LOC107786772
			LOC107787868
			LOC107788472
			LOC107788959
			LOC107792708
			LOC107793358
			LOC107794375
			LOC107794413
			LOC107795036
			LOC107800214
			LOC107800285
			LOC107801215
			LOC107802364
			LOC107803242
			LOC107805587
			LOC107807060
			LOC107812289

Table S5 Gene ontology (GO) enrichment of meDEGs at each stage.

Stages	GO accession	GO term	Normalized Frequency[1]	P value
11d	GO:0009765	photosynthesis, light harvesting	27.87420147	1.60E-09
	GO:0018298	protein-chromophore linkage	26.66227967	2.20E-09
	GO:0015995	chlorophyll biosynthetic process	12.56623837	7.00E-05
	GO:0015979	photosynthesis	9.089413524	2.70E-10
	GO:0015994	chlorophyll metabolic process	9.018124006	0.0003
	GO:0006778	porphyrin-containing compound metabolic process	7.902479799	0.00054
	GO:0006091	generation of precursor metabolites and energy	5.054113454	6.90E-06
	GO:0009617	response to bacterium	4.866924067	1.80E-06
	GO:0042742	defense response to bacterium	4.763811269	2.90E-05
	GO:0051186	cofactor metabolic process	4.729298965	1.30E-05
	GO:0009966	regulation of signal transduction	4.727562805	0.00087
	GO:0023051	regulation of signaling	4.645700246	0.00096
	GO:0010646	regulation of cell communication	4.586139986	0.001
	GO:0009607	response to biotic stimulus	2.936468152	2.90E-06
	GO:0035556	intracellular signal transduction	2.787420147	0.0015
	GO:0098542	defense response to other organism	2.784442134	0.00017
	GO:0009628	response to abiotic stimulus	2.653701179	1.30E-07
	GO:0006952	defense response	2.545345345	1.30E-05
	GO:0009605	response to external stimulus	2.422694502	4.50E-05
	GO:0009725	response to hormone	2.255913301	0.00019
	GO:0009719	response to endogenous stimulus	2.212876849	0.00018
	GO:0006950	response to stress	2.142640416	1.70E-07
13d	GO:0042742	defense response to bacterium	4.855675398	3.40E-09

	GO:0009735	response to cytokinin	4.803210947	1.20E-06
	GO:0009617	response to bacterium	4.606435412	3.60E-10
	GO:0009611	response to wounding	4.320676391	3.90E-05
	GO:0009753	response to jasmonic acid	4.167052342	5.40E-05
	GO:0009814	defense response, incompatible interaction	4.136412251	0.00048
	GO:0046686	response to cadmium ion	3.416861432	5.80E-05
	GO:0016051	carbohydrate biosynthetic process	3.198395438	0.0002
	GO:0010038	response to metal ion	2.761430216	0.00023
	GO:0002376	immune system process	2.752626039	0.0013
	GO:0044283	small molecule biosynthetic process	2.632326326	5.70E-05
	GO:0006979	response to oxidative stress	2.58715998	0.001
	GO:0044281	small molecule metabolic process	2.49514327	3.30E-09
	GO:0009725	response to hormone	2.491033742	2.40E-09
	GO:0043207	response to external biotic stimulus	2.320759349	4.10E-06
	GO:0044723	single-organism carbohydrate metabolic process	2.294258018	0.0012
	GO:0043436	oxoacid metabolic process	2.248409537	8.40E-05
	GO:0009755	hormone-mediated signaling pathway	2.093677334	0.0012
	GO:0009605	response to external stimulus	2.074307028	9.30E-06
	GO:0006952	defense response	1.995714723	3.60E-05
16d	GO:0009523	photosystem II	11.3484315	0.00011
	GO:0009521	photosystem	8.393110795	0.00041
	GO:0009505	plant-type cell wall	7.483640585	3.30E-09
	GO:0015979	photosynthesis	6.369475386	5.90E-06
	GO:0000785	chromatin	6.344398712	0.0014
	GO:0009526	plastid envelope	5.470361322	2.70E-11

GO:0048046	apoplast	5.305274972	1.00E-07
GO:0009941	chloroplast envelope	4.918753303	3.30E-09
GO:0046686	response to cadmium ion	4.698184469	7.30E-05
GO:0009570	chloroplast stroma	4.573111179	1.10E-08
GO:0005618	cell wall	4.565091424	2.60E-08
GO:0009532	plastid stroma	4.372225159	2.40E-08
GO:0031969	chloroplast membrane	4.35534398	0.0014
GO:0009266	response to temperature stimulus	4.324178728	3.00E-06
GO:0044434	chloroplast part	4.315443014	2.90E-14
GO:0042170	plastid membrane	4.256732419	0.0016
GO:0044435	plastid part	4.208669166	6.20E-14
GO:0031975	envelope	3.443327506	9.60E-08

[1]Normalized frequency = relative frequency of the inquiry set / relative frequency of the reference set.

Table S6 GO enrichment analysis of shared meDEGs.

**GO term**	**Description**	**Normalized Frequency[1]**	**P value**	**Accession**
GO:0015979	photosynthesis	15.36	1.40E-09	
GO:0009617	response to bacterium	8.81	2.30E-07	
GO:0050896	response to stimulus	2.24	2.80E-07	
GO:0009628	response to abiotic stimulus	3.46	2.90E-06	11d and 13d
GO:0042742	defense response to bacterium	8.78	4.20E-06	
GO:0009987	cellular process	1.58	5.40E-06	
GO:0043207	response to external biotic stimulus	4.17	1.30E-05	

GO:0006950	response to stress	2.55	1.50E-05	
GO:0009607	response to biotic stimulus	4.03	1.80E-05	
GO:0009719	response to endogenous stimulus	3.36	3.10E-05	
GO:0006952	defense response	3.47	4.20E-05	
GO:0009735	response to cytokinin	9.55	4.50E-05	
GO:0009605	response to external stimulus	3.44	4.70E-05	
GO:0009725	response to hormone	3.33	6.60E-05	
GO:0098542	defense response to other organism	4.15	0.00015	
GO:0048046	apoplast	1.86	0.00032	13dr and 16d

[1] Normalized frequency = the relative frequency of the inquiry set/relative frequency of the reference set.

Table S7 Description of hypermethylated and hypomethylated DMRs covered genes.

Hypomethylated				Hypermethylated	
Gene ID	Description	Gene ID	Description	Gene ID	Description
LOC107800826	ADP-ribosylation factor GTPase-activating protein	LOC107823313	enoyl-CoA hydratase 1,	LOC107776224	GATA type zinc finger protein asd-4
LOC107765114	ras-related protein Rab11D	LOC107792896	cytochrome P450 98A2	LOC107811505	photosystem II protein
LOC107814446	uncharacterized	LOC107824713	peptidyl-prolyl isomerase CYP18-1	LOC107829373	polyubiquitin
LOC107782145	uncharacterized	LOC107795060	uncharacterized	LOC107775705	aquaporin TIP1-1

LOC107774928	uncharacterized	LOC107820765	uncharacterized	LOC107817493	PAN domain protein
LOC107760969	basic endochitinase	LOC107828416	PRA1 family protein B4	LOC107799329	uncharacterized
LOC107815094	galacturonosyltransferase 11	LOC107825396	CDPK-related kinase 4	LOC107801762	elongation factor P
LOC107803843	BAHD acyltransferase DCR	LOC107802364	WRKY transcription factor 11	LOC107763560	60S ribosomal protein L12
LOC107777103	bifunctional riboflavin kinase/FMN phosphatase	LOC107793114	protein CLT3, chloroplastic	LOC107809765	glutamate receptor 2.7
LOC107761266	uncharacterized	LOC107781457	glucan endo-1,3-β-glucosidase 11	LOC107807453	replication protein A
LOC107779017	uncharacterized	LOC107800887	TMV resistance protein N	LOC107781433	uncharacterized
LOC107789393	WSC domain-containing protein ARB_07867	LOC107807848	heat shock protein 82	LOC107784622	chlorophyll a-b binding protein 8
LOC107803650	uncharacterized	LOC107798587	blue copper protein	LOC107810528	chlorophyll a-b binding protein 21
LOC107807060	S-adenosylmethionine synthase 2	LOC107831629	auxin response factor 2		
LOC107766149	inorganic phosphate transporter 1-3	LOC107778585	ABC transporter G family member		
LOC107787803	extensin-3	LOC107774689	GTPase Der		
LOC107800079	membrane-associated kinase regulator 2	LOC107769962	uncharacterized		
LOC107785175	E3 ubiquitin-protein ligase RHA2A	LOC107815833	caffeoylshikimate esterase		
LOC107826627	late blight resistance protein R1B-12	LOC107782450	uncharacterized		

LOC107793178	uncharacterized	LOC107769118	DNA/RNA polymerases superfamily protein
LOC107817057	arogenate dehydratase 6,	LOC107788033	tRNA N6-adenosine threonylcarbamoyltransferase
LOC107827529	glycine-rich RNA-binding protein 3	LOC107809245	uncharacterized
LOC107761857	pumilio homolog 1	LOC107793388	basic 7S globulin 2
LOC107771248	ATP-dependent Clp protease proteolytic subunit-related protein 1	LOC107778365	F-box protein
LOC107817883	transcription factor MYC2	LOC107784070	late blight resistance protein homolog R1A-3
LOC107769959	transcription factor TCP7	LOC107765792	7-deoxyloganetin glucosyltransferase
LOC107808755	uncharacterized	LOC107816302	fasciclin arabinogalactan protein 1
LOC107764228	uncharacterized	LOC107824272	adrenodoxin oxidoreductase
LOC107817191	cation/H(+) antiporter 18	LOC107821508	phosphatase IMPL1
LOC107805036	uncharacterized	LOC107801215	NDR1/HIN1-LIKE 10
LOC107806819	ubiquitin carboxyl-terminal hydrolase 12	LOC107824157	pentatricopeptide repeat-containing protein
LOC107826409	protein CASEIN KINASE I 3	LOC107780719	TrmH family tRNA/rRNA methyltransferase
LOC107812324	AP2 ethylene-responsive transcription factor TOE3	LOC107817722	vacuolar protein sorting-associated protein 36

LOC107832019	pectinesterase 4	LOC107767757	ethylene-responsive factor
LOC107794504	ribonuclease Z	LOC107811785	mitochondrial uncoupling protein 5
LOC107788801	Down syndrome critical region protein 3	LOC107824127	osmotin-like protein
LOC107817260	ER membrane protein complex subunit 1	LOC107780049	subtilisin protease SBT1.9
LOC107828178	ethylene-responsive transcription factor RAP2-12	LOC107793356	QUIRKY protein
LOC107773758	ATP-dependent DNA helicase 2 subunit KU80	LOC107823456	prefoldin subunit 2
LOC107830211	molybdenum cofactor sulfurase	LOC107815333	ethylene-responsive transcription factor ERF024
LOC107761968	receptor protein kinase 2	LOC107818239	RING-H2 finger protein ATL8
LOC107772232	subtilisin protease SBT1.7	LOC107773564	UDP-glucuronate 4-epimerase 1
LOC107769992	heat shock 70 kDa protein 8	LOC107784303	subtilisin protease SBT1.5
LOC107786935	chloroplast import apparatus 2	LOC107806470	SCARECROW protein
LOC107788682	WRKY transcription factor 57	LOC107826148	BAHD acyltransferase DCR
LOC107763462	uncharacterized	LOC107811831	kinase isozyme A, chloroplastic
LOC107828532	peptidyl-tRNA hydrolase ICT1	LOC107777350	protein HOTHEAD
LOC107815954	inorganic phosphate transporter 1-7	LOC107813598	pathogenesis-related protein 5

LOC107771821	ferredoxin-2	LOC107827507	3-isopropylmalate dehydratase 3
LOC107825976	BTB/POZ and TAZ domain-containing protein 3	LOC107785016	exocyst complex component
LOC107818518	uncharacterized	LOC107793322	CHUP1 protein
LOC107759882	uncharacterized	LOC107814873	fasciclin-like arabinogalactan protein 7
LOC107783922	uncharacterized		
LOC107812892	uncharacterized	LOC107806133	uncharacterized
LOC107815785	uncharacterized	LOC107762639	uncharacterized
LOC107778917	uncharacterized	LOC107785351	uncharacterized

Table S8 Differentially expressed gene descriptions of differential methylation on its promoter.

Stages	Methylation status	Gene ID	Description
11d	CG hypermethylation	LOC107776844	E3 ubiquitin-protein ligase RNF216
	CHH hypermethylation	**LOC107825473**	**Pentatricopeptide repeat (PPR-like) superfamily protein**
		LOC107763110	7-deoxyloganetic acid glucosyltransferase
		LOC107767493	B2 protein
		LOC107775219	40S ribosomal protein S24-1
		**LOC107779027**	**LRR receptor-like serine/threonine-protein kinase**
		LOC107789676	60S ribosomal protein L7a-2
		LOC107792081	repressor of silencing 1
		LOC107795928	DNA-binding protein SMUBP-2
		LOC107803698	transcriptional factor MYB1R1
		LOC107771222	uncharacterized

		LOC107805847	uncharacterized
		LOC107789908	uncharacterized
	CHH hypomethylation	LOC107765910	GIGANTEA
		**LOC107814288**	**outer envelope pore protein 16-3**
		**LOC107826423**	**mitochondrial import inner membrane translocase subunit TIM22-2**
		LOC107806884	calcium-dependent protein kinase 2
		LOC107764440	uncharacterized
		LOC107792593	uncharacterized
		LOC107804287	uncharacterized
13d	CHG hypermethylation	LOC107825777	senescence-associated gene 21
		LOC107807889	chlorophyll a-b binding protein 21
	CHH hypermethylation	LOC107818645	sodium-coupled neutral amino acid transporter 6
		LOC107813663	ras-related protein RABB1c
		LOC107761664	rho GTPase-activating protein 5
		LOC107770142	pectinesterase 2
		**LOC107775789**	**vesicle-associated membrane protein**
		LOC107795928	DNA-binding protein SMUBP-2
		LOC107805847	uncharacterized
	CHH hypomethylation	LOC107829374	trafficking protein particle complex subunit 3
		LOC107793273	guanine nucleotide-binding protein subunit beta-1
		**LOC107764440**	**mitochondrial import inner membrane translocase subunit tim22-2**
		**LOC107829500**	**proline-rich receptor-like protein kinase PERK3**
		LOC107765435	amidoxime reducing component 2
		LOC107773801	ethylene-responsive transcription factor 4
		LOC107812008	probable UMP-CMP kinase

		LOC107822770	serine/arginine repetitive matrix protein 1
		LOC107792799	sarcoplasmic reticulum histidine-rich calcium-binding protein
		LOC107773132	phenylalanine--tRNA ligase beta subunit
		LOC107759633	pectin lyase-like superfamily protein
		LOC107821897	ceramide inositolphosphotransferase
		LOC107828700	vicilin-like seed storage protein
		LOC107761556	plastid transcriptionally active protein
		LOC107790598	GTPase
		LOC107798621	low PSII accumulation 3
		LOC107816774	vacuolar amino acid transporter
		LOC107820542	60s ribosomal protein l34
		LOC107786144	60s ribosomal protein l6
		LOC107775886	acyl carrier protein 1
		LOC107821406	uncharacterized
		LOC107804287	uncharacterized
		LOC107759840	uncharacterized
		LOC107777748	uncharacterized
		LOC107788761	uncharacterized
13dr	CHG hypermethylation	LOC107825777	senescence-associated gene 21
	CHH hypermethylation	LOC107816774	vacuolar amino acid transporter 1
	CHH hypomethylation	LOC107824985	60s ribosomal protein l2
		LOC107828700	vicilin-like seed storage protein
		LOC107761838	ethylene-responsive transcription factor 9
		LOC107786144	60s ribosomal protein l6
		LOC107776470	uncharacterized

16d	CHH hypermethylation	LOC107829022	glucan endo-1,3-beta-glucosidase
		LOC107824084	glycine-rich protein a3
	CHH hypomethylation	LOC107824985	60s ribosomal protein l28
		LOC107773801	ethylene-responsive transcription factor 4
		LOC107779016	1-deoxy-d-xylulose 5-phosphate reductoisomerase
		LOC107813596	heat shock cognate protein 80
		LOC107765050	serine/arginine-rich splicing factor SR45
		LOC107795180	copper ion binding protein
		LOC107767998	hmg1/2
		**LOC107764440**	**mitochondrial import inner membrane translocase subunit tim22-2**
		LOC107765459	uncharacterized
		LOC107801583	uncharacterized
		LOC107767560	uncharacterized

Table S9 DMRs identified in this study. DMRs are separated by methylation direction (hyper-DMRs and hypo-DMRs) and developmental stages.

11days post inoculation (Hyper-DMRs)

Scaffold	Start	End	Region strain	Overlapped Site
NW_015787245.1	16608	16717	+	109
NW_015787310.1	64809	64846	+	37
NW_015787497.1	24967	25113	-	146
NW_015787551.1	30053	30120	+	67
NW_015787703.1	14837	15047	+	210
NW_015787703.1	14858	15048	+	190

NW_015787982.1	65184	65286	-	102
NW_015788485.1	16115	16232	+	117
NW_015788719.1	43423	43517	-	94
NW_015788949.1	4232	4616	-	384
NW_015789314.1	46877	46931	+	54
NW_015789314.1	46563	46934	+	256
NW_015789464.1	1983	2116	-	133
NW_015789781.1	23214	23280	-	66
NW_015789959.1	10942	11049	-	107
NW_015790036.1	14737	14844	-	107
NW_015790195.1	5714	5762	+	48
NW_015790534.1	8015	8146	+	131
NW_015790901.1	202854	203005	-	151
NW_015790989.1	41834	41870	+	36
NW_015791229.1	10824	10917	-	93
NW_015791242.1	69020	69189	-	169
NW_015791343.1	16379	16529	-	150
NW_015791379.1	5755	6064	+	309
NW_015791525.1	27800	27883	-	83
NW_015791958.1	9573	9594	+	21
NW_015792008.1	12016	12122	-	106
NW_015792008.1	11993	12126	-	133
NW_015792341.1	106386	106548	+	162
NW_015792667.1	61358	61511	+	153
NW_015792667.1	61225	61526	+	301

NW_015792667.1	60899	61530	+	631
NW_015792707.1	17301	17373	+	46
NW_015792777.1	15321	15428	+	107
NW_015792890.1	11476	11546	+	70
NW_015793009.1	4562	4629	+	67
NW_015793281.1	26966	27098	-	132
NW_015793415.1	1882	1936	-	54
NW_015793447.1	72044	72148	+	104
NW_015794027.1	17914	17970	-	56
NW_015794027.1	17904	17940	-	36
NW_015794361.1	13260	13399	+	139
NW_015794418.1	39598	39754	-	156
NW_015794790.1	9501	9729	-	228
NW_015795024.1	19933	19978	+	45
NW_015795215.1	23503	23554	+	51
NW_015795341.1	68164	68268	-	104
NW_015795462.1	4536	4691	+	155
NW_015795818.1	38050	38232	+	182
NW_015795904.1	20484	20531	+	47
NW_015795904.1	20468	20536	+	68
NW_015795913.1	6335	6462	+	127
NW_015795960.1	19623	19787	+	164
NW_015795960.1	19623	19787	-	164
NW_015796100.1	7524	7632	-	108
NW_015796427.1	1523	1700	+	177

NW_015796709.1	31348	31527	+	179
NW_015796781.1	14730	14844	+	114
NW_015796851.1	13816	13886	-	70
NW_015796928.1	69764	69870	+	106
NW_015796928.1	70299	70319	+	20
NW_015796973.1	31916	32018	+	102
NW_015797162.1	39755	39912	-	157
NW_015797899.1	59829	59878	+	49
NW_015797900.1	3728	3756	+	28
NW_015797976.1	33200	33345	-	95
NW_015798041.1	33142	33287	-	145
NW_015798463.1	49680	49788	+	108
NW_015798523.1	31747	31938	-	191
NW_015798967.1	19076	19201	-	125
NW_015799156.1	12525	12641	-	82
NW_015799159.1	5703	5908	+	205
NW_015799259.1	22333	22391	-	58
NW_015799362.1	84921	84933	-	12
NW_015799622.1	11592	11728	+	136
NW_015799707.1	13162	13386	-	224
NW_015800549.1	202656	202943	+	287
NW_015800635.1	17930	18051	-	121
NW_015800635.1	17848	18062	-	214
NW_015800721.1	6646	6783	+	137
NW_015801865.1	39071	39176	+	105

NW_015801865.1	39079	39197	+	118
NW_015801978.1	51217	51507	-	290
NW_015802091.1	22850	22960	-	110
NW_015802381.1	28161	28420	+	259
NW_015802432.1	29627	29713	+	86
NW_015802481.1	6719	6802	+	83
NW_015802646.1	105590	105681	+	91
NW_015802671.1	15708	15917	-	209
NW_015802684.1	18532	18604	-	72
NW_015802684.1	18521	18592	-	71
NW_015802852.1	6601	6776	+	175
NW_015803184.1	55129	55441	-	203
NW_015803187.1	20175	20385	+	210
NW_015803465.1	15453	15647	+	194
NW_015803465.1	42643	42684	+	41
NW_015803530.1	15758	15880	-	122
NW_015803664.1	65099	65276	+	177
NW_015803772.1	2410	2550	-	140
NW_015803796.1	18233	18299	-	66
NW_015803869.1	18709	18948	-	239
NW_015803881.1	14540	14639	+	99
NW_015804188.1	22615	22897	-	282
NW_015804188.1	22728	22848	-	120
NW_015804188.1	22569	22843	-	274
NW_015804242.1	5068	5165	-	97

NW_015804603.1	16529	16651	-	86
NW_015804894.1	110478	110522	+	44
NW_015804904.1	73240	73347	+	107
NW_015804904.1	73201	73380	+	179
NW_015804904.1	73191	73423	+	232
NW_015804964.1	12953	13076	-	28
NW_015804964.1	12950	13078	-	97
NW_015805054.1	26465	26593	-	128
NW_015805054.1	26408	26684	-	276
NW_015805116.1	39713	39750	-	37
NW_015805288.1	16533	16754	+	221
NW_015805369.1	9527	9591	-	64
NW_015805657.1	44867	45156	-	289
NW_015805756.1	30198	30297	-	99
NW_015805798.1	87106	87184	+	78
NW_015805962.1	34208	34267	-	59
NW_015806335.1	42577	42646	+	69
NW_015806572.1	93570	93696	-	126
NW_015806586.1	39579	39638	-	59
NW_015806798.1	29747	29783	-	36
NW_015806880.1	355943	356114	-	147
NW_015807042.1	36748	36836	-	88
NW_015807078.1	14763	14934	-	91
NW_015807453.1	13859	14063	-	204
NW_015807637.1	2940	3119	-	68

NW_015807748.1	10494	10602	+	108
NW_015807757.1	55622	55635	-	13
NW_015807931.1	27048	27149	-	101
NW_015807931.1	19677	19768	+	91
NW_015808541.1	128875	129053	+	178
NW_015808706.1	5184	5260	+	76
NW_015808779.1	11029	11168	-	139
NW_015809359.1	27762	27808	-	46
NW_015809359.1	27760	27825	+	65
NW_015809564.1	28786	28843	-	57
NW_015809582.1	32172	32220	-	48
NW_015809588.1	3872	3922	+	50
NW_015809729.1	7612	7705	-	93
NW_015810183.1	497	570	-	73
NW_015810190.1	11836	11977	-	141
NW_015810404.1	21623	21806	+	183
NW_015810455.1	3415	3738	-	323
NW_015810795.1	29368	29480	+	112
NW_015810838.1	21670	21844	+	174
NW_015810838.1	21579	21891	+	312
NW_015811216.1	68641	68801	+	160
NW_015811347.1	80681	80871	+	190
NW_015811347.1	80320	80353	+	33
NW_015811418.1	6173	6303	+	130
NW_015811434.1	60936	60978	+	42

NW_015811591.1	41974	42181	+	207
NW_015811603.1	27222	27262	+	40
NW_015811734.1	45862	45989	-	127
NW_015811867.1	70223	70285	+	62
NW_015812084.1	20825	20910	-	57
NW_015812084.1	20733	20938	-	149
NW_015812212.1	46943	47013	+	70
NW_015812595.1	248232	248291	-	59
NW_015812697.1	18387	18403	-	16
NW_015812702.1	57196	57338	+	142
NW_015812949.1	93330	93502	-	172
NW_015813061.1	96926	97039	-	113
NW_015813162.1	11773	11877	-	104
NW_015813218.1	14840	14987	-	147
NW_015813425.1	50995	51060	-	65
NW_015813552.1	36092	36109	-	17
NW_015814109.1	17473	17617	-	144
NW_015814172.1	29640	29753	-	113
NW_015814172.1	29687	29786	-	99
NW_015814451.1	24891	25105	-	214
NW_015815076.1	16192	16249	-	57
NW_015815179.1	42375	42527	+	136
NW_015815399.1	36000	36093	-	93
NW_015815789.1	20973	21109	+	136
NW_015816232.1	120589	120621	-	32

NW_015816487.1	17699	17966	-	267
NW_015816520.1	4924	5046	+	122
NW_015817356.1	53320	53430	-	110
NW_015817414.1	6067	6163	+	96
NW_015817414.1	6069	6303	+	125
NW_015817453.1	9851	9980	-	116
NW_015817715.1	18422	18497	-	75
NW_015818197.1	18808	18879	+	71
NW_015818386.1	139675	139980	-	305
NW_015818498.1	11663	11777	-	114
NW_015818771.1	45848	45955	+	107
NW_015819405.1	17387	17527	+	140
NW_015819541.1	74337	74554	+	217
NW_015820254.1	11205	11443	+	177
NW_015820280.1	61475	61680	-	205
NW_015820494.1	5341	5628	+	287
NW_015820661.1	3370	3549	-	179
NW_015821239.1	322790	322940	-	150
NW_015821239.1	322740	322979	-	239
NW_015821879.1	38782	38932	+	150
NW_015822001.1	13961	14053	-	79
NW_015822171.1	4823	4998	-	175
NW_015822259.1	30757	30931	+	126
NW_015822259.1	30729	30887	+	30
NW_015822502.1	18094	18172	+	78

NW_015822579.1	165064	165467	-	403
NW_015822881.1	53466	53572	+	106
NW_015822885.1	4435	4557	-	122
NW_015823240.1	21239	21283	-	44
NW_015823840.1	108170	108279	-	109
NW_015824399.1	111524	111641	+	117
NW_015824705.1	14060	14209	+	149
NW_015825133.1	93530	93643	-	113
NW_015825368.1	273984	274094	-	104
NW_015826182.1	17389	17598	-	132
NW_015826504.1	6472	6623	-	151
NW_015826616.1	4329	4461	-	126
NW_015826753.1	82874	83082	+	208
NW_015826753.1	82830	83094	+	264
NW_015827057.1	22771	22790	-	19
NW_015827057.1	18643	18759	-	116
NW_015827326.1	55029	55201	+	172
NW_015827326.1	55023	55209	+	186
NW_015827689.1	75197	75348	-	151
NW_015827913.1	30843	31103	+	260
NW_015828298.1	19039	19245	+	206
NW_015828298.1	49093	49246	-	153
NW_015829153.1	124920	125076	-	156
NW_015829153.1	124923	125032	-	109
NW_015829390.1	19504	19645	-	141

NW_015829472.1	13424	13483	+	59
NW_015829527.1	18403	18472	+	69
NW_015829606.1	35080	35189	-	109
NW_015829626.1	12111	12150	+	39
NW_015829643.1	18612	18694	+	82
NW_015829671.1	27010	27130	-	120
NW_015829824.1	8008	8167	+	159
NW_015829845.1	84160	84275	-	115
NW_015830203.1	27121	27158	-	37
NW_015830252.1	27601	27880	+	279
NW_015830272.1	58290	58374	-	84
NW_015830272.1	58234	58386	-	152
NW_015830304.1	19385	19503	+	118
NW_015830450.1	32331	32413	+	82
NW_015830513.1	22394	22542	-	148
NW_015831229.1	185784	185833	-	49
NW_015831436.1	107293	107361	+	68
NW_015831589.1	1161	1298	+	80
NW_015831880.1	13492	13661	-	169
NW_015831880.1	13523	13575	-	52
NW_015831880.1	13509	13659	-	150
NW_015831967.1	15224	15286	-	62
NW_015832227.1	26234	26380	+	146
NW_015832513.1	17399	17483	-	84
NW_015832645.1	38352	38497	-	145

NW_015833075.1	14586	14734	+	148
NW_015833255.1	21852	22034	+	182
NW_015833394.1	64406	64476	-	70
NW_015833432.1	62757	62843	-	52
NW_015833779.1	36253	36351	+	98
NW_015834002.1	28842	28901	-	59
NW_015834422.1	46446	46505	-	59
NW_015834644.1	30340	30495	+	155
NW_015834645.1	16559	16651	-	92
NW_015834645.1	16528	16673	-	145
NW_015834952.1	136503	136591	+	88
NW_015835004.1	35964	36102	+	138
NW_015835533.1	666	684	+	18
NW_015835620.1	184279	184409	-	94
NW_015835713.1	235	369	-	134
NW_015835721.1	18034	18231	+	197
NW_015835721.1	18015	18247	+	232
NW_015835784.1	10736	10842	+	106
NW_015836112.1	128242	128346	+	104
NW_015836299.1	58305	58377	-	72
NW_015836788.1	11150	11252	-	102
NW_015836876.1	2543	2657	-	114
NW_015836918.1	67289	67415	+	126
NW_015838185.1	12667	12847	+	103
NW_015838405.1	27445	27593	+	148

NW_015838877.1	59496	59517	-	21
NW_015838958.1	2443	2585	-	142
NW_015839040.1	21222	21324	+	102
NW_015839053.1	11039	11645	+	606
NW_015840985.1	18764	19062	-	209
NW_015842029.1	56794	56866	+	72
NW_015842755.1	252607	252692	+	85
NW_015843935.1	30412	30525	-	113
NW_015844058.1	8875	9043	-	168
NW_015844286.1	51062	51186	+	124
NW_015844914.1	149875	149976	-	101
NW_015844926.1	45771	45831	+	60
NW_015844978.1	8790	8905	-	115
NW_015845165.1	8794	8893	+	99
NW_015846222.1	77625	77866	+	241
NW_015846222.1	77610	77808	+	198
NW_015846331.1	52576	52613	+	37
NW_015846331.1	52547	52620	+	73
NW_015846425.1	253441	253563	+	122
NW_015846564.1	7468	7530	-	62
NW_015846569.1	23059	23148	+	89
NW_015846684.1	27554	27602	-	48
NW_015846874.1	15916	15988	+	72
NW_015846874.1	15907	16018	+	111
NW_015846929.1	69182	69291	-	109

NW_015847004.1	75780	75908	-	128
NW_015847085.1	55604	55671	+	47
NW_015847352.1	15585	15684	-	60
NW_015847612.1	19331	19471	-	127
NW_015848474.1	12642	12736	-	94
NW_015848474.1	13965	14209	-	244
NW_015848474.1	14006	14213	-	207
NW_015848601.1	52663	52818	+	155
NW_015848894.1	118974	119105	-	131
NW_015850014.1	3949	4166	-	217
NW_015850224.1	7876	7955	+	79
NW_015850267.1	37121	37169	+	48
NW_015850436.1	91404	91480	+	76
NW_015850669.1	2750	2896	-	146
NW_015851097.1	80409	80528	-	119
NW_015851162.1	23340	23383	+	43
NW_015851354.1	64711	64888	-	177
NW_015851596.1	39889	39948	+	59
NW_015851661.1	14029	14152	-	123
NW_015852267.1	32	266	+	21
NW_015852854.1	59939	60075	-	136
NW_015852854.1	59944	60109	-	165
NW_015853108.1	26348	26394	-	46
NW_015853183.1	44063	44110	+	47
NW_015853374.1	19436	19548	-	112

NW_015853692.1	28837	28948	-	111
NW_015854875.1	13130	13268	-	138
NW_015855180.1	46425	46522	+	97
NW_015855180.1	46428	46593	+	165
NW_015855245.1	12137	12291	-	81
NW_015856211.1	45208	45319	-	111
NW_015856392.1	14164	14291	-	127
NW_015856503.1	36684	36872	-	188
NW_015856685.1	127681	127757	+	76
NW_015857278.1	27935	28149	+	214
NW_015857372.1	5627	5758	-	99
NW_015857449.1	6505	6641	+	136
NW_015857847.1	3329	3441	-	112
NW_015858019.1	41170	41302	-	132
NW_015858073.1	3848	4139	+	291
NW_015858440.1	5916	6100	+	165
NW_015858521.1	129034	129146	-	112
NW_015858606.1	29112	29269	-	157
NW_015858624.1	55157	55183	-	26
NW_015858696.1	77168	77252	-	84
NW_015858696.1	77131	77253	-	122
NW_015858696.1	77134	77249	-	115
NW_015858814.1	133383	133443	+	60
NW_015858887.1	60971	61023	+	52
NW_015859072.1	86137	86314	+	177

NW_015859358.1	44207	44289	-	82
NW_015859577.1	15527	15608	-	81
NW_015860585.1	83229	83327	+	98
NW_015860891.1	42847	43059	+	204
NW_015860976.1	1937	2170	+	233
NW_015860976.1	1940	2202	+	262
NW_015861076.1	15088	15133	-	45
NW_015861581.1	23259	23367	+	108
NW_015861812.1	7193	7336	+	143
NW_015862097.1	36725	36815	-	90
NW_015862329.1	42020	42170	-	150
NW_015862711.1	7257	7292	-	35
NW_015862734.1	101443	101568	-	125
NW_015863090.1	9243	9350	-	107
NW_015863169.1	1884	2241	+	357
NW_015863210.1	220474	220563	-	89
NW_015863210.1	220464	220564	+	100
NW_015863796.1	1694	1721	+	27
NW_015864327.1	55885	55943	-	58
NW_015864800.1	59120	59445	+	325
NW_015864951.1	12706	12823	-	69
NW_015865074.1	48074	48254	-	180
NW_015865814.1	568	727	-	84
NW_015866076.1	1828	1922	+	94
NW_015866417.1	6783	7050	+	267

NW_015866444.1	3629	3711	+	82
NW_015866547.1	5394	5576	+	182
NW_015866547.1	5393	5616	-	223
NW_015866820.1	47354	47436	-	82
NW_015866820.1	47297	47444	-	147
NW_015867027.1	55823	55891	-	68
NW_015867185.1	54310	54339	+	29
NW_015867266.1	55969	56136	-	167
NW_015867331.1	8349	8382	+	33
NW_015867717.1	124805	124978	-	173
NW_015867809.1	123555	123605	-	50
NW_015867993.1	6334	6454	+	120
NW_015868019.1	117695	117896	-	201
NW_015868037.1	8080	8249	-	169
NW_015868037.1	8114	8234	-	120
NW_015868037.1	8064	8185	-	121
NW_015868079.1	26674	26840	+	154
NW_015868079.1	26655	26830	+	173
NW_015868316.1	118586	118780	+	161
NW_015868410.1	66576	66667	-	91
NW_015868935.1	21557	21719	+	162
NW_015869458.1	27777	27914	+	128
NW_015869480.1	28482	28554	+	72
NW_015869981.1	74241	74350	-	109
NW_015870347.1	73445	73583	-	138

NW_015870347.1	77836	77941	-	105
NW_015870385.1	8929	9076	+	147
NW_015870571.1	5707	5854	+	121
NW_015870962.1	35917	35959	+	42
NW_015872001.1	31631	31686	-	55
NW_015872355.1	11684	11868	+	124
NW_015872592.1	5076	5283	-	207
NW_015873125.1	170949	171185	-	236
NW_015873620.1	64931	65003	+	72
NW_015874034.1	55303	55360	+	57
NW_015874034.1	25088	25373	-	285
NW_015874108.1	76058	76150	-	92
NW_015874108.1	76019	76195	-	176
NW_015874690.1	28379	28532	+	153
NW_015875939.1	29310	29353	+	43
NW_015875985.1	5442	5598	-	106
NW_015876040.1	4666	4765	-	99
NW_015876407.1	70373	70504	+	131
NW_015876584.1	38014	38105	-	91
NW_015876682.1	2450	2581	-	131
NW_015876686.1	16406	16454	+	48
NW_015876831.1	14930	15064	-	134
NW_015877047.1	7543	7667	-	72
NW_015877851.1	19223	19544	-	321
NW_015878004.1	13327	13414	-	87

NW_015878092.1	47364	47513	-	149
NW_015878545.1	26643	26793	+	150
NW_015878553.1	6494	6614	-	120
NW_015878796.1	20353	20508	+	155
NW_015879156.1	7926	7952	+	26
NW_015879194.1	12891	12975	+	84
NW_015879228.1	36082	36150	-	68
NW_015879256.1	20696	20755	-	59
NW_015879333.1	5066	5191	-	125
NW_015879477.1	74627	74773	-	146
NW_015879733.1	13968	14052	+	84
NW_015880231.1	12780	12880	+	100
NW_015880571.1	64314	64580	-	266
NW_015880571.1	64332	64581	-	249
NW_015880571.1	64319	64605	-	286
NW_015880889.1	16026	16185	-	15
NW_015881203.1	58128	58256	+	128
NW_015881232.1	6479	6622	+	143
NW_015881359.1	24830	24851	-	21
NW_015881441.1	33150	33270	-	120
NW_015881861.1	166840	166996	-	156
NW_015882230.1	57331	57518	-	187
NW_015882277.1	37223	37320	-	97
NW_015882708.1	55117	55320	-	203
NW_015882708.1	55683	55761	-	78

NW_015882745.1	30693	30793	+	100
NW_015882749.1	2376	2532	-	156
NW_015882986.1	20725	20851	-	126
NW_015883151.1	64312	64345	+	33
NW_015884381.1	89707	89792	-	85
NW_015884425.1	3898	3961	+	63
NW_015885149.1	55036	55062	-	26
NW_015885152.1	126923	127295	-	372
NW_015885448.1	41540	41757	+	217
NW_015885516.1	21538	21595	-	57
NW_015885611.1	33227	33305	-	78
NW_015885857.1	73774	73964	-	190
NW_015885985.1	31985	32484	-	499
NW_015886190.1	33092	33242	-	150
NW_015886239.1	103137	103336	+	199
NW_015886315.1	45746	45789	+	43
NW_015886439.1	72277	72359	+	82
NW_015886440.1	53781	53860	-	79
NW_015886497.1	33223	33345	-	122
NW_015886688.1	50425	50565	+	140
NW_015886706.1	9283	9298	+	15
NW_015886706.1	67936	68103	+	167
NW_015886829.1	39014	39164	+	150
NW_015886920.1	25974	26094	-	120
NW_015886920.1	61077	61167	-	90

NW_015887012.1	52173	52311	-	138
NW_015887046.1	20682	20765	+	83
NW_015887046.1	20616	20785	+	169
NW_015887048.1	20257	20441	+	184
NW_015887124.1	12532	12628	-	96
NW_015887157.1	86656	86695	+	39
NW_015887157.1	88601	88664	+	63
NW_015887273.1	5397	5415	+	18
NW_015887336.1	59197	59341	+	144
NW_015887373.1	26517	26631	-	114
NW_015887416.1	10504	10573	-	69
NW_015887443.1	65469	65527	+	58
NW_015887446.1	19920	19986	+	66
NW_015887460.1	16302	16455	+	153
NW_015887579.1	77833	78003	-	170
NW_015887596.1	9263	9287	-	24
NW_015887642.1	46719	46860	+	141
NW_015887726.1	182019	182154	-	135
NW_015887837.1	101372	101565	-	193
NW_015887841.1	82004	82069	+	65
NW_015887983.1	81663	81732	-	69
NW_015888181.1	60178	60248	-	70
NW_015888274.1	105877	106087	-	210
NW_015888286.1	9424	9663	+	239
NW_015888421.1	7870	7984	-	85

NW_015888421.1	7857	8002	-	98
NW_015888618.1	65049	65193	-	144
NW_015888685.1	9466	9482	+	16
NW_015888830.1	6057	6192	-	135
NW_015888964.1	8859	9009	+	150
NW_015888964.1	25968	26011	+	43
NW_015889033.1	9621	9754	+	133
NW_015889092.1	23672	23881	+	209
NW_015889103.1	82386	82506	-	120
NW_015889172.1	37429	37526	-	97
NW_015889258.1	68046	68196	-	88
NW_015889271.1	59951	60203	+	252
NW_015889466.1	9059	9123	+	64
NW_015889470.1	21716	21800	-	84
NW_015889474.1	158099	158184	+	85
NW_015889612.1	6107	6223	+	116
NW_015890095.1	72759	72813	+	54
NW_015890123.1	78674	78751	+	77
NW_015890165.1	15663	15827	+	164
NW_015890230.1	34028	34178	-	86
NW_015890340.1	19586	19660	-	74
NW_015890342.1	153170	153242	+	72
NW_015890528.1	18801	18931	+	130
NW_015890569.1	6672	6736	+	64
NW_015890856.1	46144	46203	+	59

NW_015890994.1	116118	116161	-	43
NW_015891022.1	11391	11561	-	170
NW_015891049.1	77660	77756	+	96
NW_015891054.1	33531	33628	-	97
NW_015891120.1	56555	56715	+	160
NW_015891143.1	21365	21433	-	68
NW_015891252.1	67977	68093	-	116
NW_015891252.1	67993	68098	+	105
NW_015891346.1	22721	22773	+	52
NW_015891417.1	60049	60190	+	141
NW_015891443.1	195512	195723	+	161
NW_015891443.1	195498	196084	+	175
NW_015891485.1	8200	8333	-	133
NW_015891604.1	17531	17645	-	114
NW_015891604.1	17527	17655	-	128
NW_015891660.1	64430	64624	+	194
NW_015891660.1	64426	64629	+	203
NW_015891723.1	165324	165475	+	151
NW_015891764.1	81047	81142	+	95
NW_015891785.1	57122	57397	+	275
NW_015891825.1	19260	19394	+	134
NW_015892023.1	67813	68206	-	393
NW_015892253.1	91168	91301	+	133
NW_015892254.1	26979	27108	-	129
NW_015892342.1	160564	160588	-	24

NW_015893945.1	12224	12349	+	125
NW_015894675.1	22954	23048	+	94
NW_015894990.1	117291	117316	+	25
NW_015895023.1	142	425	+	237
NW_015897167.1	21415	21512	+	97
NW_015897181.1	612	837	+	225
NW_015897309.1	147331	147428	+	97
NW_015897439.1	28141	28342	+	142
NW_015897470.1	81	368	-	287
NW_015897702.1	2061	2082	+	21
NW_015897767.1	2190	2220	+	30
NW_015898069.1	450	609	-	159
NW_015898099.1	1874	2151	+	143
NW_015898590.1	1114	1197	+	7
NW_015898590.1	1098	1238	+	48
NW_015898598.1	53639	53809	+	170
NW_015898685.1	2041	2095	-	54
NW_015898908.1	6209	6316	-	107
NW_015898954.1	2741	2911	+	170
NW_015898954.1	2717	2913	+	196
NW_015899018.1	2773	2898	-	125
NW_015899071.1	8900	9013	+	113
NW_015899134.1	84245	84357	-	112
NW_015899304.1	59665	59720	-	55
NW_015899361.1	57873	58065	+	192

NW_015899361.1	57848	58042	+	194
NW_015899424.1	15144	15240	+	96
NW_015899445.1	106169	106230	+	61
NW_015899445.1	161329	161368	-	39
NW_015899512.1	8248	8420	+	172
NW_015899655.1	212564	212584	+	20
NW_015899706.1	116716	116727	+	11
NW_015899706.1	116700	116734	+	34
NW_015899712.1	154276	154429	+	153
NW_015899839.1	46182	46379	-	197
NW_015899919.1	26421	26651	+	229
NW_015899983.1	197299	197377	+	78
NW_015899988.1	8698	8749	+	51
NW_015900033.1	26633	26730	+	97
NW_015900085.1	53853	53956	+	103
NW_015900085.1	53828	53961	+	133
NW_015900220.1	114109	114160	-	51
NW_015900220.1	114092	114215	-	123
NW_015900222.1	671	712	+	41
NW_015900233.1	25078	25122	+	44
NW_015900244.1	4213	4234	+	21
NW_015900341.1	62164	62372	-	153
NW_015900396.1	42341	42494	+	153
NW_015900401.1	40489	40570	+	81
NW_015900935.1	32212	32356	-	144

NW_015900939.1	24184	24508	+	324
NW_015900939.1	24189	24552	+	363
NW_015900952.1	115727	115831	+	78
NW_015900991.1	4119	4250	+	131
NW_015901067.1	8032	8196	-	164
NW_015901126.1	43114	43140	-	26
NW_015901493.1	12459	12565	+	106
NW_015901529.1	24884	24903	+	19
NW_015901529.1	31305	31416	+	111
NW_015901532.1	39882	40004	-	122
NW_015901537.1	22829	22978	+	149
NW_015901537.1	22809	22983	+	174
NW_015901597.1	16800	16877	+	77
NW_015901622.1	22503	22641	+	138
NW_015901634.1	13539	13643	+	104
NW_015901645.1	47458	47575	+	117
NW_015901645.1	47426	47582	+	156
NW_015901681.1	126107	126174	+	67
NW_015901688.1	78543	78643	-	100
NW_015901719.1	12514	12575	-	61
NW_015902096.1	22830	22933	+	103
NW_015902239.1	39040	39158	-	118
NW_015902319.1	66064	66196	-	132
NW_015902519.1	71476	71537	-	61
NW_015902749.1	87353	87999	+	646

NW_015902823.1	18816	18880	-	64
NW_015902891.1	26081	26208	+	127
NW_015903036.1	8224	8334	+	110
NW_015903041.1	59436	59559	+	123
NW_015903179.1	50201	50219	+	18
NW_015903197.1	19665	19738	-	73
NW_015903294.1	8723	8804	+	81
NW_015903429.1	96436	96475	-	39
NW_015903429.1	27780	27933	+	73
NW_015903429.1	27783	27929	+	73
NW_015903434.1	44222	44588	+	115
NW_015903469.1	37914	38048	-	134
NW_015903630.1	28741	28844	-	103
NW_015903723.1	36749	36906	-	157
NW_015903728.1	15208	15320	+	112
NW_015903847.1	4803	4933	-	130
NW_015904002.1	45411	45538	+	127
NW_015904263.1	30948	30986	-	38
NW_015904276.1	9948	10053	+	105
NW_015904304.1	52274	52385	+	60
NW_015904325.1	2307	2406	+	99
NW_015904370.1	149560	149683	-	123
NW_015904370.1	149574	149735	-	161
NW_015904532.1	74019	74174	-	155
NW_015904609.1	54961	55082	-	121

NW_015904724.1	47748	47888	-	140
NW_015904798.1	62375	62540	-	165
NW_015904854.1	24880	24952	+	72
NW_015904904.1	34183	34542	-	206
NW_015905003.1	9235	9323	+	21
NW_015905276.1	74716	74811	+	51
NW_015905422.1	18427	18567	+	134
NW_015905615.1	10801	10962	+	161
NW_015905635.1	65762	65891	+	129
NW_015905639.1	39589	39726	+	137
NW_015905711.1	8415	8451	+	36
NW_015905725.1	114162	114220	-	58
NW_015905862.1	17485	17626	+	141
NW_015905901.1	30356	30391	-	35
NW_015905982.1	24035	24131	+	96
NW_015905984.1	51624	51686	+	62
NW_015906226.1	19835	20035	-	200
NW_015906584.1	65241	65512	+	142
NW_015906752.1	126851	126975	+	124
NW_015907225.1	21938	22048	+	110
NW_015907307.1	29140	29399	+	259
NW_015907376.1	99900	100075	-	38
NW_015907449.1	18765	18834	+	69
NW_015907489.1	26829	26920	+	91
NW_015907531.1	1662	1839	-	177

NW_015907602.1	57885	58061	+	176
NW_015907646.1	51693	51849	-	156
NW_015907696.1	22892	22986	-	94
NW_015907696.1	22886	22994	-	108
NW_015907710.1	24928	25044	+	116
NW_015907783.1	9553	9643	-	90
NW_015907866.1	72163	72285	-	98
NW_015908015.1	25418	25580	-	162
NW_015908101.1	42795	42817	-	22
NW_015908107.1	19897	20045	-	148
NW_015908150.1	120379	120500	+	121
NW_015908158.1	119932	120349	-	417
NW_015908174.1	30271	30424	+	153
NW_015908230.1	223350	223527	-	177
NW_015908325.1	21831	21982	-	151
NW_015908394.1	41092	41152	-	60
NW_015908598.1	45473	45539	+	66
NW_015908598.1	45470	45544	+	74
NW_015908769.1	129524	129664	+	140
NW_015908848.1	135666	135698	+	32
NW_015909017.1	71148	71281	-	133
NW_015909169.1	108845	109098	+	253
NW_015909524.1	19280	19352	-	72
NW_015909562.1	21380	21431	+	51
NW_015909562.1	22144	22279	+	94

NW_015909723.1	35660	35700	+	40
NW_015909772.1	17304	17378	+	74
NW_015909944.1	211011	211091	-	54
NW_015910288.1	43063	43168	-	105
NW_015910414.1	54101	54175	-	74
NW_015910447.1	74072	74186	-	114
NW_015910720.1	3389	3561	-	172
NW_015910734.1	80928	80973	+	45
NW_015910735.1	80592	80658	+	66
NW_015910859.1	3457	3534	-	77
NW_015910912.1	24619	24730	+	3
NW_015911170.1	53752	53828	-	76
NW_015911274.1	10954	11025	-	71
NW_015911352.1	32124	32285	+	161
NW_015911352.1	32475	32594	+	119
NW_015911563.1	2789	2815	-	26
NW_015911623.1	26986	27172	-	129
NW_015911809.1	24384	24440	+	56
NW_015912020.1	35261	35377	-	116
NW_015912040.1	7546	7648	+	102
NW_015912131.1	28154	28537	+	383
NW_015912281.1	12407	12576	-	169
NW_015912369.1	95692	95714	-	22
NW_015912481.1	39942	40069	+	127
NW_015912648.1	69946	70077	+	131

NW_015912724.1	160188	160368	+	180
NW_015912724.1	139877	139932	+	55
NW_015912853.1	8787	8932	+	145
NW_015912872.1	27440	27650	+	210
NW_015912912.1	60121	60394	-	273
NW_015912952.1	25535	25878	-	343
NW_015913019.1	155407	155414	-	7
NW_015913183.1	6524	6592	-	68
NW_015913329.1	21054	21109	+	55
NW_015913361.1	46572	46644	-	72
NW_015913361.1	46525	46645	-	120
NW_015913361.1	46515	46631	-	116
NW_015913398.1	16886	17014	+	128
NW_015913532.1	51704	51793	-	89
NW_015913538.1	22833	23034	-	201
NW_015913571.1	53427	53587	-	160
NW_015913678.1	6970	7169	+	105
NW_015913724.1	17873	17891	+	18
NW_015913743.1	5299	5414	+	115
NW_015913743.1	145362	145466	+	104
NW_015913743.1	5297	5395	+	98
NW_015913747.1	29820	29877	+	57
NW_015913813.1	11962	11996	-	34
NW_015913846.1	167696	167857	-	161
NW_015913846.1	183427	183465	+	38

NW_015913846.1	167692	167901	-	209
NW_015914026.1	15235	15443	+	208
NW_015914252.1	47173	47261	+	88
NW_015914252.1	47190	47260	+	70
NW_015914301.1	18754	18884	-	130
NW_015914492.1	34800	35058	+	258
NW_015914498.1	18019	18054	-	35
NW_015914631.1	27097	27415	-	318
NW_015914650.1	44059	44134	-	75
NW_015914806.1	25815	25920	+	105
NW_015914817.1	68023	68158	+	135
NW_015914889.1	19177	19348	-	171
NW_015914929.1	52282	52403	-	121
NW_015914942.1	36958	37083	-	125
NW_015915055.1	49591	49713	+	122
NW_015915328.1	50312	50349	-	37
NW_015915346.1	8540	8581	+	41
NW_015915475.1	84378	84507	+	129
NW_015915562.1	21228	21442	-	214
NW_015915563.1	22849	22959	-	110
NW_015915563.1	22856	23036	-	173
NW_015915700.1	20667	20734	+	67
NW_015916148.1	6692	6838	+	146
NW_015916354.1	19675	19820	-	145
NW_015916450.1	77426	77483	+	57

NW_015916456.1	26781	26947	+	166
NW_015916525.1	67901	67970	-	69
NW_015916540.1	3920	4171	-	88
NW_015916711.1	26595	26745	+	150
NW_015917019.1	8428	8639	+	211
NW_015917091.1	29290	29485	-	191
NW_015917182.1	35183	35408	+	211
NW_015917182.1	35174	35413	+	220
NW_015917424.1	36974	37035	+	61
NW_015917547.1	30639	30786	+	127
NW_015917758.1	77657	78167	+	510
NW_015917758.1	77469	78159	+	593
NW_015917816.1	24147	24257	-	110
NW_015917860.1	54566	54919	+	219
NW_015917911.1	9200	9377	+	177
NW_015917947.1	20967	21062	+	95
NW_015917947.1	20877	21088	+	211
NW_015918060.1	37382	37875	+	493
NW_015918104.1	19879	19892	-	13
NW_015918187.1	2909	3152	-	243
NW_015918187.1	2862	3155	-	293
NW_015918215.1	8398	8522	+	124
NW_015918253.1	198580	198641	-	61
NW_015918253.1	89291	89378	-	87
NW_015918449.1	110531	110691	-	111

NW_015918493.1	20319	20364	-	45
NW_015918578.1	37359	37397	-	38
NW_015918578.1	19674	19710	+	36
NW_015918582.1	10953	11025	+	72
NW_015918709.1	28505	28624	+	119
NW_015918760.1	32443	32533	-	90
NW_015918970.1	41123	41213	+	90
NW_015919010.1	100947	100981	+	34
NW_015919134.1	56825	57001	+	176
NW_015919197.1	1328	1478	+	150
NW_015919395.1	39141	39188	-	47
NW_015919512.1	18944	19131	-	187
NW_015919519.1	68681	68730	+	49
NW_015919596.1	18513	18617	-	104
NW_015919754.1	49796	49908	+	108
NW_015919781.1	359691	359738	+	47
NW_015919816.1	34599	34739	-	102
NW_015919816.1	34487	34733	-	83
NW_015919982.1	32318	32441	-	123
NW_015919999.1	101512	101742	+	230
NW_015919999.1	23357	23425	+	68
NW_015920008.1	133614	133728	-	114
NW_015920044.1	116230	116361	-	131
NW_015920044.1	116251	116382	-	131
NW_015920096.1	75669	75819	+	150

NW_015920320.1	11929	12058	-	129
NW_015920421.1	17376	17491	-	115
NW_015920512.1	76215	76332	+	117
NW_015920537.1	16074	16249	+	175
NW_015920670.1	150981	151072	-	91
NW_015920789.1	47638	47713	-	75
NW_015920834.1	6868	7030	+	28
NW_015920994.1	47332	47413	+	81
NW_015921169.1	93657	93809	-	152
NW_015921184.1	35238	35327	+	68
NW_015921216.1	37640	37694	+	54
NW_015921262.1	16395	16626	-	231
NW_015921348.1	3725	3826	-	101
NW_015921424.1	43424	43739	-	315
NW_015921424.1	43425	43504	-	79
NW_015921424.1	43415	43775	-	360
NW_015921452.1	99256	99328	+	45
NW_015921452.1	4302	4539	+	237
NW_015921455.1	44743	44962	+	127
NW_015921630.1	35048	35152	+	104
NW_015921695.1	20844	21011	-	167
NW_015921695.1	20831	21014	-	183
NW_015921717.1	5324	5514	-	190
NW_015921824.1	50654	50783	+	129
NW_015921825.1	5156	5251	+	95

NW_015921854.1	22482	22532	+	50
NW_015921961.1	5731	5826	-	95
NW_015922020.1	6472	6510	-	38
NW_015922140.1	77777	77897	-	120
NW_015922165.1	8086	8154	-	68
NW_015922213.1	14251	14360	-	109
NW_015922213.1	14267	14350	-	83
NW_015922412.1	34156	34187	+	31
NW_015922412.1	34147	34185	+	38
NW_015922412.1	34109	34182	+	73
NW_015922501.1	115914	115928	-	14
NW_015922526.1	39633	39939	-	306
NW_015922665.1	44009	44086	+	77
NW_015922693.1	116699	116816	-	117
NW_015922775.1	110826	110940	-	96
NW_015922866.1	173958	174120	+	162
NW_015923010.1	25287	25435	-	148
NW_015923106.1	44558	44627	-	69
NW_015923147.1	14130	14323	+	125
NW_015923147.1	14140	14320	+	122
NW_015923197.1	48414	48510	-	96
NW_015923395.1	72662	72750	+	88
NW_015923440.1	26029	26084	+	55
NW_015923544.1	8215	8358	-	143
NW_015923556.1	7853	7951	-	98

NW_015923839.1	54993	55117	-	104
NW_015923872.1	8114	8252	+	138
NW_015923882.1	22339	22467	-	128
NW_015924032.1	26869	27229	-	124
NW_015924362.1	57403	57492	+	89
NW_015924362.1	67836	67959	-	123
NW_015924568.1	6178	6328	-	150
NW_015924568.1	6180	6341	-	161
NW_015924585.1	48056	48108	+	52
NW_015924628.1	15933	15975	-	42
NW_015924670.1	80919	80987	-	68
NW_015924717.1	61796	61935	+	139
NW_015924729.1	978	1158	+	41
NW_015924783.1	18780	18930	-	150
NW_015924903.1	22734	22835	+	101
NW_015924903.1	22694	22981	+	287
NW_015924919.1	32744	32821	+	77
NW_015924919.1	27725	27893	+	168
NW_015924954.1	54958	54994	+	36
NW_015925128.1	5199	5420	-	221
NW_015925338.1	20512	20644	-	93
NW_015925357.1	31982	32196	+	214
NW_015925441.1	63402	63592	+	190
NW_015925614.1	119317	119476	+	159
NW_015925768.1	59189	59224	+	35

NW_015925772.1	114245	114342	+	97
NW_015925999.1	47675	47767	+	92
NW_015926003.1	106484	106599	+	115
NW_015926126.1	51582	51679	-	97
NW_015926130.1	104333	104413	-	80
NW_015926309.1	36754	36872	+	118
NW_015926466.1	47937	48011	+	74
NW_015926466.1	47940	48010	+	70
NW_015926470.1	41803	41953	-	124
NW_015926495.1	320823	320999	+	176
NW_015926586.1	94117	94275	-	158
NW_015926635.1	62463	62653	+	190
NW_015926652.1	19322	19533	-	211
NW_015926815.1	51349	51458	-	109
NW_015926839.1	223303	223356	-	53
NW_015926912.1	17601	17750	-	149
NW_015926912.1	17634	17740	-	106
NW_015926912.1	17589	17752	-	163
NW_015927016.1	19538	20244	+	706
NW_015927030.1	4102	4171	+	69
NW_015927089.1	9367	9650	+	41
NW_015927132.1	33927	34002	+	75
NW_015927132.1	541	608	+	54
NW_015927252.1	2341	2476	+	135
NW_015927253.1	23125	23304	+	179

NW_015927253.1	23132	23307	+	175
NW_015927625.1	26964	27050	-	86
NW_015927711.1	24009	24138	+	129
NW_015927711.1	23997	24137	+	140
NW_015927837.1	2569	2701	+	132
NW_015927959.1	11519	11655	-	136
NW_015928023.1	10957	11145	-	178
NW_015928128.1	56387	56470	+	83
NW_015928145.1	80901	81005	+	104
NW_015928276.1	34034	34128	+	94
NW_015928334.1	59890	60108	+	218
NW_015928622.1	35553	35664	+	111
NW_015928941.1	34615	34711	+	49
NW_015929212.1	61931	62203	+	272
NW_015929377.1	248459	248500	+	41
NW_015929427.1	27664	27725	-	61
NW_015929463.1	12793	12966	-	96
NW_015929491.1	3646	3910	+	264
NW_015929893.1	29970	30076	-	106
NW_015929893.1	29965	30107	-	142
NW_015929893.1	29988	30106	-	118
NW_015929896.1	45908	46333	-	425
NW_015929896.1	132625	132829	+	204
NW_015929922.1	8703	8832	+	129
NW_015929942.1	62725	62828	+	103

NW_015929962.1	37152	37193	-	41
NW_015930007.1	18461	18659	-	198
NW_015930032.1	16194	16263	-	69
NW_015930395.1	5990	6105	+	115
NW_015930498.1	29527	29740	+	171
NW_015930593.1	219515	219672	-	157
NW_015930637.1	9194	9416	+	183
NW_015930789.1	54613	54698	+	85
NW_015930821.1	2448	2741	-	293
NW_015930865.1	24424	24555	-	131
NW_015930915.1	20505	20589	+	84
NW_015930988.1	8788	8909	-	121
NW_015930990.1	126144	126285	-	141
NW_015930990.1	126072	126282	-	210
NW_015931056.1	906	1040	+	27
NW_015931076.1	57341	57437	+	96
NW_015931221.1	36123	36214	+	91
NW_015931255.1	57893	58020	-	127
NW_015931397.1	4857	4990	-	110
NW_015931444.1	22676	22837	-	161
NW_015931444.1	22671	22823	-	152
NW_015931477.1	15101	15199	-	50
NW_015931550.1	16447	16486	+	39
NW_015931566.1	1697	1731	+	34
NW_015931566.1	1679	1723	+	44

NW_015931628.1	56473	56555	+	82
NW_015931882.1	12038	12114	+	76
NW_015932222.1	13829	13975	-	146
NW_015932417.1	58225	58396	+	171
NW_015932470.1	45442	45547	-	105
NW_015932470.1	94411	94540	-	129
NW_015932485.1	9979	10155	-	89
NW_015932768.1	76730	76852	-	122
NW_015932994.1	119827	119905	+	78
NW_015933009.1	10654	10706	+	52
NW_015933212.1	2927	3050	-	123
NW_015933265.1	9610	9729	+	119
NW_015933330.1	46400	46504	+	104
NW_015933673.1	48160	48304	+	144
NW_015933684.1	23944	24179	-	235
NW_015934060.1	12333	12353	+	20
NW_015934100.1	104199	104339	-	140
NW_015934123.1	74833	74959	-	126
NW_015934124.1	25779	25994	+	215
NW_015934124.1	104943	105120	-	177
NW_015934363.1	70912	70959	+	47
NW_015934383.1	49504	49554	-	50
NW_015934455.1	9811	9872	+	61
NW_015934455.1	9821	9862	+	41
NW_015934455.1	9763	9877	+	114

NW_015934474.1	1951	1968	-	17
NW_015934476.1	54660	54797	-	137
NW_015934479.1	16027	16046	+	19
NW_015934627.1	3862	3965	+	103
NW_015934647.1	10121	10229	+	23
NW_015934647.1	10103	10216	+	87
NW_015934685.1	52318	52490	-	172
NW_015934728.1	96887	96970	-	78
NW_015935019.1	4298	4427	+	124
NW_015935086.1	31122	31232	+	110
NW_015935092.1	11256	11286	+	30
NW_015935426.1	3824	3985	-	93
NW_015935494.1	6580	6681	+	101
NW_015935610.1	57795	57921	-	126
NW_015935769.1	33362	33443	-	81
NW_015935841.1	69338	69492	-	102
NW_015936229.1	33611	33776	-	165
NW_015936296.1	10078	10224	+	84
NW_015936321.1	90724	90826	+	65
NW_015936352.1	24399	24493	-	94
NW_015936381.1	29283	29411	+	128
NW_015936671.1	123229	123382	+	153
NW_015936701.1	18998	19086	+	88
NW_015936714.1	37048	37134	-	86
NW_015936817.1	13792	13908	-	116

NW_015936845.1	11194	11357	+	82
NW_015937016.1	16959	17093	-	134
NW_015937016.1	16963	17041	-	78
NW_015937016.1	16954	17090	-	136
NW_015937152.1	16804	16900	-	91
NW_015937176.1	95011	95117	+	106
NW_015937251.1	144756	144857	-	101
NW_015937440.1	19828	19939	-	111
NW_015937848.1	48303	48347	-	44
NW_015938099.1	2805	3019	-	214
NW_015938284.1	27843	27912	+	69
NW_015938485.1	41449	41510	-	61
NW_015938485.1	41424	41588	+	164
NW_015938512.1	33709	33820	+	111
NW_015938515.1	12192	12307	-	115
NW_015938574.1	14499	14607	+	108
NW_015938964.1	11349	11464	-	115
NW_015939317.1	6120	6154	-	34
NW_015939512.1	10718	10765	-	47
NW_015939606.1	115318	115478	-	160
NW_015939970.1	92113	92177	+	64
NW_015940092.1	9943	10003	+	60
NW_015940157.1	19532	19725	+	193
NW_015940157.1	19550	19698	+	148
NW_015940333.1	155334	155431	-	97

NW_015940347.1	19523	19684	+	134
NW_015940574.1	30087	30474	+	387
NW_015940661.1	7329	7379	+	50
NW_015940734.1	5544	5674	-	130
NW_015940883.1	26327	26388	+	61
NW_015940942.1	95617	95683	+	66
NW_015941027.1	2423	2540	-	117
NW_015941041.1	2108	2219	+	111
NW_015941067.1	19932	20002	-	70
NW_015941143.1	23735	23785	-	50
NW_015941516.1	69974	70113	+	139
NW_015941661.1	93281	93541	+	164
NW_015941766.1	25389	25480	-	91
NW_015942306.1	2687	2973	-	261
NW_015942326.1	13798	13927	+	129
NW_015942389.1	2166	2297	-	131
NW_015942552.1	20124	20236	+	112
NW_015942689.1	94494	94629	+	135
NW_015942703.1	17084	17435	-	185
NW_015942802.1	19804	19970	+	85
NW_015943061.1	200354	200404	+	50
NW_015943162.1	36507	36597	+	90
NW_015943185.1	17424	17587	+	163
NW_015943317.1	27139	27431	+	292
NW_015943515.1	6336	6348	-	12

NW_015943700.1	14100	14291	-	191
NW_015943827.1	87323	87563	-	240
NW_015943849.1	9536	9669	+	133
NW_015943920.1	20302	20454	-	152
NW_015944005.1	18316	18372	-	56
NW_015944040.1	55071	55411	+	340
NW_015944518.1	59740	59812	-	72
NW_015944541.1	1143	1288	-	17
NW_015945024.1	24574	24653	-	79
NW_015945148.1	35643	35883	-	145
NW_015945287.1	19273	19320	+	47
NW_015945287.1	19282	19339	+	57
NW_015945449.1	37261	37487	-	226
NW_015945679.1	16679	16871	-	3
NW_015946011.1	23843	23900	+	57
NW_015946040.1	69036	69115	-	79
NW_015946040.1	75318	75378	-	60
NW_015946680.1	14113	14199	-	86
NW_015946706.1	67875	67970	+	95
NW_015946724.1	26210	26298	+	88
NW_015946913.1	24164	24647	-	483
NW_015947026.1	37786	37907	-	121
NW_015947193.1	78236	78248	+	12
NW_015947193.1	78231	78290	+	59
NW_015947295.1	5569	5695	-	126

NW_015947333.1	113537	113614	-	77
NW_015947368.1	68342	68457	-	115
NW_015947370.1	5569	5612	+	43
NW_015947669.1	41570	41649	-	79
NW_015947872.1	6190	6225	-	35
NW_015947872.1	6179	6352	-	173
NW_015948154.1	87871	88021	+	150
NW_015948154.1	63314	63426	-	112
NW_015948225.1	47205	47679	+	249
NW_015948290.1	27033	27096	-	63
NW_015948516.1	20280	20418	-	83
NW_015948707.1	4703	4758	-	55
NW_015948766.1	55863	56024	+	161
NW_015949055.1	10923	10968	-	45
NW_015949221.1	15434	15499	-	65
NW_015949506.1	14598	14733	+	129
NW_015949511.1	37104	37307	+	203
NW_015949578.1	4402	4539	-	137
NW_015950258.1	98904	99019	+	115
NW_015950258.1	98900	99046	+	146
NW_015950258.1	98879	99066	+	187
NW_015950862.1	16096	16260	+	164
NW_015951003.1	33968	34061	+	93
NW_015951020.1	5909	5940	-	31
NW_015951170.1	4110	4311	+	201

NW_015951267.1	8891	9011	+	120
NW_015951453.1	22976	23035	+	59
NW_015951557.1	17377	17521	-	144
NW_015951557.1	7954	8012	+	58
NW_015951571.1	4352	4449	-	97
NW_015951614.1	96614	96681	+	67
NW_015951862.1	20078	20115	+	37
NW_015951971.1	27092	27232	-	132
NW_015952105.1	52120	52223	+	103
NW_015952120.1	32554	32722	-	168
NW_015952157.1	40630	40779	+	149
NW_015952264.1	22109	22244	+	135
NW_015953191.1	170511	170595	-	71
NW_015953580.1	38647	38911	+	264
NW_015953580.1	39252	39494	+	242
NW_015953777.1	63309	63466	+	152
NW_015953989.1	17672	17723	+	51
NW_015954191.1	16110	16251	-	141
NW_015954267.1	41292	41406	-	114
NW_015954336.1	13161	13267	+	106
NW_015954347.1	45060	45128	-	68
NW_015954473.1	21964	22083	-	119
NW_015955074.1	51448	51626	-	178
NW_015955103.1	60693	60810	+	117
NW_015955262.1	2442	2484	+	42

<u>11days post inoculation (Hypo-DMRs)</u>

Scaffold	Start	End	Region strain	Overlapped site
NW_015787321.1	11250	11298	-	48
NW_015787340.1	4177	4305	+	128
NW_015787581.1	16508	16607	+	99
NW_015788158.1	8882	9177	-	295
NW_015788158.1	8879	9164	-	285
NW_015788314.1	21869	22014	-	145
NW_015788608.1	29233	29384	-	151
NW_015788908.1	37663	37744	+	81
NW_015789106.1	62505	62690	-	185
NW_015789259.1	20481	20574	+	93
NW_015789405.1	4656	4870	-	214
NW_015789585.1	7451	7607	+	156
NW_015790311.1	40925	41011	+	55
NW_015790311.1	40895	41029	+	85
NW_015791267.1	5009	5175	+	166
NW_015791267.1	88662	88739	+	77
NW_015791475.1	11302	11346	-	44
NW_015791560.1	4344	4459	+	102
NW_015792039.1	9109	9205	+	96
NW_015792185.1	12713	12771	-	58
NW_015792376.1	37058	37374	-	316

NW_015792431.1	2590	2705	-	115
NW_015792743.1	6752	6874	+	122
NW_015792755.1	13442	13553	+	111
NW_015792755.1	13454	13559	+	105
NW_015793238.1	22079	22246	-	167
NW_015793480.1	6543	6637	+	94
NW_015793573.1	73473	73742	+	269
NW_015793967.1	34767	34861	-	94
NW_015793978.1	13179	13231	-	52
NW_015794174.1	80395	80515	-	120
NW_015794196.1	10563	10666	-	103
NW_015794232.1	24845	24925	-	80
NW_015794278.1	13725	13761	-	36
NW_015794420.1	39008	39131	+	123
NW_015794640.1	35718	35910	+	192
NW_015794673.1	14425	14580	-	155
NW_015794850.1	8590	8766	-	97
NW_015795024.1	21812	21844	-	32
NW_015795042.1	11972	12193	+	221
NW_015795175.1	144913	144953	+	40
NW_015795225.1	7134	7164	+	30
NW_015795761.1	139200	139277	-	77
NW_015795840.1	97173	97198	+	25
NW_015796104.1	46042	46309	+	167
NW_015796104.1	34537	34697	+	160

NW_015796104.1	46037	46326	+	172
NW_015796173.1	5158	5262	+	104
NW_015796367.1	73800	73879	-	79
NW_015796504.1	33057	33205	+	94
NW_015796526.1	59054	59218	+	164
NW_015796928.1	59648	59782	+	104
NW_015797106.1	50852	50953	-	101
NW_015797438.1	1730	1853	+	123
NW_015798089.1	101949	102027	+	78
NW_015798388.1	28694	28828	-	134
NW_015798436.1	17648	17794	-	146
NW_015798436.1	17645	17813	-	168
NW_015798528.1	18426	18507	-	81
NW_015798807.1	13443	13527	-	84
NW_015798807.1	13434	13535	-	101
NW_015798826.1	24894	24958	-	64
NW_015798909.1	8652	8785	+	133
NW_015798995.1	14588	14676	+	88
NW_015799619.1	16523	16600	-	77
NW_015799986.1	57278	57361	+	83
NW_015799986.1	57262	57613	+	351
NW_015800001.1	32157	32401	+	244
NW_015800462.1	13771	14031	-	260
NW_015800821.1	2795	2849	-	54
NW_015801759.1	15401	15482	-	81

NW_015801759.1	15150	15469	-	319
NW_015801924.1	58518	58665	-	147
NW_015803008.1	8498	8719	-	25
NW_015803142.1	17578	17658	+	80
NW_015803437.1	62235	62244	+	9
NW_015803472.1	28674	28735	+	61
NW_015803504.1	53724	53756	-	32
NW_015803671.1	1088	1315	-	227
NW_015803836.1	1641	1892	+	245
NW_015803996.1	23179	23256	+	77
NW_015804409.1	59427	59567	+	140
NW_015804590.1	32395	32593	-	161
NW_015805049.1	39926	40030	+	104
NW_015805071.1	2961	3057	+	96
NW_015805116.1	24735	24936	+	201
NW_015805116.1	24856	24948	+	92
NW_015805172.1	96579	96631	+	52
NW_015805172.1	32515	32647	-	132
NW_015806190.1	40536	40790	-	254
NW_015806450.1	21095	21217	-	122
NW_015806485.1	21524	21579	-	55
NW_015806518.1	131562	131585	-	23
NW_015806601.1	110297	110375	+	78
NW_015807125.1	48353	48693	+	340
NW_015807148.1	8253	8402	+	149

NW_015807649.1	10179	10222	-	43
NW_015807712.1	9603	9675	+	72
NW_015808036.1	1981	2027	+	46
NW_015808036.1	1974	2036	+	62
NW_015808104.1	51368	51471	-	70
NW_015808204.1	24534	24587	-	53
NW_015808372.1	12365	12404	-	39
NW_015808379.1	4425	4671	-	246
NW_015808465.1	13047	13343	-	190
NW_015808999.1	66855	66994	-	139
NW_015809543.1	60121	60233	-	63
NW_015809613.1	11685	11785	-	100
NW_015809667.1	50808	50918	-	110
NW_015809988.1	52213	52255	-	42
NW_015810194.1	43094	43214	+	116
NW_015810259.1	24799	25131	-	332
NW_015810447.1	14761	14842	+	81
NW_015810839.1	52008	52047	+	39
NW_015810917.1	43009	43087	-	78
NW_015811144.1	23566	23645	+	79
NW_015811605.1	9837	9938	-	74
NW_015811619.1	2655	2785	-	130
NW_015811883.1	109744	109892	+	148
NW_015812026.1	13953	14051	-	98
NW_015812256.1	15446	15737	-	291

NW_015812279.1	7641	7675	+	34
NW_015812492.1	56867	57023	-	156
NW_015813067.1	100741	100819	+	78
NW_015813071.1	35281	35299	+	18
NW_015813218.1	35383	35494	-	111
NW_015813270.1	11024	11087	-	63
NW_015813270.1	11203	11524	-	321
NW_015813683.1	4835	4916	-	81
NW_015814572.1	18049	18167	-	78
NW_015814572.1	18076	18189	-	28
NW_015814937.1	53944	53952	-	8
NW_015814964.1	55503	55646	-	143
NW_015815076.1	16189	16270	-	81
NW_015815430.1	122798	123486	-	688
NW_015815745.1	7605	7696	-	91
NW_015815745.1	7551	7701	-	150
NW_015815772.1	8306	8361	-	55
NW_015815911.1	18884	19014	+	130
NW_015815911.1	18840	19047	+	207
NW_015816087.1	26546	26682	-	136
NW_015816936.1	19215	19242	-	27
NW_015817164.1	26626	26680	-	54
NW_015817388.1	4238	4416	-	178
NW_015817641.1	36586	36656	+	70
NW_015817657.1	8384	8603	-	219

NW_015818126.1	2066	2140	+	74
NW_015818386.1	139657	139932	-	275
NW_015818498.1	14944	15045	-	101
NW_015818566.1	15861	16200	+	339
NW_015818789.1	16051	16260	-	209
NW_015818917.1	47634	47801	-	167
NW_015819357.1	2103	2220	+	117
NW_015819880.1	12763	12915	-	152
NW_015820454.1	21308	21463	-	155
NW_015821688.1	64416	64502	-	86
NW_015822001.1	13952	14086	-	88
NW_015822203.1	38579	38675	+	96
NW_015822338.1	17185	17287	-	102
NW_015823466.1	62366	62460	-	94
NW_015823718.1	29391	29457	+	66
NW_015823886.1	4515	4569	-	54
NW_015824162.1	8921	8933	+	12
NW_015824181.1	86696	86783	+	87
NW_015824769.1	9229	9273	+	44
NW_015824779.1	16207	16327	-	120
NW_015825297.1	4778	4796	-	18
NW_015825305.1	49671	49691	-	20
NW_015825357.1	90174	90290	-	116
NW_015825823.1	17823	17959	+	136
NW_015826233.1	17821	18021	+	200

NW_015826970.1	11484	11634	+	150
NW_015828310.1	44860	44960	-	100
NW_015828379.1	2060	2108	+	48
NW_015828379.1	2031	2146	+	115
NW_015828505.1	43492	43568	+	76
NW_015828526.1	5744	5777	+	33
NW_015829266.1	4631	4961	-	184
NW_015829611.1	22139	22177	-	38
NW_015829824.1	7679	7830	+	151
NW_015830513.1	22364	22527	-	35
NW_015831011.1	151605	151690	+	85
NW_015831365.1	5946	6012	+	66
NW_015831798.1	58994	59219	-	188
NW_015832833.1	16607	16793	+	186
NW_015833255.1	32794	33021	-	227
NW_015833943.1	6056	6127	-	71
NW_015834252.1	16243	16379	+	136
NW_015834373.1	3118	3288	-	139
NW_015835578.1	9376	9446	+	70
NW_015835668.1	125867	125996	-	129
NW_015835668.1	125816	125945	-	82
NW_015835809.1	23961	24007	-	46
NW_015836545.1	33839	33952	+	113
NW_015836620.1	8656	8764	+	102
NW_015836634.1	16975	17015	-	23

NW_015836672.1	4969	5066	-	97
NW_015836930.1	129697	129707	+	10
NW_015836949.1	92797	92865	-	68
NW_015836983.1	15994	16095	+	101
NW_015837205.1	37375	37474	+	99
NW_015837861.1	8565	8611	+	46
NW_015838024.1	49454	49467	-	13
NW_015838084.1	3895	3949	-	54
NW_015838488.1	7857	7989	-	132
NW_015839185.1	11923	11992	+	69
NW_015839194.1	38714	38803	+	89
NW_015840179.1	12643	12732	+	89
NW_015840769.1	15507	15646	-	139
NW_015840769.1	15431	15641	-	210
NW_015840790.1	24932	25193	+	261
NW_015840953.1	29675	29818	+	75
NW_015840967.1	33806	33914	+	108
NW_015840967.1	33804	33927	+	123
NW_015841106.1	62304	62462	-	90
NW_015841810.1	38097	38138	+	41
NW_015842213.1	117143	117199	-	56
NW_015842356.1	2963	3087	+	124
NW_015842452.1	64506	64637	+	131
NW_015842764.1	193711	194027	+	316
NW_015843003.1	106496	106811	-	315

NW_015843097.1	137597	137872	-	275
NW_015843537.1	40494	40714	-	220
NW_015843816.1	52517	52582	-	65
NW_015843847.1	120146	120244	-	98
NW_015843929.1	7337	7406	+	69
NW_015843968.1	1179	1306	+	90
NW_015843980.1	27552	27638	-	86
NW_015843980.1	27515	27678	-	163
NW_015844195.1	32795	33029	-	96
NW_015844195.1	32794	32893	-	97
NW_015844808.1	3907	4020	-	113
NW_015846310.1	23352	23466	-	114
NW_015847145.1	49502	49578	+	76
NW_015847674.1	46124	46377	-	253
NW_015847674.1	46143	46323	-	180
NW_015847674.1	76218	76327	+	109
NW_015847674.1	46130	46385	-	255
NW_015847728.1	205404	205467	+	63
NW_015848009.1	80	143	+	63
NW_015848535.1	48914	49058	-	144
NW_015849672.1	64107	64149	-	42
NW_015849778.1	49630	49745	+	115
NW_015850137.1	4699	5223	+	524
NW_015850520.1	4433	4506	-	73
NW_015850992.1	12336	12474	-	138

NW_015851005.1	32728	32792	-	64
NW_015852367.1	39016	39345	-	329
NW_015852991.1	35093	35266	-	93
NW_015853176.1	27207	27303	-	96
NW_015853360.1	64948	64960	-	12
NW_015853692.1	28361	29143	-	782
NW_015853697.1	37473	37591	+	118
NW_015853716.1	13621	13672	+	51
NW_015854573.1	38476	38581	-	68
NW_015854679.1	1711	1991	-	280
NW_015854679.1	1691	2012	-	321
NW_015854682.1	38221	38411	-	131
NW_015855024.1	26155	26296	+	141
NW_015855210.1	44112	44140	+	28
NW_015855478.1	40238	40367	-	129
NW_015856591.1	28809	28914	+	105
NW_015857129.1	8133	8240	+	107
NW_015857473.1	30027	30175	-	148
NW_015857996.1	20901	21044	+	143
NW_015857996.1	12531	12675	+	144
NW_015857996.1	20890	21046	+	156
NW_015858103.1	26070	26133	-	63
NW_015858206.1	77410	77552	+	142
NW_015858469.1	50776	50814	-	38
NW_015858815.1	28447	28467	-	20

NW_015859072.1	90863	90959	+	75
NW_015859277.1	2866	2996	+	130
NW_015859277.1	2854	2990	+	136
NW_015859438.1	67184	67329	+	145
NW_015859623.1	74160	74178	+	18
NW_015859709.1	25204	25230	+	26
NW_015859920.1	17293	17318	-	25
NW_015859920.1	17295	17384	-	89
NW_015859920.1	17285	17432	-	147
NW_015859926.1	4431	4663	+	213
NW_015860027.1	33143	33192	-	49
NW_015860202.1	193020	193145	+	125
NW_015860451.1	29450	29694	-	195
NW_015861237.1	5253	5323	+	39
NW_015861441.1	63904	64168	-	264
NW_015862002.1	64098	64156	-	58
NW_015862386.1	29067	29241	+	174
NW_015862459.1	11748	11917	+	169
NW_015862803.1	52932	53117	+	185
NW_015863452.1	37755	37894	-	139
NW_015863628.1	69872	70059	-	187
NW_015863845.1	11172	11222	-	50
NW_015864256.1	39942	40036	-	94
NW_015864256.1	11482	11546	-	64
NW_015864297.1	10230	10343	-	113

NW_015864497.1	25805	25880	+	75
NW_015864801.1	13723	13815	-	92
NW_015864846.1	36441	36519	+	78
NW_015865027.1	31315	31377	+	62
NW_015865063.1	16476	16897	-	421
NW_015865370.1	4145	4226	-	81
NW_015865370.1	4139	4256	-	117
NW_015865588.1	38456	38504	-	48
NW_015865806.1	18160	18484	-	324
NW_015865964.1	8754	8794	+	40
NW_015866064.1	24605	24755	-	150
NW_015866792.1	15974	16033	+	59
NW_015867007.1	78489	78580	-	91
NW_015868129.1	4344	4439	+	95
NW_015868313.1	27247	27271	+	24
NW_015868554.1	15401	15447	-	46
NW_015868903.1	34211	34335	-	124
NW_015868996.1	108032	108159	+	24
NW_015869440.1	28066	28222	+	156
NW_015869440.1	28070	28118	+	48
NW_015870068.1	40275	40454	-	179
NW_015870340.1	80557	80607	-	50
NW_015871790.1	11444	11623	-	179
NW_015871936.1	27585	27669	-	84
NW_015872748.1	152651	152806	+	155

NW_015872969.1	174741	174795	+	54
NW_015872992.1	24368	24514	-	146
NW_015873709.1	117804	117929	+	125
NW_015873855.1	5075	5155	-	80
NW_015874831.1	79842	79997	+	155
NW_015875056.1	104749	104809	-	60
NW_015875291.1	31799	31852	+	53
NW_015875479.1	17707	17759	+	52
NW_015875479.1	17706	17788	+	82
NW_015875845.1	474	515	+	41
NW_015876049.1	54731	54860	-	129
NW_015876256.1	1331	1511	+	9
NW_015876273.1	22769	23084	-	315
NW_015876689.1	66159	66257	-	98
NW_015876976.1	73754	73816	+	62
NW_015877315.1	104024	104113	+	89
NW_015877332.1	14272	14408	-	136
NW_015877540.1	79325	79563	+	238
NW_015877628.1	34390	34472	+	82
NW_015878971.1	97216	97308	-	92
NW_015879420.1	64929	65095	-	166
NW_015880240.1	17770	17834	-	64
NW_015880286.1	15975	16065	+	90
NW_015880286.1	15969	16088	+	119
NW_015880450.1	99338	99465	+	127

NW_015881275.1	30266	30281	+	15
NW_015881394.1	4366	4426	+	60
NW_015881704.1	2057	2199	-	142
NW_015881861.1	141637	141679	+	42
NW_015882004.1	76573	76681	+	108
NW_015882230.1	50492	50712	-	117
NW_015882749.1	6314	6454	-	140
NW_015883032.1	14155	14427	+	272
NW_015883043.1	7927	8017	+	90
NW_015883454.1	28521	28631	+	110
NW_015883935.1	6959	7068	-	109
NW_015884410.1	2575	2601	-	26
NW_015885042.1	7831	7900	-	69
NW_015885149.1	55014	55040	-	26
NW_015885244.1	35796	35866	+	70
NW_015885483.1	124035	124121	+	86
NW_015885571.1	21990	22161	-	171
NW_015885583.1	1182	1236	+	50
NW_015885797.1	21522	21688	+	166
NW_015885797.1	21524	21695	+	171
NW_015886073.1	21853	22100	+	247
NW_015886146.1	18148	18386	+	127
NW_015886146.1	18128	18365	+	118
NW_015886265.1	62590	62637	+	47
NW_015886542.1	31503	31624	+	121

NW_015886687.1	9078	9206	-	128
NW_015886753.1	57827	58045	-	218
NW_015887022.1	25780	25802	-	22
NW_015887128.1	116942	117082	-	140
NW_015887133.1	218474	218576	-	102
NW_015887170.1	6290	6379	+	89
NW_015887346.1	56554	56674	+	120
NW_015887373.1	176072	176171	+	99
NW_015887552.1	6183	6340	+	157
NW_015887642.1	46748	46811	+	63
NW_015887819.1	20861	21111	-	250
NW_015887838.1	61386	61452	+	66
NW_015887841.1	82797	82868	+	71
NW_015887950.1	18140	18289	+	149
NW_015888215.1	6385	6547	-	162
NW_015888288.1	40825	40866	-	41
NW_015888572.1	29781	29882	+	101
NW_015888663.1	6650	6767	+	117
NW_015888664.1	115042	115142	-	100
NW_015888863.1	8320	8404	+	84
NW_015888899.1	284833	284907	-	74
NW_015889038.1	98352	98471	-	119
NW_015889108.1	10397	10493	-	96
NW_015889159.1	11993	12120	-	96
NW_015889470.1	21735	21785	-	50

NW_015889493.1	13424	13518	-	94
NW_015889739.1	6112	6266	+	130
NW_015889839.1	49346	49490	-	144
NW_015889889.1	114540	114607	+	67
NW_015889899.1	20946	20974	-	28
NW_015890018.1	25654	25722	-	68
NW_015890023.1	21245	21341	+	96
NW_015890026.1	55372	55560	+	19
NW_015890058.1	31916	32174	-	258
NW_015890221.1	13435	13565	-	130
NW_015890487.1	104200	104293	+	93
NW_015890619.1	47845	47949	+	104
NW_015890709.1	94450	94504	+	54
NW_015890836.1	63626	63660	+	34
NW_015891161.1	25359	25417	+	58
NW_015891230.1	40154	40351	+	107
NW_015891300.1	22360	22473	-	113
NW_015891307.1	10715	10836	+	121
NW_015891445.1	24348	24450	+	102
NW_015891723.1	174559	174740	-	165
NW_015891867.1	280323	280467	+	144
NW_015891923.1	26479	26524	-	45
NW_015892338.1	11887	12051	+	164
NW_015893144.1	782	851	+	69
NW_015893144.1	746	985	+	155

NW_015894348.1	49022	49202	+	180
NW_015894348.1	49016	49196	+	180
NW_015894675.1	17400	17823	-	423
NW_015895657.1	79531	79674	-	143
NW_015896893.1	20089	20143	+	54
NW_015897470.1	2238	2312	-	74
NW_015897470.1	2213	2358	-	145
NW_015897856.1	39665	39693	-	28
NW_015897905.1	265	471	+	206
NW_015898182.1	2328	2430	+	53
NW_015898229.1	18517	18564	+	47
NW_015898292.1	1364	1520	-	156
NW_015898644.1	228	326	+	98
NW_015898674.1	2587	2812	+	225
NW_015898700.1	1442	1554	-	112
NW_015898709.1	2371	2451	+	80
NW_015898709.1	2312	2468	+	156
NW_015898709.1	2802	3051	+	249
NW_015898808.1	3669	3723	-	54
NW_015898840.1	6174	6233	+	59
NW_015898943.1	2342	2436	-	94
NW_015899067.1	8472	8551	+	79
NW_015899198.1	139906	140113	+	207
NW_015899445.1	152400	152547	+	147
NW_015899502.1	42264	42335	+	71

NW_015899669.1	38872	38879	+	7
NW_015899892.1	59638	59826	-	92
NW_015899959.1	51755	51876	-	121
NW_015900242.1	15624	15745	+	121
NW_015900453.1	48685	48710	+	25
NW_015900580.1	5321	5966	-	497
NW_015900631.1	38763	38808	+	45
NW_015900660.1	61380	61450	-	70
NW_015900715.1	46229	46546	+	317
NW_015900953.1	192869	193021	+	52
NW_015900953.1	194055	194229	+	61
NW_015900953.1	192869	193021	+	99
NW_015900953.1	194055	194229	+	112
NW_015901427.1	19677	19744	-	67
NW_015901544.1	15884	15959	-	75
NW_015901544.1	16495	16580	-	85
NW_015901793.1	53687	53826	-	139
NW_015901793.1	53683	53848	-	165
NW_015901933.1	149363	149448	+	85
NW_015902347.1	18526	18595	-	69
NW_015902526.1	23497	23561	+	64
NW_015902585.1	25730	25813	+	83
NW_015902628.1	19781	19897	-	116
NW_015902628.1	19770	19915	-	145
NW_015902670.1	77986	78181	+	195

NW_015902670.1	78000	78213	+	213
NW_015902845.1	2806	3103	+	297
NW_015902988.1	49041	49132	-	91
NW_015903184.1	5839	6231	+	392
NW_015903184.1	14636	14721	-	85
NW_015903238.1	67234	67257	+	23
NW_015903270.1	26194	26310	-	116
NW_015903347.1	99590	99739	+	149
NW_015903924.1	21175	21474	-	299
NW_015904002.1	45438	45569	+	131
NW_015904216.1	166434	166838	-	404
NW_015904250.1	189421	189574	+	153
NW_015904437.1	16753	17187	-	434
NW_015904444.1	31300	31480	+	180
NW_015904568.1	23478	23574	-	96
NW_015904717.1	69264	69593	-	329
NW_015904731.1	22008	22194	+	186
NW_015904757.1	18519	18555	+	36
NW_015904798.1	234459	234584	-	125
NW_015904876.1	23183	23345	-	162
NW_015905192.1	108621	108988	+	367
NW_015905276.1	101018	101145	-	127
NW_015905476.1	48831	48904	+	73
NW_015905711.1	8248	8421	+	173
NW_015905785.1	2506	2637	+	131

NW_015905863.1	7819	7894	+	75
NW_015905884.1	104677	104802	-	125
NW_015905989.1	14192	14231	+	39
NW_015906190.1	107599	107746	-	147
NW_015906190.1	108127	108304	-	177
NW_015906190.1	107563	107754	-	191
NW_015906190.1	108127	108304	-	177
NW_015906202.1	19692	19763	+	71
NW_015906221.1	44856	44924	+	68
NW_015906401.1	39761	39988	+	227
NW_015906588.1	39285	39342	+	57
NW_015906621.1	116527	116578	+	51
NW_015906998.1	14279	14360	+	81
NW_015907210.1	50078	50105	+	27
NW_015907264.1	27602	27663	-	61
NW_015907282.1	58409	58437	-	28
NW_015907282.1	58405	58446	-	41
NW_015907531.1	2182	2530	-	348
NW_015907531.1	2336	2520	-	184
NW_015907531.1	2196	2554	-	358
NW_015907600.1	14168	14237	+	69
NW_015907711.1	4521	4551	-	30
NW_015908027.1	8239	8353	-	114
NW_015908246.1	15178	15282	-	104
NW_015908282.1	39442	39467	-	25

NW_015908282.1	39433	40162	-	729
NW_015908351.1	29577	29606	+	29
NW_015908367.1	13505	13614	+	109
NW_015908580.1	29419	29623	+	170
NW_015908629.1	6456	6492	-	36
NW_015908633.1	37121	37141	-	20
NW_015908648.1	25653	25787	-	134
NW_015908648.1	25616	25847	-	231
NW_015908700.1	76637	76761	+	124
NW_015908714.1	97428	97674	-	246
NW_015908769.1	40809	40852	+	43
NW_015908843.1	25768	26178	+	410
NW_015908859.1	94872	95003	-	131
NW_015908902.1	61452	61560	+	108
NW_015908995.1	185560	185596	-	36
NW_015909022.1	6329	6421	-	67
NW_015909244.1	8304	8465	+	85
NW_015909362.1	54323	54404	+	81
NW_015909875.1	46627	46751	-	124
NW_015909944.1	212643	212707	-	64
NW_015910045.1	6129	6188	+	59
NW_015910130.1	3180	3331	+	151
NW_015910200.1	26233	26296	+	63
NW_015910210.1	52589	52631	-	42
NW_015910295.1	21807	21855	+	48

NW_015910363.1	49192	49256	+	64
NW_015910399.1	11806	11870	-	64
NW_015910518.1	52237	52402	-	165
NW_015910566.1	122419	122554	-	135
NW_015910852.1	11675	11999	+	324
NW_015911030.1	1716	1844	+	128
NW_015911510.1	53469	53545	+	76
NW_015911602.1	41266	41300	-	34
NW_015911649.1	25678	25787	-	109
NW_015911663.1	69520	69641	-	67
NW_015911677.1	44341	44466	-	125
NW_015912095.1	74918	75049	-	131
NW_015912131.1	56864	56944	+	80
NW_015912177.1	10027	10091	+	64
NW_015912265.1	47267	47320	-	53
NW_015912332.1	20532	20631	-	43
NW_015912332.1	11009	11207	-	198
NW_015912332.1	20532	20631	-	55
NW_015912346.1	5680	5720	-	40
NW_015912591.1	20889	21105	+	216
NW_015913254.1	114854	114999	-	145
NW_015913386.1	61294	61357	+	63
NW_015913441.1	28883	29029	+	146
NW_015913540.1	24393	24537	-	144
NW_015913909.1	60040	60139	+	99

NW_015913926.1	93001	93107	-	106
NW_015913926.1	92994	93101	-	107
NW_015913926.1	92986	93104	-	118
NW_015913941.1	28217	28360	-	79
NW_015914025.1	37162	37245	+	83
NW_015914029.1	22068	22178	-	110
NW_015914701.1	32370	32429	+	59
NW_015914758.1	128282	128321	-	39
NW_015914776.1	21717	21743	+	26
NW_015914908.1	91262	91340	-	78
NW_015914974.1	7729	7976	-	247
NW_015914974.1	7751	8019	-	268
NW_015915042.1	27428	27533	+	105
NW_015915232.1	73764	73806	+	42
NW_015915293.1	65258	65617	-	359
NW_015915346.1	24505	24538	-	33
NW_015915811.1	36595	36770	-	175
NW_015915811.1	33774	34034	+	260
NW_015915811.1	36595	36770	-	129
NW_015915893.1	14576	14715	+	139
NW_015915980.1	59639	59736	-	97
NW_015916170.1	21223	21367	-	144
NW_015916178.1	26236	26368	+	132
NW_015916259.1	32218	32270	-	52
NW_015916273.1	32080	32243	+	163

NW_015916311.1	35711	35777	+	66
NW_015916429.1	14218	14366	-	148
NW_015916429.1	14219	14364	-	145
NW_015916578.1	26725	26872	+	111
NW_015916602.1	29685	29805	-	120
NW_015916713.1	6686	6802	+	116
NW_015916713.1	6692	6811	+	119
NW_015916964.1	23039	23124	+	85
NW_015916966.1	31006	31054	+	48
NW_015917019.1	33321	33532	-	211
NW_015917026.1	31750	31836	-	86
NW_015917354.1	51885	52128	+	243
NW_015917413.1	116961	117043	+	82
NW_015917413.1	116887	117057	+	170
NW_015917456.1	1690	1766	-	76
NW_015917480.1	27266	27348	-	82
NW_015917596.1	59578	59671	-	93
NW_015917744.1	5680	5752	-	72
NW_015917744.1	5650	5757	-	107
NW_015917758.1	33374	33609	-	235
NW_015918253.1	86855	86951	-	96
NW_015918332.1	72342	72680	+	276
NW_015918553.1	8728	8827	-	99
NW_015918723.1	11734	11830	-	96
NW_015919160.1	20829	20992	-	163

NW_015919160.1	20841	20990	-	149
NW_015919160.1	20805	21015	-	210
NW_015919368.1	88138	88266	+	128
NW_015919412.1	47673	47791	+	118
NW_015919444.1	22528	22595	+	67
NW_015919549.1	4943	5059	-	116
NW_015919696.1	14759	14834	+	75
NW_015919794.1	5956	6052	+	96
NW_015919812.1	86426	86486	-	60
NW_015919812.1	40714	40917	-	203
NW_015920182.1	6219	6276	+	57
NW_015920700.1	21780	21856	-	76
NW_015920784.1	35420	35515	-	95
NW_015920802.1	11855	12089	+	234
NW_015920827.1	143949	144062	-	113
NW_015920870.1	22532	22710	+	178
NW_015920937.1	16163	16262	+	99
NW_015921297.1	17443	17482	-	39
NW_015921331.1	926	1033	+	107
NW_015921443.1	15260	15410	+	150
NW_015921570.1	23828	23937	+	109
NW_015921570.1	23822	24022	+	200
NW_015921656.1	19901	20008	-	61
NW_015921720.1	2329	2501	+	44
NW_015921739.1	3107	3168	+	61

NW_015921800.1	47679	47796	-	117
NW_015921832.1	49369	49476	-	107
NW_015921901.1	99337	99503	-	166
NW_015922079.1	11413	11501	+	88
NW_015922099.1	5490	5608	-	118
NW_015922109.1	9267	9322	+	55
NW_015922165.1	8071	8164	-	93
NW_015922253.1	38404	38484	+	80
NW_015922346.1	17044	17084	+	40
NW_015922458.1	47458	47502	-	44
NW_015922458.1	47475	47508	-	33
NW_015922467.1	21925	22009	+	84
NW_015922631.1	6280	6306	+	26
NW_015922679.1	16449	16605	+	156
NW_015922866.1	30825	30922	+	97
NW_015922866.1	30770	30925	+	155
NW_015923145.1	39761	39912	-	151
NW_015923293.1	28983	29183	-	200
NW_015923468.1	13450	13639	-	189
NW_015923552.1	42706	42753	+	24
NW_015923671.1	26962	27048	+	86
NW_015924014.1	32490	32646	-	156
NW_015924019.1	9451	9558	-	71
NW_015924203.1	13428	13541	+	113
NW_015924494.1	70671	70776	+	105

NW_015924571.1	2551	2578	+	27
NW_015924798.1	29826	29901	-	75
NW_015924819.1	30895	31088	+	193
NW_015924910.1	40692	40735	+	43
NW_015924919.1	27777	27888	+	111
NW_015924987.1	29781	29814	+	33
NW_015925345.1	110750	110890	-	79
NW_015925504.1	63941	64008	+	67
NW_015925600.1	7248	7328	-	80
NW_015925709.1	9488	9537	+	49
NW_015925709.1	9449	9518	+	69
NW_015925709.1	9374	9551	+	177
NW_015926076.1	106909	107053	-	144
NW_015926155.1	12067	12157	-	90
NW_015926258.1	7200	7324	+	124
NW_015926271.1	15343	15452	+	109
NW_015926301.1	49766	49828	-	62
NW_015926596.1	66403	66506	-	103
NW_015926941.1	13577	13699	+	122
NW_015926945.1	50470	50506	-	36
NW_015927047.1	12632	12777	-	145
NW_015927135.1	17093	17201	-	108
NW_015927326.1	16961	17069	-	108
NW_015927546.1	16816	16939	-	123
NW_015927764.1	32531	32589	+	58

NW_015928099.1	41458	41575	-	117
NW_015928099.1	32250	32370	+	120
NW_015928153.1	33107	33127	-	20
NW_015928200.1	11026	11340	+	314
NW_015928334.1	60015	60098	+	83
NW_015928392.1	117802	117939	+	102
NW_015928560.1	103853	103961	-	108
NW_015928732.1	91642	91738	-	96
NW_015928944.1	29888	29953	+	65
NW_015929260.1	143845	143904	-	59
NW_015929377.1	246870	246937	+	67
NW_015929572.1	4449	4521	+	72
NW_015929630.1	66378	66475	+	97
NW_015929769.1	9472	9531	-	59
NW_015929871.1	23750	23817	+	67
NW_015929922.1	29396	29598	-	202
NW_015929922.1	29386	29608	-	222
NW_015930022.1	108325	108720	+	395
NW_015930022.1	108331	108734	+	403
NW_015930033.1	56774	56866	+	92
NW_015930226.1	34765	34842	-	77
NW_015930915.1	48192	48377	+	185
NW_015931220.1	56813	56854	-	41
NW_015931389.1	86313	86371	-	58
NW_015931456.1	67683	67932	-	249

NW_015931866.1	44503	44600	-	97
NW_015931902.1	36013	36041	+	28
NW_015932008.1	29534	29630	+	96
NW_015932131.1	45776	46000	-	224
NW_015932296.1	116068	116255	+	187
NW_015932329.1	10513	10589	-	76
NW_015932399.1	5232	5446	-	214
NW_015932445.1	13076	13130	-	54
NW_015932618.1	20336	20421	-	85
NW_015932618.1	20340	20429	-	89
NW_015932765.1	8261	8284	+	23
NW_015933163.1	100546	100586	+	40
NW_015933189.1	18955	19054	+	54
NW_015933252.1	15608	15809	-	201
NW_015933796.1	8757	8907	+	150
NW_015933796.1	8774	8874	+	100
NW_015934082.1	76786	76928	+	142
NW_015934220.1	9251	9438	-	187
NW_015934651.1	12442	12548	-	106
NW_015934924.1	4188	4282	-	94
NW_015934924.1	4185	4592	-	407
NW_015935052.1	39042	39135	-	93
NW_015935209.1	70269	70448	+	136
NW_015935321.1	17461	17612	+	151
NW_015935532.1	166682	166798	+	116

NW_015935532.1	166632	166810	+	178
NW_015935578.1	12334	12530	+	196
NW_015935622.1	26437	26499	+	62
NW_015935920.1	21676	21792	+	116
NW_015935958.1	9799	9974	+	175
NW_015936163.1	66167	66579	+	412
NW_015936181.1	23931	24015	+	79
NW_015936350.1	7464	7595	-	131
NW_015936576.1	2315	2445	+	109
NW_015936593.1	25825	25938	+	113
NW_015936770.1	68354	68463	+	109
NW_015936986.1	29432	29589	-	157
NW_015937099.1	36950	36994	+	44
NW_015937099.1	36904	36975	+	71
NW_015937130.1	10472	10515	+	43
NW_015937264.1	12559	12635	+	76
NW_015937308.1	46679	46753	+	74
NW_015937369.1	8447	8491	+	44
NW_015937464.1	39789	39885	-	96
NW_015937723.1	14291	14472	-	181
NW_015937951.1	7428	7480	-	52
NW_015938092.1	29888	30266	+	378
NW_015938092.1	29907	30285	+	378
NW_015938092.1	29880	30283	+	403
NW_015938099.1	5360	5423	-	63

NW_015938182.1	16891	16985	+	94
NW_015938192.1	49162	49256	+	94
NW_015938235.1	20463	20502	-	39
NW_015938275.1	59708	59831	+	123
NW_015938719.1	11191	11360	-	147
NW_015938902.1	13939	14016	+	77
NW_015939169.1	17188	17341	-	153
NW_015939258.1	45556	45628	+	72
NW_015939609.1	14356	14400	+	44
NW_015939676.1	13048	13156	-	108
NW_015939812.1	65108	65164	-	56
NW_015939934.1	22557	22620	-	63
NW_015940333.1	134870	134886	+	16
NW_015940333.1	134839	134891	+	52
NW_015940858.1	30157	30317	+	160
NW_015941126.1	259268	259414	+	146
NW_015941377.1	5336	5395	+	59
NW_015941591.1	5560	5701	+	141
NW_015941591.1	5552	5726	+	174
NW_015941725.1	42911	43041	+	6
NW_015942133.1	36590	37110	+	520
NW_015942133.1	36602	37112	+	510
NW_015942170.1	16214	16278	+	64
NW_015942429.1	12541	12706	-	165
NW_015942579.1	18341	18384	-	43

NW_015942759.1	22395	22491	+	96
NW_015942850.1	18406	18515	-	90
NW_015942910.1	37544	37616	+	72
NW_015942939.1	60093	60133	-	40
NW_015943283.1	50920	51014	+	67
NW_015943319.1	22612	22855	+	243
NW_015943368.1	36951	37016	-	65
NW_015943481.1	12802	12894	+	92
NW_015943527.1	66672	66692	-	20
NW_015943534.1	48552	48620	-	68
NW_015943606.1	13333	13422	-	89
NW_015943808.1	66423	66537	-	114
NW_015943817.1	28091	28237	+	88
NW_015943877.1	15959	16099	+	140
NW_015944017.1	19229	19352	-	123
NW_015944293.1	50191	50289	-	98
NW_015944350.1	16707	16759	-	52
NW_015944696.1	145794	145938	-	144
NW_015945193.1	6593	6696	+	103
NW_015945662.1	36138	36371	+	233
NW_015946274.1	10633	10646	-	13
NW_015946279.1	26876	26926	-	50
NW_015946636.1	53328	53428	-	100
NW_015946810.1	6397	6529	-	132
NW_015946810.1	6404	6515	-	111

NW_015946810.1	6389	6533	-	144
NW_015946812.1	73237	73393	-	156
NW_015946880.1	20947	20975	-	28
NW_015946880.1	20794	21003	-	209
NW_015946952.1	6058	6177	+	119
NW_015946985.1	28323	28428	+	105
NW_015946985.1	28280	28454	+	174
NW_015947026.1	39462	39720	-	258
NW_015947423.1	10962	11147	+	185
NW_015947764.1	29995	30124	-	88
NW_015947801.1	24778	24859	-	81
NW_015948084.1	14803	14920	-	117
NW_015948152.1	19258	19318	+	60
NW_015948156.1	38575	38687	+	112
NW_015948395.1	16839	16886	+	47
NW_015948395.1	16827	16933	+	61
NW_015948504.1	7259	7374	+	115
NW_015948505.1	34682	34954	-	272
NW_015948576.1	4639	4749	-	110
NW_015948940.1	33202	33374	-	172
NW_015949307.1	7257	7319	-	62
NW_015949414.1	14712	14835	-	123
NW_015949460.1	29060	29243	-	183
NW_015949472.1	17497	17664	-	167
NW_015949923.1	9159	9298	+	139

NW_015949979.1	15274	15398	+	124
NW_015950014.1	93362	93496	-	134
NW_015950258.1	83988	84032	+	44
NW_015950258.1	81262	81345	+	83
NW_015950372.1	4775	4788	+	13
NW_015950650.1	5200	5289	+	89
NW_015950791.1	40241	40335	+	94
NW_015951222.1	170219	170373	-	154
NW_015951222.1	170226	170327	-	101
NW_015951500.1	67567	67637	+	70
NW_015951599.1	21422	21493	-	71
NW_015952009.1	16295	16427	-	129
NW_015952274.1	52957	53186	+	105
NW_015952386.1	19795	19825	+	30
NW_015952624.1	161686	161777	+	91
NW_015952863.1	19393	19491	+	98
NW_015952919.1	10085	10360	-	275
NW_015953711.1	14148	14265	-	117
NW_015953731.1	14738	14848	-	110
NW_015953755.1	7914	8080	-	166
NW_015953888.1	51673	51871	-	198
NW_015954099.1	26012	26151	+	139
NW_015954131.1	56718	56755	+	37
NW_015954166.1	74189	74274	+	85
NW_015954178.1	11627	11695	-	68

NW_015954188.1	7277	7318	-	41
NW_015954330.1	9572	9607	+	35
NW_015954642.1	11813	12013	+	200
NW_015954709.1	26296	26482	-	186
NW_015954748.1	4679	4753	+	74
NW_015954833.1	8767	9140	-	373
NW_015954893.1	42364	42548	-	184

13days post inoculation (Hyper-DMRs)

Scaffold	Start	End	Region stain	Overlapped site
NW_015787321.1	7205	7278	+	73
NW_015788007.1	9091	9144	+	53
NW_015788007.1	9016	9153	+	137
NW_015788595.1	17030	17128	+	98
NW_015788744.1	15055	15429	+	327
NW_015788759.1	29198	29302	+	104
NW_015789325.1	21640	21751	+	111
NW_015789325.1	21626	21742	+	116
NW_015789464.1	1994	2094	-	100
NW_015789504.1	46967	47075	+	108
NW_015789933.1	71048	71262	+	214
NW_015790157.1	43479	43551	-	72
NW_015790612.1	43359	43430	-	71
NW_015790612.1	43362	43502	-	71

NW_015790886.1	153083	153277	+	194
NW_015790901.1	202854	202987	-	133
NW_015791039.1	91899	92017	+	118
NW_015791184.1	54235	54348	+	113
NW_015791379.1	5701	6050	+	349
NW_015791942.1	1623	1689	+	66
NW_015792222.1	7703	7750	-	47
NW_015792222.1	7708	7824	-	116
NW_015792341.1	105264	105394	+	130
NW_015792507.1	35759	35852	+	93
NW_015792603.1	17266	17391	+	125
NW_015792743.1	7815	7898	+	83
NW_015792755.1	13352	13559	-	207
NW_015793027.1	26590	26730	-	140
NW_015793367.1	16943	17048	+	105
NW_015793367.1	16891	17058	+	167
NW_015793517.1	26433	26506	-	73
NW_015793762.1	13520	13654	-	134
NW_015793787.1	18154	18235	-	81
NW_015793787.1	18156	18234	-	78
NW_015793957.1	6361	6757	-	396
NW_015794304.1	85285	85384	-	99
NW_015794452.1	11058	11164	+	102
NW_015794452.1	11049	11166	+	104
NW_015794790.1	9618	9729	-	111

NW_015795042.1	12055	12176	+	121
NW_015795042.1	12015	12207	+	192
NW_015795055.1	20781	20814	-	33
NW_015795619.1	85828	85945	-	117
NW_015795619.1	85818	85952	-	134
NW_015795761.1	40542	40673	+	131
NW_015796036.1	7361	7518	-	37
NW_015796100.1	7579	7632	-	53
NW_015796100.1	7517	7633	-	116
NW_015796427.1	1498	1629	+	131
NW_015796427.1	1497	1690	+	193
NW_015796427.1	1491	1700	+	209
NW_015796699.1	23775	23805	+	30
NW_015796851.1	13816	13884	-	68
NW_015797386.1	7266	7407	-	141
NW_015797386.1	7260	7455	-	195
NW_015797574.1	128440	128611	-	171
NW_015797574.1	128438	128605	-	167
NW_015797574.1	128457	128603	-	146
NW_015797895.1	32185	32323	+	118
NW_015797915.1	4993	5195	-	202
NW_015797941.1	4155	4295	+	140
NW_015798028.1	10453	10518	+	65
NW_015798051.1	4758	4934	+	176
NW_015798126.1	17543	17614	-	71

NW_015798305.1	18513	18643	-	130
NW_015798449.1	62414	62482	-	68
NW_015798800.1	12865	12923	-	58
NW_015798967.1	19047	19137	-	90
NW_015799112.1	2058	2185	+	115
NW_015799128.1	46336	46357	-	21
NW_015799227.1	6496	6752	+	256
NW_015799227.1	6527	6744	+	217
NW_015799227.1	6318	6748	+	430
NW_015800356.1	2749	2803	+	54
NW_015800471.1	129625	129781	+	156
NW_015800675.1	56378	56408	+	30
NW_015800693.1	19221	19304	-	83
NW_015800721.1	6643	6778	+	135
NW_015802527.1	12556	12586	-	30
NW_015802684.1	18532	18604	-	72
NW_015802684.1	18521	18592	-	71
NW_015802852.1	6602	6754	+	152
NW_015802924.1	76399	76466	+	67
NW_015802968.1	34213	34366	+	153
NW_015802968.1	34118	34362	+	244
NW_015803142.1	17578	17666	+	88
NW_015803772.1	2413	2538	-	125
NW_015803990.1	11328	11526	+	198
NW_015804544.1	29298	29539	-	241

NW_015804601.1	13116	13232	-	116
NW_015804697.1	39932	40083	-	151
NW_015804894.1	110523	110558	+	35
NW_015804894.1	110434	110605	+	171
NW_015804904.1	73202	73347	+	145
NW_015804904.1	73315	73362	+	47
NW_015804904.1	73191	73395	+	204
NW_015804904.1	87185	87246	-	61
NW_015804964.1	13063	13076	+	13
NW_015805110.1	21279	21396	-	117
NW_015805110.1	21695	21834	-	139
NW_015805172.1	198774	198843	+	69
NW_015805172.1	199237	199316	+	72
NW_015805172.1	198783	198850	+	67
NW_015805172.1	199226	199391	+	83
NW_015805777.1	11095	11270	+	175
NW_015805777.1	11115	11244	+	129
NW_015806404.1	19781	19856	-	75
NW_015806572.1	93579	93696	+	117
NW_015806586.1	39615	39640	-	25
NW_015806601.1	113050	113254	-	204
NW_015806601.1	104037	104087	+	50
NW_015806601.1	104242	104254	+	12
NW_015807343.1	29475	29635	+	160
NW_015807343.1	29508	29645	+	137

NW_015807453.1	13854	14049	-	195
NW_015807521.1	321465	321587	+	122
NW_015807689.1	14423	14502	+	66
NW_015807931.1	27049	27137	-	88
NW_015808043.1	5574	5701	+	127
NW_015808212.1	116281	116380	-	99
NW_015808212.1	116306	116386	-	80
NW_015808541.1	201153	201352	-	199
NW_015808541.1	201156	201357	-	201
NW_015808673.1	2999	3137	-	138
NW_015808745.1	27632	27809	-	177
NW_015808839.1	50988	51064	+	51
NW_015808839.1	50959	51103	+	80
NW_015808958.1	5858	5917	-	59
NW_015809102.1	11181	11213	-	32
NW_015809102.1	11177	11242	-	65
NW_015809415.1	4281	4326	+	45
NW_015809415.1	4279	4325	+	46
NW_015809922.1	252682	252757	-	75
NW_015810161.1	17900	17948	-	48
NW_015810161.1	17890	17956	-	66
NW_015810161.1	49888	50061	-	173
NW_015810190.1	11836	11977	-	141
NW_015810838.1	21668	21855	+	187
NW_015810838.1	21597	21891	+	294

NW_015811033.1	251920	252208	+	205
NW_015811093.1	30678	30772	-	94
NW_015811093.1	30674	30810	-	136
NW_015811386.1	29918	29939	+	21
NW_015811548.1	78404	78452	-	48
NW_015811614.1	3897	4012	-	115
NW_015811619.1	2629	3002	-	373
NW_015811734.1	7206	7268	+	62
NW_015811734.1	7204	7242	+	38
NW_015811734.1	7214	7262	+	48
NW_015812212.1	46411	46518	+	68
NW_015812377.1	17364	17507	+	143
NW_015812680.1	2265	2458	-	193
NW_015812697.1	3683	3897	+	214
NW_015813067.1	100724	100844	+	120
NW_015813263.1	12420	12500	-	80
NW_015813936.1	25649	25723	-	74
NW_015813936.1	25636	25716	-	80
NW_015814379.1	1881	1942	-	61
NW_015814787.1	46103	46163	-	60
NW_015814787.1	46086	46189	-	103
NW_015815679.1	41773	41849	+	76
NW_015815718.1	60045	60322	-	203
NW_015816087.1	26546	26683	-	137
NW_015816278.1	19562	19734	-	172

NW_015816487.1	17679	17808	-	129
NW_015816487.1	5285	5352	-	67
NW_015816682.1	35868	35938	+	70
NW_015816682.1	35865	35936	+	71
NW_015816700.1	14569	14686	-	117
NW_015816849.1	23857	23949	+	92
NW_015816896.1	23559	23738	-	179
NW_015816896.1	23465	23760	-	295
NW_015817414.1	16906	17018	+	112
NW_015817439.1	23129	23313	+	184
NW_015818032.1	38778	38889	-	111
NW_015818139.1	19045	19334	+	289
NW_015818172.1	16686	16740	+	54
NW_015818172.1	17880	17960	+	80
NW_015818422.1	3433	3630	+	197
NW_015818755.1	17356	17406	-	50
NW_015820254.1	11224	11440	+	158
NW_015820468.1	56882	57069	-	148
NW_015820785.1	95778	95993	+	215
NW_015820820.1	44235	44344	+	109
NW_015820820.1	44222	44362	+	140
NW_015820853.1	2760	2857	-	97
NW_015821299.1	54140	54229	+	89
NW_015821299.1	54142	54249	+	107
NW_015821335.1	120	329	+	209

NW_015821757.1	26800	26903	+	103
NW_015821757.1	26642	26912	+	270
NW_015822450.1	36243	36323	+	80
NW_015822502.1	19477	19565	+	88
NW_015823041.1	52860	52923	-	63
NW_015823886.1	4510	4583	-	73
NW_015824270.1	55665	55788	+	123
NW_015824421.1	71923	71979	-	56
NW_015824421.1	71735	71977	-	242
NW_015824562.1	64837	64922	-	85
NW_015825368.1	269137	269250	-	113
NW_015825823.1	30544	30805	-	261
NW_015826290.1	25402	25535	-	133
NW_015826290.1	25407	25526	-	119
NW_015826295.1	12353	12446	+	93
NW_015826406.1	97166	97318	+	152
NW_015826406.1	97164	97365	+	201
NW_015826758.1	23781	23924	+	143
NW_015827065.1	20933	21078	-	145
NW_015827184.1	28355	28503	-	148
NW_015828298.1	19032	19245	+	213
NW_015828413.1	7598	7849	+	178
NW_015829266.1	4631	4961	-	184
NW_015829606.1	35093	35181	-	88
NW_015829606.1	35080	35189	-	109

NW_015829611.1	22282	22409	-	127
NW_015829845.1	84142	84275	-	133
NW_015830304.1	57644	57719	+	57
NW_015830579.1	14064	14216	+	152
NW_015831087.1	6312	6466	-	154
NW_015831087.1	6306	6482	-	176
NW_015831229.1	185754	185837	-	83
NW_015831320.1	8163	8264	-	101
NW_015831320.1	8171	8267	-	96
NW_015831523.1	21307	21487	+	180
NW_015831606.1	180170	180207	+	37
NW_015831967.1	5170	5214	+	44
NW_015831977.1	45863	46007	+	144
NW_015832017.1	48893	48962	+	69
NW_015832168.1	18086	18323	-	222
NW_015833229.1	7781	8043	+	262
NW_015833255.1	14749	14824	-	49
NW_015833603.1	43029	43130	+	101
NW_015833681.1	106432	106492	+	60
NW_015834311.1	123789	123895	-	106
NW_015834644.1	30340	30495	+	155
NW_015834645.1	16559	16651	-	92
NW_015834645.1	16538	16660	-	122
NW_015835004.1	128451	128597	-	41
NW_015835037.1	6916	6968	-	52

NW_015835713.1	231	346	-	115
NW_015835713.1	234	381	-	147
NW_015835792.1	5774	5850	+	76
NW_015836116.1	19609	19711	+	102
NW_015836299.1	143906	143959	-	53
NW_015836502.1	43149	43196	+	47
NW_015837020.1	1549	1661	+	112
NW_015837315.1	81579	81764	+	185
NW_015837398.1	2723	2890	-	167
NW_015837544.1	49599	49828	+	229
NW_015837707.1	57496	57515	+	19
NW_015838891.1	16539	16595	+	56
NW_015839770.1	11384	11467	-	83
NW_015840209.1	1422	1532	-	15
NW_015840234.1	41973	42101	-	128
NW_015840790.1	25022	25184	+	162
NW_015840790.1	26251	26299	+	48
NW_015840790.1	26454	26478	+	24
NW_015840790.1	24948	25187	+	239
NW_015840790.1	25292	25346	+	54
NW_015840790.1	26262	26321	+	59
NW_015840790.1	24939	25194	+	255
NW_015840790.1	26225	26336	+	111
NW_015840953.1	33194	33349	+	155
NW_015841508.1	61196	61349	+	153

NW_015841918.1	75578	75826	+	248
NW_015842217.1	49748	49819	+	71
NW_015842217.1	49725	49847	+	122
NW_015842217.1	82709	82730	-	21
NW_015843003.1	18653	18791	+	138
NW_015844965.1	149587	149619	+	32
NW_015845424.1	3233	3372	+	139
NW_015845451.1	44300	44437	-	137
NW_015845654.1	8294	8377	-	83
NW_015846305.1	4427	4657	-	230
NW_015846322.1	24366	24436	-	70
NW_015846361.1	203097	203111	+	14
NW_015846564.1	1437	1593	-	156
NW_015846918.1	14286	14420	+	134
NW_015847085.1	2525	2647	+	122
NW_015847361.1	64348	64592	+	244
NW_015847361.1	64339	64612	+	273
NW_015847728.1	211766	211839	+	51
NW_015848019.1	11639	11872	+	233
NW_015848107.1	12604	12720	+	116
NW_015848535.1	43753	44078	-	325
NW_015848535.1	43745	44083	-	338
NW_015848535.1	43744	44100	-	356
NW_015849438.1	6773	7044	+	271
NW_015849664.1	59718	59833	+	71

NW_015849728.1	18166	18355	+	189
NW_015850380.1	6405	6478	+	73
NW_015850448.1	180946	180992	+	46
NW_015850448.1	180948	180977	+	29
NW_015850448.1	180944	180987	+	43
NW_015851661.1	14033	14143	-	110
NW_015851919.1	76014	76075	-	61
NW_015851919.1	76023	76092	-	69
NW_015852199.1	11397	11506	-	109
NW_015852199.1	11408	11503	-	95
NW_015852267.1	5611	5759	+	148
NW_015853183.1	44063	44110	+	47
NW_015853374.1	19437	19548	-	111
NW_015853460.1	45487	45612	-	125
NW_015853673.1	82954	83398	+	374
NW_015853692.1	28361	29147	-	786
NW_015853874.1	40508	40553	+	45
NW_015854679.1	1691	2016	-	325
NW_015855009.1	200513	200970	-	457
NW_015855617.1	23889	23972	-	83
NW_015855617.1	23809	24003	-	188
NW_015857144.1	42221	42302	+	81
NW_015857748.1	19460	19482	-	22
NW_015857847.1	3334	3444	-	110
NW_015857996.1	12565	12681	+	116

NW_015857996.1	12562	12657	+	95
NW_015857996.1	20895	21044	+	149
NW_015857996.1	20875	21046	+	171
NW_015858170.1	94796	94976	+	164
NW_015858372.1	147416	147477	+	61
NW_015858629.1	72874	72943	-	69
NW_015858696.1	77168	77252	-	84
NW_015858696.1	67848	68062	+	214
NW_015858696.1	77134	77254	-	120
NW_015858792.1	60126	60247	+	121
NW_015859129.1	5504	5569	-	65
NW_015859307.1	368	411	-	43
NW_015860150.1	2022	2138	+	116
NW_015860285.1	50777	50860	+	83
NW_015860285.1	50784	51118	+	255
NW_015860585.1	20612	20715	+	103
NW_015860772.1	23974	24021	+	47
NW_015860837.1	729	777	+	48
NW_015861076.1	15088	15133	-	45
NW_015861576.1	95011	95206	+	133
NW_015861840.1	1938	2022	+	84
NW_015861840.1	1937	2039	+	102
NW_015861840.1	1770	2044	+	208
NW_015862391.1	24338	24539	-	201
NW_015862824.1	3299	3428	+	129

NW_015862830.1	153756	153827	+	71
NW_015862830.1	153750	153828	+	78
NW_015863028.1	7976	8018	+	42
NW_015863338.1	32230	32332	-	102
NW_015863431.1	3722	3904	-	182
NW_015863431.1	3715	3916	-	201
NW_015863832.1	12407	12485	+	78
NW_015863845.1	10759	10879	-	120
NW_015864653.1	26866	26951	-	85
NW_015864951.1	78742	79019	+	277
NW_015865063.1	16476	16897	-	421
NW_015865123.1	22283	22486	+	203
NW_015865279.1	25013	25083	+	70
NW_015865392.1	5093	5188	+	95
NW_015866444.1	3619	3718	+	99
NW_015866503.1	49959	50046	-	87
NW_015866503.1	49963	50056	-	93
NW_015866547.1	5393	5577	+	184
NW_015867097.1	2911	3293	-	382
NW_015868090.1	89724	89831	+	107
NW_015868569.1	22113	22394	-	281
NW_015868612.1	225700	225900	-	200
NW_015868935.1	21565	21800	+	235
NW_015869104.1	10437	10511	-	74
NW_015869579.1	19092	19251	+	159

NW_015870078.1	22461	22501	-	40
NW_015870422.1	23566	23701	+	135
NW_015871407.1	2714	2784	+	70
NW_015872355.1	11686	11867	+	122
NW_015872389.1	91410	91464	+	54
NW_015872823.1	67042	67144	+	102
NW_015873401.1	2237	2257	-	20
NW_015873620.1	64931	64998	+	67
NW_015874034.1	25088	25373	-	285
NW_015874183.1	8954	9006	+	52
NW_015874690.1	28379	28532	+	153
NW_015874746.1	23028	23237	+	209
NW_015874746.1	23015	23208	+	193
NW_015875320.1	8169	8336	+	167
NW_015876040.1	4699	4765	-	66
NW_015876070.1	96173	96289	+	116
NW_015876070.1	96155	96281	+	126
NW_015876584.1	38014	38109	-	95
NW_015876939.1	25817	25851	-	34
NW_015877093.1	13134	13204	-	70
NW_015877093.1	13133	13190	-	57
NW_015877540.1	8928	9123	-	195
NW_015877742.1	53121	53200	-	79
NW_015878853.1	15304	15472	-	168
NW_015879146.1	65518	65563	-	45

NW_015879194.1	12891	13062	+	171
NW_015879256.1	20696	20769	-	73
NW_015880231.1	12792	12873	+	81
NW_015880571.1	64313	64395	-	82
NW_015880571.1	64332	64387	-	55
NW_015880571.1	64304	64403	-	99
NW_015880575.1	5186	5254	-	68
NW_015881441.1	33150	33254	-	104
NW_015882231.1	79125	79244	-	99
NW_015882580.1	1236	1331	+	95
NW_015882881.1	13595	14048	+	453
NW_015883513.1	41200	41331	+	131
NW_015883564.1	1635	1712	-	77
NW_015885378.1	44974	45112	-	138
NW_015885671.1	12998	13119	+	121
NW_015885681.1	3902	3961	+	59
NW_015885756.1	5918	6019	+	101
NW_015885903.1	49683	50104	-	421
NW_015885903.1	49779	50112	-	333
NW_015886073.1	21916	22102	+	186
NW_015886247.1	31512	31583	+	71
NW_015886303.1	78615	78759	+	144
NW_015886303.1	78638	78778	+	140
NW_015886487.1	43812	43907	+	95
NW_015886591.1	14751	14841	+	90

NW_015886706.1	67936	68060	+	124
NW_015886753.1	53496	53616	-	120
NW_015886799.1	108748	109074	+	326
NW_015886799.1	109295	109375	+	80
NW_015886799.1	108769	109053	+	284
NW_015886799.1	108726	109386	+	660
NW_015886840.1	21885	22026	-	141
NW_015886864.1	92494	92521	-	27
NW_015886924.1	21567	21713	+	146
NW_015886977.1	192290	192354	+	64
NW_015886977.1	192240	192381	+	141
NW_015887022.1	26444	26567	-	123
NW_015887038.1	57755	57894	+	139
NW_015887170.1	113885	114005	+	120
NW_015887195.1	13072	13224	-	152
NW_015887409.1	50765	50952	-	187
NW_015887443.1	65475	65611	+	136
NW_015887443.1	65469	65573	+	104
NW_015887579.1	81438	81568	-	130
NW_015887614.1	27599	27749	+	150
NW_015887669.1	49049	49134	-	85
NW_015887883.1	35995	36181	-	186
NW_015887941.1	23175	23341	-	166
NW_015888111.1	63642	63751	+	109
NW_015888166.1	52084	52400	-	262

NW_015888217.1	15127	15234	+	107
NW_015888217.1	15148	15226	+	78
NW_015888217.1	15129	15245	+	116
NW_015888243.1	16084	16405	-	321
NW_015888274.1	105873	106094	-	221
NW_015888321.1	91732	91953	-	221
NW_015888340.1	202288	202488	-	200
NW_015888409.1	13259	13386	-	127
NW_015888421.1	7876	7982	-	79
NW_015888801.1	52572	52708	+	136
NW_015888830.1	72942	73002	-	60
NW_015888852.1	66390	66442	-	52
NW_015889258.1	68225	68322	-	97
NW_015889258.1	68223	68376	-	153
NW_015889282.1	18495	18592	+	97
NW_015889319.1	22971	23151	-	180
NW_015889470.1	21740	21769	-	29
NW_015889470.1	21720	21800	-	80
NW_015889609.1	3860	3957	-	97
NW_015889778.1	20810	20846	+	36
NW_015890168.1	14825	15011	-	186
NW_015890226.1	133964	134018	+	54
NW_015890245.1	63207	63239	+	32
NW_015890831.1	24058	24160	-	102
NW_015890831.1	24041	24156	-	115

NW_015890836.1	139823	139861	-	38
NW_015890885.1	21952	22036	-	84
NW_015890885.1	21736	22120	-	384
NW_015890995.1	115843	116017	+	174
NW_015891040.1	7273	7428	-	155
NW_015891040.1	7268	7444	-	176
NW_015891048.1	24943	24987	+	44
NW_015891100.1	52229	52398	+	169
NW_015891100.1	52216	52382	+	166
NW_015891143.1	21366	21433	-	67
NW_015891174.1	8201	8576	-	375
NW_015891230.1	41930	42066	-	136
NW_015891356.1	13631	14161	+	530
NW_015891485.1	8392	8578	-	186
NW_015891485.1	8197	8576	-	379
NW_015891490.1	34954	34998	-	44
NW_015892080.1	48682	48732	-	50
NW_015892123.1	27442	27571	-	129
NW_015892254.1	26978	27146	-	168
NW_015892301.1	34948	35014	+	66
NW_015892301.1	34914	34978	+	64
NW_015892304.1	31591	31741	+	150
NW_015892309.1	96057	96216	+	159
NW_015892338.1	14029	14273	+	244
NW_015893017.1	889	1038	+	94

NW_015894176.1	6578	7119	-	541
NW_015894176.1	6555	7109	-	554
NW_015894176.1	6560	6963	-	403
NW_015894860.1	41609	41713	+	104
NW_015894860.1	41602	41758	+	156
NW_015895077.1	32110	32232	-	122
NW_015895787.1	62529	62821	+	292
NW_015895787.1	62528	62876	+	348
NW_015897470.1	81	334	-	253
NW_015897942.1	2315	2405	-	90
NW_015897951.1	2922	3058	-	136
NW_015898292.1	2315	2389	-	74
NW_015898598.1	53682	53819	+	137
NW_015898709.1	2802	3051	+	249
NW_015898765.1	1362	1461	+	99
NW_015898851.1	5287	5382	-	95
NW_015898864.1	5129	5304	+	175
NW_015898921.1	3683	3795	+	112
NW_015898921.1	3648	4124	+	476
NW_015898947.1	6682	6743	+	61
NW_015899018.1	2773	2937	-	164
NW_015899138.1	84248	84360	-	112
NW_015899228.1	12688	12806	-	118
NW_015899435.1	51251	51309	-	58
NW_015899449.1	43022	43328	-	160

NW_015899502.1	40631	40658	+	27
NW_015899512.1	8248	8420	+	172
NW_015899706.1	116684	116734	+	50
NW_015899866.1	87516	87565	+	49
NW_015899919.1	26440	26651	+	211
NW_015900112.1	27561	27666	+	105
NW_015900112.1	27536	27681	+	145
NW_015900220.1	114092	114225	-	133
NW_015900322.1	20858	20988	+	130
NW_015900396.1	42341	42494	+	153
NW_015900939.1	24202	24554	+	352
NW_015900939.1	24183	24561	+	378
NW_015901023.1	86630	86703	-	73
NW_015901026.1	129014	129096	-	82
NW_015901029.1	7779	7826	-	47
NW_015901353.1	58584	58631	+	47
NW_015901493.1	12459	12565	+	106
NW_015901529.1	31324	31386	+	62
NW_015901537.1	22813	23041	+	228
NW_015901537.1	22829	23053	+	224
NW_015901537.1	23832	23940	+	108
NW_015901537.1	22809	23064	+	255
NW_015901537.1	23861	23948	+	87
NW_015901544.1	16510	16553	-	43
NW_015901544.1	15884	15959	-	75

NW_015901544.1	16495	16580	-	85
NW_015901713.1	132394	132487	+	93
NW_015901713.1	132386	132527	+	141
NW_015901739.1	38061	38253	+	192
NW_015901757.1	44846	44963	-	117
NW_015901864.1	107835	107966	+	131
NW_015902033.1	63075	63157	+	82
NW_015902239.1	165221	165244	+	23
NW_015902493.1	112455	112636	-	181
NW_015902628.1	19757	19916	-	159
NW_015902633.1	20094	20153	-	59
NW_015902639.1	1681	1826	+	145
NW_015902639.1	1671	1869	+	198
NW_015902872.1	21871	21971	+	100
NW_015902993.1	21641	21653	+	12
NW_015902993.1	21553	21639	+	86
NW_015903020.1	85670	85790	-	120
NW_015903041.1	59446	59559	+	113
NW_015903099.1	32654	32685	-	31
NW_015903247.1	30917	31042	+	125
NW_015903380.1	10229	10307	+	78
NW_015903469.1	37914	37951	-	37
NW_015903495.1	57647	57705	+	58
NW_015903630.1	28741	28863	-	122
NW_015903939.1	19203	19238	-	35

NW_015903939.1	19208	19365	-	157
NW_015904002.1	45411	45539	+	128
NW_015904002.1	45423	45569	+	146
NW_015904042.1	2102	2182	+	80
NW_015904042.1	2103	2167	+	64
NW_015904042.1	2090	2174	+	84
NW_015904174.1	68386	68602	+	179
NW_015904174.1	68392	68564	+	141
NW_015904287.1	62477	62532	+	55
NW_015904325.1	2308	2332	+	24
NW_015904370.1	55390	55438	-	48
NW_015904417.1	49684	49876	-	192
NW_015904417.1	49683	49885	-	202
NW_015904609.1	54975	55094	-	119
NW_015904756.1	40483	40614	-	131
NW_015904756.1	40480	40625	-	145
NW_015904798.1	59582	59648	-	66
NW_015905140.1	28283	28342	+	59
NW_015905275.1	31192	31353	-	161
NW_015905309.1	65482	65591	-	109
NW_015905476.1	48807	48902	+	95
NW_015905576.1	11518	11625	+	107
NW_015905576.1	11514	11622	+	108
NW_015905674.1	23646	23694	-	48
NW_015905901.1	16879	16953	+	74

NW_015905909.1	27509	27582	+	66
NW_015905941.1	41161	41283	-	122
NW_015905951.1	17298	17499	-	201
NW_015905982.1	24035	24137	-	102
NW_015906141.1	53377	53555	-	178
NW_015906190.1	107560	107775	-	215
NW_015906226.1	19659	19709	-	50
NW_015906394.1	39223	39308	-	85
NW_015906418.1	166336	166481	+	145
NW_015906582.1	20130	20284	-	154
NW_015907125.1	77779	77874	+	95
NW_015907225.1	21961	22048	+	87
NW_015907486.1	28715	28756	+	41
NW_015907489.1	26868	26920	+	52
NW_015907538.1	125479	125535	-	56
NW_015907803.1	102834	103069	-	235
NW_015908015.1	23952	24103	-	151
NW_015908254.1	48048	48185	-	137
NW_015908254.1	48047	48238	-	191
NW_015908424.1	12391	12475	-	84
NW_015908424.1	12388	12478	-	90
NW_015908598.1	45473	45539	+	66
NW_015908643.1	119759	119805	-	46
NW_015908995.1	240997	241172	+	175
NW_015908995.1	240971	241166	+	195

NW_015909023.1	87472	87533	-	61
NW_015909339.1	6137	6214	+	77
NW_015909339.1	6147	6291	+	144
NW_015909421.1	7977	8064	+	87
NW_015909524.1	19302	19349	-	47
NW_015909533.1	32164	32711	+	547
NW_015909562.1	21380	21431	+	51
NW_015909610.1	8896	9030	-	134
NW_015909765.1	15993	16036	-	43
NW_015909843.1	1132	1276	-	144
NW_015909951.1	32055	32083	-	28
NW_015910200.1	26233	26319	+	86
NW_015910226.1	177873	177897	+	24
NW_015910263.1	61632	61789	-	157
NW_015910429.1	131199	131266	+	67
NW_015910523.1	44068	44195	-	127
NW_015910523.1	44070	44186	-	116
NW_015910523.1	44072	44206	-	134
NW_015910734.1	80928	80973	+	45
NW_015910735.1	80613	80673	-	60
NW_015910764.1	71058	71213	+	155
NW_015910843.1	31790	31951	+	90
NW_015910859.1	3471	3537	-	66
NW_015911182.1	15543	15698	+	155
NW_015911274.1	11365	11439	-	74

NW_015911274.1	10954	11177	-	223
NW_015911274.1	11371	11508	-	137
NW_015911510.1	53474	53540	+	66
NW_015911612.1	22514	22633	+	119
NW_015911652.1	40777	40872	-	95
NW_015912020.1	35260	35384	-	124
NW_015912040.1	11034	11118	+	84
NW_015912040.1	11027	11182	+	155
NW_015912118.1	44616	44879	+	263
NW_015912118.1	44617	44753	+	136
NW_015912118.1	44621	44908	+	287
NW_015912131.1	28149	28512	+	363
NW_015912190.1	177697	177803	+	106
NW_015912199.1	8717	8752	+	35
NW_015912254.1	43396	43487	+	91
NW_015912254.1	43395	43502	+	107
NW_015912254.1	43388	43545	+	157
NW_015912288.1	55937	56053	-	116
NW_015912288.1	55940	56064	-	124
NW_015912288.1	55948	56095	-	147
NW_015912352.1	8793	8987	-	194
NW_015912481.1	39942	40069	+	127
NW_015912543.1	17792	18021	+	210
NW_015912609.1	52241	52470	+	229
NW_015912648.1	69939	70074	+	135

NW_015912807.1	17523	17635	-	112
NW_015912835.1	16767	16806	+	39
NW_015912920.1	36610	36738	+	128
NW_015912962.1	133246	133338	+	92
NW_015912962.1	133248	133344	+	96
NW_015913151.1	17743	17833	+	90
NW_015913157.1	35451	35561	-	110
NW_015913157.1	35438	35569	-	131
NW_015913254.1	115591	115618	-	27
NW_015913275.1	9105	9203	+	98
NW_015913329.1	11332	11414	-	82
NW_015913329.1	21065	21137	+	72
NW_015913538.1	22845	22996	-	151
NW_015913653.1	43257	43535	-	278
NW_015913653.1	43244	43538	-	294
NW_015913653.1	43286	43528	-	242
NW_015913743.1	145366	145469	+	103
NW_015913756.1	96524	96646	-	122
NW_015913757.1	19627	19742	+	115
NW_015913954.1	19943	20112	-	169
NW_015914026.1	113100	113304	-	204
NW_015914026.1	113043	113290	-	247
NW_015914026.1	113007	113322	-	315
NW_015914227.1	7873	7962	+	89
NW_015914227.1	7740	7998	+	258

NW_015914252.1	47178	47260	+	82
NW_015914718.1	1369	1475	+	106
NW_015914776.1	21717	21743	+	26
NW_015914889.1	19269	19350	-	81
NW_015914929.1	52097	52153	-	56
NW_015914942.1	36965	37095	-	130
NW_015915700.1	19617	19707	+	90
NW_015915768.1	5137	5281	+	144
NW_015915768.1	5134	5286	+	152
NW_015916189.1	66348	66391	-	43
NW_015916429.1	14212	14375	-	163
NW_015916474.1	11099	11465	-	281
NW_015916479.1	46498	46642	+	144
NW_015916495.1	25467	25571	-	104
NW_015916509.1	27250	27483	-	233
NW_015916540.1	3920	4136	-	156
NW_015916741.1	1040	1197	+	157
NW_015917104.1	106651	107007	+	356
NW_015917182.1	35174	35365	+	191
NW_015917256.1	50037	50220	-	183
NW_015917572.1	11610	11649	+	39
NW_015917701.1	15354	15586	-	232
NW_015917758.1	48755	48807	+	52
NW_015917816.1	24205	24244	-	39
NW_015917816.1	24147	24257	-	110

NW_015918107.1	9529	9571	-	42
NW_015918243.1	43212	43315	-	103
NW_015918253.1	198550	198641	-	91
NW_015918282.1	38903	38996	+	93
NW_015918282.1	38904	38985	+	81
NW_015918282.1	38894	38994	+	100
NW_015918332.1	43689	43740	+	51
NW_015918332.1	43649	43761	+	112
NW_015918482.1	25983	26081	-	98
NW_015918519.1	32893	33106	+	147
NW_015918578.1	37359	37397	-	38
NW_015918582.1	10953	11025	+	72
NW_015918638.1	14765	14864	-	99
NW_015919026.1	38400	38455	+	55
NW_015919026.1	38403	38451	+	48
NW_015919290.1	4493	4585	+	92
NW_015919290.1	4488	4582	+	94
NW_015919343.1	13608	13670	+	62
NW_015919343.1	13616	13699	+	83
NW_015920008.1	133614	133731	-	117
NW_015920281.1	88469	88506	-	37
NW_015920380.1	17525	17568	-	43
NW_015920380.1	73579	73614	+	35
NW_015920380.1	17522	17566	-	44
NW_015921032.1	11372	11669	-	297

NW_015921235.1	34958	35133	-	175
NW_015921366.1	24479	24540	-	61
NW_015921366.1	24493	24547	-	54
NW_015921424.1	43635	43734	-	99
NW_015921424.1	43420	43775	-	355
NW_015921455.1	47023	47127	+	104
NW_015921513.1	116006	116078	-	72
NW_015921570.1	23828	23939	+	111
NW_015921570.1	23822	24022	+	200
NW_015921668.1	51981	52173	+	192
NW_015921961.1	5731	5826	-	95
NW_015922475.1	25508	25589	-	81
NW_015922501.1	93774	93899	-	125
NW_015922526.1	25070	25154	+	84
NW_015922526.1	25060	25160	+	100
NW_015923011.1	26015	26114	-	99
NW_015923088.1	24184	24276	-	92
NW_015923102.1	77059	77166	+	107
NW_015923157.1	42896	43012	+	116
NW_015923197.1	48414	48510	-	96
NW_015923366.1	3449	3764	+	315
NW_015923440.1	26029	26071	+	42
NW_015923540.1	20780	20802	+	22
NW_015923842.1	59515	59671	-	156
NW_015923961.1	60883	61029	-	146

NW_015923998.1	11665	11856	-	191
NW_015924026.1	4953	5096	-	143
NW_015924362.1	57403	57492	+	89
NW_015924769.1	28994	29092	-	98
NW_015924769.1	28993	29102	-	109
NW_015924769.1	28985	29120	-	135
NW_015924783.1	18785	18920	-	135
NW_015924813.1	67623	67655	-	32
NW_015924977.1	164816	164877	-	61
NW_015924996.1	143709	143783	+	74
NW_015925225.1	23164	23272	-	56
NW_015925339.1	112990	113092	+	102
NW_015925570.1	88111	88602	+	491
NW_015925580.1	25352	25517	-	165
NW_015925665.1	13038	13239	+	201
NW_015925723.1	61578	61673	-	95
NW_015925781.1	14286	14347	+	61
NW_015925838.1	24834	24913	-	79
NW_015926003.1	106484	106599	+	115
NW_015926130.1	104340	104413	-	73
NW_015926331.1	2298	2461	-	163
NW_015926445.1	6070	6237	+	167
NW_015926476.1	74284	74531	+	225
NW_015926538.1	83552	83608	+	56
NW_015926586.1	94133	94265	-	132

NW_015926586.1	94129	94275	-	146
NW_015926652.1	19343	19533	-	190
NW_015926811.1	73386	73497	+	111
NW_015926811.1	73373	73517	+	144
NW_015926811.1	73350	73529	+	179
NW_015926811.1	81903	82029	-	94
NW_015926815.1	51323	51458	-	135
NW_015926839.1	223303	223350	-	47
NW_015926839.1	120252	120369	-	117
NW_015926845.1	6112	6169	-	57
NW_015926845.1	5880	6234	-	354
NW_015926872.1	113116	113268	+	131
NW_015927376.1	42850	43085	+	235
NW_015927878.1	4860	5049	-	189
NW_015927926.1	302913	303022	-	109
NW_015928315.1	104353	104440	+	87
NW_015928315.1	104336	104446	+	110
NW_015928450.1	27338	27554	-	216
NW_015928664.1	2154	2215	+	61
NW_015928664.1	2091	2224	+	133
NW_015928664.1	2157	2221	+	64
NW_015928919.1	18973	19070	-	97
NW_015929368.1	34365	34466	-	101
NW_015929535.1	13784	13927	+	143
NW_015929535.1	13792	13924	+	132

NW_015929565.1	11758	11879	-	121
NW_015929565.1	11730	11904	-	174
NW_015929842.1	29108	29365	-	257
NW_015929893.1	29965	30164	-	199
NW_015929922.1	29386	29608	-	222
NW_015930022.1	108325	108722	+	397
NW_015930022.1	108331	108734	+	403
NW_015930366.1	2048	2222	+	174
NW_015930723.1	29299	29317	+	18
NW_015930723.1	29261	29316	+	55
NW_015930799.1	19876	19963	+	87
NW_015930843.1	61584	61696	+	112
NW_015930915.1	20505	20577	+	72
NW_015930988.1	8788	8909	-	121
NW_015931076.1	57352	57433	+	81
NW_015931361.1	58453	58490	+	37
NW_015931444.1	22654	22881	-	227
NW_015931806.1	16322	16413	-	91
NW_015932044.1	66827	66984	-	111
NW_015932214.1	8003	8109	+	38
NW_015932470.1	94411	94545	-	134
NW_015932752.1	1976	2037	-	61
NW_015932884.1	58662	58762	+	100
NW_015932994.1	119831	119905	+	74
NW_015933045.1	2622	2748	-	126

NW_015933085.1	3378	3589	-	211
NW_015933212.1	46995	47022	-	27
NW_015933265.1	9628	9740	+	112
NW_015933278.1	43692	43766	-	39
NW_015934100.1	104199	104271	-	72
NW_015934124.1	104944	105120	-	176
NW_015934159.1	83740	83797	-	57
NW_015934159.1	83747	83803	-	56
NW_015934476.1	54630	54797	-	167
NW_015934627.1	4162	4312	+	150
NW_015934718.1	13723	13810	-	22
NW_015934762.1	153538	153605	-	67
NW_015934837.1	38878	38955	-	77
NW_015935386.1	123422	123542	+	120
NW_015935397.1	14189	14486	+	297
NW_015935397.1	14207	14488	+	281
NW_015935397.1	14169	14494	+	325
NW_015935628.1	37952	38262	+	310
NW_015935628.1	37953	38152	+	199
NW_015935628.1	37954	38397	+	443
NW_015935834.1	42612	42634	-	22
NW_015935841.1	69338	69492	-	102
NW_015936229.1	33611	33751	-	140
NW_015936350.1	7529	7748	-	219
NW_015936350.1	7449	7765	-	316

NW_015937094.1	17731	17787	+	56
NW_015937423.1	10997	11242	-	245
NW_015937468.1	48603	48814	-	211
NW_015937468.1	48614	48868	-	254
NW_015937976.1	48917	49108	+	106
NW_015938092.1	30090	30283	+	193
NW_015938192.1	52490	52546	+	56
NW_015938613.1	11047	11209	-	162
NW_015938844.1	23765	23831	+	66
NW_015938902.1	15450	15532	+	82
NW_015938902.1	15408	15546	+	138
NW_015939157.1	95682	95753	+	71
NW_015939157.1	95679	95785	+	106
NW_015939165.1	7130	7226	-	96
NW_015939165.1	7133	7218	-	85
NW_015939169.1	17223	17318	-	95
NW_015939169.1	17179	17341	-	162
NW_015939409.1	17416	17577	+	161
NW_015939606.1	115318	115467	-	149
NW_015939894.1	2345	2400	-	55
NW_015939970.1	93481	93564	+	83
NW_015939977.1	14724	14784	+	60
NW_015940213.1	34116	34308	+	192
NW_015940213.1	34089	34330	+	241
NW_015940213.1	34065	34351	+	286

NW_015940941.1	18527	18586	-	59
NW_015941031.1	9358	9498	-	140
NW_015941547.1	8362	8613	-	251
NW_015941661.1	93266	93541	+	164
NW_015941727.1	6392	6422	+	30
NW_015942054.1	20216	20265	-	49
NW_015942054.1	20218	20267	-	49
NW_015942212.1	28098	28166	-	68
NW_015942305.1	15216	15275	+	59
NW_015942306.1	2756	2980	-	224
NW_015942336.1	38773	38886	-	113
NW_015942389.1	2166	2297	-	131
NW_015942586.1	39612	39703	-	91
NW_015942586.1	39626	39716	-	90
NW_015942689.1	94494	94629	+	135
NW_015942703.1	17084	17264	-	166
NW_015942792.1	12249	12332	-	83
NW_015942822.1	9307	9447	-	140
NW_015942822.1	9312	9437	-	125
NW_015942831.1	17591	17700	-	109
NW_015942937.1	72529	72616	+	87
NW_015942937.1	72516	72604	+	88
NW_015943152.1	57294	57363	-	65
NW_015943297.1	14599	14687	-	88
NW_015943297.1	14523	14649	-	126

NW_015943297.1	14516	14690	-	174
NW_015943817.1	28091	28257	+	88
NW_015943827.1	87379	87563	-	184
NW_015943849.1	9572	9669	+	97
NW_015943920.1	20322	20427	-	105
NW_015943920.1	20302	20453	-	151
NW_015943952.1	4713	4822	+	109
NW_015944005.1	18284	18434	-	150
NW_015944040.1	55261	55411	+	150
NW_015944428.1	24752	24907	+	155
NW_015944541.1	1143	1288	-	17
NW_015944541.1	1187	1282	-	11
NW_015944541.1	1148	1286	-	15
NW_015944606.1	66431	66515	-	84
NW_015944696.1	123689	123811	-	15
NW_015944732.1	67959	68074	+	115
NW_015944834.1	30463	30603	-	140
NW_015945047.1	17096	17215	-	119
NW_015945148.1	35648	35800	-	140
NW_015945235.1	20244	20313	-	69
NW_015945258.1	26557	26574	-	17
NW_015945258.1	26484	26562	-	78
NW_015945258.1	26349	26616	-	267
NW_015945275.1	18737	18773	-	36
NW_015945275.1	19116	19280	-	164

NW_015945275.1	18714	18790	-	76
NW_015945275.1	19104	19277	-	173
NW_015945287.1	19269	19350	+	81
NW_015945368.1	20441	20529	+	88
NW_015945368.1	20373	20516	+	143
NW_015945478.1	6192	6421	-	229
NW_015945658.1	70842	70960	+	118
NW_015945791.1	36327	36414	-	87
NW_015945791.1	36083	36526	-	443
NW_015945972.1	24490	24591	+	101
NW_015946040.1	202660	202726	+	66
NW_015946040.1	69037	69118	-	81
NW_015946639.1	20189	20298	-	109
NW_015946639.1	20191	20284	-	93
NW_015946945.1	11824	11992	-	168
NW_015946956.1	108900	109074	+	174
NW_015946985.1	28323	28428	+	105
NW_015947005.1	14950	15155	+	205
NW_015947005.1	14895	15151	+	256
NW_015947390.1	70179	70328	-	149
NW_015947690.1	25489	25591	+	102
NW_015947780.1	9888	9990	+	102
NW_015947943.1	6808	6853	-	45
NW_015948125.1	13739	13885	+	146
NW_015948286.1	18059	18130	-	71

NW_015948513.1	3155	3206	+	51
NW_015948585.1	50154	50238	+	84
NW_015948766.1	55866	56021	+	155
NW_015948940.1	33195	33357	-	162
NW_015948940.1	33197	33344	-	147
NW_015948940.1	33175	33385	-	210
NW_015949511.1	37104	37307	+	203
NW_015949542.1	28713	29065	-	352
NW_015949542.1	28642	29085	-	443
NW_015949558.1	3480	3684	+	204
NW_015949558.1	3492	3641	+	149
NW_015949601.1	25627	25709	-	82
NW_015949806.1	31806	32009	-	203
NW_015949825.1	79861	79944	+	60
NW_015950147.1	8871	8942	-	71
NW_015950258.1	98905	99019	+	114
NW_015950258.1	81286	81328	+	42
NW_015950258.1	99515	99551	+	36
NW_015950258.1	81262	81345	+	83
NW_015950258.1	84579	84655	+	76
NW_015950258.1	98878	99066	+	188
NW_015950258.1	99495	99574	+	79
NW_015951046.1	25334	25435	+	101
NW_015951453.1	22852	23035	+	183
NW_015951599.1	21408	21461	-	53

NW_015951811.1	23701	23731	+	30
NW_015951896.1	70229	70385	-	156
NW_015951974.1	17288	17499	-	211
NW_015951974.1	17291	17507	-	216
NW_015952009.1	16295	16427	-	129
NW_015952024.1	22213	22336	-	123
NW_015952120.1	32556	32743	+	187
NW_015952120.1	32555	32744	+	189
NW_015952120.1	32551	32728	+	177
NW_015952413.1	50153	50285	+	132
NW_015953192.1	34534	34653	+	119
NW_015953192.1	34525	34796	+	271
NW_015953320.1	12687	12905	+	218
NW_015953492.1	12710	12805	+	95
NW_015953723.1	51646	51713	-	67
NW_015953731.1	23514	23535	-	21
NW_015954099.1	8516	8700	+	184
NW_015954191.1	16142	16251	-	109
NW_015954347.1	44995	45189	-	194
NW_015954473.1	21967	22083	-	116
NW_015954610.1	2204	2292	+	88
NW_015954636.1	17276	17425	-	149
NW_015954636.1	17278	17417	-	139
NW_015954636.1	17264	17423	-	159
NW_015955103.1	60689	60850	+	161

NW_015955427.1	12295	12404	-	109
NW_015955427.1	12832	12983	-	151

13days post inoculation (Hypo-DMRs)

Scaffold	Start	End	Region stain	Overlapped site
NW_015787320.1	85108	85195	-	87
NW_015787321.1	11238	11344	-	106
NW_015787326.1	23935	24158	+	223
NW_015787340.1	4198	4299	+	101
NW_015787340.1	4177	4284	+	107
NW_015787444.1	10343	10384	+	41
NW_015787444.1	27510	27713	-	203
NW_015787463.1	21997	22103	-	106
NW_015787466.1	19628	19765	-	137
NW_015787503.1	4474	4587	+	113
NW_015787806.1	42609	42961	+	352
NW_015788158.1	8921	9169	-	248
NW_015788158.1	8925	9164	-	239
NW_015788210.1	10965	11088	-	73
NW_015788314.1	21883	22015	-	132
NW_015788317.1	27663	27697	-	34
NW_015788528.1	12621	12655	-	34
NW_015788570.1	63332	63474	-	142
NW_015788608.1	29233	29384	-	151

NW_015788661.1	33763	33966	-	203
NW_015788678.1	10425	10600	+	175
NW_015788757.1	16421	16526	+	105
NW_015788759.1	24700	24779	+	79
NW_015788908.1	33041	33107	+	66
NW_015788953.1	21718	21834	+	116
NW_015789051.1	27947	28023	-	76
NW_015789100.1	45327	45369	-	42
NW_015789314.1	46573	46738	+	165
NW_015789314.1	13362	13502	-	130
NW_015789314.1	46566	46906	+	253
NW_015789404.1	3180	3271	+	91
NW_015789405.1	4782	4863	-	81
NW_015789405.1	4656	4870	-	214
NW_015789559.1	85955	86037	-	82
NW_015789620.1	81358	81447	-	89
NW_015789683.1	133514	133683	+	169
NW_015789752.1	27853	27875	-	22
NW_015789854.1	35792	35941	+	149
NW_015789913.1	41478	41805	-	327
NW_015789913.1	41454	41679	-	225
NW_015790036.1	14741	14848	-	107
NW_015790061.1	37223	37369	-	146
NW_015790118.1	88406	88574	+	168
NW_015790311.1	50641	50789	-	148

NW_015790413.1	7538	7707	+	169
NW_015790534.1	7418	7462	+	44
NW_015790638.1	3265	3383	+	118
NW_015790718.1	10456	10478	+	22
NW_015791028.1	26822	27056	+	132
NW_015791165.1	67355	67507	-	152
NW_015791165.1	14272	14323	-	51
NW_015791242.1	72536	72688	+	152
NW_015791267.1	88673	88726	+	53
NW_015791267.1	88662	88739	+	77
NW_015791293.1	53709	53783	-	74
NW_015791444.1	14965	15038	+	73
NW_015791444.1	14919	15043	+	124
NW_015791453.1	128908	129034	-	93
NW_015791525.1	27800	27939	-	139
NW_015791560.1	4344	4459	+	102
NW_015791672.1	6056	6174	-	118
NW_015791925.1	51043	51093	+	50
NW_015791955.1	18293	18404	+	111
NW_015792055.1	122843	122956	-	113
NW_015792220.1	36371	36419	-	48
NW_015792301.1	8250	8439	+	189
NW_015792376.1	37058	37374	-	316
NW_015792437.1	21256	21394	-	114
NW_015792483.1	37781	38238	+	457

NW_015792507.1	27504	27723	+	219
NW_015792520.1	11230	11291	+	61
NW_015792589.1	8562	8879	+	317
NW_015792642.1	36224	36402	+	178
NW_015792646.1	17803	17849	-	46
NW_015792682.1	42768	43000	+	232
NW_015793041.1	65257	65331	-	74
NW_015793234.1	7948	8048	-	100
NW_015793360.1	21822	21942	-	120
NW_015793434.1	11970	12023	+	53
NW_015793434.1	11974	12014	+	40
NW_015793434.1	11966	12009	+	43
NW_015793460.1	50217	50264	-	47
NW_015793485.1	22303	22380	-	77
NW_015793546.1	25549	25707	-	158
NW_015793573.1	73483	73651	+	168
NW_015793609.1	41023	41059	-	36
NW_015793615.1	17569	17700	-	131
NW_015793638.1	7460	7593	-	133
NW_015793650.1	39149	39200	-	51
NW_015793782.1	5413	5504	-	91
NW_015793792.1	33249	33375	+	126
NW_015793793.1	36248	36341	+	93
NW_015793793.1	36235	36336	+	101
NW_015793813.1	8199	8314	+	115

NW_015793863.1	128588	128716	+	128
NW_015793978.1	13186	13231	-	45
NW_015794132.1	171072	171126	-	54
NW_015794132.1	171041	171148	-	107
NW_015794132.1	199298	199565	-	267
NW_015794193.1	43111	43213	+	102
NW_015794196.1	5734	5810	+	76
NW_015794278.1	13681	13763	-	82
NW_015794460.1	11719	11821	-	102
NW_015794475.1	149402	149474	-	47
NW_015794503.1	61901	61940	+	39
NW_015794503.1	61912	61962	+	50
NW_015794511.1	38160	38247	+	87
NW_015794640.1	35718	35910	+	192
NW_015794785.1	71552	71653	-	101
NW_015794811.1	30811	30905	+	94
NW_015794883.1	7321	7493	+	172
NW_015795024.1	19936	19998	+	62
NW_015795175.1	144913	144953	+	40
NW_015795225.1	7134	7164	+	30
NW_015795237.1	12195	12240	-	45
NW_015795290.1	87917	87997	-	80
NW_015795290.1	50549	50733	+	184
NW_015795308.1	12310	12435	-	125
NW_015795428.1	8148	8207	-	59

NW_015795498.1	40213	40287	+	74
NW_015795498.1	41263	41487	+	224
NW_015795512.1	97351	97452	+	101
NW_015795689.1	6049	6156	+	57
NW_015795724.1	17595	17723	-	128
NW_015795761.1	135477	135567	+	90
NW_015795761.1	139200	139277	-	77
NW_015795960.1	120073	120531	-	458
NW_015796009.1	5456	5513	-	57
NW_015796104.1	46042	46309	+	99
NW_015796104.1	46037	46326	+	116
NW_015796123.1	22497	22690	+	193
NW_015796173.1	5158	5262	+	104
NW_015796185.1	26412	26529	+	117
NW_015796366.1	77817	77864	+	47
NW_015796367.1	73800	73879	-	79
NW_015796367.1	14532	14824	+	292
NW_015796411.1	19827	19924	-	97
NW_015796504.1	33057	33205	+	94
NW_015796526.1	59054	59218	+	164
NW_015796699.1	19417	19500	+	83
NW_015796699.1	7719	7862	-	143
NW_015796735.1	12611	12822	+	211
NW_015796770.1	12769	12885	+	116
NW_015796835.1	2827	2953	+	126

NW_015796928.1	59648	59782	+	104
NW_015796940.1	16596	16645	-	49
NW_015796940.1	5542	5722	+	129
NW_015797034.1	41231	41294	-	63
NW_015797040.1	18008	18168	+	160
NW_015797157.1	72138	72178	-	40
NW_015797183.1	106299	106526	-	227
NW_015797349.1	26982	27146	-	164
NW_015797403.1	4563	4654	-	91
NW_015797403.1	4586	4678	-	92
NW_015797438.1	1735	1853	+	118
NW_015797568.1	46286	46402	+	116
NW_015797568.1	47710	47884	+	113
NW_015797875.1	10945	11014	-	69
NW_015797936.1	16742	16874	+	132
NW_015797950.1	21559	21588	+	29
NW_015798067.1	31032	31199	-	104
NW_015798073.1	37926	38003	-	77
NW_015798165.1	12728	12939	+	211
NW_015798270.1	10993	11041	+	48
NW_015798310.1	11825	11856	-	31
NW_015798355.1	5681	5776	+	95
NW_015798375.1	28690	28745	+	55
NW_015798380.1	35260	35390	+	130
NW_015798388.1	28721	28732	-	11

NW_015798388.1	28690	28795	-	105
NW_015798390.1	31627	31696	-	69
NW_015798390.1	77097	77190	-	93
NW_015798507.1	143625	143762	+	137
NW_015798528.1	18426	18507	-	81
NW_015798528.1	18468	18651	-	183
NW_015798719.1	723	814	+	91
NW_015798786.1	21265	21347	+	82
NW_015798807.1	13443	13527	-	84
NW_015798807.1	13434	13535	-	101
NW_015798826.1	24894	24969	-	75
NW_015798863.1	11065	11239	+	103
NW_015798877.1	6861	7148	-	287
NW_015798886.1	13443	13573	-	130
NW_015798967.1	19141	19208	-	67
NW_015798989.1	6459	6578	+	119
NW_015799019.1	5512	5750	+	238
NW_015799036.1	78604	78695	+	91
NW_015799115.1	7967	8053	-	59
NW_015799159.1	5690	5771	+	81
NW_015799259.1	24193	24275	-	82
NW_015799308.1	8578	8719	-	141
NW_015799321.1	12430	12495	-	65
NW_015799347.1	220745	221172	+	327
NW_015799398.1	14656	14765	+	109

NW_015799619.1	16523	16600	-	77
NW_015799621.1	6894	6985	-	91
NW_015799748.1	21656	21824	-	168
NW_015799897.1	15697	15781	-	84
NW_015799963.1	1669	1800	-	131
NW_015799986.1	57278	57361	+	83
NW_015799986.1	57262	57494	+	232
NW_015800001.1	32186	32401	+	215
NW_015800049.1	3871	4004	-	133
NW_015800091.1	33732	33851	+	119
NW_015800126.1	6009	6194	+	94
NW_015800145.1	110031	110224	+	193
NW_015800263.1	10680	10865	-	115
NW_015800391.1	2718	2748	+	30
NW_015800391.1	2670	2897	+	188
NW_015800428.1	75016	75167	-	151
NW_015800467.1	3241	3359	+	118
NW_015800471.1	10739	10846	+	107
NW_015800589.1	10629	10728	+	99
NW_015800590.1	32389	32534	-	145
NW_015800595.1	1427	1597	-	170
NW_015800604.1	32218	32297	-	79
NW_015800635.1	17864	18194	-	330
NW_015800658.1	14373	14599	-	226
NW_015800675.1	52284	52367	+	83

NW_015800693.1	7810	7867	+	57
NW_015800702.1	25937	25999	+	62
NW_015800713.1	23796	23858	+	62
NW_015800760.1	19721	19777	+	56
NW_015800870.1	15324	15461	+	114
NW_015801051.1	6607	6935	-	328
NW_015801233.1	51977	52064	+	87
NW_015801281.1	80858	80968	-	110
NW_015801297.1	46382	46554	+	172
NW_015801552.1	62319	62557	+	238
NW_015801629.1	34044	34257	+	213
NW_015801726.1	75947	76107	+	160
NW_015801759.1	15384	15482	-	98
NW_015801759.1	15128	15469	-	341
NW_015801786.1	12668	12799	+	131
NW_015801816.1	32211	32303	-	61
NW_015801873.1	17660	17791	+	75
NW_015801924.1	58518	58665	-	147
NW_015801952.1	38315	38520	-	205
NW_015801952.1	38170	38600	-	430
NW_015801978.1	51278	51463	-	185
NW_015802410.1	23448	23510	+	62
NW_015802430.1	6778	6914	+	136
NW_015802432.1	33076	33447	+	371
NW_015802438.1	32515	32563	-	48

NW_015802636.1	61225	61275	+	50
NW_015802748.1	25231	25368	-	137
NW_015802895.1	57023	57305	+	282
NW_015802928.1	32859	32942	+	83
NW_015802928.1	32854	33080	+	226
NW_015802963.1	15368	15459	+	91
NW_015803008.1	8498	8719	-	25
NW_015803120.1	27310	27413	+	103
NW_015803333.1	13595	13739	-	135
NW_015803472.1	28674	28735	+	61
NW_015803517.1	66240	66344	-	104
NW_015803671.1	1088	1315	-	227
NW_015803686.1	14577	14644	-	67
NW_015803686.1	14464	14757	-	267
NW_015803742.1	16984	17080	-	96
NW_015803759.1	1740	1846	+	106
NW_015803783.1	54232	54290	+	58
NW_015803794.1	65228	65325	-	97
NW_015803836.1	1612	1847	+	200
NW_015803949.1	19777	19936	-	159
NW_015803996.1	23086	23247	+	161
NW_015804188.1	22569	22789	-	220
NW_015804242.1	4963	5169	-	206
NW_015804352.1	1298	1353	-	55
NW_015804499.1	55595	55675	+	80

NW_015804590.1	32412	32595	-	144
NW_015804590.1	32451	32596	-	105
NW_015804590.1	32395	32593	-	161
NW_015804671.1	12816	12880	-	64
NW_015804843.1	7545	7635	-	90
NW_015804894.1	116773	116819	-	46
NW_015804904.1	40133	40401	+	268
NW_015805049.1	39924	40140	+	216
NW_015805054.1	10625	10712	+	87
NW_015805116.1	24850	24936	+	86
NW_015805116.1	24856	24971	+	115
NW_015805125.1	5593	5733	+	140
NW_015805172.1	96579	96631	+	52
NW_015805172.1	32519	32647	-	128
NW_015805172.1	96607	96663	+	56
NW_015805344.1	17792	17923	-	131
NW_015805539.1	74368	74452	-	84
NW_015805636.1	27929	28086	-	83
NW_015805863.1	22957	23023	-	66
NW_015806168.1	11792	11961	-	169
NW_015806177.1	13963	14060	-	97
NW_015806190.1	40398	40430	-	32
NW_015806190.1	40536	40790	-	254
NW_015806450.1	21110	21217	-	107
NW_015806485.1	21524	21579	-	55

NW_015806485.1	21527	21595	-	68
NW_015806525.1	30245	30416	-	134
NW_015806650.1	13516	13709	-	193
NW_015806880.1	429290	429386	-	96
NW_015807019.1	23798	23895	+	97
NW_015807042.1	41229	41406	-	82
NW_015807042.1	59855	59932	+	77
NW_015807042.1	36748	36830	-	82
NW_015807125.1	48523	48692	+	169
NW_015807148.1	8253	8402	+	149
NW_015807318.1	7941	7984	-	43
NW_015807343.1	30572	30594	+	22
NW_015807383.1	17850	18043	+	193
NW_015807419.1	16805	16889	-	84
NW_015807433.1	2684	2758	-	74
NW_015807521.1	450275	450334	-	59
NW_015807570.1	2297	2364	+	67
NW_015807602.1	17526	17644	-	118
NW_015807602.1	9563	9709	+	91
NW_015807629.1	164402	164453	+	51
NW_015807712.1	9594	9737	+	143
NW_015807828.1	16448	16565	+	117
NW_015807834.1	26367	26434	+	67
NW_015808065.1	8675	9052	-	377
NW_015808212.1	103141	103179	-	38

NW_015808282.1	24839	24913	-	54
NW_015808424.1	30178	30286	+	108
NW_015808465.1	13046	13340	-	191
NW_015808587.1	20832	20976	+	144
NW_015808679.1	6441	6580	+	139
NW_015808796.1	17825	17920	-	95
NW_015808808.1	49808	50089	-	281
NW_015808839.1	20652	20794	+	142
NW_015808904.1	998	1196	-	103
NW_015808958.1	8755	8968	-	213
NW_015808973.1	11913	12148	-	235
NW_015808999.1	66856	66984	-	128
NW_015809059.1	23145	23302	-	157
NW_015809059.1	23128	23311	-	183
NW_015809094.1	16913	17007	-	68
NW_015809223.1	19728	19859	-	131
NW_015809247.1	25392	25561	-	169
NW_015809346.1	93140	93270	+	108
NW_015809447.1	2254	2282	-	28
NW_015809447.1	2269	2304	-	35
NW_015809526.1	102178	102323	-	145
NW_015809543.1	60121	60431	-	117
NW_015809582.1	32074	32199	-	125
NW_015809596.1	2889	3084	-	195
NW_015809613.1	11692	11784	-	92

NW_015809613.1	11685	11785	-	100
NW_015809667.1	50808	50912	-	104
NW_015809705.1	31300	31351	-	51
NW_015809922.1	258977	259134	-	157
NW_015810183.1	469	606	-	137
NW_015810183.1	9125	9246	-	121
NW_015810194.1	43106	43214	+	104
NW_015810259.1	24794	25006	-	212
NW_015810259.1	24799	25113	-	314
NW_015810285.1	21791	21917	-	126
NW_015810395.1	5795	5983	-	111
NW_015810449.1	2285	2394	-	109
NW_015810452.1	2773	2924	+	151
NW_015810795.1	1961	2085	+	124
NW_015810839.1	52008	52045	+	37
NW_015810839.1	96274	96527	+	253
NW_015810846.1	38174	38249	-	75
NW_015810891.1	4305	4353	+	48
NW_015810909.1	3035	3201	+	166
NW_015810917.1	43009	43219	-	210
NW_015811024.1	15467	15607	-	140
NW_015811032.1	21080	21272	-	175
NW_015811046.1	12524	12648	-	124
NW_015811144.1	23566	23645	+	79
NW_015811149.1	14072	14149	+	77

NW_015811149.1	13882	14286	+	404
NW_015811167.1	153228	153327	+	99
NW_015811179.1	20207	20357	-	105
NW_015811280.1	28278	28425	-	147
NW_015811527.1	24570	24758	+	188
NW_015811553.1	3011	3198	-	187
NW_015811571.1	41227	41316	-	89
NW_015811605.1	9837	9938	-	74
NW_015811787.1	20945	21365	+	420
NW_015811867.1	70204	70309	+	105
NW_015811883.1	109744	109892	+	148
NW_015811940.1	26226	26397	+	171
NW_015811940.1	27514	27632	+	118
NW_015812026.1	13953	14051	-	98
NW_015812256.1	15446	15737	-	291
NW_015812256.1	15428	15674	-	246
NW_015812279.1	7641	7675	+	34
NW_015812421.1	35585	35719	-	134
NW_015812421.1	31305	31407	-	102
NW_015812492.1	56628	57067	-	439
NW_015812517.1	35234	35345	-	111
NW_015812562.1	14784	14887	-	103
NW_015812596.1	158435	158540	+	105
NW_015812707.1	40602	40659	-	57
NW_015812716.1	13897	13992	+	67

NW_015812872.1	12465	12699	+	234
NW_015812872.1	13938	14120	+	182
NW_015812873.1	5298	5358	+	60
NW_015813061.1	96943	97027	-	84
NW_015813214.1	30422	30509	-	87
NW_015813270.1	58746	58828	-	82
NW_015813270.1	11024	11087	-	63
NW_015813270.1	59024	59129	-	105
NW_015813270.1	11203	11344	-	141
NW_015813270.1	59041	59125	-	84
NW_015813457.1	8814	8873	+	59
NW_015813499.1	85602	85632	+	30
NW_015813595.1	6931	7175	+	244
NW_015813708.1	107401	107506	-	105
NW_015813738.1	48425	48522	-	97
NW_015813771.1	20098	20190	+	92
NW_015813915.1	14311	14415	-	104
NW_015813924.1	1591	1777	+	104
NW_015814079.1	3188	3251	-	63
NW_015814572.1	18049	18167	-	78
NW_015814572.1	18076	18189	-	28
NW_015814595.1	23473	23515	+	42
NW_015814616.1	7659	7748	+	89
NW_015814715.1	1788	1899	+	111
NW_015814740.1	12042	12150	-	108

NW_015814766.1	8275	8409	-	134
NW_015814964.1	103101	103245	-	144
NW_015815014.1	3149	3290	+	141
NW_015815186.1	55454	55645	-	191
NW_015815195.1	107967	108126	-	29
NW_015815430.1	122804	123043	-	239
NW_015815430.1	122811	123486	-	675
NW_015815661.1	3291	3374	+	83
NW_015815745.1	7551	7701	-	150
NW_015815757.1	68165	68297	+	132
NW_015815772.1	8153	8361	-	208
NW_015815911.1	18921	19012	+	91
NW_015815911.1	18840	19018	+	178
NW_015815950.1	14474	14593	-	119
NW_015815959.1	92966	93111	-	145
NW_015816087.1	26877	27100	-	223
NW_015816359.1	186018	186203	-	185
NW_015816450.1	17465	17554	+	55
NW_015816626.1	3557	3640	-	83
NW_015816700.1	41478	41565	-	87
NW_015816928.1	16818	16944	+	126
NW_015816976.1	11874	12054	+	180
NW_015817139.1	136593	136711	-	118
NW_015817148.1	17160	17256	+	96
NW_015817164.1	26626	26680	-	54

NW_015817388.1	4244	4416	-	172
NW_015817445.1	6079	6211	+	101
NW_015817507.1	12784	12949	-	165
NW_015817575.1	20878	21059	-	181
NW_015817657.1	8377	8590	-	213
NW_015818126.1	2066	2152	+	86
NW_015818305.1	38260	38349	+	60
NW_015818386.1	139657	139932	-	275
NW_015818529.1	5250	5378	-	47
NW_015818566.1	15861	16229	+	368
NW_015818635.1	18278	18453	-	175
NW_015818789.1	16071	16260	-	189
NW_015818998.1	15040	15102	-	60
NW_015819123.1	6937	7016	+	79
NW_015819179.1	196857	197018	+	93
NW_015819357.1	2103	2220	+	117
NW_015819357.1	2085	2267	+	182
NW_015819405.1	17453	17496	+	43
NW_015819643.1	25090	25137	-	47
NW_015819643.1	25096	25135	-	39
NW_015819698.1	16309	16584	+	37
NW_015819880.1	12763	12915	-	152
NW_015819909.1	32538	32678	-	140
NW_015819923.1	40403	40462	-	59
NW_015820176.1	27904	27997	-	93

NW_015820259.1	74701	74961	+	260
NW_015820923.1	18030	18173	-	143
NW_015820924.1	29020	29175	-	155
NW_015820962.1	528	642	-	114
NW_015821064.1	23366	23392	-	26
NW_015821092.1	18	326	+	203
NW_015821240.1	17257	17338	-	81
NW_015821277.1	14695	14942	-	247
NW_015821305.1	24923	25061	+	138
NW_015821418.1	9400	9534	+	134
NW_015821418.1	9409	9541	+	132
NW_015821476.1	126414	126524	+	110
NW_015821533.1	42536	42578	+	42
NW_015821718.1	16375	16529	+	154
NW_015821718.1	16385	16554	+	169
NW_015821994.1	80395	80445	-	50
NW_015822001.1	13952	14086	-	88
NW_015822052.1	46156	46250	-	94
NW_015822338.1	17185	17287	-	102
NW_015822674.1	52779	52973	-	194
NW_015822755.1	4630	4793	-	163
NW_015822828.1	10260	10429	-	169
NW_015822881.1	53466	53572	+	106
NW_015822915.1	50798	50836	+	38
NW_015822943.1	9833	9943	-	110

NW_015823041.1	52869	52931	-	62
NW_015823203.1	12921	13025	+	104
NW_015823272.1	29390	29401	-	11
NW_015823374.1	17012	17054	+	42
NW_015823838.1	12927	13009	-	82
NW_015823838.1	3395	3674	-	279
NW_015823840.1	75310	75451	-	141
NW_015823858.1	27170	27315	-	145
NW_015823883.1	38265	38441	+	176
NW_015824270.1	54218	54297	+	79
NW_015824635.1	5399	5448	+	49
NW_015824723.1	77236	77268	+	32
NW_015824769.1	9229	9273	+	44
NW_015824925.1	47653	47676	-	23
NW_015825287.1	4616	4743	-	127
NW_015825357.1	90174	90353	-	172
NW_015825368.1	273985	274094	-	71
NW_015825418.1	76428	76521	-	93
NW_015825460.1	4480	4541	+	61
NW_015825712.1	31366	31407	-	41
NW_015825712.1	31681	31933	-	252
NW_015825823.1	17828	17959	+	131
NW_015825914.1	52119	52232	-	113
NW_015825953.1	23853	24073	-	220
NW_015826083.1	3963	4105	-	142

NW_015826087.1	16361	16444	-	83
NW_015826102.1	18926	19005	+	79
NW_015826229.1	24478	24548	-	70
NW_015826233.1	17845	18013	+	168
NW_015826233.1	17834	18025	+	191
NW_015826233.1	17821	18007	+	186
NW_015826295.1	12339	12481	+	142
NW_015826469.1	21520	21578	-	58
NW_015826530.1	12974	13124	+	150
NW_015826660.1	10262	10298	+	36
NW_015826704.1	33663	33876	+	213
NW_015827184.1	28639	28766	-	127
NW_015827447.1	21640	21819	+	41
NW_015827689.1	75167	75382	-	215
NW_015827739.1	4856	5001	+	145
NW_015827773.1	1924	2034	+	110
NW_015827801.1	116321	116411	-	70
NW_015827801.1	175842	176029	-	115
NW_015827801.1	187355	187479	-	124
NW_015827913.1	30723	30975	+	252
NW_015828189.1	2643	2715	+	72
NW_015828196.1	26663	26735	+	63
NW_015828218.1	12433	12597	+	164
NW_015828272.1	28558	28602	-	44
NW_015828272.1	28333	28594	-	261

NW_015828413.1	9109	9360	+	251
NW_015828505.1	33752	33771	-	19
NW_015828505.1	43492	43568	+	76
NW_015828634.1	63391	63558	+	167
NW_015828882.1	36187	36338	-	117
NW_015828926.1	11658	11916	-	258
NW_015829103.1	38480	38567	-	87
NW_015829308.1	15290	15360	-	70
NW_015829444.1	85677	85819	+	142
NW_015829534.1	59350	59737	-	387
NW_015829638.1	3618	3762	+	144
NW_015829643.1	11946	12069	+	123
NW_015829661.1	83951	84145	+	194
NW_015829671.1	27010	27077	-	67
NW_015829679.1	19228	19344	-	116
NW_015829824.1	7679	7830	+	151
NW_015829915.1	9238	9300	-	62
NW_015829975.1	4809	4857	-	48
NW_015829975.1	5303	5366	-	63
NW_015830040.1	18612	18716	+	104
NW_015830272.1	39726	39820	+	94
NW_015830592.1	3039	3243	+	204
NW_015830779.1	6310	6420	+	110
NW_015830873.1	24809	24945	+	113
NW_015830948.1	91774	91823	+	49

NW_015830966.1	3721	3835	+	114
NW_015831077.1	3824	3983	+	159
NW_015831077.1	3782	3959	+	177
NW_015831258.1	16461	16568	+	90
NW_015831538.1	33418	33515	-	97
NW_015831606.1	22240	22323	-	83
NW_015831630.1	16104	16231	-	127
NW_015831798.1	59010	59219	-	172
NW_015831835.1	22855	22973	-	118
NW_015832227.1	26310	26387	+	77
NW_015832227.1	27589	27672	+	83
NW_015832678.1	5590	5685	+	95
NW_015832748.1	31798	31827	-	29
NW_015833075.1	14586	14726	+	140
NW_015833075.1	17751	17955	+	72
NW_015833169.1	4377	4580	-	203
NW_015833195.1	3507	3597	+	90
NW_015833306.1	53656	53794	+	138
NW_015833460.1	36870	37198	+	328
NW_015833563.1	12836	12943	-	107
NW_015833585.1	26513	26622	-	109
NW_015833943.1	6056	6127	-	71
NW_015834007.1	22422	22473	+	51
NW_015834229.1	15542	15625	-	83
NW_015834252.1	16242	16381	+	139

NW_015834252.1	16243	16379	+	136
NW_015834252.1	16154	16388	+	234
NW_015834252.1	16821	16951	+	130
NW_015834252.1	29314	29735	+	401
NW_015834373.1	3118	3308	-	139
NW_015834379.1	91675	91793	-	118
NW_015834423.1	29952	30088	+	136
NW_015834901.1	222387	222650	-	263
NW_015834952.1	137383	137649	+	266
NW_015835004.1	250072	250134	+	62
NW_015835108.1	51407	51436	-	29
NW_015835141.1	5524	5667	+	143
NW_015835169.1	48110	48316	-	206
NW_015835205.1	82006	82293	-	287
NW_015835254.1	63409	63660	-	251
NW_015835254.1	63413	63643	-	230
NW_015835299.1	11054	11280	-	226
NW_015835316.1	27293	27399	-	106
NW_015835420.1	8299	8393	-	94
NW_015835466.1	5764	5914	-	150
NW_015835530.1	17554	17689	-	129
NW_015835557.1	6575	6720	+	104
NW_015835620.1	184275	184428	-	42
NW_015835668.1	125867	125996	-	129
NW_015835668.1	125701	125945	-	161

NW_015835809.1	23961	24007	-	46
NW_015835822.1	3515	3669	+	154
NW_015835841.1	29625	29717	+	92
NW_015835842.1	4928	4990	+	62
NW_015835922.1	51585	51713	-	128
NW_015836007.1	7413	7473	-	60
NW_015836007.1	7670	7980	-	310
NW_015836056.1	4605	4666	-	61
NW_015836126.1	16084	16205	-	121
NW_015836160.1	64369	64530	+	90
NW_015836396.1	1781	1921	-	140
NW_015836620.1	8656	8764	+	102
NW_015836672.1	3037	3164	-	127
NW_015836672.1	4969	5066	-	97
NW_015836983.1	15985	16074	+	89
NW_015836983.1	27433	27494	-	61
NW_015837106.1	79891	79943	-	52
NW_015837151.1	83016	83106	+	2
NW_015837315.1	117301	117513	+	212
NW_015837436.1	154094	154327	-	233
NW_015837461.1	4503	4602	-	99
NW_015837843.1	74317	74389	-	72
NW_015837843.1	313622	313732	-	110
NW_015837861.1	14610	14684	-	74
NW_015837861.1	8565	8617	+	52

NW_015837869.1	5867	5957	+	90
NW_015837991.1	108742	108790	+	48
NW_015838006.1	63397	63504	+	107
NW_015838024.1	25403	25504	-	101
NW_015838024.1	25402	25486	-	84
NW_015838024.1	25417	25499	-	82
NW_015838024.1	49404	49624	-	220
NW_015838100.1	24580	24839	+	259
NW_015838133.1	3176	3433	-	257
NW_015838488.1	7857	7989	-	132
NW_015838618.1	17505	17624	+	119
NW_015838732.1	129439	129570	-	131
NW_015838911.1	24275	24332	-	57
NW_015839053.1	11024	11117	+	93
NW_015839194.1	38714	38803	+	89
NW_015839479.1	85480	85537	-	57
NW_015840034.1	12714	12783	+	69
NW_015840054.1	3237	3622	+	385
NW_015840082.1	23257	23390	-	133
NW_015840082.1	23829	23933	-	68
NW_015840099.1	2449	2625	-	176
NW_015840344.1	24914	24992	-	76
NW_015840600.1	23805	23864	+	59
NW_015840644.1	55150	55322	+	172
NW_015840758.1	142746	142983	+	237

NW_015840769.1	15431	15641	-	210
NW_015840967.1	33806	33914	+	108
NW_015840967.1	33804	33927	+	123
NW_015841106.1	62304	62446	-	90
NW_015841254.1	31194	31236	+	42
NW_015841532.1	14993	15167	-	174
NW_015841952.1	11011	11037	+	26
NW_015842213.1	102864	102954	-	58
NW_015842290.1	15619	15906	-	287
NW_015842356.1	21491	21635	+	144
NW_015842452.1	64506	64637	+	131
NW_015842637.1	85501	85677	-	176
NW_015842647.1	1463	1567	+	104
NW_015842735.1	6631	6712	+	81
NW_015842749.1	81202	81306	+	104
NW_015842833.1	164077	164214	+	137
NW_015843003.1	106496	106809	-	313
NW_015843060.1	278	401	-	123
NW_015843061.1	4417	4665	+	248
NW_015843064.1	4604	4723	+	119
NW_015843241.1	11869	11984	+	71
NW_015843409.1	47731	47871	+	140
NW_015843537.1	40494	40709	-	215
NW_015843805.1	8425	8702	+	277
NW_015843847.1	120143	120244	-	101

NW_015843865.1	19838	19942	+	104
NW_015843929.1	7337	7403	+	66
NW_015843980.1	27552	27638	-	86
NW_015843980.1	27550	27678	-	128
NW_015844092.1	130955	131146	+	191
NW_015844117.1	68856	68954	+	98
NW_015844125.1	5195	5292	-	97
NW_015844511.1	31546	31665	+	119
NW_015844808.1	3881	4020	-	139
NW_015844855.1	17123	17201	-	78
NW_015845111.1	75674	75860	+	186
NW_015845277.1	61375	61463	+	88
NW_015845424.1	11546	11612	+	66
NW_015845892.1	7556	7625	-	69
NW_015846045.1	5823	6194	+	371
NW_015846225.1	7366	7474	+	108
NW_015846278.1	16086	16237	-	151
NW_015846331.1	15788	16109	-	321
NW_015846331.1	52073	52181	+	108
NW_015846425.1	253433	253578	-	145
NW_015846493.1	68273	68453	+	135
NW_015846684.1	27516	27623	-	107
NW_015847079.1	2960	3098	+	138
NW_015847101.1	23788	23867	-	79
NW_015847145.1	49502	49578	+	76

NW_015847259.1	51296	51395	-	99
NW_015847361.1	40194	40530	-	336
NW_015847483.1	10103	10279	-	23
NW_015847589.1	5061	5256	+	195
NW_015847612.1	13732	13881	+	96
NW_015847612.1	18760	18926	+	84
NW_015847674.1	46129	46325	-	196
NW_015847690.1	12934	13039	-	105
NW_015847728.1	205404	205467	+	63
NW_015848009.1	63	143	+	80
NW_015848425.1	35349	35446	+	97
NW_015848535.1	66707	67062	+	355
NW_015848535.1	48914	49058	-	144
NW_015848846.1	75313	75505	-	192
NW_015849056.1	8365	8631	+	266
NW_015849405.1	7096	7216	+	120
NW_015849632.1	27810	28001	+	191
NW_015849778.1	49630	49745	+	115
NW_015849848.1	3228	3427	+	199
NW_015850078.1	48280	48429	+	149
NW_015850137.1	4699	5223	+	524
NW_015850182.1	27452	27511	-	59
NW_015850191.1	157891	157993	+	102
NW_015850369.1	3653	3765	-	112
NW_015850497.1	35926	35989	-	63

NW_015850520.1	4433	4506	-	73
NW_015850520.1	4388	4482	-	94
NW_015850570.1	4554	4662	+	108
NW_015850570.1	4330	4657	+	327
NW_015850766.1	37041	37074	-	33
NW_015850766.1	37039	37072	-	33
NW_015850766.1	37036	37099	-	63
NW_015850846.1	2932	2989	-	57
NW_015850890.1	2938	3104	-	166
NW_015851005.1	32691	32838	-	147
NW_015851138.1	36471	36674	+	203
NW_015851465.1	47759	47840	-	81
NW_015851491.1	7120	7176	-	56
NW_015851534.1	58807	58915	+	108
NW_015851596.1	29563	29765	+	202
NW_015851810.1	22144	22513	-	369
NW_015852066.1	8179	8345	-	166
NW_015852154.1	5535	5625	-	90
NW_015852189.1	53808	53995	+	187
NW_015852229.1	12942	12976	-	34
NW_015852367.1	39016	39345	-	329
NW_015852473.1	16026	16107	+	81
NW_015852649.1	37447	37604	-	29
NW_015852649.1	57514	57572	-	58
NW_015852803.1	152428	152480	-	52

NW_015852854.1	59937	60113	-	176
NW_015853201.1	28123	28313	+	118
NW_015853252.1	14187	14321	+	134
NW_015853252.1	15355	15519	-	164
NW_015853270.1	17352	17574	-	155
NW_015853326.1	110490	110591	+	101
NW_015853488.1	41352	41451	-	99
NW_015853692.1	32247	32346	+	75
NW_015853978.1	27833	27899	+	66
NW_015853978.1	28792	28966	+	174
NW_015854074.1	70884	71129	-	245
NW_015854114.1	13869	13902	+	33
NW_015854246.1	8539	8658	+	119
NW_015854284.1	132828	133254	-	426
NW_015854361.1	57673	57890	+	217
NW_015854495.1	2638	2812	-	90
NW_015854664.1	5194	5327	-	133
NW_015854682.1	38193	38411	-	58
NW_015854738.1	4265	4319	+	54
NW_015854875.1	4242	4398	-	156
NW_015855245.1	12151	12296	-	77
NW_015855478.1	40238	40367	-	129
NW_015856130.1	35801	35946	-	145
NW_015856211.1	43536	43635	+	51
NW_015856440.1	2759	2897	-	87

NW_015856591.1	51652	51868	+	216
NW_015856591.1	28825	28908	+	83
NW_015856591.1	28809	28914	+	105
NW_015856988.1	58115	58175	-	60
NW_015857129.1	8133	8240	+	107
NW_015857273.1	8763	8859	-	96
NW_015857278.1	27938	28014	+	76
NW_015857309.1	21857	21873	+	16
NW_015857473.1	30031	30171	-	140
NW_015857473.1	30027	30175	-	148
NW_015857518.1	73589	73632	-	43
NW_015857522.1	6220	6263	+	43
NW_015857528.1	16198	16306	-	89
NW_015857968.1	25548	25627	+	79
NW_015857980.1	3119	3262	-	107
NW_015858103.1	26070	26133	-	63
NW_015858206.1	77410	77552	+	142
NW_015858469.1	50785	50814	-	29
NW_015858595.1	98238	98355	+	117
NW_015858629.1	70609	70842	-	233
NW_015858705.1	8222	8285	+	63
NW_015858865.1	8874	9087	+	213
NW_015858865.1	13829	13892	+	63
NW_015858887.1	60817	60870	+	53
NW_015858915.1	8392	8758	+	366

NW_015858944.1	42926	43122	+	196
NW_015858944.1	46133	46215	+	57
NW_015859095.1	54387	54699	-	312
NW_015859095.1	44122	44457	+	335
NW_015859285.1	22627	22725	+	98
NW_015859350.1	37847	37979	-	132
NW_015859602.1	38380	38904	-	524
NW_015859645.1	35212	35425	+	154
NW_015859709.1	25180	25384	+	204
NW_015859774.1	5671	5797	+	126
NW_015859920.1	17295	17384	-	89
NW_015859926.1	4431	4663	+	213
NW_015860134.1	12195	12325	-	122
NW_015860193.1	60163	60297	-	115
NW_015860202.1	49525	49708	-	183
NW_015860202.1	193020	193145	+	125
NW_015860202.1	49511	49622	-	111
NW_015860202.1	49495	49651	-	156
NW_015860202.1	88144	88458	-	314
NW_015860202.1	180166	180233	+	67
NW_015860375.1	37991	38030	-	39
NW_015860800.1	5246	5304	-	58
NW_015860977.1	148314	148410	-	73
NW_015861192.1	9140	9238	-	98
NW_015861237.1	5253	5323	+	39

NW_015861375.1	77839	78122	+	283
NW_015861375.1	77831	78123	+	292
NW_015861375.1	92342	92497	-	155
NW_015861441.1	63886	63967	-	81
NW_015861485.1	9070	9429	+	359
NW_015861531.1	13075	13226	+	78
NW_015861812.1	96429	96557	-	92
NW_015861972.1	71390	71498	-	108
NW_015862136.1	166595	166722	+	127
NW_015862256.1	39900	39944	+	44
NW_015862325.1	1932	2098	+	166
NW_015862329.1	35545	35650	-	105
NW_015862386.1	29114	29178	+	64
NW_015862386.1	29067	29241	+	174
NW_015862463.1	16203	16406	-	203
NW_015862590.1	4552	4772	+	220
NW_015862625.1	2606	2696	+	90
NW_015862657.1	13529	13607	+	78
NW_015862657.1	13342	13583	+	241
NW_015862776.1	32415	32508	+	93
NW_015862803.1	52932	53117	+	185
NW_015863028.1	132278	132345	+	67
NW_015863090.1	13811	13994	-	183
NW_015863095.1	27067	27278	+	58
NW_015863115.1	117987	118140	+	153

NW_015863169.1	1889	2239	+	350
NW_015863195.1	37931	38052	+	121
NW_015863248.1	29196	29222	-	26
NW_015863297.1	15734	15804	+	70
NW_015863298.1	37368	37761	+	393
NW_015863355.1	205096	205172	-	76
NW_015863429.1	25282	25371	+	89
NW_015863580.1	3045	3098	-	53
NW_015863661.1	1446	1574	-	128
NW_015863661.1	1440	1581	-	141
NW_015863675.1	149478	149561	+	83
NW_015863709.1	25246	25300	-	54
NW_015863778.1	16089	16132	-	43
NW_015863803.1	57809	58055	+	246
NW_015863857.1	13007	13272	+	265
NW_015864017.1	160683	160772	+	89
NW_015864136.1	29928	30188	+	260
NW_015864256.1	11482	11576	-	94
NW_015864371.1	29651	29801	-	150
NW_015864383.1	39808	40083	+	275
NW_015864532.1	133899	134062	-	163
NW_015864776.1	33868	33996	+	128
NW_015864800.1	59155	59428	+	273
NW_015864801.1	13731	13841	-	110
NW_015864846.1	44312	44357	-	45

NW_015864951.1	12944	12996	-	52
NW_015865234.1	30732	30824	-	92
NW_015865234.1	30716	30843	-	127
NW_015865244.1	18893	18971	-	65
NW_015865250.1	4316	4549	-	233
NW_015865282.1	166820	166928	-	108
NW_015865451.1	63786	63936	+	150
NW_015865486.1	770	1021	+	51
NW_015865588.1	38456	38504	-	48
NW_015865736.1	746	912	+	145
NW_015865812.1	32815	32922	+	107
NW_015865964.1	8754	8794	+	40
NW_015865988.1	21720	22012	-	224
NW_015866069.1	29723	29820	+	97
NW_015866417.1	6237	6393	+	156
NW_015866472.1	21450	21579	-	129
NW_015866573.1	99411	99485	-	74
NW_015866788.1	77199	77576	-	377
NW_015866792.1	15974	16033	+	59
NW_015866792.1	13354	13517	+	163
NW_015866820.1	47329	47443	+	114
NW_015867070.1	6055	6157	+	102
NW_015867266.1	56775	56876	-	101
NW_015867266.1	56647	56878	-	231
NW_015867266.1	56599	56883	-	284

NW_015867332.1	52014	52106	+	92
NW_015867373.1	122317	122414	+	97
NW_015867373.1	183017	183096	-	79
NW_015867414.1	22385	22421	-	36
NW_015867726.1	86869	86892	-	23
NW_015867761.1	12319	12691	-	104
NW_015867942.1	37312	37464	-	77
NW_015867979.1	135680	135768	-	88
NW_015868019.1	117707	117848	-	141
NW_015868054.1	51828	51879	+	25
NW_015868068.1	40723	40836	+	113
NW_015868090.1	97058	97464	+	406
NW_015868129.1	4344	4439	+	95
NW_015868191.1	72994	73159	+	165
NW_015868713.1	45457	45502	-	45
NW_015869203.1	19553	19718	+	165
NW_015869440.1	28066	28222	+	156
NW_015869440.1	28070	28118	+	48
NW_015869440.1	28076	28302	+	226
NW_015869630.1	3528	3652	+	124
NW_015869744.1	67955	68033	+	78
NW_015869887.1	10911	11199	+	288
NW_015870077.1	4523	4543	+	20
NW_015870077.1	4511	4573	+	62
NW_015870188.1	9428	9479	-	51

NW_015870340.1	31579	31665	-	86
NW_015870571.1	24527	24950	-	423
NW_015870645.1	42915	43015	+	100
NW_015870775.1	37556	37645	+	89
NW_015870822.1	26633	26784	+	151
NW_015870921.1	115878	115964	-	86
NW_015871032.1	2744	2919	-	175
NW_015871337.1	3527	3648	-	121
NW_015871411.1	7394	7567	+	173
NW_015871536.1	28647	28844	+	110
NW_015871635.1	146654	146748	+	94
NW_015871936.1	27585	27669	-	84
NW_015871965.1	10681	10759	+	78
NW_015871972.1	69897	70070	-	117
NW_015872001.1	32335	32431	-	96
NW_015872327.1	136276	136422	+	146
NW_015872592.1	5073	5155	-	82
NW_015872848.1	2118	2224	+	106
NW_015872852.1	2928	3045	-	117
NW_015872969.1	174741	174795	+	54
NW_015872981.1	128159	128285	+	126
NW_015872981.1	128023	128319	+	296
NW_015873293.1	26072	26278	-	206
NW_015873392.1	35165	35199	+	34
NW_015873614.1	8993	9152	+	159

NW_015873709.1	117724	117929	+	205
NW_015873761.1	51765	51839	-	74
NW_015873787.1	14234	14346	-	112
NW_015873855.1	5075	5155	-	80
NW_015873979.1	141239	141320	-	81
NW_015873979.1	141253	141395	-	142
NW_015874034.1	55303	55360	+	57
NW_015874093.1	55367	55562	+	195
NW_015874209.1	108947	109049	-	102
NW_015874389.1	24054	24160	-	104
NW_015874397.1	37275	37357	-	79
NW_015874831.1	79849	79943	+	94
NW_015874831.1	79842	79997	+	155
NW_015875034.1	2092	2232	-	137
NW_015875147.1	103	206	+	103
NW_015875291.1	35444	35553	+	109
NW_015875291.1	31794	31908	+	114
NW_015875291.1	34374	34515	+	141
NW_015875291.1	43128	43298	-	170
NW_015875383.1	60206	60304	-	66
NW_015875415.1	33249	33382	-	133
NW_015875724.1	57295	57399	-	104
NW_015876049.1	52273	52422	-	149
NW_015876049.1	54731	54860	-	129
NW_015876256.1	1331	1511	+	9

NW_015876301.1	203671	203774	+	87
NW_015876479.1	6888	6923	+	35
NW_015876563.1	139921	140058	-	74
NW_015876640.1	58278	58746	+	252
NW_015876645.1	4188	4474	+	286
NW_015876660.1	76376	76428	-	52
NW_015876710.1	51343	51477	-	124
NW_015876710.1	16344	16432	-	88
NW_015876772.1	8708	8840	+	132
NW_015876940.1	12181	12309	-	128
NW_015876969.1	47999	48131	-	132
NW_015877093.1	3422	3517	+	95
NW_015877132.1	69242	69504	+	93
NW_015877220.1	125124	125180	-	48
NW_015877324.1	36175	36257	-	82
NW_015877345.1	46480	46574	-	94
NW_015877540.1	79325	79563	+	238
NW_015877542.1	5288	5351	-	63
NW_015877638.1	26053	26129	-	76
NW_015878004.1	13558	13802	-	244
NW_015878320.1	73772	74054	-	282
NW_015878584.1	39585	39688	-	103
NW_015878644.1	130707	130875	-	168
NW_015878644.1	152089	152175	-	86
NW_015878786.1	24559	24741	-	182

NW_015878834.1	37136	37253	+	117
NW_015878836.1	7118	7178	+	60
NW_015878971.1	97216	97308	-	92
NW_015878971.1	97243	97281	-	38
NW_015879146.1	66250	66368	-	118
NW_015879256.1	10253	10289	-	36
NW_015879420.1	64929	65095	-	166
NW_015879420.1	64921	65252	-	331
NW_015879465.1	3919	4092	+	173
NW_015879547.1	107040	107126	+	86
NW_015879722.1	12623	12744	-	121
NW_015879830.1	10759	10900	+	141
NW_015880025.1	47249	47478	-	229
NW_015880240.1	17770	17834	-	64
NW_015880286.1	15975	16065	+	90
NW_015880286.1	15969	16085	+	116
NW_015880408.1	60154	60212	-	58
NW_015880450.1	99338	99465	+	127
NW_015880477.1	7000	7099	-	99
NW_015880531.1	25562	25650	+	88
NW_015880531.1	25133	25174	+	41
NW_015880571.1	64519	64605	-	86
NW_015880582.1	19843	19916	+	73
NW_015880582.1	12236	12333	+	97
NW_015880724.1	9662	9817	-	155

NW_015881068.1	4705	4806	-	53
NW_015881211.1	12019	12387	+	368
NW_015881469.1	756	931	-	156
NW_015881495.1	42278	42319	-	41
NW_015881495.1	42231	42327	-	96
NW_015881763.1	84254	84356	+	102
NW_015881786.1	22783	22857	+	74
NW_015882161.1	5545	5675	+	130
NW_015882277.1	9183	9300	+	117
NW_015882401.1	97633	97838	+	205
NW_015882452.1	48817	48858	+	41
NW_015882749.1	6312	6446	-	134
NW_015882749.1	6314	6454	-	140
NW_015882873.1	6161	6275	+	110
NW_015882938.1	2914	3172	+	258
NW_015883209.1	13763	13958	+	195
NW_015883269.1	102	209	-	38
NW_015883454.1	28503	28631	+	128
NW_015883696.1	19092	19641	+	549
NW_015883696.1	19302	19580	+	278
NW_015883696.1	19077	19670	+	593
NW_015884318.1	338	371	+	18
NW_015884339.1	80623	80797	+	113
NW_015884343.1	8357	8603	-	246
NW_015884381.1	89697	89788	-	91

NW_015884475.1	12105	12274	+	169
NW_015884635.1	46830	46953	-	123
NW_015885024.1	4294	4438	-	91
NW_015885042.1	7831	7900	-	69
NW_015885098.1	162938	163172	-	234
NW_015885141.1	25118	25277	-	159
NW_015885149.1	50575	50762	+	187
NW_015885159.1	253235	253320	-	85
NW_015885206.1	19323	19399	-	76
NW_015885206.1	19326	19429	-	103
NW_015885225.1	13138	13171	-	33
NW_015885244.1	35796	35866	+	70
NW_015885262.1	39598	39737	-	139
NW_015885307.1	21500	21516	-	16
NW_015885320.1	98105	98211	-	106
NW_015885407.1	68498	68613	-	115
NW_015885412.1	54431	54602	-	171
NW_015885435.1	26945	27007	-	62
NW_015885460.1	49989	50061	-	72
NW_015885498.1	26088	26326	-	238
NW_015885545.1	6954	7113	+	159
NW_015885606.1	96683	96859	+	176
NW_015885731.1	43276	43396	+	120
NW_015885780.1	162396	162478	+	82
NW_015885790.1	151418	151550	-	132

NW_015885797.1	21599	21688	+	89
NW_015885797.1	21614	21695	+	81
NW_015885805.1	126449	126476	+	27
NW_015885841.1	15106	15505	-	241
NW_015885869.1	31241	31276	+	35
NW_015885932.1	35412	35488	-	76
NW_015885938.1	86294	86381	+	87
NW_015885956.1	40423	40506	+	83
NW_015885985.1	32023	32489	-	466
NW_015886073.1	24340	24397	-	57
NW_015886146.1	18148	18386	+	127
NW_015886146.1	18128	18365	+	118
NW_015886209.1	14332	14382	+	50
NW_015886224.1	8621	8802	-	181
NW_015886265.1	62590	62637	+	47
NW_015886316.1	8106	8238	+	132
NW_015886361.1	79699	79796	+	97
NW_015886494.1	103682	103770	+	88
NW_015886494.1	115092	115380	+	288
NW_015886530.1	65622	65766	+	144
NW_015886542.1	31503	31624	+	121
NW_015886640.1	24750	24972	+	222
NW_015886687.1	9078	9206	-	128
NW_015886717.1	17304	17397	+	93
NW_015886717.1	17273	17406	+	133

NW_015886732.1	15686	15761	-	75
NW_015886753.1	57827	58053	-	226
NW_015886798.1	69532	69613	+	81
NW_015886814.1	38504	38729	+	225
NW_015886829.1	106344	106573	+	122
NW_015886909.1	4358	4611	-	253
NW_015886917.1	29964	30140	-	176
NW_015886924.1	21622	21753	+	131
NW_015886973.1	6287	6422	-	135
NW_015886973.1	6314	6414	-	100
NW_015886993.1	6384	6465	+	81
NW_015886993.1	6746	6780	+	34
NW_015886993.1	6738	6803	+	65
NW_015887068.1	30651	30929	-	278
NW_015887133.1	209276	209387	+	111
NW_015887163.1	18840	18941	+	101
NW_015887170.1	27812	27905	-	93
NW_015887272.1	72163	72295	+	132
NW_015887273.1	74010	74143	-	128
NW_015887350.1	3402	3665	+	263
NW_015887350.1	3325	3674	+	349
NW_015887373.1	176072	176171	+	99
NW_015887416.1	10515	10570	-	55
NW_015887430.1	4516	4635	+	119
NW_015887604.1	368151	368420	+	269

NW_015887675.1	21191	21331	-	140
NW_015887689.1	59675	59850	-	175
NW_015887692.1	17072	17182	+	110
NW_015887695.1	164639	164774	+	135
NW_015887726.1	182019	182154	-	135
NW_015887745.1	34551	34704	+	99
NW_015887761.1	41163	41372	+	209
NW_015887820.1	52247	52356	-	109
NW_015887820.1	52234	52348	-	114
NW_015887822.1	231965	232069	-	104
NW_015887828.1	46102	46213	-	64
NW_015887838.1	61386	61452	+	66
NW_015887841.1	82806	82872	+	66
NW_015887897.1	115730	115816	+	86
NW_015887950.1	18140	18289	+	149
NW_015887956.1	78490	78558	+	68
NW_015887976.1	78275	78338	+	63
NW_015888035.1	12260	12501	+	241
NW_015888119.1	30070	30260	+	190
NW_015888119.1	35210	35280	-	70
NW_015888133.1	13165	13263	-	98
NW_015888138.1	40040	40193	-	153
NW_015888207.1	96542	96661	+	119
NW_015888345.1	62701	62805	-	104
NW_015888425.1	57768	58151	-	383

NW_015888447.1	141666	141793	-	127
NW_015888462.1	39055	39111	+	56
NW_015888462.1	47474	47618	-	144
NW_015888518.1	5984	6141	+	157
NW_015888518.1	5981	6167	+	186
NW_015888536.1	49062	49137	+	75
NW_015888571.1	15178	15227	+	49
NW_015888575.1	56144	56249	-	105
NW_015888594.1	46405	46550	+	112
NW_015888618.1	51669	51745	+	76
NW_015888619.1	21362	21463	+	66
NW_015888663.1	6650	6767	+	117
NW_015888735.1	23561	23689	-	120
NW_015888785.1	161500	161617	-	117
NW_015888848.1	47128	47173	+	45
NW_015888863.1	8320	8404	+	84
NW_015889033.1	9544	9737	+	193
NW_015889038.1	21322	21489	-	167
NW_015889038.1	98352	98471	-	119
NW_015889070.1	5263	5331	-	68
NW_015889091.1	34819	34896	-	77
NW_015889108.1	10404	10501	-	97
NW_015889183.1	8601	8711	+	110
NW_015889224.1	18856	19020	-	164
NW_015889244.1	70326	70461	+	135

NW_015889250.1	37073	37215	+	120
NW_015889358.1	18169	18269	+	100
NW_015889381.1	108706	108879	+	139
NW_015889381.1	108713	108914	+	174
NW_015889400.1	150373	150455	-	82
NW_015889423.1	30168	30392	+	224
NW_015889466.1	31745	31887	+	142
NW_015889469.1	12175	12242	+	67
NW_015889491.1	116626	116693	-	67
NW_015889520.1	25313	25425	+	112
NW_015889562.1	12394	12480	+	86
NW_015889571.1	35201	35494	-	150
NW_015889581.1	19744	19945	+	154
NW_015889585.1	60301	60399	+	98
NW_015889625.1	68032	68179	+	147
NW_015889739.1	6118	6255	+	119
NW_015889739.1	6112	6266	+	130
NW_015889778.1	7258	7372	-	114
NW_015889806.1	40232	40427	+	195
NW_015889839.1	49346	49490	-	144
NW_015889858.1	146544	146712	+	168
NW_015889868.1	102429	102610	+	181
NW_015889879.1	13423	13525	+	102
NW_015889899.1	20963	20989	-	26
NW_015889934.1	22900	22953	-	53

NW_015890018.1	25654	25722	-	68
NW_015890023.1	21245	21341	+	96
NW_015890058.1	31916	32174	-	258
NW_015890058.1	32702	32866	-	164
NW_015890195.1	40645	40753	-	69
NW_015890216.1	4160	4185	+	25
NW_015890221.1	13435	13500	-	65
NW_015890270.1	40396	40603	-	207
NW_015890356.1	51300	51427	+	127
NW_015890547.1	38121	38203	+	82
NW_015890557.1	72561	72647	+	86
NW_015890593.1	20747	20924	+	115
NW_015890709.1	94450	94504	+	54
NW_015890709.1	94389	94529	+	140
NW_015890776.1	21175	21284	-	109
NW_015890817.1	12649	12795	-	109
NW_015890876.1	47310	47451	+	141
NW_015890884.1	72948	73098	-	77
NW_015890936.1	6988	7081	+	66
NW_015890942.1	37354	37729	-	375
NW_015891018.1	22097	22187	-	90
NW_015891022.1	28923	29015	-	92
NW_015891048.1	24936	24993	+	57
NW_015891085.1	30919	31032	-	113
NW_015891103.1	52849	52935	-	86

NW_015891104.1	193335	193466	-	73
NW_015891132.1	11087	11207	-	120
NW_015891132.1	20714	20763	+	49
NW_015891143.1	12634	13068	-	434
NW_015891143.1	12612	13066	-	454
NW_015891214.1	35251	35364	-	113
NW_015891257.1	31580	31724	+	105
NW_015891307.1	10715	10836	+	121
NW_015891425.1	48734	49013	+	279
NW_015891445.1	24348	24450	+	102
NW_015891517.1	27722	27865	+	143
NW_015891625.1	26885	26970	-	85
NW_015891625.1	26878	27017	-	139
NW_015891675.1	37837	37982	-	145
NW_015891723.1	79815	79892	+	77
NW_015891723.1	174559	174740	-	165
NW_015891764.1	81054	81142	+	88
NW_015891793.1	16595	16711	+	116
NW_015891796.1	63864	63955	-	91
NW_015891805.1	191002	191090	-	88
NW_015891867.1	280291	280436	+	145
NW_015891946.1	213301	213455	+	154
NW_015891946.1	281188	281278	+	90
NW_015892102.1	28370	28508	-	138
NW_015892102.1	28379	28560	-	181

NW_015892123.1	32496	32796	+	242
NW_015892219.1	16217	16308	-	91
NW_015892250.1	12098	12242	-	144
NW_015892338.1	11887	12051	+	164
NW_015892502.1	27208	27291	+	83
NW_015893144.1	746	985	+	155
NW_015893245.1	28501	28593	+	92
NW_015893570.1	55	75	+	20
NW_015893661.1	24552	24633	-	81
NW_015894633.1	23565	23800	+	235
NW_015894675.1	22949	23063	+	114
NW_015894708.1	14867	14933	-	66
NW_015894860.1	51861	52032	+	171
NW_015894954.1	535	647	-	112
NW_015895371.1	58369	58537	-	168
NW_015895536.1	28741	28882	-	141
NW_015895592.1	46337	46426	-	89
NW_015896148.1	6434	6451	+	17
NW_015896652.1	3482	3572	+	90
NW_015897178.1	93263	93338	-	75
NW_015897211.1	7480	7511	-	31
NW_015897495.1	3967	4178	+	211
NW_015897531.1	2075	2215	+	140
NW_015897627.1	94408	94518	-	110
NW_015897671.1	147539	147604	-	65

NW_015897767.1	2179	2441	+	262
NW_015897840.1	546	727	-	93
NW_015897856.1	39665	39693	-	28
NW_015897905.1	274	455	+	181
NW_015897905.1	273	471	+	198
NW_015897911.1	44435	44776	+	341
NW_015898036.1	198	234	+	36
NW_015898182.1	2278	2432	+	97
NW_015898218.1	95277	95336	-	59
NW_015898292.1	1364	1520	-	156
NW_015898386.1	771	880	+	109
NW_015898386.1	536	649	+	113
NW_015898468.1	343	421	+	50
NW_015898539.1	3116	3254	-	138
NW_015898590.1	1125	1238	+	48
NW_015898644.1	229	307	+	78
NW_015898644.1	228	326	+	98
NW_015898653.1	11228	11292	-	57
NW_015898700.1	4290	4503	-	35
NW_015898776.1	2557	2740	+	123
NW_015898782.1	4495	4625	+	113
NW_015898808.1	3671	3723	-	52
NW_015898845.1	2379	2516	+	137
NW_015898861.1	5318	5571	-	253
NW_015898877.1	6266	6435	-	169

NW_015898944.1	486	637	+	151
NW_015898986.1	392	501	+	109
NW_015899006.1	6135	6454	-	131
NW_015899040.1	24574	24615	-	41
NW_015899060.1	19648	19767	+	119
NW_015899120.1	71646	71775	-	129
NW_015899126.1	112536	112698	+	162
NW_015899134.1	41271	41379	-	108
NW_015899134.1	9368	9493	+	125
NW_015899136.1	32216	32415	-	199
NW_015899198.1	139906	140113	+	207
NW_015899201.1	16644	16842	+	198
NW_015899213.1	64945	64985	-	40
NW_015899245.1	45861	46014	-	153
NW_015899245.1	37617	37703	+	86
NW_015899255.1	26406	26469	+	63
NW_015899299.1	39739	39858	+	119
NW_015899321.1	17286	17366	-	80
NW_015899407.1	3014	3106	+	92
NW_015899443.1	107370	107438	+	68
NW_015899445.1	152400	152547	+	147
NW_015899519.1	23164	23204	-	40
NW_015899519.1	43183	43250	+	67
NW_015899562.1	18569	18709	-	140
NW_015899637.1	104222	104287	-	65

NW_015899650.1	59799	59873	-	74
NW_015899669.1	39163	39248	+	85
NW_015899800.1	21374	21688	-	174
NW_015899802.1	22825	22960	+	135
NW_015899863.1	170200	170256	-	56
NW_015899881.1	29722	30059	-	337
NW_015899892.1	59623	59773	-	107
NW_015899892.1	59609	59834	-	121
NW_015899892.1	59627	59826	-	103
NW_015899900.1	14575	14769	-	194
NW_015899943.1	33339	33499	+	160
NW_015899959.1	51759	51999	-	240
NW_015899983.1	200838	200963	+	125
NW_015899998.1	12470	12585	+	79
NW_015900033.1	26637	26748	+	111
NW_015900074.1	53495	53548	-	53
NW_015900090.1	12714	12872	+	158
NW_015900116.1	5376	5532	+	156
NW_015900222.1	671	758	+	87
NW_015900372.1	2490	2725	-	235
NW_015900434.1	18852	18916	-	64
NW_015900434.1	5638	5687	-	49
NW_015900449.1	78166	78239	+	73
NW_015900453.1	48676	48864	+	188
NW_015900541.1	16281	16338	-	57

NW_015900541.1	16266	16349	-	83
NW_015900558.1	82288	82352	+	64
NW_015900580.1	5321	5398	-	77
NW_015900580.1	5669	5934	-	149
NW_015900673.1	74288	74396	-	108
NW_015900673.1	74292	74387	-	95
NW_015900676.1	22488	22579	+	75
NW_015900711.1	39528	39685	+	157
NW_015900715.1	46297	46546	+	249
NW_015900715.1	46225	46535	+	310
NW_015900846.1	30876	31013	+	137
NW_015900953.1	192869	193021	+	99
NW_015900987.1	16618	16828	+	210
NW_015901028.1	56100	56190	-	90
NW_015901028.1	55420	55473	-	53
NW_015901029.1	65925	66024	+	99
NW_015901124.1	23615	23645	+	30
NW_015901126.1	84296	84458	-	162
NW_015901126.1	84240	84462	-	222
NW_015901130.1	21178	21290	-	112
NW_015901151.1	36081	36261	+	180
NW_015901153.1	159422	159529	-	107
NW_015901240.1	124027	124208	+	142
NW_015901249.1	48783	48859	-	76
NW_015901257.1	45106	45147	-	41

NW_015901274.1	62369	62497	-	128
NW_015901367.1	29438	29502	+	64
NW_015901515.1	86430	86759	-	329
NW_015901719.1	13729	13920	-	191
NW_015901722.1	31605	31752	+	147
NW_015901729.1	13916	14059	-	143
NW_015901739.1	54995	55087	-	92
NW_015901776.1	87507	87541	-	34
NW_015901793.1	53687	53826	-	139
NW_015901837.1	12821	12882	-	61
NW_015902209.1	41729	41801	+	72
NW_015902213.1	50661	50921	+	260
NW_015902217.1	41766	41889	-	123
NW_015902311.1	16007	16290	+	283
NW_015902319.1	56504	56759	-	255
NW_015902330.1	108553	108665	-	112
NW_015902345.1	135104	135307	+	203
NW_015902347.1	110648	111299	+	651
NW_015902436.1	65468	65613	+	125
NW_015902526.1	23411	23618	+	207
NW_015902592.1	29685	29796	-	111
NW_015902784.1	55378	55543	+	165
NW_015902845.1	2806	3101	+	295
NW_015902877.1	52062	52147	-	85
NW_015902905.1	77863	78122	+	259

NW_015902905.1	41461	41559	+	97
NW_015902988.1	49041	49132	-	91
NW_015903093.1	365	391	+	26
NW_015903115.1	84869	85003	-	80
NW_015903116.1	13776	13936	-	160
NW_015903179.1	30102	30341	-	239
NW_015903185.1	39844	39915	+	71
NW_015903238.1	67099	67257	+	158
NW_015903239.1	9590	9670	+	80
NW_015903242.1	34077	34150	+	73
NW_015903304.1	21973	22151	-	178
NW_015903342.1	17755	17856	-	101
NW_015903347.1	92317	92553	+	236
NW_015903404.1	37307	37471	-	164
NW_015903404.1	186186	186304	-	118
NW_015903464.1	24036	24059	+	23
NW_015903481.1	56484	56544	-	60
NW_015903497.1	29364	29518	-	154
NW_015903570.1	62667	62775	-	70
NW_015903570.1	19990	20111	+	121
NW_015903672.1	8258	8306	+	48
NW_015903716.1	57865	58168	+	303
NW_015903759.1	66990	67054	+	64
NW_015903759.1	67305	67429	+	124
NW_015903797.1	126747	126901	+	154

NW_015903853.1	48929	49012	+	83
NW_015903864.1	13478	13529	+	51
NW_015903892.1	183604	183908	+	304
NW_015903924.1	21607	21649	-	42
NW_015903976.1	36623	36723	-	100
NW_015904084.1	25537	25632	-	95
NW_015904084.1	37265	37341	-	76
NW_015904174.1	57814	57893	+	79
NW_015904216.1	166442	166850	-	408
NW_015904263.1	30923	30987	-	64
NW_015904287.1	26068	26081	+	13
NW_015904325.1	2320	2439	+	119
NW_015904349.1	26893	26979	+	86
NW_015904444.1	31944	32115	+	171
NW_015904444.1	31300	31480	+	180
NW_015904463.1	63358	63598	-	57
NW_015904532.1	73549	73682	+	94
NW_015904552.1	24841	25094	-	174
NW_015904552.1	24839	25109	-	189
NW_015904563.1	58085	58228	-	143
NW_015904563.1	57920	58148	-	228
NW_015904564.1	44438	44641	+	203
NW_015904568.1	23478	23574	-	96
NW_015904671.1	13818	13974	-	156
NW_015904699.1	63524	63858	-	109

NW_015904709.1	43832	43894	-	62
NW_015904717.1	69379	69528	-	149
NW_015904717.1	69264	69593	-	329
NW_015904717.1	36015	36070	+	55
NW_015904780.1	4439	4527	+	88
NW_015904798.1	234555	234584	-	29
NW_015904799.1	15229	15465	-	236
NW_015904799.1	15227	15485	-	258
NW_015904876.1	23294	23345	-	51
NW_015904934.1	34904	34986	+	82
NW_015904974.1	12811	12844	-	33
NW_015905071.1	36361	36634	+	273
NW_015905177.1	25731	25796	-	65
NW_015905204.1	39600	39647	+	47
NW_015905204.1	24962	25101	+	139
NW_015905228.1	48583	48678	-	95
NW_015905338.1	69256	69390	+	134
NW_015905343.1	17429	17516	+	87
NW_015905354.1	61978	62180	+	202
NW_015905354.1	61947	62152	+	205
NW_015905484.1	37036	37192	-	156
NW_015905548.1	12828	12952	-	124
NW_015905609.1	69868	69966	-	98
NW_015905674.1	15507	15599	+	92
NW_015905711.1	8241	8395	+	154

NW_015905711.1	8248	8421	+	173
NW_015905712.1	75480	75742	+	262
NW_015905785.1	2923	2996	+	73
NW_015905801.1	28218	28267	+	49
NW_015905802.1	56761	56858	+	97
NW_015905884.1	104677	104803	-	126
NW_015905901.1	30350	30427	-	77
NW_015905941.1	52876	53047	+	171
NW_015905989.1	14192	14231	+	39
NW_015906001.1	5481	5520	-	39
NW_015906017.1	160151	160266	+	115
NW_015906078.1	9871	10101	+	60
NW_015906117.1	5262	5348	+	86
NW_015906117.1	5727	5769	-	42
NW_015906215.1	42232	42274	-	42
NW_015906340.1	50255	50347	+	92
NW_015906375.1	95121	95386	+	265
NW_015906389.1	38403	38463	-	60
NW_015906389.1	38401	38483	-	82
NW_015906401.1	45531	45965	-	434
NW_015906465.1	31001	31112	-	111
NW_015906536.1	4700	4948	+	248
NW_015906548.1	124449	124523	-	74
NW_015906577.1	194525	194578	+	53
NW_015906621.1	125287	125393	+	106

NW_015906659.1	67742	67828	-	86
NW_015906659.1	81392	81478	-	61
NW_015906863.1	11398	11459	-	61
NW_015907009.1	2521	2558	-	37
NW_015907125.1	81619	81681	+	62
NW_015907179.1	64770	64904	+	134
NW_015907210.1	50078	50105	+	27
NW_015907282.1	58405	58446	-	41
NW_015907302.1	35520	35612	-	92
NW_015907369.1	50500	50671	-	171
NW_015907369.1	61159	61263	-	104
NW_015907450.1	4878	4921	-	43
NW_015907494.1	10194	10311	+	117
NW_015907496.1	24983	25307	+	324
NW_015907531.1	1662	1828	-	166
NW_015907531.1	2196	2554	-	358
NW_015907646.1	71933	72139	-	206
NW_015907664.1	21251	21292	+	41
NW_015907678.1	4858	4996	+	138
NW_015907696.1	22905	22994	-	89
NW_015907716.1	82778	82928	-	150
NW_015907768.1	48780	48866	+	86
NW_015907768.1	62030	62111	-	81
NW_015907819.1	37974	38117	+	143
NW_015907888.1	98178	98256	+	78

NW_015907898.1	3526	3608	+	82
NW_015907898.1	3513	3618	+	105
NW_015907994.1	9762	9861	-	99
NW_015908027.1	8239	8353	-	114
NW_015908050.1	37903	37967	+	64
NW_015908158.1	119940	120101	-	161
NW_015908240.1	685	720	+	35
NW_015908286.1	5518	5598	-	80
NW_015908347.1	44866	44991	+	125
NW_015908443.1	118577	118770	-	144
NW_015908522.1	3846	3881	+	35
NW_015908550.1	38115	38191	-	76
NW_015908554.1	7861	7908	+	47
NW_015908554.1	10552	10634	+	82
NW_015908554.1	7859	7926	+	67
NW_015908566.1	6255	6306	+	51
NW_015908580.1	29548	29633	+	43
NW_015908580.1	29419	29623	+	170
NW_015908589.1	94273	94335	+	62
NW_015908629.1	6248	6492	-	244
NW_015908648.1	25616	25847	-	231
NW_015908677.1	49360	49476	-	116
NW_015908684.1	5881	5945	-	64
NW_015908689.1	60124	60187	+	63
NW_015908714.1	38125	38227	+	102

NW_015908714.1	97447	97769	-	295
NW_015908758.1	26980	27067	-	87
NW_015908769.1	82941	83072	+	131
NW_015908838.1	89439	89480	-	41
NW_015908841.1	67552	67690	+	107
NW_015908843.1	25786	26205	+	419
NW_015908848.1	135678	135698	+	20
NW_015908924.1	33400	33509	-	18
NW_015908944.1	8309	8420	-	111
NW_015908944.1	8833	9004	-	171
NW_015908950.1	103405	103606	-	201
NW_015908995.1	185560	185596	-	36
NW_015908995.1	293057	293103	+	46
NW_015909012.1	3895	4082	-	120
NW_015909017.1	29919	29990	+	71
NW_015909017.1	19594	19681	+	87
NW_015909017.1	19588	19705	+	117
NW_015909017.1	29901	29999	+	98
NW_015909017.1	71121	71283	-	162
NW_015909017.1	97131	97195	+	64
NW_015909019.1	73704	73829	+	125
NW_015909022.1	6329	6421	-	67
NW_015909023.1	84884	85036	-	152
NW_015909096.1	15389	15463	+	74
NW_015909096.1	15397	15502	+	105

NW_015909096.1	24572	24727	-	155
NW_015909237.1	32576	32613	-	37
NW_015909244.1	8304	8465	+	85
NW_015909272.1	28449	28564	-	115
NW_015909272.1	28435	28823	-	388
NW_015909421.1	57686	57811	-	125
NW_015909562.1	28997	29129	-	132
NW_015909565.1	26249	26348	+	99
NW_015909610.1	13629	13817	+	188
NW_015909610.1	13665	13835	+	170
NW_015909627.1	53392	53557	-	165
NW_015909627.1	49408	49516	-	108
NW_015909909.1	106722	107028	+	306
NW_015909944.1	212643	212805	-	162
NW_015909954.1	3349	3540	-	155
NW_015909994.1	65466	65580	-	114
NW_015910036.1	16617	17237	-	620
NW_015910045.1	6129	6188	+	59
NW_015910150.1	15686	15799	+	113
NW_015910210.1	52589	52631	-	42
NW_015910226.1	177531	177552	+	21
NW_015910363.1	49192	49256	+	64
NW_015910399.1	11806	11870	-	64
NW_015910429.1	39290	39612	+	322
NW_015910429.1	133338	133486	+	148

NW_015910467.1	13260	13433	+	173
NW_015910518.1	52239	52402	-	163
NW_015910523.1	35042	35462	+	420
NW_015910536.1	13935	14113	-	178
NW_015910566.1	122426	122518	-	92
NW_015910566.1	122419	122554	-	135
NW_015910619.1	47966	48079	-	113
NW_015910710.1	31605	31670	-	65
NW_015910719.1	20070	20194	+	124
NW_015910852.1	11098	11395	+	297
NW_015910852.1	10866	11204	+	338
NW_015910852.1	11686	11992	+	306
NW_015910887.1	89238	89324	-	86
NW_015910939.1	159871	160005	+	77
NW_015910990.1	8914	9047	+	133
NW_015911029.1	27697	28031	-	334
NW_015911032.1	12767	12870	+	103
NW_015911183.1	15797	16118	+	321
NW_015911212.1	61985	62077	+	92
NW_015911271.1	70007	70186	+	179
NW_015911312.1	44596	44674	+	78
NW_015911340.1	28132	28246	+	114
NW_015911352.1	32128	32277	+	149
NW_015911468.1	24000	24081	+	81
NW_015911561.1	22781	22971	+	121

NW_015911561.1	51829	52016	-	187
NW_015911561.1	22781	22971	+	68
NW_015911627.1	53476	53565	+	89
NW_015911639.1	41054	41115	-	61
NW_015911639.1	41058	41117	-	59
NW_015911649.1	25674	25791	-	117
NW_015911677.1	44341	44462	-	121
NW_015911738.1	28484	28618	-	134
NW_015911745.1	12781	12880	-	99
NW_015911790.1	26695	26851	-	156
NW_015911817.1	19857	19977	-	120
NW_015911817.1	16294	16419	-	75
NW_015911857.1	32436	32571	-	135
NW_015911876.1	23618	23815	-	197
NW_015911940.1	4416	4473	-	57
NW_015911992.1	15644	15692	-	48
NW_015912002.1	96883	97001	-	118
NW_015912032.1	3566	3602	+	36
NW_015912050.1	37001	37326	+	325
NW_015912095.1	74918	75055	-	137
NW_015912097.1	67911	67983	-	72
NW_015912131.1	56864	56967	+	103
NW_015912224.1	7732	7903	+	171
NW_015912299.1	95774	95932	+	132
NW_015912317.1	17949	18061	-	112

NW_015912433.1	3502	3757	+	207
NW_015912461.1	62080	62147	-	67
NW_015912591.1	4807	5306	-	499
NW_015912607.1	51472	51575	+	103
NW_015912835.1	47805	47931	+	126
NW_015912852.1	88214	88265	-	29
NW_015912908.1	6825	6922	+	97
NW_015912913.1	5613	5751	-	138
NW_015912952.1	25528	25688	-	160
NW_015913302.1	21802	21857	+	46
NW_015913333.1	69865	69916	+	51
NW_015913333.1	64872	64985	+	113
NW_015913333.1	9110	9275	-	165
NW_015913361.1	47002	47105	-	103
NW_015913362.1	6334	6435	-	101
NW_015913480.1	4314	4978	-	67
NW_015913495.1	41618	41825	-	104
NW_015913501.1	3882	4069	-	187
NW_015913593.1	36659	36726	-	67
NW_015913702.1	88105	88334	+	229
NW_015913724.1	17894	18090	+	196
NW_015913725.1	38175	38522	+	347
NW_015913728.1	18337	18422	+	85
NW_015913738.1	30453	30550	-	97
NW_015913772.1	69407	69589	+	182

NW_015913811.1	67047	67199	-	152
NW_015913845.1	44636	44732	-	96
NW_015913846.1	12588	12667	-	79
NW_015913846.1	167698	167901	-	203
NW_015913856.1	60120	60226	+	106
NW_015913856.1	60102	60252	+	150
NW_015913909.1	60040	60139	+	99
NW_015913926.1	93001	93107	-	106
NW_015913926.1	92986	93104	-	118
NW_015913941.1	28221	28351	-	70
NW_015913941.1	28217	28360	-	79
NW_015913982.1	14548	14709	+	161
NW_015913982.1	14559	14760	+	201
NW_015914009.1	4745	4928	-	183
NW_015914025.1	37166	37245	+	79
NW_015914029.1	15705	15759	-	54
NW_015914045.1	14059	14202	-	143
NW_015914055.1	149211	149521	-	218
NW_015914106.1	2097	2189	+	92
NW_015914121.1	65551	65766	+	215
NW_015914121.1	65556	65781	+	225
NW_015914148.1	92833	92906	+	73
NW_015914336.1	8910	8964	-	54
NW_015914468.1	10134	10211	+	77
NW_015914479.1	66940	67014	+	74

NW_015914529.1	13740	13793	+	53
NW_015914701.1	32370	32429	+	59
NW_015914711.1	127238	127408	-	170
NW_015914758.1	128282	128321	-	39
NW_015914861.1	1473	1541	+	68
NW_015914974.1	7729	7975	-	246
NW_015914974.1	7707	8087	-	380
NW_015914996.1	43817	43965	-	148
NW_015915018.1	157394	157756	-	362
NW_015915042.1	22869	22978	+	109
NW_015915055.1	49645	49703	+	58
NW_015915070.1	14073	14176	+	103
NW_015915137.1	40399	40495	-	96
NW_015915292.1	35716	35898	+	182
NW_015915293.1	65267	65513	-	246
NW_015915412.1	11168	11189	-	21
NW_015915451.1	56551	56653	+	102
NW_015915476.1	35381	35440	+	59
NW_015915503.1	5948	6011	+	63
NW_015915503.1	5951	6013	+	62
NW_015915612.1	3787	3847	-	60
NW_015915645.1	76976	77142	-	166
NW_015915699.1	43805	43866	+	61
NW_015915705.1	96570	96761	+	113
NW_015915720.1	43738	44043	-	305

NW_015915722.1	68177	68348	-	171
NW_015915727.1	38439	38550	-	111
NW_015915772.1	90453	90544	+	91
NW_015915811.1	33774	34037	+	263
NW_015915819.1	131249	131393	+	144
NW_015916011.1	86972	87041	-	69
NW_015916059.1	56473	56881	+	408
NW_015916059.1	57142	57274	+	132
NW_015916072.1	31368	31515	-	147
NW_015916075.1	126518	126709	-	191
NW_015916130.1	32867	32917	-	50
NW_015916148.1	3230	3260	+	30
NW_015916170.1	21284	21384	-	100
NW_015916178.1	26251	26362	+	111
NW_015916178.1	86115	86467	-	352
NW_015916227.1	189646	189764	+	118
NW_015916273.1	32080	32243	+	163
NW_015916311.1	35711	35777	+	66
NW_015916319.1	55839	55989	+	150
NW_015916342.1	59614	59695	+	77
NW_015916356.1	39632	39695	+	63
NW_015916372.1	71639	71847	+	208
NW_015916372.1	72864	73047	+	183
NW_015916373.1	215102	215262	-	160
NW_015916373.1	206726	206832	-	106

NW_015916400.1	55796	55907	+	66
NW_015916407.1	10679	10790	-	111
NW_015916498.1	56591	56727	-	136
NW_015916499.1	34087	34188	-	101
NW_015916526.1	109070	109384	-	314
NW_015916546.1	15776	15914	+	138
NW_015916713.1	6692	6811	+	119
NW_015916713.1	32428	32527	-	99
NW_015916737.1	14286	14394	-	101
NW_015916753.1	8777	8959	-	182
NW_015916753.1	16472	16713	-	146
NW_015916964.1	23039	23107	+	68
NW_015916966.1	31006	31060	+	54
NW_015916998.1	65211	65326	-	115
NW_015917019.1	33348	33496	-	148
NW_015917026.1	31750	31825	-	75
NW_015917079.1	23417	23515	+	98
NW_015917081.1	38052	38167	+	115
NW_015917182.1	33068	33202	+	116
NW_015917211.1	90596	90718	+	122
NW_015917319.1	11043	11163	+	120
NW_015917424.1	37182	37257	+	71
NW_015917434.1	46207	46294	+	87
NW_015917434.1	46159	46330	+	171
NW_015917466.1	7524	7682	+	158

NW_015917480.1	27266	27348	-	82
NW_015917517.1	48914	49083	+	169
NW_015917544.1	7702	7777	+	75
NW_015917721.1	154275	154435	-	160
NW_015917737.1	23801	24162	-	361
NW_015917760.1	63303	63336	+	33
NW_015917781.1	35218	35474	-	256
NW_015917798.1	47638	47808	-	92
NW_015917820.1	100404	100620	-	216
NW_015917988.1	131803	132049	-	246
NW_015918003.1	32336	32420	+	84
NW_015918015.1	33140	33228	+	88
NW_015918229.1	18863	18955	-	92
NW_015918253.1	86855	86951	-	96
NW_015918329.1	64462	64535	-	52
NW_015918332.1	72342	72694	+	290
NW_015918335.1	6758	7037	+	279
NW_015918365.1	11839	12040	-	201
NW_015918388.1	67976	68148	-	172
NW_015918466.1	19518	19641	+	123
NW_015918466.1	19446	19642	+	196
NW_015918466.1	42963	43091	-	128
NW_015918523.1	16113	16723	-	610
NW_015918573.1	4633	4775	-	142
NW_015918609.1	38701	38833	-	125

NW_015918663.1	21159	21429	-	270
NW_015918718.1	71723	71762	+	39
NW_015918723.1	11734	11830	-	96
NW_015918900.1	75210	75268	-	58
NW_015918909.1	247838	247925	+	87
NW_015918920.1	15977	16131	-	154
NW_015919061.1	78160	78276	+	116
NW_015919075.1	19327	19450	-	107
NW_015919160.1	2572	2662	-	90
NW_015919160.1	20805	21015	-	210
NW_015919185.1	174499	174552	+	53
NW_015919220.1	66670	66749	-	79
NW_015919290.1	20318	20616	+	298
NW_015919292.1	77360	77498	-	138
NW_015919454.1	38189	38272	+	60
NW_015919564.1	32765	32927	+	162
NW_015919589.1	16152	16266	-	114
NW_015919603.1	92081	92199	-	118
NW_015919648.1	61174	61326	+	152
NW_015919660.1	48948	49060	-	112
NW_015919696.1	14759	14834	+	75
NW_015919730.1	3466	3625	-	159
NW_015919748.1	16009	16102	-	93
NW_015919764.1	22652	22752	-	100
NW_015919868.1	93004	93152	+	148

NW_015919885.1	7243	7430	-	187
NW_015919953.1	30460	30515	+	55
NW_015919954.1	47172	47634	-	462
NW_015919964.1	67137	67296	+	159
NW_015919969.1	18241	18379	+	138
NW_015919999.1	24367	24571	+	204
NW_015920044.1	106822	106909	+	87
NW_015920065.1	2710	2953	-	243
NW_015920065.1	5118	5275	+	157
NW_015920069.1	5821	5935	+	114
NW_015920142.1	56369	56504	+	135
NW_015920281.1	13636	13692	+	56
NW_015920442.1	49662	49729	-	67
NW_015920493.1	42450	42552	-	102
NW_015920557.1	6363	6419	+	56
NW_015920827.1	143949	144062	-	113
NW_015920827.1	143942	144186	-	244
NW_015920834.1	63409	63532	+	100
NW_015920870.1	8694	8748	+	47
NW_015920870.1	22532	22710	+	178
NW_015920910.1	37808	37960	+	112
NW_015920924.1	5450	5548	-	98
NW_015921066.1	22801	22957	-	156
NW_015921077.1	109307	109400	-	93
NW_015921142.1	26032	26124	+	92

NW_015921164.1	4565	4793	+	228
NW_015921253.1	13040	13130	+	90
NW_015921297.1	17443	17482	-	39
NW_015921366.1	29197	29329	-	132
NW_015921430.1	77967	78091	+	124
NW_015921443.1	15265	15344	+	79
NW_015921443.1	15260	15416	+	156
NW_015921449.1	26280	26368	-	88
NW_015921449.1	41536	41620	-	84
NW_015921452.1	4396	4504	+	108
NW_015921484.1	8339	8529	+	190
NW_015921484.1	8788	8823	+	35
NW_015921526.1	29576	29663	-	61
NW_015921526.1	29513	29677	-	75
NW_015921646.1	123930	124293	-	363
NW_015921660.1	52535	52623	-	88
NW_015921739.1	3103	3162	+	59
NW_015921743.1	177730	177799	+	69
NW_015921743.1	188788	188960	-	117
NW_015921757.1	39863	39961	+	98
NW_015921758.1	17658	17729	-	71
NW_015921780.1	23292	23346	-	54
NW_015921829.1	20702	20907	-	205
NW_015921874.1	14439	14625	-	186
NW_015921901.1	99337	99503	-	166

NW_015921961.1	5749	5816	-	67
NW_015922001.1	21991	22085	+	94
NW_015922001.1	32844	32961	-	117
NW_015922048.1	20439	20577	+	138
NW_015922079.1	11421	11481	+	60
NW_015922079.1	2338	2630	+	49
NW_015922079.1	11413	11501	+	88
NW_015922165.1	8071	8164	-	93
NW_015922166.1	27969	28059	-	90
NW_015922182.1	261630	261934	-	304
NW_015922183.1	80495	80662	-	167
NW_015922253.1	4354	4732	+	378
NW_015922253.1	37156	37339	+	183
NW_015922253.1	52696	52785	+	89
NW_015922307.1	59834	59987	+	153
NW_015922346.1	17042	17093	+	51
NW_015922412.1	34109	34182	+	73
NW_015922419.1	36704	36838	+	49
NW_015922419.1	51347	51439	-	92
NW_015922458.1	47458	47502	-	44
NW_015922458.1	47475	47508	-	33
NW_015922467.1	21937	22105	+	168
NW_015922467.1	21925	22011	+	86
NW_015922480.1	18402	18455	-	53
NW_015922523.1	13838	13897	+	59

NW_015922590.1	66641	66773	-	132
NW_015922638.1	6887	7061	-	174
NW_015922679.1	16439	16610	+	171
NW_015922693.1	254950	255063	+	113
NW_015922800.1	12744	13036	-	292
NW_015922835.1	74952	74982	-	30
NW_015922866.1	30825	30922	+	97
NW_015922866.1	30766	30927	+	161
NW_015922869.1	20224	20253	-	29
NW_015922992.1	14004	14132	-	128
NW_015923089.1	1686	1750	+	64
NW_015923102.1	84491	84556	-	65
NW_015923156.1	245874	246033	-	159
NW_015923273.1	4922	4956	-	34
NW_015923293.1	28983	29184	-	201
NW_015923297.1	8503	8657	-	154
NW_015923350.1	7447	7530	+	83
NW_015923358.1	39940	40006	+	66
NW_015923454.1	43600	44042	+	442
NW_015923456.1	24305	24368	+	63
NW_015923456.1	59787	59892	-	52
NW_015923468.1	13450	13639	-	189
NW_015923552.1	42694	42755	+	36
NW_015923562.1	27263	27332	-	69
NW_015923671.1	26974	27068	+	94

NW_015923671.1	24332	24393	+	61
NW_015923706.1	2300	2449	+	149
NW_015923717.1	45465	45667	-	202
NW_015923849.1	22791	22865	-	74
NW_015923882.1	22344	22440	-	96
NW_015924019.1	9451	9558	-	71
NW_015924026.1	5421	5602	-	181
NW_015924032.1	14455	14633	-	178
NW_015924067.1	90134	90338	-	204
NW_015924203.1	13428	13541	+	113
NW_015924266.1	32975	33163	+	188
NW_015924283.1	136792	136906	+	114
NW_015924338.1	3746	3854	-	108
NW_015924571.1	2551	2578	+	27
NW_015924609.1	101	432	+	331
NW_015924670.1	80545	80727	-	182
NW_015924692.1	4449	4532	+	83
NW_015924718.1	33387	33583	+	196
NW_015924743.1	69540	69741	-	201
NW_015924749.1	41430	41500	+	70
NW_015924776.1	15767	15896	+	129
NW_015924789.1	60043	60196	-	153
NW_015924819.1	30895	31086	+	191
NW_015924824.1	62821	62913	+	92
NW_015924910.1	40692	40735	+	43

NW_015924983.1	14842	15027	+	185
NW_015924987.1	29769	29815	+	46
NW_015924987.1	29781	29814	+	33
NW_015924987.1	29767	29824	+	57
NW_015925131.1	22579	22692	-	113
NW_015925145.1	73710	73878	-	168
NW_015925192.1	6241	6624	+	383
NW_015925203.1	3730	4055	-	325
NW_015925224.1	21581	21639	+	58
NW_015925290.1	83979	84113	+	134
NW_015925345.1	110750	110943	-	71
NW_015925440.1	79002	79096	-	94
NW_015925463.1	224270	224334	-	64
NW_015925504.1	63941	64008	+	67
NW_015925570.1	87448	87501	+	53
NW_015925600.1	7248	7328	-	80
NW_015925608.1	60389	60625	+	236
NW_015925614.1	119326	119493	+	167
NW_015925619.1	23604	23706	+	102
NW_015925634.1	233770	233901	+	131
NW_015925709.1	9488	9537	+	49
NW_015925709.1	9449	9518	+	69
NW_015925709.1	51008	51387	+	379
NW_015925709.1	9374	9551	+	177
NW_015925734.1	51241	51433	-	115

NW_015925760.1	18769	18912	+	143
NW_015925777.1	72114	72211	-	97
NW_015925781.1	53456	53556	+	100
NW_015925819.1	17021	17159	-	138
NW_015925906.1	16667	16709	+	42
NW_015925982.1	20613	20783	-	170
NW_015926060.1	12388	12523	-	117
NW_015926091.1	22542	22748	+	206
NW_015926155.1	12066	12157	-	91
NW_015926186.1	28927	29122	-	118
NW_015926256.1	14041	14155	-	114
NW_015926256.1	37244	37377	+	133
NW_015926258.1	10845	11007	+	162
NW_015926271.1	15343	15452	+	109
NW_015926301.1	50271	50328	-	57
NW_015926317.1	11770	11849	-	79
NW_015926495.1	341081	341122	-	41
NW_015926539.1	16624	16774	+	150
NW_015926850.1	58603	58765	+	115
NW_015926850.1	58605	58675	+	70
NW_015926895.1	1892	2030	+	134
NW_015926911.1	26231	26301	-	70
NW_015926944.1	64684	64951	+	267
NW_015926945.1	50459	50537	-	78
NW_015927047.1	12632	12761	-	129

NW_015927089.1	9388	9640	+	20
NW_015927091.1	34225	34323	+	98
NW_015927176.1	2989	3177	+	188
NW_015927180.1	4767	5017	+	250
NW_015927253.1	22181	22458	+	277
NW_015927253.1	23133	23283	+	150
NW_015927299.1	12254	12421	-	167
NW_015927404.1	91016	91103	+	87
NW_015927546.1	16816	16939	-	123
NW_015927745.1	23712	23885	+	99
NW_015927863.1	52959	53090	-	131
NW_015927898.1	44927	45086	-	159
NW_015928099.1	40493	40589	-	96
NW_015928099.1	32250	32376	+	126
NW_015928128.1	56377	56468	+	91
NW_015928128.1	56403	56502	+	99
NW_015928145.1	16735	16802	+	67
NW_015928200.1	11068	11280	+	212
NW_015928200.1	11031	11340	-	309
NW_015928200.1	5222	5379	+	157
NW_015928368.1	2938	3078	+	140
NW_015928423.1	11944	12005	+	61
NW_015928548.1	44205	44319	-	114
NW_015928560.1	103853	103961	-	108
NW_015928592.1	50819	50907	+	88

NW_015928732.1	91642	91752	-	110
NW_015928739.1	26586	26750	-	164
NW_015928944.1	29888	29953	+	65
NW_015928944.1	29881	29974	+	93
NW_015929051.1	120482	120564	-	82
NW_015929181.1	5974	6203	+	229
NW_015929226.1	26881	27272	-	391
NW_015929283.1	8115	8243	+	86
NW_015929432.1	5876	5918	+	42
NW_015929603.1	84441	84488	+	47
NW_015929630.1	66361	66461	+	100
NW_015929642.1	25012	25171	+	159
NW_015929858.1	108181	108338	-	157
NW_015929871.1	23750	23817	+	67
NW_015929894.1	18105	18364	+	259
NW_015929942.1	66771	66829	+	58
NW_015929960.1	15451	15503	-	52
NW_015930033.1	56774	56866	+	92
NW_015930042.1	32614	32768	+	154
NW_015930226.1	34765	34842	-	77
NW_015930343.1	12405	12508	+	103
NW_015930447.1	14225	14480	+	255
NW_015930491.1	40435	40566	-	131
NW_015930491.1	24381	24632	+	251
NW_015930609.1	13612	13699	+	63

NW_015930843.1	21169	21300	+	131
NW_015930915.1	48208	48347	+	139
NW_015930921.1	86390	86442	-	52
NW_015931044.1	39279	39366	+	87
NW_015931076.1	56499	56589	+	90
NW_015931221.1	32643	32696	+	53
NW_015931255.1	49848	49938	+	90
NW_015931375.1	27575	27633	+	58
NW_015931393.1	70834	70854	+	20
NW_015931416.1	73815	73968	+	153
NW_015931532.1	26527	26652	-	125
NW_015931583.1	49234	49362	+	79
NW_015931628.1	57051	57167	+	116
NW_015931690.1	7793	7818	+	25
NW_015931842.1	5238	5451	-	213
NW_015931866.1	44401	44592	-	191
NW_015931892.1	26337	26474	-	137
NW_015931929.1	2636	2757	+	121
NW_015931959.1	17828	17880	+	52
NW_015932008.1	29534	29630	+	96
NW_015932029.1	18431	18576	-	145
NW_015932131.1	45776	46000	-	224
NW_015932141.1	34336	34460	+	124
NW_015932185.1	14917	14956	-	39
NW_015932412.1	5253	5300	+	47

NW_015932412.1	33628	33728	+	100
NW_015932445.1	13076	13096	-	20
NW_015932470.1	46391	46587	-	164
NW_015932494.1	29174	29266	+	92
NW_015932520.1	100	177	+	77
NW_015932557.1	24625	24745	+	120
NW_015932609.1	26678	26824	-	146
NW_015932637.1	19625	19950	+	325
NW_015932660.1	20090	20152	+	62
NW_015932691.1	46581	46728	+	147
NW_015932742.1	34923	35074	+	151
NW_015932765.1	8261	8284	+	23
NW_015932842.1	17891	17959	-	63
NW_015932885.1	89626	89703	-	77
NW_015932988.1	44906	44957	+	51
NW_015933041.1	23110	23159	-	49
NW_015933163.1	100546	100586	+	40
NW_015933163.1	100543	100594	+	51
NW_015933189.1	18955	19091	+	91
NW_015933252.1	15608	15809	-	201
NW_015933253.1	2181	2328	+	147
NW_015933280.1	169680	169993	-	313
NW_015933282.1	51936	52123	+	187
NW_015933353.1	6441	6599	+	158
NW_015933371.1	9131	9669	+	538

NW_015933432.1	114100	114150	+	50
NW_015933463.1	55140	55328	+	188
NW_015933568.1	7976	8035	+	59
NW_015933568.1	7970	8031	+	61
NW_015933631.1	27965	28091	-	126
NW_015933680.1	9236	9265	+	29
NW_015933743.1	39036	39066	-	18
NW_015933762.1	8121	8279	+	158
NW_015933796.1	8757	8907	+	150
NW_015933796.1	8774	8874	+	100
NW_015933796.1	8738	8917	+	179
NW_015933808.1	30873	30945	+	72
NW_015933840.1	114654	114780	-	126
NW_015933846.1	89941	89954	-	13
NW_015933874.1	57357	57499	-	142
NW_015933996.1	3440	3564	+	124
NW_015934082.1	76786	76928	+	142
NW_015934123.1	74829	74911	-	82
NW_015934273.1	5793	5928	+	135
NW_015934288.1	93053	93113	-	60
NW_015934384.1	14470	14600	+	130
NW_015934407.1	1117	1179	+	62
NW_015934651.1	12445	12546	-	101
NW_015934726.1	126577	126697	+	120
NW_015934728.1	45686	45801	-	115

NW_015934781.1	24015	24174	+	159
NW_015934794.1	45444	45554	+	71
NW_015934794.1	44995	45161	+	78
NW_015934893.1	39695	39742	-	47
NW_015934914.1	42969	43104	+	135
NW_015934933.1	121485	121568	+	83
NW_015934996.1	23747	23778	-	31
NW_015934999.1	1123	1192	-	69
NW_015935085.1	48408	48491	+	83
NW_015935087.1	36341	36677	-	336
NW_015935138.1	40602	40676	+	74
NW_015935321.1	17454	17612	+	158
NW_015935532.1	166740	166808	+	68
NW_015935554.1	15293	15468	+	99
NW_015935561.1	136308	136450	+	142
NW_015935619.1	5678	5988	-	310
NW_015935659.1	4681	4934	-	253
NW_015935691.1	80294	80401	+	107
NW_015935769.1	33281	33466	-	185
NW_015935769.1	36641	36871	-	159
NW_015935920.1	21690	21801	+	111
NW_015935920.1	21676	21792	+	116
NW_015935936.1	36300	36416	+	116
NW_015935988.1	21506	21654	+	148
NW_015935988.1	20935	21075	+	140

NW_015936089.1	49448	49575	+	127
NW_015936163.1	88138	88183	-	45
NW_015936241.1	46709	46904	+	195
NW_015936352.1	24384	24464	-	80
NW_015936593.1	25825	25938	+	113
NW_015936598.1	37918	38096	+	178
NW_015936776.1	44141	44253	+	112
NW_015936852.1	66669	66713	-	44
NW_015936986.1	29432	29589	-	157
NW_015936987.1	47906	47958	+	52
NW_015936988.1	11969	12101	-	87
NW_015937130.1	10472	10515	+	43
NW_015937130.1	10475	10516	+	41
NW_015937464.1	39789	39885	-	96
NW_015937585.1	13949	14028	-	79
NW_015937585.1	89203	89276	-	73
NW_015937666.1	8887	9020	+	133
NW_015937723.1	14404	14472	-	68
NW_015937776.1	28595	28692	-	97
NW_015937850.1	2616	2904	-	288
NW_015937850.1	20476	20685	-	209
NW_015937880.1	33530	33599	+	69
NW_015937880.1	52839	52983	+	144
NW_015937916.1	44066	44181	-	73
NW_015937940.1	6421	6591	-	170

NW_015937951.1	7428	7480	-	52
NW_015938041.1	12760	12869	-	109
NW_015938041.1	5441	5654	-	213
NW_015938045.1	8424	8542	-	118
NW_015938045.1	8445	8596	-	151
NW_015938063.1	8766	8829	-	63
NW_015938091.1	89531	89625	+	94
NW_015938092.1	29889	29950	+	61
NW_015938092.1	29907	29954	+	47
NW_015938092.1	29880	29960	+	80
NW_015938099.1	2805	3017	-	212
NW_015938099.1	2829	3022	-	193
NW_015938182.1	16892	16984	+	92
NW_015938192.1	49162	49256	+	94
NW_015938303.1	39490	39567	-	77
NW_015938512.1	47527	47627	+	100
NW_015938529.1	135816	135884	+	68
NW_015938557.1	38510	38698	-	188
NW_015938613.1	16114	16298	-	184
NW_015938708.1	46682	46722	+	40
NW_015938719.1	11191	11360	-	147
NW_015938729.1	10738	10883	+	145
NW_015938745.1	13590	13773	+	183
NW_015938751.1	62649	62918	-	269
NW_015938751.1	59599	59686	-	87

NW_015938751.1	62613	62931	-	318
NW_015938902.1	15061	15269	+	208
NW_015938902.1	13939	14016	+	77
NW_015938930.1	83080	83183	+	103
NW_015938958.1	57087	57300	+	213
NW_015938964.1	17120	17304	-	110
NW_015938979.1	36447	36605	+	158
NW_015939057.1	38665	38758	-	93
NW_015939093.1	15758	15841	-	83
NW_015939209.1	32286	32408	+	122
NW_015939239.1	8823	9073	-	250
NW_015939246.1	14149	14265	-	116
NW_015939353.1	42403	42457	-	54
NW_015939676.1	13048	13156	-	108
NW_015939915.1	27937	28006	-	69
NW_015940113.1	51673	51832	+	159
NW_015940116.1	124650	124875	-	225
NW_015940146.1	37339	37535	-	196
NW_015940157.1	19516	19632	+	116
NW_015940167.1	29049	29073	-	24
NW_015940213.1	41695	41830	+	135
NW_015940213.1	34482	34630	+	148
NW_015940220.1	2216	2406	+	74
NW_015940481.1	24963	25238	+	275
NW_015940540.1	13988	14095	+	107

NW_015940548.1	30255	30417	+	162
NW_015940632.1	156926	157109	+	183
NW_015940635.1	7702	7777	-	75
NW_015940712.1	42356	42463	-	107
NW_015940826.1	32637	32708	-	71
NW_015940858.1	30200	30327	+	127
NW_015940858.1	30163	30317	+	154
NW_015940961.1	6679	6841	-	162
NW_015940969.1	33740	33757	+	17
NW_015940996.1	38892	39111	+	219
NW_015941027.1	2450	2518	-	68
NW_015941041.1	2155	2229	+	74
NW_015941118.1	45399	45560	+	161
NW_015941126.1	259268	259414	+	146
NW_015941143.1	22998	23089	-	91
NW_015941143.1	23016	23123	-	107
NW_015941144.1	2512	2604	+	92
NW_015941377.1	5261	5395	+	134
NW_015941377.1	5227	5410	+	183
NW_015941486.1	6587	6674	+	87
NW_015941516.1	63947	64009	+	62
NW_015941552.1	29315	29390	-	75
NW_015941628.1	1654	1754	+	100
NW_015941741.1	56582	56625	-	43
NW_015941788.1	3519	3619	+	100

NW_015941847.1	7535	7755	+	220
NW_015942133.1	36893	37097	+	204
NW_015942170.1	16214	16278	+	64
NW_015942170.1	19901	19981	+	80
NW_015942260.1	15765	15862	+	97
NW_015942299.1	29924	30033	-	109
NW_015942306.1	5034	5108	-	74
NW_015942395.1	65893	66063	-	170
NW_015942468.1	6450	6593	-	76
NW_015942531.1	32431	32504	-	73
NW_015942531.1	45844	46142	-	298
NW_015942546.1	90	158	+	68
NW_015942759.1	22395	22491	+	96
NW_015942792.1	22575	22662	+	87
NW_015942820.1	32228	32326	-	98
NW_015942850.1	18395	18547	-	101
NW_015942886.1	12568	12829	+	261
NW_015942910.1	37544	37616	+	72
NW_015943089.1	26523	26667	-	144
NW_015943099.1	31316	31631	+	315
NW_015943136.1	12893	13087	-	194
NW_015943161.1	38021	38080	-	59
NW_015943181.1	10288	10524	-	236
NW_015943185.1	60321	60471	+	150
NW_015943230.1	72397	72551	-	154

NW_015943233.1	6323	6438	-	113
NW_015943245.1	35185	35419	+	234
NW_015943283.1	50920	51014	+	67
NW_015943317.1	27407	27424	-	17
NW_015943319.1	22612	22855	+	243
NW_015943341.1	6160	6325	+	165
NW_015943355.1	3371	4025	+	654
NW_015943368.1	36951	37016	-	65
NW_015943534.1	48552	48620	-	68
NW_015943586.1	36458	36615	+	157
NW_015943606.1	13333	13422	-	89
NW_015943790.1	5822	6014	-	176
NW_015943812.1	2700	3066	+	366
NW_015943877.1	15959	16099	+	140
NW_015943915.1	88774	88828	+	54
NW_015943983.1	42903	43012	+	109
NW_015944017.1	19229	19352	-	123
NW_015944054.1	4087	4284	+	197
NW_015944096.1	27982	28273	-	291
NW_015944131.1	63756	63893	+	137
NW_015944148.1	98481	98601	-	120
NW_015944250.1	29547	29682	+	135
NW_015944258.1	19214	19609	+	395
NW_015944309.1	12800	12935	-	135
NW_015944350.1	16707	16766	-	59

NW_015944521.1	18018	18134	+	112
NW_015944565.1	59529	59643	-	114
NW_015944631.1	7442	7622	+	180
NW_015944632.1	12141	12285	-	109
NW_015944650.1	30062	30225	-	163
NW_015944696.1	145794	145964	-	170
NW_015944710.1	109157	109262	-	105
NW_015944710.1	26719	26951	-	200
NW_015944763.1	45385	45528	-	143
NW_015944851.1	13033	13200	+	167
NW_015944851.1	12958	13183	+	225
NW_015944917.1	56170	56307	+	137
NW_015945086.1	50556	50793	+	237
NW_015945158.1	188652	188837	-	185
NW_015945193.1	6593	6696	+	103
NW_015945193.1	6575	6727	+	152
NW_015945193.1	13168	13358	+	190
NW_015945330.1	9838	10011	-	81
NW_015945335.1	7612	7652	-	40
NW_015945359.1	127935	128092	-	157
NW_015945363.1	7190	7341	-	115
NW_015945414.1	32547	32704	-	108
NW_015945564.1	14917	15029	-	112
NW_015945587.1	49295	49392	-	97
NW_015945818.1	2367	2559	+	192

NW_015945958.1	75518	75626	-	100
NW_015945995.1	5188	5263	+	75
NW_015946040.1	115952	116065	-	113
NW_015946110.1	31519	31739	+	220
NW_015946167.1	51906	52185	+	279
NW_015946218.1	3493	3679	+	186
NW_015946357.1	2175	2503	-	328
NW_015946492.1	7330	7493	+	163
NW_015946496.1	2306	2462	-	156
NW_015946496.1	2104	2367	-	263
NW_015946521.1	6138	6251	+	113
NW_015946604.1	2367	2532	-	110
NW_015946678.1	38351	38380	+	29
NW_015946740.1	13431	13503	-	72
NW_015946810.1	6397	6558	-	161
NW_015946810.1	6404	6515	-	111
NW_015946810.1	6389	6533	-	144
NW_015946812.1	73240	73357	-	117
NW_015946812.1	73258	73378	-	120
NW_015946934.1	41151	41175	+	24
NW_015946985.1	28280	28454	+	174
NW_015946993.1	52330	52492	-	78
NW_015947005.1	26128	26394	-	266
NW_015947080.1	25737	25785	+	48
NW_015947306.1	34680	34801	-	121

NW_015947342.1	11810	11944	+	134
NW_015947344.1	7572	7672	-	100
NW_015947391.1	23510	23681	+	171
NW_015947416.1	21290	21332	-	42
NW_015947578.1	4027	4201	-	174
NW_015947630.1	71354	71484	+	130
NW_015947637.1	21274	21422	+	148
NW_015947703.1	82724	82751	+	27
NW_015947703.1	83157	83236	+	79
NW_015947764.1	29995	30124	-	88
NW_015947801.1	24778	24859	-	81
NW_015947834.1	75513	75616	-	103
NW_015947850.1	7442	7495	-	53
NW_015948067.1	17479	17740	+	261
NW_015948156.1	38575	38687	+	112
NW_015948222.1	28757	28918	-	161
NW_015948283.1	7034	7102	+	68
NW_015948422.1	17946	18113	-	167
NW_015948425.1	109012	109274	-	262
NW_015948504.1	7254	7374	+	120
NW_015948504.1	39086	39177	-	87
NW_015948562.1	13768	13989	-	221
NW_015948576.1	4652	4749	-	97
NW_015948690.1	3905	4060	-	113
NW_015948828.1	25788	25861	+	73

NW_015948831.1	7870	7901	+	31
NW_015948933.1	27809	27911	-	102
NW_015948933.1	27787	27909	-	122
NW_015949087.1	46369	46464	+	95
NW_015949113.1	21182	21386	+	204
NW_015949307.1	7293	7319	-	26
NW_015949353.1	3629	3781	-	152
NW_015949412.1	19007	19030	+	23
NW_015949412.1	18984	19048	+	64
NW_015949414.1	14712	14835	-	123
NW_015949460.1	29060	29243	-	183
NW_015949554.1	1693	1814	+	121
NW_015949603.1	15810	15844	+	34
NW_015949618.1	37935	37974	+	39
NW_015949622.1	45022	45188	+	143
NW_015949695.1	5862	5941	-	79
NW_015949877.1	105874	105932	+	58
NW_015949979.1	15281	15388	+	107
NW_015950109.1	1271	1417	-	146
NW_015950202.1	68493	68614	-	81
NW_015950368.1	54535	54596	-	61
NW_015950374.1	43184	43283	+	99
NW_015950377.1	27302	27346	+	44
NW_015950559.1	36644	36724	+	80
NW_015950776.1	7003	7065	+	62

NW_015950798.1	25394	25486	+	92
NW_015950799.1	27046	27207	-	161
NW_015950934.1	11443	11507	+	64
NW_015950934.1	74387	74607	-	220
NW_015951006.1	5391	5623	+	232
NW_015951020.1	2421	2505	-	84
NW_015951026.1	51724	51948	-	224
NW_015951026.1	51774	51955	-	181
NW_015951026.1	51694	51987	-	293
NW_015951085.1	16582	16678	-	80
NW_015951196.1	154964	155062	+	98
NW_015951222.1	170219	170373	-	154
NW_015951538.1	128966	129082	+	72
NW_015951599.1	21422	21493	-	71
NW_015951690.1	12301	12450	+	149
NW_015951788.1	16734	16948	-	142
NW_015951933.1	69324	69384	-	60
NW_015951945.1	78436	78610	-	100
NW_015952097.1	39533	39628	+	95
NW_015952222.1	6883	6974	+	91
NW_015952235.1	22809	23000	-	191
NW_015952300.1	7764	7887	+	123
NW_015952325.1	7928	8047	+	119
NW_015952433.1	35826	35948	+	122
NW_015952513.1	35780	35913	-	133

NW_015952580.1	42799	42965	+	166
NW_015952624.1	161720	161777	+	57
NW_015952863.1	19145	19483	+	250
NW_015952863.1	19393	19491	+	98
NW_015952863.1	19346	19488	+	142
NW_015952888.1	17572	17668	-	96
NW_015952919.1	10040	10341	-	301
NW_015952919.1	10041	10360	-	319
NW_015953096.1	7657	7749	+	92
NW_015953099.1	73079	73124	-	45
NW_015953242.1	30468	30619	-	151
NW_015953249.1	70156	70373	+	217
NW_015953319.1	16267	16423	-	156
NW_015953504.1	88958	89344	-	386
NW_015953557.1	6656	6764	+	90
NW_015953585.1	22077	22231	+	154
NW_015953746.1	19706	20163	-	457
NW_015953746.1	19698	20347	-	649
NW_015953755.1	7914	8080	-	166
NW_015953831.1	16107	16279	-	172
NW_015953833.1	30786	30980	+	98
NW_015953877.1	24445	24522	-	77
NW_015953888.1	51673	51874	-	201
NW_015953907.1	180872	181074	+	202
NW_015953907.1	125913	125964	+	51

NW_015954099.1	26012	26151	+	139
NW_015954264.1	60716	60906	+	190
NW_015954267.1	41314	41401	-	87
NW_015954368.1	66711	66769	+	58
NW_015954448.1	24632	24683	-	51
NW_015954473.1	42900	42928	+	28
NW_015954522.1	18013	18174	-	161
NW_015954584.1	12720	13194	+	474
NW_015954668.1	14249	14305	-	56
NW_015954748.1	4679	4753	+	74
NW_015954809.1	48145	48207	-	62
NW_015954833.1	8767	9140	-	373
NW_015954901.1	21798	22028	-	93
NW_015955055.1	38122	38258	+	136
NW_015955103.1	60723	60825	+	102
NW_015955234.1	37655	37760	-	105

13days post inoculation: recovery site (Hyper-DMRs)

Scaffold	Start	End	Region stain	Overlapped site
NW_015787570.1	5166	5300	-	134
NW_015787570.1	5102	5245	-	143
NW_015787570.1	5088	5351	-	263
NW_015787709.1	12799	12856	+	57
NW_015787709.1	12802	12867	+	61

NW_015787756.1	29600	29637	-	37
NW_015788036.1	66948	67090	+	142
NW_015789314.1	46578	46787	+	209
NW_015789314.1	46566	46838	+	253
NW_015789335.1	80139	80253	+	114
NW_015789335.1	80067	80240	+	173
NW_015789913.1	41684	41744	-	60
NW_015789913.1	41478	41737	-	259
NW_015789913.1	41454	41734	-	280
NW_015791475.1	11619	11668	-	49
NW_015791475.1	11620	11661	-	41
NW_015791980.1	46616	46794	+	178
NW_015792254.1	14170	14199	-	29
NW_015792603.1	17283	17404	+	121
NW_015794929.1	7542	7638	-	96
NW_015795042.1	12026	12193	+	167
NW_015795225.1	33677	33779	+	102
NW_015795290.1	87917	88026	-	109
NW_015795761.1	40542	40673	+	131
NW_015796444.1	46413	46562	-	149
NW_015796444.1	46346	46565	-	219
NW_015797040.1	17994	18159	+	165
NW_015797568.1	51223	51244	-	21
NW_015797643.1	16047	16080	+	33
NW_015797899.1	59829	59866	+	37

NW_015798053.1	6638	6734	+	96
NW_015798323.1	6343	6443	+	100
NW_015798323.1	6339	6461	+	122
NW_015798390.1	31564	31706	-	142
NW_015798390.1	31599	31707	-	108
NW_015798390.1	31569	31704	-	135
NW_015799056.1	48392	48558	-	166
NW_015799227.1	6330	6637	+	307
NW_015799227.1	6483	6601	+	118
NW_015799227.1	6318	6691	+	373
NW_015799362.1	88482	88588	-	106
NW_015799362.1	88477	88585	-	108
NW_015800713.1	22009	22100	+	91
NW_015802527.1	12556	12586	-	30
NW_015802928.1	32854	33102	+	248
NW_015803487.1	3609	3678	+	69
NW_015803487.1	3587	3690	+	103
NW_015803487.1	3591	3693	+	102
NW_015804735.1	53584	53676	+	92
NW_015805172.1	199226	199329	+	83
NW_015805359.1	11360	11436	-	76
NW_015806601.1	104048	104079	+	31
NW_015806601.1	104037	104087	+	50
NW_015806601.1	104242	104256	+	14
NW_015807358.1	31037	31088	+	5

NW_015808069.1	86998	87076	-	78
NW_015808204.1	19601	19676	-	75
NW_015809391.1	14657	14728	-	71
NW_015809415.1	4281	4326	+	45
NW_015809415.1	4279	4325	+	46
NW_015809459.1	26458	26539	+	81
NW_015809922.1	252688	252778	-	90
NW_015810161.1	17900	17948	-	48
NW_015810161.1	17890	17956	-	66
NW_015810909.1	3044	3189	+	145
NW_015810909.1	3035	3202	+	167
NW_015811030.1	14521	14560	+	39
NW_015811093.1	30678	30774	-	96
NW_015811093.1	30674	30810	-	136
NW_015811492.1	26720	26764	+	44
NW_015811548.1	78425	78452	-	27
NW_015812697.1	18399	18507	-	108
NW_015812697.1	18375	18505	-	130
NW_015813552.1	26881	26911	-	30
NW_015813573.1	19503	19533	+	30
NW_015813708.1	107379	107501	-	122
NW_015814212.1	30973	31107	-	134
NW_015814616.1	7659	7748	+	89
NW_015816087.1	26546	26652	+	106
NW_015816087.1	26891	27100	+	209

NW_015816487.1	17722	17740	-	18
NW_015816700.1	58291	58354	-	63
NW_015816896.1	23630	23760	-	130
NW_015817312.1	71932	71987	+	55
NW_015817414.1	10503	10622	+	72
NW_015819081.1	26887	26984	+	97
NW_015819155.1	6014	6078	+	64
NW_015819901.1	35916	35962	-	46
NW_015819901.1	35934	35970	-	36
NW_015824421.1	71730	71807	-	77
NW_015824421.1	71735	71824	-	89
NW_015824765.1	4982	5140	-	158
NW_015825133.1	93547	93604	-	57
NW_015825212.1	32488	32618	-	130
NW_015825212.1	32481	32615	-	134
NW_015825712.1	31733	31838	-	105
NW_015826128.1	9337	9447	+	79
NW_015827070.1	124734	124793	+	59
NW_015827184.1	102378	102486	+	108
NW_015827951.1	93309	93362	+	53
NW_015827951.1	93288	93360	+	72
NW_015828298.1	19045	19144	+	99
NW_015828889.1	2724	2777	-	53
NW_015828889.1	2743	2774	-	31
NW_015828889.1	2544	2788	-	244

NW_015829845.1	54256	54323	+	67
NW_015830779.1	6297	6428	+	131
NW_015832748.1	27591	27760	-	169
NW_015832833.1	16594	16804	+	210
NW_015832833.1	16597	16816	+	219
NW_015833229.1	7849	7915	+	66
NW_015833252.1	11284	11432	-	148
NW_015838383.1	4585	4691	-	106
NW_015838383.1	4590	4702	-	112
NW_015840179.1	13832	13957	+	125
NW_015840179.1	13837	13953	+	116
NW_015840790.1	25021	25184	+	163
NW_015840790.1	26251	26299	+	48
NW_015840790.1	26454	26478	+	24
NW_015840790.1	25038	25187	+	149
NW_015840790.1	26262	26321	+	59
NW_015840790.1	25015	25194	+	179
NW_015840790.1	26225	26336	+	111
NW_015841211.1	174854	175033	+	179
NW_015841211.1	174840	175038	+	198
NW_015841918.1	75704	75820	+	116
NW_015841918.1	75571	75837	+	266
NW_015843003.1	18644	18807	+	163
NW_015843003.1	18643	18793	+	150
NW_015843003.1	18649	18803	+	154

NW_015845141.1	51747	51819	+	72
NW_015847361.1	64348	64595	+	247
NW_015847361.1	64400	64593	+	193
NW_015847361.1	64375	64613	+	238
NW_015848008.1	4652	4691	-	39
NW_015849664.1	59725	59803	+	41
NW_015850369.1	3710	3776	-	66
NW_015850369.1	3660	3770	-	110
NW_015850369.1	3647	3766	-	119
NW_015851354.1	64752	64858	-	106
NW_015851354.1	64706	64850	-	144
NW_015851838.1	2183	2208	-	25
NW_015853692.1	28585	28641	-	56
NW_015854679.1	1876	2008	-	132
NW_015854679.1	1711	1991	-	280
NW_015854679.1	1615	2016	-	401
NW_015854775.1	10888	10937	-	49
NW_015854997.1	78100	78123	+	23
NW_015855009.1	200665	200745	-	80
NW_015855009.1	200655	200751	-	96
NW_015855210.1	44112	44134	+	22
NW_015855617.1	23946	24003	-	57
NW_015856685.1	64210	64235	-	25
NW_015856793.1	18992	19062	+	70
NW_015857501.1	62435	62471	+	36

NW_015857996.1	20904	21042	+	138
NW_015857996.1	20914	21046	+	132
NW_015858103.1	26066	26144	-	78
NW_015858696.1	77168	77252	-	84
NW_015858696.1	77135	77249	-	114
NW_015858936.1	2194	2267	+	73
NW_015859358.1	35861	35960	+	99
NW_015859577.1	15550	15608	-	58
NW_015859720.1	25738	25908	+	170
NW_015860585.1	90054	90140	-	86
NW_015860585.1	90053	90105	-	52
NW_015860585.1	90045	90143	-	98
NW_015860976.1	2130	2202	+	72
NW_015862129.1	11692	11731	+	39
NW_015862129.1	11686	11734	+	48
NW_015864800.1	59128	59453	+	325
NW_015866444.1	3619	3673	+	54
NW_015866746.1	13197	13303	+	106
NW_015867459.1	61869	61908	+	39
NW_015867465.1	26107	26192	-	85
NW_015867465.1	26125	26193	-	68
NW_015868264.1	6323	6416	+	93
NW_015868471.1	33344	33394	-	50
NW_015868471.1	33347	33412	-	65
NW_015869079.1	9237	9341	-	104

NW_015869079.1	9192	9348	-	156
NW_015870571.1	180766	180836	-	70
NW_015873620.1	64931	64983	+	52
NW_015877345.1	66030	66087	-	57
NW_015877516.1	24245	24295	+	50
NW_015877516.1	24234	24339	+	105
NW_015880571.1	64417	64580	-	163
NW_015880571.1	64411	64605	-	194
NW_015881616.1	30253	30325	+	72
NW_015883696.1	19333	19668	+	335
NW_015885042.1	7831	7900	-	69
NW_015885121.1	33892	33958	+	66
NW_015885121.1	33874	33955	+	81
NW_015885498.1	26086	26179	-	93
NW_015885617.1	9772	9874	-	102
NW_015885617.1	9774	9837	-	63
NW_015885851.1	42360	42429	+	69
NW_015886012.1	55256	55324	-	68
NW_015886012.1	55263	55319	-	56
NW_015886012.1	55266	55306	-	40
NW_015886127.1	29852	30059	-	207
NW_015886127.1	29854	29983	-	129
NW_015886127.1	29850	30069	-	219
NW_015886146.1	30401	30464	+	63
NW_015886670.1	70822	70867	-	45

NW_015886670.1	70809	70883	-	74
NW_015886799.1	109057	109147	+	90
NW_015886799.1	109050	109158	+	108
NW_015886839.1	21641	21699	+	58
NW_015886839.1	21655	21705	+	50
NW_015886840.1	21882	22097	-	215
NW_015886840.1	21885	22026	-	141
NW_015887883.1	35987	36076	-	89
NW_015888274.1	106815	106900	-	85
NW_015888421.1	11513	11613	-	90
NW_015888612.1	215072	215133	+	61
NW_015888685.1	9466	9482	+	16
NW_015889447.1	86388	86540	+	152
NW_015890066.1	8552	8589	+	37
NW_015890123.1	78683	78751	+	68
NW_015890168.1	14850	15003	-	153
NW_015890168.1	14842	15011	-	169
NW_015890230.1	34013	34061	+	48
NW_015890619.1	47845	47949	+	104
NW_015890831.1	24058	24161	-	103
NW_015890831.1	24041	24156	-	115
NW_015890876.1	47302	47414	+	112
NW_015890876.1	47288	47445	+	157
NW_015890876.1	47286	47451	+	165
NW_015891490.1	34948	35033	-	85

NW_015891636.1	60171	60255	+	84
NW_015892309.1	95903	96215	+	312
NW_015892309.1	95884	96220	+	336
NW_015892309.1	95889	96219	+	330
NW_015892882.1	17408	17483	-	75
NW_015894176.1	6579	6945	-	366
NW_015894176.1	6555	6818	-	263
NW_015894176.1	6546	6806	-	260
NW_015894860.1	41609	41711	+	102
NW_015894860.1	41602	41755	+	153
NW_015894954.1	33	147	-	114
NW_015897470.1	2238	2312	-	74
NW_015897470.1	99	348	-	249
NW_015897470.1	2182	2319	-	137
NW_015898069.1	456	615	-	159
NW_015898292.1	1369	1511	-	142
NW_015898292.1	1371	1516	-	145
NW_015898292.1	1363	1520	-	157
NW_015898669.1	4829	4889	+	60
NW_015898700.1	1731	1837	-	106
NW_015898709.1	2308	2454	+	146
NW_015898709.1	2310	2451	+	141
NW_015898709.1	2833	3012	+	179
NW_015898709.1	2306	2471	+	165
NW_015898709.1	2803	3051	+	248

NW_015898861.1	5335	5563	-	228
NW_015898864.1	5135	5319	+	184
NW_015898864.1	5136	5283	+	147
NW_015898985.1	10868	10912	-	44
NW_015899502.1	40640	40658	+	18
NW_015899502.1	40642	40663	+	21
NW_015899767.1	24453	24589	+	136
NW_015900112.1	27606	27667	+	61
NW_015900112.1	27587	27676	+	89
NW_015900220.1	114092	114228	-	136
NW_015900255.1	30794	30875	+	81
NW_015900466.1	4603	4655	-	52
NW_015900939.1	24224	24485	+	261
NW_015900939.1	24202	24510	+	308
NW_015900939.1	24199	24552	+	353
NW_015901537.1	22831	22940	+	109
NW_015901537.1	23832	23940	+	108
NW_015901537.1	22809	22915	+	106
NW_015901544.1	16510	16553	-	43
NW_015901544.1	15884	15959	-	75
NW_015901544.1	16495	16580	-	85
NW_015902891.1	26086	26134	+	48
NW_015902993.1	21641	21653	+	12
NW_015902993.1	21553	21639	+	86
NW_015903273.1	39120	39207	-	87

NW_015903755.1	93532	93625	+	93
NW_015903797.1	126747	126907	+	160
NW_015903846.1	51357	51823	-	466
NW_015903932.1	10767	10825	-	58
NW_015904444.1	31944	32211	+	267
NW_015904444.1	32071	32205	+	134
NW_015904622.1	4419	4455	+	36
NW_015904981.1	64935	64968	+	33
NW_015904981.1	64931	65021	+	90
NW_015905140.1	28283	28342	+	59
NW_015905901.1	30356	30391	-	35
NW_015906117.1	5744	5762	-	18
NW_015906190.1	107599	107776	-	177
NW_015906190.1	107560	107775	-	215
NW_015906375.1	95121	95386	+	265
NW_015906375.1	95090	95387	+	297
NW_015906621.1	197120	197162	-	42
NW_015906788.1	39679	39883	+	204
NW_015906788.1	39653	40075	+	422
NW_015906892.1	134680	134775	-	95
NW_015907203.1	44246	44351	+	105
NW_015907203.1	44224	44363	+	139
NW_015907225.1	21938	22013	+	75
NW_015907265.1	20547	20631	+	84
NW_015907265.1	20554	20729	+	175

NW_015907489.1	26868	26920	+	52
NW_015907631.1	8199	8214	+	15
NW_015908325.1	25779	25832	-	53
NW_015908325.1	25788	25886	-	98
NW_015908633.1	37121	37141	-	20
NW_015908995.1	240968	241172	+	204
NW_015908995.1	240971	241166	+	195
NW_015909244.1	8272	8465	+	107
NW_015909256.1	25601	25685	-	84
NW_015909256.1	25535	25686	-	151
NW_015909256.1	25522	25697	-	175
NW_015910200.1	26233	26321	+	88
NW_015910200.1	26238	26332	+	94
NW_015910523.1	44068	44196	-	128
NW_015910523.1	44070	44188	-	118
NW_015910523.1	44072	44203	-	131
NW_015911274.1	11065	11177	-	112
NW_015911627.1	53476	53565	+	89
NW_015912020.1	35276	35356	-	80
NW_015912288.1	55944	56095	-	151
NW_015912853.1	8787	8932	+	145
NW_015913029.1	32849	32951	-	102
NW_015913029.1	32840	32921	-	81
NW_015913686.1	35738	35840	-	102
NW_015913720.1	47068	47363	+	163

NW_015914227.1	7858	7946	+	88
NW_015914389.1	18466	18559	-	93
NW_015914389.1	18474	18590	-	116
NW_015914675.1	45490	45539	+	49
NW_015914817.1	68029	68150	+	121
NW_015915485.1	15042	15318	-	276
NW_015916664.1	61844	61939	+	95
NW_015916737.1	14286	14394	-	101
NW_015916741.1	1042	1197	+	155
NW_015917211.1	89768	89860	+	92
NW_015917774.1	1527	1677	-	150
NW_015918282.1	38894	38973	+	79
NW_015918415.1	21353	21436	-	83
NW_015919061.1	78176	78328	+	152
NW_015919160.1	20829	20991	-	162
NW_015919160.1	20841	20988	-	147
NW_015919160.1	20805	21015	-	210
NW_015919290.1	4493	4585	+	92
NW_015919290.1	4488	4582	+	94
NW_015919747.1	51180	51309	-	129
NW_015919747.1	51183	51316	-	133
NW_015919812.1	40875	40917	-	42
NW_015920546.1	62836	62889	-	53
NW_015920676.1	70316	70475	+	159
NW_015920676.1	70304	70496	+	192

NW_015920910.1	76985	77063	+	78
NW_015921235.1	34965	35131	-	166
NW_015921235.1	34958	35133	-	175
NW_015921424.1	43423	43486	-	63
NW_015921439.1	41802	41855	+	53
NW_015921466.1	79892	79997	-	105
NW_015921466.1	79884	79959	-	75
NW_015921800.1	47856	47904	-	48
NW_015922161.1	13362	13374	+	12
NW_015923102.1	86155	86183	-	28
NW_015923440.1	26030	26077	+	47
NW_015923478.1	10656	10797	+	105
NW_015923998.1	5573	5642	+	69
NW_015923998.1	5548	5691	+	143
NW_015924512.1	5526	5601	-	75
NW_015924771.1	20615	20728	+	113
NW_015924987.1	29622	29815	+	193
NW_015924987.1	29624	29814	+	190
NW_015924987.1	29630	29824	+	194
NW_015925245.1	63729	63777	-	48
NW_015925505.1	3321	3498	+	177
NW_015925505.1	3318	3437	+	119
NW_015925709.1	55140	55184	+	44
NW_015926076.1	65116	65217	+	101
NW_015926256.1	37349	37377	+	28

NW_015926811.1	73373	73509	+	136
NW_015926811.1	73350	73519	+	169
NW_015927007.1	41388	41570	-	182
NW_015927007.1	41387	41581	-	194
NW_015927007.1	41396	41580	-	184
NW_015928664.1	2154	2215	+	61
NW_015928664.1	2167	2224	+	57
NW_015928664.1	2157	2221	+	64
NW_015929020.1	5102	5127	+	25
NW_015929455.1	27178	27311	+	131
NW_015929623.1	52090	52134	-	44
NW_015929893.1	29970	30076	-	106
NW_015929893.1	29965	30107	-	142
NW_015929893.1	29988	30156	-	168
NW_015929962.1	26560	26617	-	57
NW_015930022.1	108338	108615	+	277
NW_015930022.1	108325	108720	+	395
NW_015930022.1	108329	108734	+	405
NW_015930988.1	8788	8912	-	124
NW_015932419.1	27674	27760	-	86
NW_015933677.1	22423	22545	-	122
NW_015934159.1	83747	83803	-	56
NW_015934187.1	152898	153000	+	102
NW_015934187.1	152906	153010	+	104
NW_015934627.1	3878	3933	+	55

NW_015934705.1	97092	97159	+	67
NW_015934762.1	153538	153602	-	64
NW_015934893.1	39611	39747	-	136
NW_015935386.1	123423	123525	+	102
NW_015935397.1	14362	14487	+	125
NW_015935397.1	14285	14488	+	203
NW_015936986.1	29436	29528	-	92
NW_015937021.1	16347	16412	-	65
NW_015937021.1	16344	16418	-	74
NW_015937119.1	25360	25400	-	40
NW_015937468.1	48178	48217	-	39
NW_015938613.1	11042	11184	-	142
NW_015938920.1	36542	36565	-	23
NW_015939165.1	7128	7170	-	42
NW_015939283.1	3613	3704	-	91
NW_015939283.1	3652	3700	-	48
NW_015939450.1	11774	11892	+	118
NW_015939455.1	21566	21661	+	95
NW_015939606.1	115325	115495	-	170
NW_015940037.1	26720	26777	-	57
NW_015940632.1	157202	157393	+	191
NW_015940632.1	157268	157419	+	151
NW_015941143.1	23029	23130	-	101
NW_015941741.1	18730	18870	+	140
NW_015942779.1	51828	51975	-	147

NW_015942779.1	51833	51968	-	135
NW_015942779.1	51837	51973	-	136
NW_015942802.1	19804	19938	+	85
NW_015943297.1	14516	14540	-	24
NW_015943434.1	11478	11540	+	62
NW_015943817.1	28099	28257	+	80
NW_015944541.1	1144	1288	-	17
NW_015944541.1	1202	1282	-	11
NW_015944541.1	1155	1286	-	15
NW_015945478.1	6206	6493	-	287
NW_015945478.1	6238	6348	-	110
NW_015945478.1	6328	6495	-	167
NW_015945687.1	4682	4723	+	41
NW_015945791.1	36090	36195	-	105
NW_015945791.1	36097	36216	-	119
NW_015945791.1	36327	36500	-	173
NW_015945791.1	36079	36526	-	447
NW_015946081.1	21339	21410	-	71
NW_015946508.1	71161	71266	-	105
NW_015946545.1	19464	19590	+	126
NW_015946760.1	77007	77055	+	48
NW_015946913.1	24164	24233	-	69
NW_015946913.1	24170	24236	-	66
NW_015946913.1	24380	24444	-	64
NW_015946913.1	24551	24651	-	100

NW_015947193.1	78236	78248	+	12
NW_015947295.1	5456	5681	-	225
NW_015947669.1	41527	41563	-	36
NW_015948225.1	47255	47306	+	51
NW_015948286.1	18059	18130	-	71
NW_015948616.1	45057	45096	+	39
NW_015948616.1	45064	45117	+	53
NW_015948616.1	45061	45094	+	33
NW_015948766.1	55874	56010	+	136
NW_015948766.1	55866	56024	+	158
NW_015949806.1	31799	32013	-	214
NW_015949806.1	31817	31876	-	59
NW_015949806.1	31806	31997	-	191
NW_015950258.1	81286	81328	+	42
NW_015950258.1	81262	81347	+	85
NW_015950258.1	99495	99574	+	79
NW_015950514.1	27294	27401	-	107
NW_015952120.1	32556	32742	+	186
NW_015952120.1	32555	32729	+	174
NW_015952120.1	32551	32728	+	177
NW_015952592.1	4390	4466	-	76
NW_015953580.1	38630	38810	-	180
NW_015953580.1	38634	38757	-	123
NW_015953580.1	38483	38986	-	503
NW_015953774.1	68318	68389	+	71

NW_015953774.1	68320	68409	+	89
NW_015954636.1	17276	17425	-	149
NW_015954636.1	17321	17417	-	96
NW_015954636.1	17264	17423	-	159

13days post inoculation: recovery site (Hypo-DMRs)

Scaffold	Start	End	Region stain	Overlapped site
NW_015787245.1	16646	16702	+	56
NW_015787340.1	4235	4291	+	56
NW_015787340.1	4198	4299	+	101
NW_015787340.1	4177	4284	+	107
NW_015787408.1	33338	33352	+	14
NW_015787444.1	10343	10384	+	41
NW_015787466.1	19628	19765	-	137
NW_015787581.1	16527	16607	+	80
NW_015787655.1	25044	25132	+	88
NW_015787679.1	23068	23131	-	63
NW_015787703.1	14858	14924	+	66
NW_015787775.1	33801	33918	+	117
NW_015787854.1	27743	27815	-	72
NW_015788138.1	67168	67212	+	44
NW_015788281.1	48593	48693	-	100
NW_015788317.1	27663	27697	-	34
NW_015788476.1	22944	22962	-	18

NW_015788608.1	29248	29378	-	130
NW_015788608.1	29233	29373	-	140
NW_015788719.1	36282	36340	-	58
NW_015788759.1	24700	24779	+	79
NW_015788794.1	29612	29694	-	82
NW_015788908.1	33022	33135	+	113
NW_015789061.1	328769	328865	-	65
NW_015789251.1	12151	12230	-	79
NW_015789251.1	12147	12290	-	143
NW_015789259.1	20462	20552	+	90
NW_015789435.1	60999	61125	+	71
NW_015789501.1	50521	50577	+	56
NW_015789559.1	85955	86037	-	82
NW_015789620.1	81358	81447	-	89
NW_015789785.1	18510	18664	-	154
NW_015789834.1	2212	2468	-	256
NW_015789853.1	1789	1833	-	44
NW_015789944.1	6670	6888	+	95
NW_015789982.1	35402	35633	-	231
NW_015789982.1	35384	35981	-	597
NW_015790061.1	37237	37402	-	165
NW_015790061.1	37219	37393	-	174
NW_015790311.1	40604	40744	+	140
NW_015790359.1	51038	51121	+	83
NW_015790638.1	12900	12930	-	30

NW_015790646.1	48881	49008	+	127
NW_015790646.1	22571	22653	+	82
NW_015790989.1	84071	84199	-	128
NW_015790989.1	84096	84207	-	111
NW_015791039.1	91890	92013	+	123
NW_015791090.1	2344	2438	-	94
NW_015791242.1	72536	72652	+	116
NW_015791267.1	88666	88736	+	70
NW_015791267.1	88662	88739	+	77
NW_015791267.1	5009	5175	-	166
NW_015791370.1	36259	36331	+	72
NW_015791433.1	29394	29491	-	97
NW_015791444.1	14986	15019	+	33
NW_015791444.1	14991	15038	+	47
NW_015791453.1	128911	129017	-	90
NW_015791453.1	128908	129034	-	93
NW_015791472.1	17142	17367	+	225
NW_015791475.1	11302	11346	-	44
NW_015791789.1	8994	9062	-	68
NW_015791861.1	30426	30514	+	88
NW_015791955.1	18319	18403	+	84
NW_015791978.1	10615	10677	-	62
NW_015792055.1	122843	122956	-	113
NW_015792085.1	53743	53780	-	24
NW_015792092.1	2591	2721	-	118

NW_015792093.1	38359	38469	+	110
NW_015792093.1	38352	38460	+	108
NW_015792093.1	22133	22228	+	95
NW_015792254.1	17861	17952	-	91
NW_015792437.1	21256	21394	-	114
NW_015792642.1	36252	36386	+	134
NW_015792642.1	36224	36402	+	178
NW_015792740.1	31910	31951	-	41
NW_015792849.1	68480	68579	+	99
NW_015792849.1	68447	68573	+	126
NW_015792876.1	60246	60414	-	168
NW_015793024.1	9280	9393	+	113
NW_015793027.1	26603	26720	-	117
NW_015793027.1	26590	26701	-	111
NW_015793035.1	101355	101383	-	28
NW_015793173.1	16365	16460	+	95
NW_015793231.1	66055	66093	-	38
NW_015793234.1	7948	8048	-	100
NW_015793281.1	87501	87595	-	94
NW_015793413.1	13600	13710	+	110
NW_015793434.1	11970	12023	+	53
NW_015793434.1	11974	12014	+	40
NW_015793434.1	11966	12009	+	43
NW_015793546.1	24720	24979	-	259
NW_015793546.1	25526	25641	-	115

NW_015793546.1	25549	25707	-	158
NW_015793609.1	41023	41059	-	36
NW_015793638.1	7538	7579	-	41
NW_015793693.1	7474	7543	+	69
NW_015793693.1	7466	7574	+	108
NW_015793793.1	36222	36339	+	117
NW_015793793.1	36248	36341	+	93
NW_015793807.1	62853	62916	+	63
NW_015793957.1	6495	6598	-	103
NW_015793957.1	6497	6583	-	86
NW_015794029.1	232654	232848	+	194
NW_015794132.1	199383	199451	-	68
NW_015794232.1	24845	24925	-	80
NW_015794475.1	148446	148532	-	86
NW_015794475.1	149402	149474	-	47
NW_015794503.1	61912	61962	+	50
NW_015794511.1	38160	38247	+	87
NW_015794640.1	35733	35905	+	172
NW_015794667.1	1387	1417	+	30
NW_015794667.1	1384	1452	+	68
NW_015794716.1	4640	4864	+	224
NW_015794811.1	30846	30882	+	36
NW_015794904.1	10967	11113	+	113
NW_015794943.1	1700	1824	+	124
NW_015795024.1	19936	19998	+	62

NW_015795027.1	205	231	+	26
NW_015795187.1	22752	22786	+	34
NW_015795187.1	22678	22774	+	96
NW_015795193.1	3213	3328	+	115
NW_015795233.1	37982	38108	-	126
NW_015795243.1	48774	48833	-	59
NW_015795290.1	50549	50733	+	184
NW_015795412.1	19528	19665	-	137
NW_015795426.1	27376	27448	-	72
NW_015795512.1	97351	97452	+	101
NW_015795620.1	18963	18987	-	24
NW_015795660.1	36239	36303	+	64
NW_015795724.1	17595	17723	-	128
NW_015795724.1	18896	19083	-	187
NW_015795761.1	135477	135567	+	90
NW_015795769.1	43937	43997	+	60
NW_015795779.1	1184	1249	-	65
NW_015795818.1	159555	159673	+	67
NW_015795820.1	2155	2302	+	147
NW_015796081.1	7357	7453	-	96
NW_015796100.1	7542	7635	-	93
NW_015796173.1	1876	1993	-	117
NW_015796367.1	73800	73879	-	79
NW_015796367.1	14637	14824	+	187
NW_015796367.1	73811	73872	-	61

NW_015796504.1	33067	33205	+	84
NW_015796526.1	59054	59206	+	152
NW_015796699.1	7719	7849	-	130
NW_015796835.1	2827	2953	+	126
NW_015796881.1	2336	2388	+	52
NW_015797238.1	17098	17159	+	61
NW_015797342.1	138996	139072	-	76
NW_015797903.1	7918	8061	+	143
NW_015797936.1	16742	16894	+	152
NW_015797950.1	21559	21588	+	29
NW_015797979.1	89946	89970	+	24
NW_015798079.1	22005	22092	-	87
NW_015798079.1	21991	22091	-	100
NW_015798375.1	28690	28745	+	55
NW_015798390.1	77097	77190	-	93
NW_015798507.1	143625	143762	+	137
NW_015798807.1	13443	13527	-	84
NW_015798807.1	13434	13535	-	101
NW_015798921.1	15456	15516	+	60
NW_015798928.1	26363	26403	+	25
NW_015799019.1	5512	5750	+	238
NW_015799022.1	36806	36946	+	140
NW_015799308.1	8578	8719	-	141
NW_015799321.1	12430	12495	-	65
NW_015799321.1	12427	12727	-	300

NW_015799362.1	84891	84995	+	104
NW_015799535.1	11378	11431	-	53
NW_015799598.1	74550	74703	+	153
NW_015799621.1	6894	6985	-	91
NW_015799622.1	11625	11728	+	103
NW_015799653.1	74598	74636	-	38
NW_015799669.1	22014	22169	-	155
NW_015799705.1	10676	10756	+	80
NW_015799748.1	21656	21824	-	168
NW_015799963.1	1678	1766	-	88
NW_015800029.1	24523	24644	+	121
NW_015800091.1	33692	33870	+	178
NW_015800126.1	6009	6194	+	94
NW_015800145.1	110031	110224	+	193
NW_015800329.1	18041	18171	+	130
NW_015800329.1	18048	18151	+	103
NW_015800675.1	52290	52316	+	26
NW_015800693.1	7810	7867	+	57
NW_015800721.1	6643	6690	+	47
NW_015800760.1	19721	19777	+	56
NW_015800821.1	2608	2685	-	77
NW_015800821.1	2795	2849	-	54
NW_015801051.1	6607	6726	-	119
NW_015801051.1	6604	6792	-	188
NW_015801269.1	5018	5125	+	107

NW_015801275.1	37718	37834	+	116
NW_015801281.1	80858	80968	-	110
NW_015801296.1	24532	24688	+	156
NW_015801414.1	16247	16378	-	131
NW_015801522.1	7050	7245	-	138
NW_015801552.1	62319	62557	+	238
NW_015801641.1	8664	8737	+	73
NW_015801684.1	49462	49568	-	106
NW_015801843.1	10817	11223	-	406
NW_015801843.1	10912	11222	-	310
NW_015801843.1	10740	10893	-	153
NW_015801924.1	58549	58665	-	116
NW_015801952.1	38315	38520	-	205
NW_015801952.1	38303	38600	-	297
NW_015801978.1	51439	51502	-	63
NW_015802430.1	6794	6900	+	106
NW_015802451.1	28676	28815	+	139
NW_015802603.1	11901	12021	-	120
NW_015802882.1	19046	19155	+	109
NW_015802919.1	3053	3254	+	201
NW_015802919.1	3077	3265	+	188
NW_015802928.1	32859	32942	+	83
NW_015802931.1	56698	56771	-	73
NW_015802947.1	25671	25746	+	75
NW_015802956.1	51136	51235	+	99

NW_015803142.1	17625	17655	+	30
NW_015803187.1	20213	20373	+	160
NW_015803306.1	17294	17550	-	256
NW_015803360.1	6102	6214	-	112
NW_015803472.1	28674	28735	+	61
NW_015803517.1	66240	66344	-	104
NW_015803680.1	56914	57196	-	282
NW_015803686.1	14647	14757	-	110
NW_015803742.1	16984	17080	-	96
NW_015803759.1	1754	1831	+	77
NW_015803759.1	1740	1846	+	106
NW_015803772.1	2426	2538	-	112
NW_015803796.1	18233	18299	-	66
NW_015804073.1	57144	57290	+	146
NW_015804073.1	63010	63110	+	79
NW_015804073.1	62213	62278	+	65
NW_015804242.1	4963	5169	-	206
NW_015804352.1	1298	1353	-	55
NW_015804499.1	55633	55674	+	41
NW_015804791.1	14517	14581	-	64
NW_015804894.1	123329	123404	+	75
NW_015804948.1	5931	6049	-	118
NW_015805054.1	27261	27330	-	69
NW_015805116.1	24599	24661	+	62
NW_015805172.1	96579	96631	+	52

NW_015805288.1	16605	16734	+	129
NW_015805407.1	7771	7918	-	147
NW_015805720.1	11273	11342	-	69
NW_015805896.1	6023	6162	+	139
NW_015806020.1	5541	5572	+	31
NW_015806335.1	42518	42596	+	78
NW_015806335.1	42515	42659	+	144
NW_015806352.1	101	203	-	102
NW_015806442.1	8201	8323	+	122
NW_015806442.1	8216	8311	+	95
NW_015806485.1	21524	21579	-	55
NW_015806485.1	21527	21595	-	68
NW_015806525.1	30319	30416	-	60
NW_015806621.1	186	329	-	143
NW_015806672.1	132482	132538	-	56
NW_015806686.1	7630	7721	-	64
NW_015806849.1	14215	14325	-	110
NW_015806933.1	58493	58531	-	38
NW_015806933.1	58498	58521	-	23
NW_015807019.1	23798	23895	+	97
NW_015807042.1	59855	59914	+	59
NW_015807273.1	12403	12499	-	96
NW_015807368.1	15014	15162	-	109
NW_015807383.1	17850	18014	+	164
NW_015807433.1	2684	2748	-	64

NW_015807521.1	34363	34522	-	159
NW_015807570.1	2297	2364	+	67
NW_015807629.1	164399	164437	+	38
NW_015807629.1	164402	164453	+	51
NW_015807646.1	40878	41024	-	146
NW_015807950.1	125335	125392	+	57
NW_015808065.1	8882	9041	-	159
NW_015808065.1	8698	9052	-	354
NW_015808069.1	86680	86876	-	196
NW_015808282.1	24839	24913	-	54
NW_015808424.1	30242	30318	+	76
NW_015808465.1	13113	13293	-	124
NW_015808465.1	13047	13323	-	190
NW_015808796.1	17838	17923	-	85
NW_015808796.1	17825	17920	-	95
NW_015809059.1	23145	23301	-	156
NW_015809059.1	23128	23311	-	183
NW_015809359.1	27774	27825	+	51
NW_015809440.1	6470	6528	-	58
NW_015809447.1	2254	2282	-	28
NW_015809447.1	2269	2304	-	35
NW_015809596.1	2889	3084	-	195
NW_015809596.1	2847	3141	-	294
NW_015809613.1	11692	11784	-	92
NW_015809613.1	11685	11785	-	100

NW_015809667.1	42707	42777	-	70
NW_015809667.1	50808	50912	+	104
NW_015809729.1	7614	7705	-	91
NW_015809729.1	7608	7695	-	87
NW_015809825.1	60628	60697	+	69
NW_015809867.1	16498	16578	+	80
NW_015809920.1	16831	16926	+	95
NW_015809920.1	17075	17240	+	165
NW_015809922.1	258977	259134	-	157
NW_015810004.1	15765	15846	-	81
NW_015810058.1	27371	27507	-	136
NW_015810194.1	43106	43214	+	104
NW_015810199.1	25976	26044	-	68
NW_015810426.1	51286	51402	+	116
NW_015810449.1	2293	2394	-	101
NW_015810452.1	2773	2873	+	100
NW_015810795.1	1974	2085	+	111
NW_015810839.1	52008	52035	+	27
NW_015810861.1	99394	99522	+	128
NW_015810923.1	19496	19537	-	41
NW_015811030.1	28918	28967	-	49
NW_015811033.1	251913	251949	+	36
NW_015811033.1	251920	251957	+	37
NW_015811033.1	275748	275801	+	53
NW_015811167.1	153228	153327	+	99

NW_015811571.1	41227	41316	-	89
NW_015811717.1	43125	43143	+	18
NW_015811787.1	21055	21196	+	141
NW_015811876.1	52045	52146	-	101
NW_015811883.1	109744	109885	+	141
NW_015811973.1	12955	13048	-	93
NW_015812007.1	8154	8243	+	89
NW_015812026.1	13953	14051	-	98
NW_015812155.1	18473	18611	+	138
NW_015812182.1	32988	33118	+	130
NW_015812182.1	32957	33110	+	153
NW_015812256.1	18834	18947	+	113
NW_015812279.1	7641	7675	+	34
NW_015812421.1	31305	31407	-	102
NW_015812517.1	35229	35336	-	107
NW_015812517.1	35234	35345	-	111
NW_015812596.1	213089	213209	+	120
NW_015812697.1	3821	3895	+	74
NW_015812873.1	5298	5358	+	60
NW_015813218.1	14439	14489	-	50
NW_015813270.1	11221	11341	-	120
NW_015813270.1	59034	59131	-	97
NW_015813270.1	11024	11087	-	63
NW_015813270.1	10650	10773	-	123
NW_015813270.1	11012	11099	-	87

NW_015813270.1	11203	11344	-	141
NW_015813270.1	59041	59125	-	84
NW_015813472.1	18623	18666	+	43
NW_015813499.1	85602	85632	+	30
NW_015813573.1	19127	19228	+	101
NW_015813708.1	107401	107506	-	105
NW_015813738.1	34159	34258	+	99
NW_015813738.1	48425	48522	-	97
NW_015813771.1	20066	20190	+	124
NW_015813936.1	25639	25699	-	60
NW_015814130.1	53695	53759	-	64
NW_015814225.1	5812	5969	-	102
NW_015814451.1	31145	31274	-	129
NW_015814715.1	1788	1899	+	111
NW_015814964.1	207450	207502	-	52
NW_015815014.1	3149	3290	+	141
NW_015815014.1	3131	3383	+	252
NW_015815030.1	72530	72723	-	193
NW_015815149.1	6889	6995	-	106
NW_015815430.1	123680	123727	-	47
NW_015815430.1	122823	123335	-	512
NW_015815745.1	7567	7701	-	134
NW_015815911.1	18923	19012	+	89
NW_015815911.1	18840	19018	+	178
NW_015816359.1	35470	35541	-	71

NW_015816773.1	19117	19265	+	148
NW_015816773.1	19190	19263	+	73
NW_015816928.1	16818	16944	+	126
NW_015816976.1	11892	12054	+	162
NW_015817148.1	17160	17256	+	96
NW_015817164.1	26635	26675	-	40
NW_015817186.1	36108	36255	-	147
NW_015817312.1	71906	71982	+	76
NW_015817657.1	7893	8088	-	195
NW_015817657.1	8454	8590	-	136
NW_015817695.1	25745	25854	+	109
NW_015817715.1	18469	18499	-	30
NW_015817897.1	14525	14611	-	86
NW_015818032.1	38653	38887	-	234
NW_015818074.1	22326	22371	-	32
NW_015818104.1	7336	7379	+	43
NW_015818139.1	19048	19334	+	286
NW_015818197.1	18781	18882	+	101
NW_015818207.1	17913	17970	-	57
NW_015818214.1	44014	44115	-	101
NW_015818238.1	52705	52852	+	147
NW_015818305.1	38260	38349	+	60
NW_015818566.1	15861	15990	+	129
NW_015818713.1	10504	10528	+	24
NW_015818917.1	47642	47801	-	159

NW_015819123.1	6937	7016	+	79
NW_015819357.1	2095	2267	+	172
NW_015819412.1	32649	32757	-	108
NW_015819412.1	32657	32747	-	90
NW_015819412.1	32659	32764	-	105
NW_015819541.1	104661	104727	-	66
NW_015819643.1	25090	25137	-	47
NW_015819643.1	25096	25135	-	39
NW_015819643.1	24954	25145	-	191
NW_015819923.1	40385	40504	-	119
NW_015820176.1	27904	27997	-	93
NW_015820325.1	23331	23445	-	114
NW_015820325.1	22891	22928	-	37
NW_015820359.1	32920	32956	+	36
NW_015820359.1	32949	33012	+	63
NW_015820416.1	10129	10196	-	67
NW_015820453.1	36769	36965	+	187
NW_015820454.1	21308	21463	-	155
NW_015820494.1	4821	4956	+	135
NW_015820820.1	44223	44359	+	136
NW_015820852.1	227003	227067	+	64
NW_015820923.1	18048	18172	-	124
NW_015820923.1	18056	18173	-	117
NW_015821064.1	23366	23392	-	26
NW_015821066.1	59264	59441	+	177

NW_015821066.1	60254	60330	+	76
NW_015821092.1	142	299	+	79
NW_015821092.1	18	326	+	203
NW_015821193.1	2217	2276	-	59
NW_015821240.1	17257	17338	-	81
NW_015821299.1	54142	54234	+	92
NW_015821305.1	24923	25061	+	138
NW_015821370.1	38709	38821	-	112
NW_015821533.1	42536	42578	+	42
NW_015821688.1	64416	64502	-	86
NW_015821718.1	16385	16478	+	93
NW_015821763.1	4810	4929	-	119
NW_015821919.1	3820	3942	+	122
NW_015821919.1	3797	3951	+	154
NW_015821994.1	80395	80445	-	50
NW_015822001.1	13952	14079	-	88
NW_015822028.1	23161	23220	-	59
NW_015822028.1	23148	23337	-	189
NW_015822203.1	38579	38646	+	67
NW_015822450.1	36245	36408	+	163
NW_015822502.1	18120	18234	+	114
NW_015822824.1	213867	214008	-	141
NW_015822824.1	212963	213087	-	124
NW_015822915.1	50798	50836	+	38
NW_015822943.1	9833	9943	-	110

NW_015823374.1	17012	17054	+	42
NW_015823637.1	46762	46825	-	63
NW_015823772.1	8481	8596	+	115
NW_015823838.1	12928	12982	-	54
NW_015823838.1	12918	13020	-	102
NW_015823840.1	75310	75451	-	141
NW_015823886.1	4506	4564	-	58
NW_015824054.1	155625	155741	+	116
NW_015824250.1	6948	7036	-	88
NW_015824250.1	6958	7029	-	71
NW_015824270.1	54212	54303	+	91
NW_015824270.1	54218	54297	+	79
NW_015824330.1	12637	12701	+	64
NW_015824421.1	71927	71977	-	50
NW_015824445.1	18219	18300	+	81
NW_015824500.1	23326	23433	-	107
NW_015824756.1	35465	35603	+	138
NW_015824769.1	9229	9273	+	44
NW_015824830.1	19729	19769	-	40
NW_015824926.1	4275	4346	+	71
NW_015824988.1	1745	1835	+	90
NW_015825133.1	127606	127689	-	83
NW_015825234.1	2049	2174	+	125
NW_015825287.1	4616	4743	-	127
NW_015825567.1	4239	4362	+	123

NW_015825634.1	6681	6757	+	64
NW_015825641.1	23980	24043	-	63
NW_015825712.1	31357	31416	-	59
NW_015825712.1	31366	31407	-	41
NW_015826233.1	17845	18013	+	168
NW_015826233.1	17821	18007	+	186
NW_015826421.1	15981	16115	-	134
NW_015826469.1	21520	21578	-	58
NW_015826561.1	1596	1778	-	182
NW_015826704.1	33721	33857	+	136
NW_015826799.1	69675	69758	+	83
NW_015827436.1	12114	12172	+	58
NW_015827689.1	75167	75382	-	215
NW_015827801.1	116321	116411	-	70
NW_015827850.1	9040	9144	-	104
NW_015827913.1	30792	30902	+	110
NW_015828131.1	26093	26141	+	48
NW_015828189.1	2649	2705	+	56
NW_015828272.1	28452	28722	-	270
NW_015828272.1	28558	28602	-	44
NW_015828413.1	9258	9360	-	102
NW_015828413.1	9511	9576	-	65
NW_015828505.1	33704	33768	-	64
NW_015828505.1	43492	43568	+	76
NW_015828634.1	45122	45205	+	83

NW_015828634.1	63391	63558	+	167
NW_015828926.1	11705	11916	-	211
NW_015829143.1	32447	32542	-	95
NW_015829262.1	44090	44225	-	135
NW_015829454.1	175684	175773	+	89
NW_015829454.1	175510	175733	+	223
NW_015829534.1	59363	59737	-	374
NW_015829643.1	11946	12069	+	123
NW_015829716.1	6978	7047	-	69
NW_015829783.1	15659	15920	-	261
NW_015829975.1	5067	5190	-	123
NW_015830040.1	19539	19610	+	71
NW_015830339.1	46924	47057	+	133
NW_015830448.1	59406	59520	+	114
NW_015830473.1	121375	121513	+	138
NW_015830513.1	22394	22524	-	130
NW_015830579.1	14089	14213	+	124
NW_015830873.1	24814	24945	+	108
NW_015831409.1	123340	123490	+	150
NW_015831538.1	33417	33515	-	98
NW_015831606.1	22240	22323	-	83
NW_015831638.1	42117	42226	+	109
NW_015831719.1	36630	36713	+	83
NW_015832017.1	74051	74177	-	126
NW_015832227.1	27589	27672	+	83

NW_015832684.1	12612	12675	+	63
NW_015832748.1	31798	31827	-	29
NW_015832833.1	25247	25328	+	81
NW_015833169.1	4375	4565	-	190
NW_015833169.1	4414	4512	-	98
NW_015833169.1	4377	4580	-	203
NW_015833195.1	3507	3597	+	90
NW_015833432.1	60125	60232	+	107
NW_015833460.1	48640	48714	-	74
NW_015833477.1	34805	34871	+	66
NW_015833564.1	39954	40126	-	172
NW_015833943.1	6068	6129	-	61
NW_015833943.1	6050	6128	-	78
NW_015833943.1	6056	6127	-	71
NW_015834182.1	18698	18751	-	53
NW_015834252.1	16242	16381	+	139
NW_015834252.1	16243	16379	+	136
NW_015834373.1	3118	3229	-	111
NW_015834423.1	29953	30092	+	139
NW_015834423.1	29952	30088	+	136
NW_015834495.1	55380	55510	+	130
NW_015834965.1	4184	4268	-	84
NW_015834981.1	52051	52117	+	66
NW_015835169.1	48190	48289	-	99
NW_015835254.1	63449	63660	-	211

NW_015835254.1	63413	63643	-	230
NW_015835299.1	11056	11261	-	205
NW_015835330.1	22073	22168	-	95
NW_015835420.1	8299	8393	-	94
NW_015835455.1	2828	2929	-	101
NW_015835466.1	5776	5896	-	120
NW_015835466.1	5764	5914	-	150
NW_015835530.1	17554	17689	-	129
NW_015835842.1	4928	4990	+	62
NW_015836007.1	7413	7473	-	60
NW_015836056.1	4605	4666	-	61
NW_015836154.1	57970	58025	-	55
NW_015836186.1	17167	17213	-	46
NW_015836367.1	31324	31379	+	55
NW_015836416.1	20938	20993	+	55
NW_015836416.1	20936	21028	+	92
NW_015836566.1	26328	26430	+	102
NW_015836609.1	36621	36719	+	98
NW_015836620.1	8686	8762	+	76
NW_015836620.1	8656	8764	+	102
NW_015836634.1	16975	17015	-	23
NW_015836718.1	16316	16422	+	106
NW_015836983.1	16029	16065	+	36
NW_015836983.1	27433	27494	-	61
NW_015837106.1	79891	79943	-	52

NW_015837106.1	79841	80006	-	165
NW_015837463.1	3408	3451	-	43
NW_015837463.1	3387	3462	-	75
NW_015837486.1	2095	2276	-	181
NW_015837486.1	2124	2281	-	157
NW_015837861.1	14626	14686	-	60
NW_015838024.1	21753	21822	-	69
NW_015838024.1	25417	25499	-	82
NW_015838024.1	21726	21813	-	87
NW_015838084.1	3895	3949	-	54
NW_015838084.1	3899	3954	-	55
NW_015838100.1	24458	24541	+	83
NW_015838488.1	7857	7989	-	132
NW_015838630.1	18044	18170	-	126
NW_015838732.1	9725	9873	-	148
NW_015838761.1	14410	14511	+	64
NW_015838911.1	24275	24332	-	57
NW_015839053.1	11024	11117	+	93
NW_015839053.1	11305	11473	+	168
NW_015839262.1	3627	3704	+	77
NW_015839309.1	5445	5501	+	56
NW_015839452.1	21080	21145	+	65
NW_015839479.1	85480	85537	-	57
NW_015839553.1	12617	12663	+	46
NW_015839553.1	12571	12662	+	91

NW_015839723.1	27076	27344	-	268
NW_015839723.1	27113	27354	-	241
NW_015840082.1	23829	23933	-	68
NW_015840200.1	17736	17786	+	50
NW_015840230.1	2466	2528	+	62
NW_015840282.1	113013	113145	+	132
NW_015840644.1	55205	55313	+	108
NW_015840657.1	7670	7745	+	75
NW_015840657.1	18692	18767	+	75
NW_015840758.1	142746	142983	+	237
NW_015840769.1	15580	15641	-	61
NW_015840789.1	4774	4867	-	93
NW_015840790.1	47781	48090	+	309
NW_015841055.1	1358	1566	+	208
NW_015841283.1	3493	3591	+	98
NW_015841390.1	10179	10270	+	78
NW_015841952.1	11011	11037	+	26
NW_015842213.1	117143	117199	-	56
NW_015842452.1	64506	64636	+	130
NW_015842637.1	85507	85677	-	170
NW_015842735.1	6631	6712	+	81
NW_015842749.1	129458	129511	-	53
NW_015842764.1	193782	193994	+	212
NW_015842764.1	193757	194027	+	270
NW_015842833.1	164077	164214	+	137

NW_015842948.1	1374	1536	+	162
NW_015842981.1	57441	57510	-	69
NW_015843003.1	106549	106719	-	170
NW_015843003.1	106602	106743	-	141
NW_015843003.1	106496	106735	-	239
NW_015843060.1	313	385	-	72
NW_015843060.1	278	401	-	123
NW_015843061.1	4417	4723	+	306
NW_015843290.1	5458	5534	-	76
NW_015843406.1	8221	8293	-	72
NW_015843805.1	8599	8702	+	103
NW_015843847.1	120209	120244	-	35
NW_015843929.1	7337	7403	+	66
NW_015844092.1	130955	131146	+	191
NW_015844165.1	110490	110553	+	63
NW_015844195.1	33118	33188	-	70
NW_015844286.1	51070	51151	+	81
NW_015844808.1	3907	4020	-	113
NW_015844978.1	8803	8862	-	59
NW_015845018.1	31776	31840	+	64
NW_015845424.1	11546	11612	+	66
NW_015845800.1	28872	28986	-	114
NW_015846331.1	15806	16109	-	303
NW_015846425.1	253433	253578	-	145
NW_015847004.1	71347	71478	+	131

NW_015847145.1	40378	40431	-	53
NW_015847249.1	20297	20405	-	108
NW_015847259.1	19791	19927	+	69
NW_015847612.1	13732	13881	+	96
NW_015847674.1	46130	46190	-	60
NW_015847675.1	5174	5298	+	124
NW_015847728.1	205404	205467	+	63
NW_015847855.1	58208	58388	+	180
NW_015848148.1	10686	10760	-	74
NW_015848468.1	9549	9728	-	179
NW_015848804.1	63588	63668	+	80
NW_015848846.1	75313	75505	-	192
NW_015849056.1	8365	8417	+	52
NW_015849632.1	27819	27978	+	159
NW_015849632.1	27810	28011	+	201
NW_015849728.1	18166	18259	+	93
NW_015849778.1	49630	49741	+	111
NW_015850191.1	157750	157784	+	34
NW_015850191.1	157901	157982	+	81
NW_015850267.1	33554	33678	+	124
NW_015850520.1	4433	4506	-	73
NW_015850570.1	4383	4657	+	274
NW_015850766.1	37041	37074	-	33
NW_015850890.1	2938	3104	-	166
NW_015850901.1	2108	2245	-	137

NW_015851005.1	32782	32848	-	66
NW_015851058.1	1506	1555	+	27
NW_015851138.1	36484	36657	+	173
NW_015851248.1	5537	5692	+	155
NW_015851491.1	11011	11128	-	46
NW_015851596.1	29563	29765	+	202
NW_015851701.1	21902	21951	-	49
NW_015851789.1	13323	13374	-	51
NW_015851810.1	22144	22439	-	295
NW_015852229.1	12942	12976	-	34
NW_015852267.1	5611	5749	+	138
NW_015852367.1	39016	39085	-	69
NW_015852473.1	16031	16118	+	87
NW_015852473.1	16026	16107	+	81
NW_015852649.1	37456	37600	-	25
NW_015852649.1	57514	57572	-	58
NW_015852740.1	66046	66096	+	50
NW_015852828.1	5693	5822	+	129
NW_015852828.1	5679	5821	+	142
NW_015852828.1	3651	3739	+	88
NW_015853028.1	8251	8373	+	122
NW_015853252.1	15355	15517	+	162
NW_015853392.1	5919	5943	-	24
NW_015853673.1	48397	48498	+	101
NW_015853696.1	7114	7203	+	74

NW_015853978.1	27833	27899	+	66
NW_015854074.1	4964	5196	+	232
NW_015854361.1	57824	57890	+	66
NW_015854397.1	7552	7786	-	234
NW_015854478.1	17488	17626	-	138
NW_015854573.1	38476	38581	-	68
NW_015854875.1	4268	4363	-	95
NW_015855200.1	1753	1851	-	98
NW_015855800.1	25640	25718	-	78
NW_015856093.1	95710	95833	+	123
NW_015856424.1	38706	38773	+	67
NW_015856503.1	37014	37156	-	142
NW_015856591.1	28825	28908	+	83
NW_015856591.1	28809	28914	+	105
NW_015856680.1	17213	17406	-	193
NW_015856680.1	17142	17401	-	259
NW_015857158.1	55438	55485	+	47
NW_015857372.1	5680	5798	-	72
NW_015857449.1	6567	6623	+	56
NW_015857525.1	268774	268850	-	76
NW_015857528.1	16198	16306	-	89
NW_015857996.1	12531	12669	+	138
NW_015858696.1	75499	75650	-	151
NW_015858724.1	57013	57038	+	25
NW_015858865.1	8874	9087	+	213

NW_015858865.1	8857	9105	+	248
NW_015858887.1	60829	60969	+	140
NW_015858887.1	59893	59963	+	70
NW_015858915.1	8667	8767	+	100
NW_015858944.1	42956	42998	+	42
NW_015858944.1	46133	46215	+	57
NW_015859152.1	7100	7149	+	49
NW_015859204.1	21804	21872	+	68
NW_015859285.1	14234	14294	+	60
NW_015859346.1	88062	88123	-	61
NW_015859544.1	17178	17437	-	259
NW_015859602.1	38549	38691	-	142
NW_015859602.1	38795	38904	-	109
NW_015859623.1	74160	74178	+	18
NW_015859913.1	17904	18047	-	143
NW_015860202.1	49525	49682	-	157
NW_015860285.1	50830	51118	+	255
NW_015860451.1	29540	29694	-	154
NW_015860585.1	20612	20715	+	103
NW_015860746.1	29412	29534	-	63
NW_015860891.1	114751	114807	+	56
NW_015861237.1	5253	5323	+	39
NW_015861237.1	5250	5452	+	116
NW_015861260.1	10167	10268	-	11
NW_015861265.1	15514	15617	-	103

NW_015861375.1	86710	86794	-	84
NW_015861405.1	10850	10932	+	82
NW_015861812.1	96442	96544	-	79
NW_015861812.1	96429	96557	-	92
NW_015861812.1	7217	7325	+	108
NW_015862386.1	29114	29178	+	64
NW_015862386.1	29085	29190	+	105
NW_015862386.1	29067	29241	+	174
NW_015862625.1	2612	2705	+	93
NW_015862625.1	2606	2696	+	90
NW_015862657.1	13344	13412	+	68
NW_015862803.1	53077	53109	+	32
NW_015863115.1	117974	118087	+	113
NW_015863135.1	33116	33174	+	58
NW_015863195.1	37922	38020	+	98
NW_015863248.1	29196	29222	-	26
NW_015863334.1	26758	26795	-	37
NW_015863334.1	26733	26831	-	98
NW_015863452.1	37755	37894	-	139
NW_015863524.1	93063	93166	-	103
NW_015863564.1	13040	13162	+	122
NW_015863580.1	2309	2394	-	85
NW_015863628.1	69872	70059	-	187
NW_015863628.1	69906	70050	-	144
NW_015863647.1	96692	96810	+	11

NW_015863661.1	1446	1574	-	128
NW_015863709.1	25246	25300	-	54
NW_015863803.1	57855	57878	+	23
NW_015863803.1	57848	57993	+	145
NW_015863803.1	57809	57999	+	190
NW_015864017.1	160683	160772	+	89
NW_015864017.1	130677	130786	+	104
NW_015864383.1	39937	40117	+	180
NW_015864383.1	39804	40083	+	279
NW_015864532.1	175003	175040	+	37
NW_015864719.1	330330	330417	+	87
NW_015864719.1	282197	282274	+	77
NW_015864846.1	36441	36519	+	78
NW_015864951.1	12944	12996	-	52
NW_015865063.1	16644	16719	-	75
NW_015865095.1	53637	53721	+	84
NW_015865282.1	166820	166916	-	96
NW_015865642.1	49329	49385	-	56
NW_015866069.1	29755	29880	+	125
NW_015866417.1	6237	6291	+	54
NW_015866417.1	6240	6389	+	149
NW_015866492.1	44666	44707	-	41
NW_015866573.1	99411	99485	-	74
NW_015866701.1	72709	72788	-	79
NW_015866820.1	46547	46604	-	57

NW_015866974.1	4291	4392	-	101
NW_015867070.1	6055	6157	+	102
NW_015867266.1	56726	56883	-	157
NW_015867277.1	15857	15916	+	59
NW_015867373.1	183025	183107	-	82
NW_015867373.1	2899	2935	-	36
NW_015867373.1	122317	122414	+	97
NW_015867373.1	183017	183096	-	79
NW_015867385.1	114992	115151	-	159
NW_015867414.1	22385	22421	-	36
NW_015867420.1	68948	69029	-	81
NW_015867717.1	134699	134781	-	49
NW_015868019.1	117707	117824	-	117
NW_015868053.1	189067	189207	-	140
NW_015868057.1	12121	12202	-	81
NW_015868129.1	4344	4439	+	95
NW_015868166.1	29967	30045	+	78
NW_015868319.1	16730	16856	+	82
NW_015868569.1	22164	22317	-	153
NW_015868903.1	34211	34322	-	111
NW_015868973.1	38019	38044	-	25
NW_015869052.1	108676	108735	-	59
NW_015869104.1	10437	10502	-	65
NW_015869342.1	15964	16099	+	135
NW_015869399.1	15676	15739	-	63

NW_015869403.1	4084	4168	-	84
NW_015869630.1	3538	3655	+	117
NW_015869630.1	3528	3641	+	113
NW_015869647.1	60282	60440	-	158
NW_015869674.1	69621	69771	+	108
NW_015869744.1	21727	21884	+	157
NW_015869744.1	67955	67986	+	31
NW_015870077.1	4523	4543	+	20
NW_015870077.1	4511	4573	+	62
NW_015870188.1	9435	9484	-	49
NW_015870340.1	31579	31665	-	86
NW_015870571.1	24631	24926	-	295
NW_015870571.1	133718	133793	+	75
NW_015870645.1	42915	43015	+	100
NW_015870775.1	37556	37645	+	89
NW_015870921.1	161988	162101	+	113
NW_015871032.1	2750	2859	-	109
NW_015871032.1	2744	2826	-	82
NW_015871337.1	3239	3343	-	104
NW_015871407.1	2724	2775	+	51
NW_015871411.1	7394	7567	+	173
NW_015871536.1	28647	28844	+	110
NW_015871653.1	46544	46627	-	83
NW_015871737.1	24050	24189	-	139
NW_015871821.1	26302	26428	-	126

NW_015871936.1	27585	27669	-	84
NW_015871965.1	51512	51648	+	136
NW_015871965.1	10681	10759	+	78
NW_015872426.1	15426	15597	-	171
NW_015872513.1	284	315	+	31
NW_015872592.1	5073	5155	-	82
NW_015872969.1	174741	174795	+	54
NW_015872981.1	128159	128285	+	126
NW_015872981.1	128036	128278	+	242
NW_015873053.1	28113	28202	+	74
NW_015873125.1	170956	171026	-	70
NW_015873125.1	170949	171029	-	80
NW_015873173.1	104893	104975	+	82
NW_015873203.1	55245	55317	+	72
NW_015873372.1	12506	12559	+	53
NW_015873761.1	51765	51839	-	74
NW_015873855.1	5047	5176	-	129
NW_015873964.1	5998	6062	+	64
NW_015874034.1	55303	55360	+	57
NW_015874183.1	9708	9778	+	70
NW_015874209.1	108947	109049	-	102
NW_015874616.1	56731	56810	-	79
NW_015874616.1	56707	56779	-	72
NW_015874746.1	20890	21086	+	196
NW_015874831.1	79849	79943	+	94

NW_015874831.1	79842	79997	+	155
NW_015875034.1	2131	2205	-	74
NW_015875147.1	97	212	+	115
NW_015875291.1	43116	43238	-	122
NW_015875291.1	31794	31908	+	114
NW_015875383.1	60206	60304	-	66
NW_015875387.1	9472	9526	+	54
NW_015875415.1	33249	33382	-	133
NW_015875415.1	30967	30996	-	29
NW_015875645.1	55196	55277	-	81
NW_015875645.1	55188	55333	-	145
NW_015875709.1	1855	2027	-	172
NW_015875746.1	15486	15502	-	16
NW_015876085.1	13246	13363	-	117
NW_015876273.1	22769	22830	-	61
NW_015876415.1	35726	35840	+	114
NW_015876443.1	4016	4074	-	58
NW_015876479.1	6888	6923	+	35
NW_015876645.1	4212	4474	+	262
NW_015876660.1	76376	76428	-	52
NW_015876668.1	27128	27194	-	66
NW_015876686.1	16400	16463	+	63
NW_015876807.1	66860	66898	+	38
NW_015876831.1	14945	15020	-	75
NW_015876940.1	12181	12294	-	113

NW_015877540.1	79352	79563	+	211
NW_015877628.1	34390	34472	+	82
NW_015878220.1	5409	5459	-	50
NW_015878239.1	85587	85672	+	85
NW_015878320.1	73834	74062	-	228
NW_015878836.1	7118	7178	+	60
NW_015879362.1	4595	4698	+	103
NW_015879766.1	3602	3697	-	95
NW_015879810.1	14538	14761	-	223
NW_015880039.1	1490	1548	-	58
NW_015880240.1	17770	17834	-	64
NW_015880302.1	42064	42133	-	69
NW_015880302.1	42023	42135	-	112
NW_015880354.1	12737	12813	-	76
NW_015880408.1	60154	60212	-	58
NW_015880582.1	19843	19916	+	73
NW_015880582.1	12236	12330	+	94
NW_015880857.1	76013	76074	+	61
NW_015881068.1	4714	4806	-	53
NW_015881121.1	112970	113087	+	117
NW_015881156.1	57155	57283	+	101
NW_015881359.1	13741	13899	-	158
NW_015881495.1	42231	42327	-	96
NW_015881706.1	6641	6760	-	119
NW_015881706.1	6645	6758	-	113

NW_015881734.1	43281	43493	+	212
NW_015881786.1	22783	22857	+	74
NW_015882197.1	6453	6592	-	70
NW_015882343.1	59447	59513	-	66
NW_015882452.1	48807	48854	+	47
NW_015882708.1	55238	55316	-	78
NW_015882749.1	6312	6446	-	134
NW_015882749.1	6314	6454	-	140
NW_015882881.1	13597	13795	+	198
NW_015882881.1	13601	13685	+	84
NW_015882918.1	2754	2871	+	117
NW_015882957.1	25560	25589	+	29
NW_015883405.1	2514	2553	-	39
NW_015883696.1	19077	19188	+	111
NW_015884150.1	193	287	+	94
NW_015884150.1	4630	4753	-	123
NW_015884425.1	3914	3951	+	37
NW_015884475.1	12105	12274	+	169
NW_015884635.1	46830	46953	-	123
NW_015884761.1	15197	15231	+	34
NW_015884818.1	2057	2237	+	180
NW_015884818.1	2034	2278	+	244
NW_015885141.1	25141	25288	-	147
NW_015885141.1	25157	25267	-	110
NW_015885152.1	119120	119227	-	107

NW_015885159.1	253246	253286	-	40
NW_015885206.1	19323	19399	-	76
NW_015885206.1	19326	19429	-	103
NW_015885244.1	35796	35865	+	69
NW_015885315.1	59619	59667	-	48
NW_015885320.1	53636	53702	+	66
NW_015885390.1	13176	13297	-	121
NW_015885396.1	122841	122893	-	52
NW_015885407.1	68505	68613	-	108
NW_015885604.1	6230	6518	+	288
NW_015885638.1	52853	53004	-	151
NW_015885681.1	3845	3939	+	94
NW_015885681.1	3592	3658	+	66
NW_015885740.1	24856	24981	+	125
NW_015885780.1	162370	162499	+	129
NW_015885814.1	23223	23348	-	125
NW_015885858.1	22980	23012	+	32
NW_015885903.1	49825	49907	-	82
NW_015885985.1	31408	31498	-	90
NW_015885985.1	32230	32470	-	240
NW_015886074.1	30709	30808	+	99
NW_015886163.1	31011	31072	-	61
NW_015886190.1	39704	39763	-	59
NW_015886209.1	14332	14382	+	50
NW_015886224.1	8621	8802	-	181

NW_015886241.1	20577	20716	+	139
NW_015886265.1	62590	62637	+	47
NW_015886265.1	62589	62613	+	24
NW_015886265.1	62581	62634	+	53
NW_015886316.1	3920	3979	+	59
NW_015886403.1	14526	14562	-	36
NW_015886405.1	52259	52313	-	54
NW_015886486.1	23859	23990	+	131
NW_015886540.1	25316	25413	+	97
NW_015886542.1	31503	31624	+	121
NW_015886717.1	17304	17397	+	93
NW_015886730.1	101995	102097	+	102
NW_015886732.1	15686	15761	-	75
NW_015886798.1	69528	69613	+	85
NW_015886864.1	92487	92541	-	54
NW_015886865.1	88145	88246	-	101
NW_015886988.1	140452	140483	+	31
NW_015886993.1	6738	6803	+	65
NW_015887022.1	25780	25802	-	22
NW_015887038.1	57797	57894	+	97
NW_015887111.1	89712	89803	-	91
NW_015887128.1	48896	48956	+	60
NW_015887133.1	209276	209387	+	111
NW_015887133.1	218528	218689	-	161
NW_015887170.1	6290	6379	+	89

NW_015887170.1	113885	113998	+	113
NW_015887291.1	236921	237164	-	243
NW_015887291.1	236927	237046	-	119
NW_015887350.1	3402	3665	+	263
NW_015887350.1	3063	3674	+	611
NW_015887416.1	10515	10570	-	55
NW_015887614.1	34685	34756	-	71
NW_015887671.1	67217	67320	+	103
NW_015887671.1	118301	118449	+	148
NW_015887671.1	67209	67493	+	284
NW_015887682.1	18001	18060	-	59
NW_015887687.1	6159	6362	-	203
NW_015887726.1	182040	182129	-	89
NW_015887726.1	182019	182154	-	135
NW_015887745.1	34551	34704	+	99
NW_015887819.1	20861	21111	-	250
NW_015887822.1	231976	232069	-	93
NW_015887838.1	61397	61446	+	49
NW_015887838.1	61386	61452	+	66
NW_015887920.1	69115	69151	+	36
NW_015888002.1	95240	95449	-	209
NW_015888002.1	76365	76433	-	68
NW_015888035.1	12634	12727	+	93
NW_015888119.1	35210	35280	-	70
NW_015888119.1	29768	29852	+	84

NW_015888133.1	13165	13263	-	98
NW_015888178.1	10853	10963	+	110
NW_015888225.1	18891	19016	+	125
NW_015888345.1	62701	62805	-	104
NW_015888392.1	84049	84077	-	28
NW_015888399.1	83353	83492	-	139
NW_015888421.1	9376	9452	-	10
NW_015888457.1	47295	47338	+	43
NW_015888457.1	47286	47426	+	140
NW_015888462.1	47474	47512	-	38
NW_015888540.1	44505	44668	-	163
NW_015888594.1	46405	46503	+	65
NW_015888618.1	51669	51745	+	76
NW_015888640.1	86151	86226	-	75
NW_015888663.1	6650	6759	+	109
NW_015888734.1	92112	92160	-	48
NW_015888734.1	92089	92166	-	77
NW_015888735.1	23621	23689	-	60
NW_015888746.1	30138	30203	-	65
NW_015888813.1	66653	66780	-	127
NW_015888848.1	47138	47172	+	34
NW_015888866.1	7942	8049	+	107
NW_015888938.1	16105	16208	+	103
NW_015888942.1	42094	42121	-	27
NW_015889038.1	98352	98471	-	119

NW_015889047.1	202992	203124	-	132
NW_015889091.1	34819	34896	-	77
NW_015889108.1	10397	10493	-	96
NW_015889108.1	10404	10501	-	97
NW_015889108.1	10399	10505	-	106
NW_015889163.1	93603	93746	-	143
NW_015889183.1	105855	105935	+	80
NW_015889233.1	7858	7888	+	30
NW_015889319.1	23295	23369	-	74
NW_015889364.1	17184	17324	-	140
NW_015889409.1	25747	25861	-	114
NW_015889753.1	80133	80209	+	76
NW_015889753.1	25493	25537	-	44
NW_015889778.1	7292	7356	-	64
NW_015889778.1	7258	7372	-	114
NW_015889799.1	42182	42288	-	106
NW_015889800.1	41801	41842	-	41
NW_015889806.1	39632	39736	+	104
NW_015889806.1	40236	40427	+	191
NW_015889806.1	39641	39740	+	99
NW_015889839.1	49346	49490	-	144
NW_015889878.1	46061	46130	-	69
NW_015889889.1	114540	114607	+	67
NW_015889899.1	20963	20989	-	26
NW_015889899.1	20946	20974	-	28

NW_015889918.1	37085	37145	-	60
NW_015890018.1	25654	25722	-	68
NW_015890023.1	21245	21341	+	96
NW_015890058.1	31916	32177	-	261
NW_015890095.1	72760	72798	+	38
NW_015890095.1	72763	72922	+	159
NW_015890136.1	61715	61833	+	118
NW_015890139.1	4048	4127	+	79
NW_015890218.1	234323	234378	-	55
NW_015890239.1	71631	71761	-	92
NW_015890453.1	27070	27164	+	94
NW_015890487.1	104206	104293	+	87
NW_015890530.1	18703	18847	-	144
NW_015890547.1	38121	38178	+	57
NW_015890579.1	63676	63790	-	114
NW_015890589.1	91777	91924	+	105
NW_015890766.1	135620	135724	-	104
NW_015890776.1	21175	21284	-	109
NW_015890856.1	46160	46203	+	43
NW_015890913.1	25459	25510	+	51
NW_015890936.1	79082	79104	-	22
NW_015890980.1	68009	68090	-	81
NW_015890992.1	195710	195782	-	72
NW_015890994.1	116366	116503	-	137
NW_015891048.1	24949	24993	+	44

NW_015891104.1	193335	193466	-	73
NW_015891143.1	12634	12794	-	160
NW_015891168.1	66439	66674	+	235
NW_015891168.1	66488	66687	+	199
NW_015891168.1	84470	84681	+	211
NW_015891178.1	69776	69952	+	176
NW_015891215.1	72644	72682	+	38
NW_015891269.1	58866	59021	+	155
NW_015891269.1	58813	59028	+	215
NW_015891273.1	60231	60296	-	65
NW_015891307.1	6391	6557	+	166
NW_015891307.1	6315	6570	+	255
NW_015891343.1	32554	32587	+	33
NW_015891369.1	46994	47129	-	135
NW_015891604.1	17527	17652	-	125
NW_015891625.1	26885	26970	-	85
NW_015891625.1	26878	27017	-	139
NW_015891636.1	18697	18816	-	119
NW_015891723.1	77953	78053	+	100
NW_015891723.1	79815	79892	+	77
NW_015891727.1	46212	46277	+	65
NW_015891738.1	39668	39717	-	35
NW_015891793.1	16595	16711	+	116
NW_015891850.1	3570	3726	+	156
NW_015891867.1	280323	280467	+	144

NW_015891867.1	280291	280436	+	145
NW_015891870.1	28880	29025	-	134
NW_015891933.1	13245	13404	+	159
NW_015891966.1	35423	35502	-	79
NW_015892219.1	16217	16308	-	91
NW_015892302.1	7574	7838	+	264
NW_015892302.1	7591	7673	+	82
NW_015892304.1	31592	31718	+	126
NW_015892338.1	11900	12051	+	151
NW_015892882.1	24243	24282	+	39
NW_015892882.1	24235	24292	+	57
NW_015893245.1	28501	28593	+	92
NW_015893710.1	221	341	+	73
NW_015894107.1	640	877	+	237
NW_015895536.1	28740	28862	-	122
NW_015895536.1	28741	28882	-	141
NW_015895592.1	46337	46426	-	89
NW_015896148.1	6434	6451	+	17
NW_015896652.1	3482	3572	+	90
NW_015896893.1	20089	20143	+	54
NW_015896893.1	20060	20188	+	128
NW_015897167.1	17053	17176	+	123
NW_015897426.1	2148	2261	-	113
NW_015897564.1	554	719	+	154
NW_015897627.1	92071	92152	-	81

NW_015897627.1	92057	92145	-	88
NW_015897824.1	8613	8686	+	73
NW_015897824.1	69023	69086	-	63
NW_015897905.1	274	455	+	181
NW_015897905.1	273	471	+	198
NW_015897911.1	44486	44646	+	160
NW_015898292.1	2329	2389	-	60
NW_015898386.1	536	649	+	113
NW_015898539.1	3116	3254	-	138
NW_015898642.1	174378	174488	+	110
NW_015898644.1	228	326	+	98
NW_015898674.1	2587	2812	+	225
NW_015898861.1	5318	5571	-	253
NW_015898913.1	36326	36389	+	63
NW_015898921.1	3890	4012	+	122
NW_015898942.1	1343	1606	+	263
NW_015898942.1	1327	1609	+	282
NW_015898951.1	5718	5782	+	64
NW_015898986.1	392	501	+	109
NW_015899006.1	6349	6442	-	93
NW_015899006.1	6135	6454	-	131
NW_015899060.1	19648	19718	+	70
NW_015899071.1	17759	17814	-	55
NW_015899113.1	32635	32699	-	64
NW_015899120.1	71646	71775	-	129

NW_015899124.1	170157	170217	+	60
NW_015899134.1	41271	41379	-	108
NW_015899228.1	12636	12721	-	85
NW_015899245.1	37617	37703	+	86
NW_015899301.1	4019	4069	-	50
NW_015899361.1	28197	28265	-	68
NW_015899361.1	57960	58042	+	82
NW_015899439.1	34198	34294	+	96
NW_015899443.1	107360	107420	+	60
NW_015899519.1	23164	23204	-	40
NW_015899519.1	23161	23231	-	70
NW_015899586.1	42278	42339	-	61
NW_015899637.1	104222	104287	-	65
NW_015899637.1	123594	123759	+	165
NW_015899644.1	19829	20032	-	203
NW_015899800.1	21376	21513	-	137
NW_015899881.1	29991	30059	-	68
NW_015899988.1	14893	14972	+	79
NW_015899998.1	12470	12585	+	79
NW_015900033.1	26637	26748	+	111
NW_015900085.1	56780	56869	+	89
NW_015900222.1	671	758	+	87
NW_015900318.1	113133	113207	-	74
NW_015900372.1	2490	2725	-	235
NW_015900434.1	18852	18916	-	64

NW_015900434.1	18850	18942	-	92
NW_015900449.1	78146	78265	+	119
NW_015900460.1	17988	18100	-	112
NW_015900476.1	64405	64512	+	107
NW_015900506.1	8100	8198	-	98
NW_015900538.1	98170	98334	-	164
NW_015900608.1	70105	70147	+	42
NW_015900638.1	74291	74363	+	72
NW_015900638.1	74304	74359	+	55
NW_015900681.1	71037	71115	+	78
NW_015900681.1	99171	99482	-	149
NW_015900715.1	46402	46546	+	144
NW_015900715.1	46407	46535	+	128
NW_015900944.1	16388	16440	-	52
NW_015900984.1	9065	9237	-	172
NW_015900987.1	16704	16796	+	92
NW_015901026.1	103663	103823	-	160
NW_015901029.1	65925	66024	+	99
NW_015901051.1	25211	25288	-	77
NW_015901126.1	149948	150167	-	219
NW_015901130.1	21178	21290	-	112
NW_015901153.1	195383	195435	-	52
NW_015901153.1	195371	195540	-	169
NW_015901240.1	124027	124208	+	142
NW_015901390.1	198699	198806	+	107

NW_015901464.1	6087	6130	+	43
NW_015901515.1	87154	87246	-	92
NW_015901515.1	87811	87862	-	51
NW_015901554.1	41908	41989	-	81
NW_015901597.1	16773	16857	+	84
NW_015901645.1	47456	47582	+	126
NW_015901688.1	78894	78957	-	63
NW_015901757.1	44846	44962	-	116
NW_015901798.1	14421	14486	+	65
NW_015901798.1	14445	14487	+	42
NW_015901798.1	14427	14564	+	137
NW_015901933.1	13168	13307	-	139
NW_015902072.1	38350	38441	+	91
NW_015902105.1	30268	30458	+	190
NW_015902155.1	42521	42606	+	85
NW_015902191.1	6284	6396	+	112
NW_015902213.1	50665	50845	+	180
NW_015902213.1	50661	50921	+	260
NW_015902266.1	15402	15462	-	60
NW_015902293.1	39880	39947	+	67
NW_015902311.1	16007	16110	+	103
NW_015902330.1	108553	108665	-	112
NW_015902345.1	135177	135222	+	45
NW_015902347.1	111164	111279	+	115
NW_015902389.1	34999	35082	+	83

NW_015902507.1	14299	14385	-	86
NW_015902526.1	23504	23618	+	114
NW_015902590.1	30890	31054	+	164
NW_015902605.1	33682	33715	-	33
NW_015902629.1	17638	17806	-	168
NW_015902638.1	69956	70226	+	270
NW_015902638.1	75497	75549	+	44
NW_015902638.1	75686	75849	+	163
NW_015902670.1	47453	47570	-	117
NW_015902845.1	2953	3115	+	162
NW_015902845.1	3030	3101	+	71
NW_015902988.1	49041	49132	-	91
NW_015903020.1	96857	96908	-	51
NW_015903099.1	32652	32736	-	84
NW_015903136.1	24078	24270	-	192
NW_015903184.1	14636	14721	-	85
NW_015903185.1	39844	39915	+	71
NW_015903294.1	19843	19925	+	82
NW_015903342.1	17755	17856	-	101
NW_015903372.1	46633	46736	+	103
NW_015903376.1	14814	14891	+	77
NW_015903404.1	37314	37471	-	157
NW_015903409.1	76244	76380	-	136
NW_015903459.1	16861	16997	+	136
NW_015903459.1	16842	17002	+	160

NW_015903481.1	56492	56567	-	75
NW_015903481.1	56484	56544	-	60
NW_015903497.1	29364	29518	-	154
NW_015903533.1	94770	94865	-	95
NW_015903536.1	50235	50413	-	178
NW_015903564.1	90765	90904	-	139
NW_015903564.1	90786	91100	-	314
NW_015903570.1	21067	21160	+	93
NW_015903570.1	21270	21351	+	81
NW_015903622.1	43520	43598	+	78
NW_015903630.1	28738	28847	-	109
NW_015903859.1	24432	24461	-	29
NW_015903864.1	13478	13529	+	51
NW_015903919.1	5755	5855	+	100
NW_015903924.1	21607	21649	-	42
NW_015903924.1	21599	21643	-	44
NW_015903932.1	10729	10827	-	98
NW_015903948.1	44784	44846	-	62
NW_015903976.1	36623	36723	-	100
NW_015904007.1	14274	14329	-	55
NW_015904048.1	37136	37197	+	61
NW_015904084.1	25542	25614	-	72
NW_015904084.1	25537	25632	-	95
NW_015904084.1	37265	37341	-	76
NW_015904094.1	29555	29688	-	133

NW_015904216.1	166614	166840	-	226
NW_015904250.1	189421	189554	+	133
NW_015904349.1	26893	26944	+	51
NW_015904370.1	149574	149721	-	147
NW_015904437.1	17145	17205	-	60
NW_015904478.1	44489	44662	-	173
NW_015904478.1	44623	44656	-	33
NW_015904568.1	23478	23574	-	96
NW_015904601.1	10961	11025	+	64
NW_015904671.1	13818	13974	-	156
NW_015904709.1	43832	43894	-	62
NW_015904717.1	69379	69528	-	149
NW_015904735.1	13838	13883	+	45
NW_015904735.1	13874	13982	+	108
NW_015904756.1	40486	40616	-	130
NW_015904761.1	23053	23147	+	94
NW_015904780.1	4439	4527	+	88
NW_015904799.1	15391	15484	-	93
NW_015904978.1	78522	78596	+	74
NW_015905084.1	43157	43210	-	53
NW_015905144.1	121921	122024	+	103
NW_015905170.1	189625	189675	+	50
NW_015905264.1	56829	56884	-	55
NW_015905269.1	55171	55236	+	65
NW_015905331.1	24386	24461	+	75

NW_015905331.1	24305	24509	+	204
NW_015905331.1	24356	24474	+	118
NW_015905344.1	21057	21113	+	56
NW_015905354.1	61947	62100	+	153
NW_015905376.1	18032	18126	+	94
NW_015905484.1	37036	37192	-	156
NW_015905488.1	45159	45222	+	63
NW_015905491.1	21203	21299	+	96
NW_015905505.1	28574	28690	+	116
NW_015905548.1	11320	11413	+	93
NW_015905548.1	12828	12952	+	124
NW_015905674.1	15507	15599	+	92
NW_015905712.1	75502	75534	+	32
NW_015905769.1	14893	14978	+	85
NW_015905783.1	92419	92452	+	33
NW_015905783.1	92417	92490	+	73
NW_015905783.1	78928	79082	+	154
NW_015905785.1	2923	2996	+	73
NW_015905801.1	28218	28267	+	49
NW_015905884.1	104765	104802	-	37
NW_015905901.1	30349	30427	-	78
NW_015905941.1	52978	53058	+	80
NW_015905954.1	50436	50576	+	140
NW_015906064.1	101400	101466	+	66
NW_015906196.1	1614	1687	+	73

NW_015906202.1	19692	19739	+	47
NW_015906217.1	8362	8464	-	102
NW_015906218.1	19339	19361	+	22
NW_015906218.1	21333	21498	+	144
NW_015906340.1	50255	50347	+	92
NW_015906340.1	50278	50358	+	80
NW_015906389.1	38403	38463	-	60
NW_015906389.1	38401	38483	-	82
NW_015906418.1	172231	172357	-	126
NW_015906418.1	176581	176653	-	72
NW_015906466.1	8530	8622	-	92
NW_015906536.1	4700	4948	+	248
NW_015906621.1	116528	116569	+	41
NW_015906621.1	116527	116578	+	51
NW_015906659.1	80979	81042	-	63
NW_015906688.1	265902	265994	-	92
NW_015906780.1	4810	4935	-	125
NW_015906791.1	31995	32092	-	97
NW_015906863.1	11398	11459	-	61
NW_015906863.1	11392	11479	-	87
NW_015906973.1	38799	38863	+	64
NW_015907062.1	33941	34012	-	71
NW_015907228.1	17818	17864	-	46
NW_015907237.1	70624	70684	+	60
NW_015907265.1	37139	37252	+	113

NW_015907282.1	58405	58446	-	41
NW_015907450.1	4878	4921	-	43
NW_015907460.1	46394	46432	+	38
NW_015907486.1	28719	28756	+	37
NW_015907496.1	24996	25166	+	170
NW_015907531.1	1536	1676	-	140
NW_015907531.1	1578	1828	-	250
NW_015907600.1	14168	14237	+	69
NW_015907602.1	10675	10815	-	80
NW_015907696.1	23097	23205	-	108
NW_015907711.1	4696	4721	-	25
NW_015907716.1	82778	82928	-	150
NW_015907765.1	84875	85045	+	170
NW_015907781.1	7072	7284	-	212
NW_015907803.1	102835	102906	-	71
NW_015907886.1	51178	51304	-	126
NW_015907940.1	82459	82512	+	53
NW_015907987.1	9046	9097	+	51
NW_015908135.1	38819	38920	-	101
NW_015908228.1	7703	7737	+	34
NW_015908240.1	685	720	+	35
NW_015908345.1	34143	34183	+	40
NW_015908347.1	44945	45092	+	147
NW_015908489.1	176111	176174	+	63
NW_015908589.1	94273	94335	+	62

NW_015908633.1	55285	55442	+	157
NW_015908633.1	37112	37208	-	96
NW_015908648.1	25616	25847	-	231
NW_015908672.1	16752	16841	+	17
NW_015908689.1	60077	60176	+	99
NW_015908689.1	60068	60187	+	119
NW_015908841.1	65441	65504	+	63
NW_015908841.1	65415	65553	+	138
NW_015908848.1	115089	115258	-	169
NW_015908859.1	94897	95014	-	117
NW_015908939.1	32034	32163	+	129
NW_015908950.1	22418	22461	-	43
NW_015908950.1	33013	33055	+	42
NW_015908950.1	103432	103545	-	113
NW_015908986.1	14678	14777	-	99
NW_015908986.1	14620	14810	-	190
NW_015909017.1	19611	19694	+	83
NW_015909017.1	19594	19681	+	87
NW_015909096.1	15397	15502	+	105
NW_015909155.1	71896	72001	-	105
NW_015909155.1	71917	71997	-	80
NW_015909169.1	109053	109105	+	52
NW_015909272.1	28454	28551	-	97
NW_015909272.1	28554	28823	-	269
NW_015909346.1	49797	49854	+	57

NW_015909346.1	49140	49229	+	89
NW_015909357.1	68958	69056	+	98
NW_015909362.1	53870	53963	+	93
NW_015909362.1	54323	54404	+	81
NW_015909389.1	114854	114915	+	61
NW_015909492.1	55059	55125	+	66
NW_015909533.1	32164	32292	+	128
NW_015909595.1	22444	22537	-	93
NW_015909610.1	13629	13817	+	188
NW_015909610.1	13655	13835	+	180
NW_015909681.1	15302	15470	-	168
NW_015909777.1	31507	31655	-	148
NW_015909848.1	52100	52260	-	160
NW_015909848.1	52001	52102	-	101
NW_015909951.1	28946	28995	-	49
NW_015909984.1	72876	72999	-	123
NW_015909994.1	66538	66606	-	68
NW_015910019.1	70101	70124	+	23
NW_015910130.1	3180	3331	+	151
NW_015910210.1	52589	52631	-	42
NW_015910210.1	52830	52922	-	92
NW_015910226.1	177531	177552	+	21
NW_015910226.1	28283	28373	+	90
NW_015910399.1	11802	11854	-	52
NW_015910399.1	11806	11870	-	64

NW_015910414.1	54102	54175	-	73
NW_015910566.1	109991	110050	-	59
NW_015910566.1	122438	122518	-	80
NW_015910566.1	109982	110043	-	61
NW_015910566.1	122419	122554	-	135
NW_015910710.1	31615	31630	-	15
NW_015910727.1	12139	12400	+	261
NW_015910735.1	80613	80659	-	46
NW_015910755.1	111682	111889	+	207
NW_015910852.1	10866	11080	+	214
NW_015910852.1	11874	11992	+	118
NW_015910898.1	16500	16539	+	39
NW_015910959.1	74610	74833	-	223
NW_015910959.1	74608	74867	-	259
NW_015910977.1	43132	43212	+	80
NW_015910990.1	8913	9077	+	164
NW_015911029.1	27697	28031	-	334
NW_015911139.1	7955	8229	-	242
NW_015911153.1	161894	162198	+	304
NW_015911314.1	52080	52150	-	70
NW_015911340.1	28132	28246	+	114
NW_015911352.1	32128	32277	+	149
NW_015911410.1	129659	129742	+	83
NW_015911500.1	57837	57906	-	69
NW_015911502.1	2860	2944	-	84

NW_015911561.1	22813	22971	+	89
NW_015911639.1	41054	41115	-	61
NW_015911639.1	41058	41117	-	59
NW_015911676.1	27777	27870	+	93
NW_015911677.1	44333	44449	-	116
NW_015911677.1	44352	44450	-	98
NW_015911677.1	44341	44462	-	121
NW_015911706.1	125855	125932	-	77
NW_015911743.1	126337	126440	+	103
NW_015911817.1	19866	19966	-	100
NW_015911817.1	19873	19977	-	104
NW_015911857.1	32436	32571	-	135
NW_015911953.1	19380	19512	-	112
NW_015911986.1	59315	59442	+	127
NW_015911992.1	15644	15692	-	48
NW_015912017.1	37280	37336	-	56
NW_015912050.1	37145	37293	+	148
NW_015912095.1	74918	75049	-	131
NW_015912097.1	67911	67983	-	72
NW_015912155.1	8704	8820	-	116
NW_015912224.1	7732	7770	+	38
NW_015912299.1	95774	95932	+	132
NW_015912382.1	33244	33373	-	129
NW_015912461.1	52882	52961	+	79
NW_015912473.1	37664	37818	+	154

NW_015912631.1	18567	18635	+	68
NW_015912631.1	18544	18625	+	81
NW_015912648.1	60391	60545	+	154
NW_015912668.1	30468	30562	-	94
NW_015912724.1	149774	149817	-	43
NW_015912765.1	4605	4702	-	97
NW_015912835.1	47805	47931	+	126
NW_015912909.1	26887	26903	-	16
NW_015912909.1	26864	26925	-	61
NW_015912920.1	41220	41308	+	88
NW_015912952.1	25535	25608	-	73
NW_015913302.1	21802	21857	+	46
NW_015913333.1	69865	69916	+	51
NW_015913333.1	13130	13194	-	64
NW_015913333.1	64872	64985	+	113
NW_015913333.1	13081	13221	-	140
NW_015913361.1	47008	47105	-	97
NW_015913362.1	138188	138229	-	41
NW_015913362.1	113959	114045	+	86
NW_015913397.1	257040	257134	-	94
NW_015913501.1	3882	4069	-	187
NW_015913653.1	44086	44168	-	82
NW_015913653.1	16567	16616	+	49
NW_015913738.1	30459	30519	-	60
NW_015913772.1	69421	69585	+	164

NW_015913846.1	167700	167854	-	154
NW_015913846.1	167724	167898	-	174
NW_015913846.1	12588	12667	-	79
NW_015913926.1	93001	93107	-	106
NW_015913926.1	92986	93104	-	118
NW_015913941.1	28221	28351	-	70
NW_015913941.1	28217	28360	-	79
NW_015913954.1	19943	20087	-	144
NW_015913993.1	14430	14504	-	74
NW_015914011.1	42835	42922	-	87
NW_015914025.1	37166	37245	+	79
NW_015914029.1	41426	41555	-	129
NW_015914045.1	14060	14202	-	142
NW_015914055.1	149287	149509	-	206
NW_015914055.1	149223	149521	-	218
NW_015914058.1	96562	96626	+	64
NW_015914067.1	7111	7151	+	40
NW_015914067.1	7026	7156	+	130
NW_015914088.1	10657	10723	-	66
NW_015914106.1	2097	2189	+	92
NW_015914121.1	65551	65766	+	215
NW_015914121.1	65550	65771	+	221
NW_015914121.1	65556	65781	+	225
NW_015914286.1	78794	78871	-	77
NW_015914301.1	18776	18884	-	108

NW_015914562.1	6138	6171	+	33
NW_015914758.1	114434	114555	-	121
NW_015914776.1	21696	21766	+	70
NW_015914861.1	1473	1541	+	68
NW_015914974.1	7751	7817	-	66
NW_015914974.1	7945	8019	-	74
NW_015915042.1	22869	22978	+	109
NW_015915055.1	49645	49703	+	58
NW_015915232.1	73768	73803	+	35
NW_015915279.1	5338	5439	+	53
NW_015915293.1	65364	65447	-	83
NW_015915346.1	24488	24525	-	37
NW_015915412.1	11152	11190	-	38
NW_015915476.1	35381	35440	+	59
NW_015915498.1	60774	60842	+	68
NW_015915503.1	5948	6011	+	63
NW_015915503.1	5951	6013	+	62
NW_015915562.1	38464	38567	+	103
NW_015915609.1	62231	62275	-	44
NW_015915612.1	3787	3847	-	60
NW_015915681.1	18146	18202	-	56
NW_015915700.1	19405	19551	+	146
NW_015915720.1	43936	44291	-	355
NW_015915722.1	85507	85554	+	47
NW_015915722.1	68177	68301	-	124

NW_015915811.1	33774	33990	+	216
NW_015915819.1	131272	131393	+	121
NW_015915893.1	37169	37286	+	117
NW_015915893.1	37173	37345	+	172
NW_015916059.1	56205	56297	+	92
NW_015916059.1	57142	57274	+	132
NW_015916070.1	65430	65478	+	48
NW_015916098.1	12650	12711	+	61
NW_015916170.1	21223	21367	-	144
NW_015916311.1	35711	35777	+	66
NW_015916319.1	55839	55989	+	150
NW_015916450.1	77403	77491	+	88
NW_015916474.1	11095	11413	-	285
NW_015916474.1	11088	11417	-	292
NW_015916498.1	56591	56727	-	136
NW_015916526.1	109318	109384	-	66
NW_015916546.1	81985	82029	-	44
NW_015916546.1	15776	15914	+	138
NW_015916596.1	32732	32814	-	82
NW_015916630.1	37072	37126	-	54
NW_015916713.1	6697	6811	+	114
NW_015916753.1	8781	8949	-	168
NW_015916768.1	20869	21009	+	140
NW_015916966.1	31420	31513	+	88
NW_015916966.1	31036	31049	+	13

NW_015917026.1	31750	31825	-	75
NW_015917044.1	12012	12151	+	139
NW_015917044.1	12010	12133	+	123
NW_015917081.1	38052	38167	+	115
NW_015917182.1	33083	33202	+	101
NW_015917211.1	90968	91042	+	74
NW_015917302.1	7267	7355	+	88
NW_015917466.1	7534	7664	+	130
NW_015917480.1	27266	27348	-	82
NW_015917544.1	7702	7777	+	75
NW_015917572.1	9018	9079	+	61
NW_015917596.1	59578	59664	-	86
NW_015917721.1	154275	154435	-	160
NW_015917758.1	78086	78156	+	70
NW_015917820.1	100404	100526	-	122
NW_015917988.1	131825	132009	-	184
NW_015918015.1	33140	33228	+	88
NW_015918060.1	37396	37495	+	99
NW_015918187.1	774	849	-	75
NW_015918243.1	27403	27544	+	141
NW_015918329.1	64462	64535	-	52
NW_015918335.1	6783	7014	+	231
NW_015918449.1	81032	81127	-	95
NW_015918466.1	19518	19641	+	123
NW_015918493.1	16569	16644	-	75

NW_015918519.1	27043	27120	+	77
NW_015918523.1	16555	16716	-	161
NW_015918523.1	17041	17136	-	95
NW_015918663.1	21262	21429	-	167
NW_015918718.1	71723	71762	+	39
NW_015918900.1	75210	75268	-	58
NW_015919075.1	19327	19450	-	107
NW_015919075.1	19248	19464	-	121
NW_015919083.1	11519	11620	+	101
NW_015919083.1	11532	11622	+	90
NW_015919089.1	36650	36777	+	127
NW_015919185.1	160027	160136	-	109
NW_015919220.1	66670	66749	-	79
NW_015919220.1	66661	66711	-	50
NW_015919243.1	79632	79701	-	69
NW_015919446.1	41307	41452	+	145
NW_015919519.1	68674	68741	+	67
NW_015919596.1	18535	18637	-	102
NW_015919596.1	18538	18654	-	116
NW_015919648.1	61183	61326	+	143
NW_015919660.1	48948	49089	-	141
NW_015919748.1	15956	16067	-	111
NW_015919748.1	16009	16102	-	93
NW_015919764.1	22652	22752	-	100
NW_015919773.1	30673	30752	+	79

NW_015919773.1	30669	30810	+	141
NW_015919779.1	4518	4601	+	83
NW_015919816.1	21942	22116	+	174
NW_015919861.1	48590	48686	-	96
NW_015919868.1	93004	93152	+	148
NW_015919953.1	30460	30515	+	55
NW_015919953.1	30109	30148	+	39
NW_015919954.1	47172	47634	-	462
NW_015919964.1	67151	67272	+	121
NW_015919969.1	18018	18107	+	89
NW_015920003.1	59508	59622	-	114
NW_015920005.1	16081	16173	-	92
NW_015920065.1	5118	5217	+	99
NW_015920137.1	35979	36051	-	72
NW_015920298.1	62601	62630	+	29
NW_015920380.1	17515	17622	-	107
NW_015920545.1	74158	74309	-	151
NW_015920546.1	62652	62716	-	64
NW_015920546.1	62501	62731	-	230
NW_015920667.1	18710	18863	-	153
NW_015920690.1	52065	52172	-	107
NW_015920789.1	47638	47713	-	75
NW_015920802.1	11855	12089	+	234
NW_015920827.1	38002	38078	-	76
NW_015920867.1	197421	197521	+	100

NW_015920870.1	8694	8748	+	47
NW_015920910.1	23812	23984	+	172
NW_015920924.1	5464	5546	-	82
NW_015921032.1	11823	11964	-	141
NW_015921100.1	2579	2726	-	147
NW_015921142.1	26032	26124	+	92
NW_015921164.1	4747	4796	+	49
NW_015921172.1	16113	16195	-	82
NW_015921366.1	29197	29329	-	132
NW_015921424.1	43699	43739	-	40
NW_015921443.1	15260	15410	+	150
NW_015921452.1	95930	95983	+	53
NW_015921526.1	29576	29646	-	44
NW_015921526.1	29513	29643	-	41
NW_015921551.1	6833	6945	+	112
NW_015921626.1	63158	63253	-	95
NW_015921636.1	40642	40682	+	40
NW_015921636.1	41212	41266	+	54
NW_015921636.1	41221	41289	+	68
NW_015921645.1	29634	29814	-	180
NW_015921660.1	52535	52623	-	88
NW_015921668.1	49861	49922	+	61
NW_015921676.1	44415	44477	-	62
NW_015921739.1	3103	3162	+	59
NW_015921940.1	8707	8772	-	65

NW_015922001.1	32857	32950	-	93
NW_015922044.1	11129	11214	-	85
NW_015922048.1	20439	20563	+	124
NW_015922079.1	11421	11481	+	60
NW_015922165.1	1746	1844	+	98
NW_015922165.1	8071	8164	-	93
NW_015922166.1	27969	28059	-	90
NW_015922346.1	17044	17084	+	40
NW_015922346.1	17042	17093	+	51
NW_015922419.1	36704	36838	+	49
NW_015922458.1	47458	47502	-	44
NW_015922458.1	47475	47508	-	33
NW_015922467.1	21937	22105	+	168
NW_015922467.1	21925	22009	+	84
NW_015922631.1	6280	6306	+	26
NW_015922631.1	6259	6337	+	78
NW_015922665.1	47606	47712	+	106
NW_015922693.1	21559	21630	+	71
NW_015922915.1	7065	7222	-	157
NW_015922992.1	14012	14136	-	124
NW_015922992.1	14004	14132	-	128
NW_015922992.1	13968	14128	-	160
NW_015923032.1	11172	11389	-	217
NW_015923089.1	1686	1750	+	64
NW_015923358.1	39940	40006	+	66

NW_015923358.1	39628	39716	+	88
NW_015923366.1	3476	3544	+	68
NW_015923373.1	22839	22925	+	86
NW_015923447.1	264643	264790	-	147
NW_015923454.1	43600	43670	+	70
NW_015923456.1	24305	24368	+	63
NW_015923456.1	59787	59892	-	52
NW_015923475.1	5116	5152	+	36
NW_015923483.1	12649	12772	-	123
NW_015923552.1	42706	42747	+	24
NW_015923642.1	4324	4381	+	57
NW_015923717.1	45528	45625	-	97
NW_015923906.1	9362	9403	-	41
NW_015924019.1	9451	9558	-	71
NW_015924032.1	14455	14633	-	178
NW_015924246.1	19937	20058	+	121
NW_015924319.1	108255	108355	-	100
NW_015924319.1	108249	108382	-	133
NW_015924319.1	18027	18059	-	32
NW_015924383.1	97323	97388	+	65
NW_015924383.1	97332	97395	+	63
NW_015924430.1	75436	75569	-	133
NW_015924442.1	123433	123517	-	84
NW_015924569.1	25275	25353	-	78
NW_015924571.1	2551	2578	+	27

NW_015924609.1	298	400	+	102
NW_015924647.1	70374	70610	+	236
NW_015924653.1	25350	25410	+	60
NW_015924653.1	25360	25701	+	341
NW_015924749.1	41430	41500	+	70
NW_015924776.1	19748	19839	+	91
NW_015924789.1	60716	60774	-	58
NW_015924819.1	30902	31086	+	184
NW_015924957.1	35092	35177	+	85
NW_015924957.1	35090	35150	+	60
NW_015924967.1	219718	219840	-	122
NW_015924977.1	16274	16331	+	57
NW_015925143.1	6508	6623	+	115
NW_015925224.1	21581	21639	+	58
NW_015925272.1	9549	9586	-	37
NW_015925290.1	83997	84113	+	116
NW_015925504.1	63941	64008	+	67
NW_015925504.1	63943	63980	+	37
NW_015925504.1	63933	64002	+	69
NW_015925570.1	88324	88581	+	257
NW_015925570.1	88319	88602	+	283
NW_015925580.1	25359	25417	-	58
NW_015925600.1	7248	7328	-	80
NW_015925632.1	31060	31136	+	76
NW_015925690.1	10668	10718	+	50

NW_015925697.1	117859	117982	+	123
NW_015925709.1	51251	51301	+	50
NW_015925749.1	110237	110498	-	261
NW_015925754.1	30342	30505	-	163
NW_015925760.1	18769	18912	+	143
NW_015925760.1	18254	18278	+	24
NW_015925781.1	53456	53556	+	100
NW_015925862.1	5174	5240	-	66
NW_015925999.1	20766	20858	+	92
NW_015925999.1	47683	47777	+	94
NW_015926091.1	22542	22748	+	206
NW_015926155.1	12066	12157	-	91
NW_015926317.1	12008	12058	-	50
NW_015926396.1	8204	8363	+	159
NW_015926470.1	41813	41951	-	114
NW_015926689.1	2374	2445	+	71
NW_015926741.1	4926	4993	+	67
NW_015926741.1	4578	4647	+	69
NW_015926837.1	17034	17091	-	57
NW_015926850.1	58615	58676	+	61
NW_015926875.1	5132	5151	+	19
NW_015926945.1	50470	50506	-	36
NW_015926945.1	50459	50527	-	68
NW_015926962.1	46700	46732	+	32
NW_015926966.1	9293	9391	-	98

NW_015927016.1	19727	19927	+	200
NW_015927030.1	4082	4158	+	76
NW_015927053.1	52664	52756	+	92
NW_015927118.1	23925	24098	+	141
NW_015927132.1	31458	31548	+	90
NW_015927176.1	2989	3177	+	188
NW_015927299.1	28686	28767	-	81
NW_015927404.1	91025	91107	+	82
NW_015927467.1	5268	5420	+	152
NW_015927481.1	57859	57985	+	126
NW_015927517.1	27082	27166	+	84
NW_015927568.1	4610	4715	+	105
NW_015927568.1	4613	4683	+	70
NW_015927575.1	4340	4482	+	130
NW_015927617.1	45252	45343	-	91
NW_015927625.1	28044	28192	-	148
NW_015927898.1	44927	45043	-	116
NW_015927982.1	14984	15092	-	108
NW_015928099.1	40493	40589	-	96
NW_015928107.1	16350	16463	-	113
NW_015928264.1	13830	13912	+	82
NW_015928315.1	104392	104432	+	40
NW_015928368.1	2938	3078	+	140
NW_015928428.1	135759	135839	+	80
NW_015928433.1	11772	11791	+	19

NW_015928832.1	172127	172182	-	55
NW_015928944.1	29881	29974	+	93
NW_015929181.1	5974	6203	+	229
NW_015929212.1	58668	58896	+	228
NW_015929226.1	27183	27252	-	69
NW_015929377.1	246870	246937	+	67
NW_015929427.1	27661	27741	-	80
NW_015929489.1	22604	22683	-	79
NW_015929563.1	138207	138262	-	55
NW_015929630.1	66378	66461	+	83
NW_015929777.1	8104	8194	+	90
NW_015929848.1	44611	44634	-	23
NW_015929865.1	15160	15387	-	227
NW_015929871.1	23750	23817	+	67
NW_015929894.1	18113	18196	+	83
NW_015929894.1	18105	18364	+	259
NW_015929960.1	15451	15503	-	52
NW_015930032.1	22405	22497	-	92
NW_015930045.1	69469	69581	+	112
NW_015930045.1	69434	69585	+	151
NW_015930076.1	64232	64276	-	44
NW_015930125.1	116694	116710	-	16
NW_015930395.1	6347	6454	+	107
NW_015930491.1	40438	40562	-	124
NW_015930491.1	40422	40582	-	160

NW_015930498.1	29561	29740	+	171
NW_015930588.1	57895	58068	+	173
NW_015930707.1	31783	31850	+	67
NW_015930828.1	7642	7710	-	68
NW_015930828.1	7656	7726	-	70
NW_015930843.1	21169	21260	+	91
NW_015930858.1	21688	21779	-	91
NW_015930915.1	48192	48377	+	185
NW_015931181.1	33437	33641	+	204
NW_015931221.1	32643	32696	+	53
NW_015931255.1	66602	66678	-	76
NW_015931294.1	22211	22422	-	211
NW_015931294.1	22288	22410	-	122
NW_015931325.1	6066	6157	-	91
NW_015931393.1	70834	70854	+	20
NW_015931489.1	32535	32801	-	266
NW_015931489.1	32539	32827	-	288
NW_015931526.1	36671	36763	+	92
NW_015931547.1	32728	32836	-	55
NW_015931583.1	49234	49362	+	79
NW_015931690.1	7793	7818	+	25
NW_015931842.1	5238	5451	-	213
NW_015931859.1	42484	42589	+	105
NW_015931882.1	12038	12114	-	76
NW_015931902.1	35995	36037	+	42

NW_015931902.1	35996	36029	+	33
NW_015931902.1	36013	36041	+	28
NW_015931959.1	17828	17880	+	52
NW_015932008.1	29534	29630	+	96
NW_015932131.1	45801	45905	-	104
NW_015932141.1	34364	34460	+	96
NW_015932141.1	34360	34783	+	423
NW_015932174.1	15083	15162	+	79
NW_015932287.1	48192	48330	+	138
NW_015932287.1	48121	48352	+	231
NW_015932296.1	116173	116255	+	82
NW_015932329.1	10530	10589	-	59
NW_015932381.1	12916	13033	+	95
NW_015932381.1	12920	13044	+	91
NW_015932410.1	267470	267611	-	141
NW_015932410.1	267516	267675	-	159
NW_015932470.1	30629	30759	-	130
NW_015932470.1	46391	46472	-	49
NW_015932609.1	26709	26774	-	65
NW_015932731.1	50100	50204	+	104
NW_015932765.1	8261	8284	+	23
NW_015932842.1	17891	17959	-	63
NW_015932988.1	44906	44957	+	51
NW_015933076.1	8123	8226	-	103
NW_015933085.1	35132	35260	-	128

NW_015933116.1	6022	6093	-	71
NW_015933116.1	5700	5818	-	118
NW_015933163.1	100546	100586	+	40
NW_015933234.1	9597	9847	-	250
NW_015933252.1	15608	15809	-	201
NW_015933253.1	2181	2328	+	147
NW_015933277.1	84108	84176	-	68
NW_015933280.1	169689	169993	-	304
NW_015933282.1	51936	52123	+	187
NW_015933313.1	3238	3309	+	71
NW_015933337.1	8883	8966	-	83
NW_015933343.1	33881	33990	-	109
NW_015933371.1	9131	9643	+	512
NW_015933371.1	45659	45761	+	102
NW_015933432.1	114100	114150	+	50
NW_015933512.1	11369	11568	-	199
NW_015933536.1	12296	12358	-	62
NW_015933715.1	64606	64716	-	110
NW_015933743.1	27243	27338	+	95
NW_015933743.1	39036	39066	-	18
NW_015933796.1	8757	8880	+	123
NW_015933796.1	8783	8874	+	91
NW_015933808.1	30873	30945	+	72
NW_015933808.1	30863	30973	+	110
NW_015933846.1	89907	89942	-	35

NW_015933858.1	53648	53772	-	124
NW_015933866.1	19849	19959	-	110
NW_015934006.1	53876	53948	+	72
NW_015934010.1	15460	15487	-	27
NW_015934124.1	26693	26764	+	71
NW_015934242.1	38212	38312	+	60
NW_015934288.1	93053	93113	-	60
NW_015934383.1	49504	49554	-	50
NW_015934637.1	8336	8443	+	107
NW_015934728.1	46025	46094	-	69
NW_015934728.1	45531	45809	-	278
NW_015934781.1	24015	24174	+	159
NW_015934787.1	77121	77178	+	57
NW_015934794.1	44998	45161	+	78
NW_015934914.1	42969	43104	+	135
NW_015934924.1	4155	4431	-	276
NW_015934933.1	121485	121568	+	83
NW_015935052.1	39042	39135	-	93
NW_015935085.1	48408	48491	+	83
NW_015935085.1	70582	70615	+	33
NW_015935230.1	5138	5410	+	272
NW_015935321.1	69721	69846	-	125
NW_015935321.1	69706	69867	-	161
NW_015935532.1	166276	166487	+	211
NW_015935532.1	166740	166810	+	70

NW_015935577.1	19209	19468	+	259
NW_015935578.1	12334	12481	+	147
NW_015935628.1	38115	38196	+	81
NW_015935659.1	4255	4323	-	68
NW_015935691.1	80294	80401	+	107
NW_015935691.1	80619	80667	+	48
NW_015935715.1	10104	10172	-	68
NW_015935759.1	18436	18635	-	199
NW_015935769.1	36641	36871	-	159
NW_015935875.1	18599	18629	+	30
NW_015935875.1	18561	18675	+	114
NW_015935936.1	36300	36416	+	116
NW_015935938.1	12771	12901	-	21
NW_015935988.1	21506	21654	+	148
NW_015935988.1	21357	21738	+	381
NW_015936024.1	61447	61472	-	25
NW_015936080.1	31781	31869	+	88
NW_015936089.1	49448	49575	+	127
NW_015936089.1	49432	49568	+	136
NW_015936241.1	46709	46904	+	195
NW_015936275.1	143878	143943	-	65
NW_015936275.1	159587	159705	-	118
NW_015936350.1	7464	7595	-	131
NW_015936350.1	7449	7491	-	42
NW_015936416.1	28593	28641	+	48

NW_015936416.1	28565	28648	+	83
NW_015936549.1	37866	37912	-	46
NW_015936596.1	42430	42620	+	190
NW_015936596.1	42569	42679	+	110
NW_015936598.1	37918	38096	+	178
NW_015936654.1	11502	11671	-	169
NW_015936725.1	11953	12069	+	116
NW_015936852.1	66669	66713	-	44
NW_015937016.1	16980	17085	-	105
NW_015937086.1	14692	14724	-	32
NW_015937099.1	36931	36975	+	44
NW_015937176.1	95022	95117	+	95
NW_015937264.1	12566	12622	+	56
NW_015937333.1	75434	75487	-	53
NW_015937348.1	21100	21247	-	89
NW_015937465.1	32350	32369	+	19
NW_015937585.1	13949	14028	-	79
NW_015937666.1	8887	9020	+	133
NW_015937723.1	14404	14472	-	68
NW_015937771.1	22913	22944	+	31
NW_015937783.1	23841	23913	-	72
NW_015937790.1	32809	32860	-	51
NW_015937940.1	6421	6591	-	170
NW_015937940.1	6457	6638	-	181
NW_015937951.1	7428	7480	-	52

NW_015938045.1	8424	8542	-	118
NW_015938092.1	29907	29954	+	47
NW_015938092.1	29880	29960	+	80
NW_015938099.1	2824	2917	-	93
NW_015938182.1	16892	16984	+	92
NW_015938327.1	81729	81859	-	130
NW_015938557.1	38510	38698	-	188
NW_015938557.1	38562	38728	-	166
NW_015938709.1	5112	5236	-	124
NW_015938775.1	57234	57297	+	63
NW_015938775.1	57243	57334	+	91
NW_015938892.1	1862	1913	+	51
NW_015938902.1	15061	15269	+	208
NW_015938902.1	15050	15235	+	185
NW_015938902.1	15408	15546	+	138
NW_015938930.1	83080	83183	+	103
NW_015938979.1	36469	36605	+	136
NW_015939093.1	15742	15820	-	78
NW_015939093.1	15755	15821	-	66
NW_015939093.1	15758	15841	-	83
NW_015939200.1	93860	93955	-	95
NW_015939246.1	14149	14265	-	116
NW_015939311.1	18866	18985	+	119
NW_015939311.1	13447	13516	+	69
NW_015939353.1	42426	42454	-	28

NW_015939353.1	42403	42457	-	54
NW_015939409.1	17442	17582	+	140
NW_015939676.1	18525	18551	-	26
NW_015939752.1	27133	27174	-	41
NW_015939812.1	94610	94651	+	41
NW_015939830.1	4656	4793	-	137
NW_015939934.1	22557	22620	-	63
NW_015939970.1	92155	92162	+	7
NW_015940213.1	34482	34630	+	148
NW_015940316.1	52655	52679	+	24
NW_015940333.1	134839	134891	+	52
NW_015940333.1	155356	155436	-	80
NW_015940427.1	2219	2335	+	116
NW_015940427.1	3145	3242	+	97
NW_015940444.1	4564	4694	+	120
NW_015940481.1	25084	25241	+	157
NW_015940632.1	156926	157092	+	166
NW_015940727.1	15662	15786	-	124
NW_015940826.1	32637	32708	-	71
NW_015940915.1	6007	6066	+	59
NW_015941028.1	17029	17144	+	115
NW_015941118.1	45399	45560	+	161
NW_015941221.1	17983	18056	-	73
NW_015941288.1	17172	17298	+	126
NW_015941516.1	51020	51063	-	43

NW_015941597.1	7147	7222	+	75
NW_015941628.1	1667	1757	+	90
NW_015941628.1	1668	1750	+	82
NW_015941676.1	105486	105564	-	78
NW_015941766.1	25389	25475	-	86
NW_015941789.1	29054	29098	-	44
NW_015941847.1	7535	7759	+	224
NW_015941915.1	16649	16834	+	163
NW_015941915.1	16686	16811	+	125
NW_015941949.1	6803	6913	-	110
NW_015942166.1	3810	3843	+	33
NW_015942170.1	16214	16278	+	64
NW_015942170.1	73223	73319	-	96
NW_015942243.1	11084	11174	+	90
NW_015942429.1	12541	12706	-	165
NW_015942448.1	8745	8819	-	74
NW_015942468.1	6686	6804	-	118
NW_015942531.1	45844	45913	-	69
NW_015942546.1	90	154	+	64
NW_015942608.1	25628	25696	+	68
NW_015942608.1	25610	25683	+	73
NW_015942759.1	22395	22491	+	96
NW_015942792.1	22575	22658	+	83
NW_015942886.1	12577	12615	+	38
NW_015943061.1	200333	200430	+	97

NW_015943089.1	26513	26604	-	91
NW_015943089.1	26523	26667	-	144
NW_015943181.1	10379	10524	-	145
NW_015943233.1	10737	10808	+	71
NW_015943297.1	10356	10452	+	96
NW_015943341.1	6004	6370	+	366
NW_015943355.1	3883	3997	+	114
NW_015943355.1	4930	5108	+	178
NW_015943355.1	3763	4025	+	262
NW_015943368.1	36951	37016	-	65
NW_015943412.1	632	791	-	122
NW_015943573.1	9224	9339	-	115
NW_015943599.1	73239	73334	+	95
NW_015943606.1	13333	13422	-	89
NW_015943682.1	322032	322146	-	114
NW_015943808.1	66423	66537	-	114
NW_015943812.1	2713	2878	+	165
NW_015943877.1	15959	16099	+	140
NW_015944000.1	21566	21640	-	71
NW_015944017.1	17675	17870	-	195
NW_015944096.1	28025	28245	-	220
NW_015944181.1	93481	93556	+	75
NW_015944309.1	12800	12935	-	135
NW_015944327.1	33868	33963	-	95
NW_015944340.1	5778	5832	-	54

NW_015944482.1	8527	8663	-	136
NW_015944710.1	109159	109253	-	94
NW_015944763.1	45385	45502	-	117
NW_015944851.1	13033	13200	+	167
NW_015944917.1	56170	56307	+	137
NW_015944917.1	57282	57413	+	131
NW_015945158.1	188670	188832	-	162
NW_015945193.1	6598	6714	+	116
NW_015945235.1	20262	20328	-	66
NW_015945235.1	20244	20313	-	69
NW_015945335.1	2575	2875	+	297
NW_015945464.1	40059	40134	+	75
NW_015945564.1	14917	15029	-	112
NW_015945827.1	6925	6991	+	66
NW_015945949.1	19481	19575	+	94
NW_015946040.1	75318	75378	-	60
NW_015946218.1	3617	3679	+	62
NW_015946357.1	2369	2503	-	134
NW_015946492.1	4681	4843	+	162
NW_015946492.1	7404	7493	+	89
NW_015946810.1	6397	6526	-	129
NW_015946810.1	6404	6515	-	111
NW_015946812.1	73240	73357	-	117
NW_015946812.1	73258	73378	-	120
NW_015946934.1	12473	12616	+	143

NW_015946952.1	6058	6177	+	119
NW_015946985.1	6980	7020	+	40
NW_015947005.1	25617	25662	-	45
NW_015947005.1	26334	26394	-	60
NW_015947063.1	48951	49116	-	165
NW_015947190.1	11555	11754	-	199
NW_015947265.1	9761	9783	-	22
NW_015947306.1	34680	34801	-	121
NW_015947448.1	3576	3946	+	370
NW_015947703.1	82724	82751	+	27
NW_015947703.1	83172	83233	+	61
NW_015947703.1	83165	83225	+	60
NW_015947703.1	83157	83236	+	79
NW_015947787.1	39851	39955	+	104
NW_015947801.1	24778	24859	-	81
NW_015947834.1	75513	75616	-	103
NW_015947850.1	7469	7614	-	145
NW_015947872.1	3651	3708	-	57
NW_015948010.1	4210	4283	+	73
NW_015948152.1	19258	19318	+	60
NW_015948156.1	38575	38687	+	112
NW_015948194.1	15954	16033	-	79
NW_015948200.1	18474	18577	-	103
NW_015948222.1	28788	28938	-	150
NW_015948283.1	7034	7102	+	68

NW_015948425.1	109180	109274	-	94
NW_015948435.1	43047	43153	+	106
NW_015948504.1	39086	39177	-	87
NW_015948505.1	34682	34792	-	110
NW_015948505.1	34719	35006	-	287
NW_015948672.1	23863	23992	+	129
NW_015948672.1	23860	23978	+	118
NW_015948700.1	4167	4278	-	111
NW_015948779.1	12973	13007	+	34
NW_015948966.1	12167	12215	+	41
NW_015949087.1	46369	46464	+	95
NW_015949221.1	15467	15512	-	45
NW_015949221.1	17558	17641	-	83
NW_015949397.1	130895	131001	-	56
NW_015949412.1	18984	19048	+	64
NW_015949438.1	141682	141734	-	52
NW_015949542.1	28652	29076	-	424
NW_015949578.1	4402	4539	-	137
NW_015949578.1	4404	4535	-	131
NW_015949603.1	15810	15844	+	34
NW_015949622.1	45033	45215	+	170
NW_015949652.1	22566	22633	-	67
NW_015949670.1	53650	53809	-	159
NW_015950109.1	1271	1417	-	146
NW_015950258.1	98879	99066	+	187

NW_015950259.1	186778	187057	-	171
NW_015950291.1	22595	22694	+	88
NW_015950374.1	43184	43283	+	99
NW_015950559.1	36644	36724	+	80
NW_015950597.1	3845	3921	-	76
NW_015950710.1	38674	38823	-	149
NW_015950710.1	38697	38820	-	123
NW_015950934.1	11443	11507	+	64
NW_015950934.1	74435	74607	-	172
NW_015950950.1	16462	16549	+	87
NW_015950986.1	8555	8656	+	101
NW_015951006.1	5391	5623	+	232
NW_015951085.1	16582	16678	-	80
NW_015951222.1	170332	170373	-	41
NW_015951453.1	22988	23035	+	47
NW_015951690.1	12301	12450	+	149
NW_015951912.1	45527	45671	+	134
NW_015951945.1	78436	78605	-	95
NW_015952033.1	18878	18994	+	116
NW_015952033.1	18862	18978	+	116
NW_015952097.1	39781	39845	+	64
NW_015952432.1	66312	66418	-	106
NW_015952523.1	14773	14844	-	71
NW_015952616.1	10281	10555	+	274
NW_015952863.1	19145	19483	+	250

NW_015952919.1	10211	10341	-	130
NW_015952919.1	10200	10360	-	160
NW_015953320.1	12687	12776	+	89
NW_015953320.1	12677	12786	+	109
NW_015953369.1	33687	33724	+	37
NW_015953397.1	92017	92248	-	231
NW_015953492.1	12306	12368	+	62
NW_015953492.1	12710	12805	+	95
NW_015953580.1	37790	37939	+	149
NW_015953607.1	27712	27857	+	145
NW_015953755.1	7914	8080	-	166
NW_015953756.1	16618	16668	-	50
NW_015953774.1	21674	21751	-	77
NW_015953785.1	45472	45489	+	17
NW_015953888.1	51673	51871	-	198
NW_015953989.1	36092	36121	-	29
NW_015953989.1	17683	17715	+	32
NW_015954264.1	56261	56445	-	184
NW_015954336.1	13180	13234	+	54
NW_015954336.1	13166	13267	+	101
NW_015954448.1	24632	24683	-	51
NW_015954448.1	24639	24729	-	90
NW_015954522.1	18013	18112	-	99
NW_015954560.1	146947	147026	+	79
NW_015954584.1	13097	13194	+	97

NW_015954747.1	35055	35133	+	78
NW_015954748.1	4679	4753	+	74
NW_015954809.1	48150	48210	-	60
NW_015954809.1	48145	48207	-	62
NW_015954833.1	8771	8848	-	77
NW_015954833.1	8774	8858	-	84
NW_015954833.1	8767	8839	-	72
NW_015954833.1	9050	9140	-	90
NW_015954901.1	30144	30212	-	68
NW_015955156.1	145304	145454	-	150
NW_015955234.1	37655	37760	-	105
NW_015955364.1	19683	19778	-	95
NW_015955408.1	16647	16722	-	75
NW_015955427.1	13009	13156	-	147
NW_015955427.1	12563	12629	-	66
NW_015955427.1	12993	13161	-	168

16days post inoculation (Hyper-DMRs)

Scaffold	Start	End	Region stain	Overlapped site
NW_015787321.1	11415	11564	-	149
NW_015787444.1	12034	12162	+	70
NW_015787709.1	12838	12867	+	25
NW_015788036.1	66948	67090	+	142
NW_015788476.1	22944	22962	-	18

NW_015789135.1	35816	35981	-	165
NW_015789235.1	4043	4217	+	174
NW_015789235.1	3835	4227	+	213
NW_015789325.1	21640	21751	+	111
NW_015789325.1	21626	21742	+	116
NW_015789335.1	80093	80207	+	114
NW_015789335.1	80062	80256	+	194
NW_015789514.1	19773	19885	-	112
NW_015789913.1	41450	41556	-	106
NW_015789913.1	41454	41679	-	225
NW_015789933.1	71075	71262	+	187
NW_015790340.1	49049	49125	+	76
NW_015790612.1	43362	43450	-	68
NW_015790646.1	48972	49021	+	49
NW_015790688.1	22514	22744	-	230
NW_015790688.1	22525	22720	-	195
NW_015790688.1	22420	22770	-	350
NW_015790901.1	207657	207738	-	81
NW_015790901.1	207625	207735	-	110
NW_015791371.1	30404	30579	-	76
NW_015791379.1	5756	6050	+	294
NW_015791453.1	128883	129018	-	118
NW_015791453.1	128908	129034	-	93
NW_015791475.1	11620	11661	-	41
NW_015791678.1	28523	28641	+	118

NW_015792667.1	60519	60593	-	74
NW_015792667.1	61218	61511	-	60
NW_015792667.1	61225	61526	+	301
NW_015792667.1	61213	61530	+	317
NW_015792755.1	13352	13559	-	207
NW_015792876.1	60419	60543	-	124
NW_015792876.1	60184	60549	-	365
NW_015792972.1	31772	31867	+	95
NW_015792972.1	30584	30701	+	117
NW_015792972.1	31765	31888	+	123
NW_015793025.1	38034	38202	+	168
NW_015793025.1	38024	38180	+	156
NW_015793290.1	32380	32464	+	84
NW_015793290.1	32386	32478	+	92
NW_015793546.1	24749	24992	-	243
NW_015793546.1	24696	24998	-	302
NW_015793644.1	17465	17518	-	53
NW_015793678.1	8893	8953	+	60
NW_015793782.1	5413	5504	-	91
NW_015794452.1	11058	11146	+	84
NW_015794452.1	11049	11166	+	104
NW_015794475.1	148536	148737	-	201
NW_015794475.1	149143	149267	-	124
NW_015794475.1	149150	149274	-	124
NW_015794615.1	5406	5514	-	108

NW_015794615.1	5414	5649	-	235
NW_015794667.1	1387	1417	+	30
NW_015794770.1	33656	33870	+	214
NW_015794999.1	219530	219613	+	83
NW_015795225.1	33529	33722	+	193
NW_015795225.1	33520	33779	+	259
NW_015795341.1	10443	10658	-	119
NW_015795619.1	2760	2887	-	127
NW_015795761.1	40542	40673	+	131
NW_015795854.1	27929	27989	+	60
NW_015796003.1	17035	17142	-	107
NW_015796236.1	26035	26114	+	79
NW_015796236.1	26024	26128	+	104
NW_015796295.1	14603	14734	-	131
NW_015796422.1	46115	46264	-	149
NW_015796422.1	46109	46285	-	176
NW_015796427.1	1497	1690	+	193
NW_015796427.1	1491	1700	+	209
NW_015796444.1	46254	46565	-	311
NW_015796596.1	6553	6641	-	88
NW_015796781.1	14725	14837	+	112
NW_015796812.1	16728	16864	-	136
NW_015797243.1	39771	39929	-	158
NW_015797243.1	39760	39901	-	141
NW_015797342.1	138983	139086	-	103

NW_015797342.1	138971	139101	-	130
NW_015797568.1	46286	46452	+	166
NW_015797568.1	46123	46450	+	327
NW_015797574.1	128461	128612	-	151
NW_015797574.1	128450	128607	-	157
NW_015797574.1	128453	128598	-	145
NW_015798007.1	2223	2377	-	154
NW_015798079.1	21878	22037	-	159
NW_015798126.1	17594	17643	-	49
NW_015798126.1	17564	17662	-	98
NW_015798126.1	17543	17650	-	107
NW_015798173.1	40157	40307	+	150
NW_015798463.1	49540	49788	+	171
NW_015799056.1	48398	48544	-	146
NW_015799056.1	48378	48558	-	180
NW_015799308.1	8578	8719	-	141
NW_015799362.1	88255	88405	-	150
NW_015799362.1	83084	83163	+	79
NW_015799362.1	83063	83143	-	80
NW_015799362.1	88243	88575	-	332
NW_015799362.1	83058	83157	+	99
NW_015799589.1	26664	26741	-	77
NW_015799589.1	26663	26742	-	79
NW_015799589.1	26662	26750	-	88
NW_015799868.1	25479	25587	+	108

NW_015800658.1	14364	14532	-	168
NW_015800658.1	14360	14610	-	250
NW_015801146.1	10342	10503	-	161
NW_015801457.1	9352	9420	-	68
NW_015801684.1	35527	35635	+	108
NW_015801758.1	68492	68534	-	42
NW_015801865.1	39191	39265	+	74
NW_015802527.1	12556	12586	-	30
NW_015802646.1	112998	113113	-	115
NW_015803246.1	30188	30287	+	99
NW_015803246.1	30160	30381	+	221
NW_015803498.1	25817	25870	-	53
NW_015803759.1	1754	1833	+	79
NW_015803759.1	1738	1846	+	108
NW_015803836.1	1689	1753	+	64
NW_015803836.1	1549	1888	+	241
NW_015803881.1	14540	14639	-	99
NW_015803990.1	11283	11352	+	69
NW_015804174.1	16003	16062	+	59
NW_015804174.1	16002	16063	+	61
NW_015804735.1	53604	53681	+	77
NW_015804735.1	53577	53676	+	99
NW_015804894.1	110434	110664	+	230
NW_015804904.1	73382	73469	+	87
NW_015805110.1	21695	21834	-	139

NW_015805288.1	16526	16741	+	215
NW_015805344.1	13935	14048	-	113
NW_015805344.1	13862	14069	-	207
NW_015805359.1	11316	11414	-	98
NW_015805359.1	11201	11436	-	235
NW_015805407.1	7577	7708	-	131
NW_015805407.1	7515	7978	-	463
NW_015805657.1	39265	39386	-	121
NW_015805910.1	62945	63019	-	74
NW_015806082.1	167033	167053	+	20
NW_015806601.1	104037	104087	+	50
NW_015806798.1	31432	31644	-	212
NW_015806798.1	29747	29783	-	36
NW_015807090.1	12811	12874	+	63
NW_015807649.1	10179	10295	-	116
NW_015807747.1	3796	3829	+	33
NW_015807834.1	26327	26388	+	61
NW_015808372.1	21494	21660	-	166
NW_015808403.1	15043	15146	+	103
NW_015808479.1	1961	2061	-	100
NW_015808490.1	31527	31647	+	120
NW_015808490.1	31510	31640	+	130
NW_015808490.1	31514	31651	+	137
NW_015808745.1	27689	27792	-	103
NW_015808811.1	35532	35779	+	247

NW_015809102.1	11197	11254	-	57
NW_015809459.1	26230	26539	+	309
NW_015809867.1	16474	16589	+	115
NW_015809867.1	16472	16582	+	110
NW_015809920.1	16818	16958	+	140
NW_015810190.1	11836	11977	-	141
NW_015810838.1	21597	21891	+	294
NW_015810923.1	19429	19541	-	112
NW_015810989.1	62506	62630	+	124
NW_015811149.1	15443	15563	+	120
NW_015811179.1	20207	20357	-	105
NW_015811434.1	60896	60965	+	69
NW_015811553.1	3006	3076	-	70
NW_015811553.1	2999	3250	-	251
NW_015811614.1	3897	4134	-	237
NW_015812697.1	18385	18508	-	123
NW_015812697.1	18379	18505	-	126
NW_015812800.1	58695	58788	-	93
NW_015813270.1	10644	11087	-	443
NW_015813270.1	5640	5689	+	49
NW_015813270.1	10639	11099	-	460
NW_015813573.1	20063	20132	+	69
NW_015814106.1	14480	14670	+	190
NW_015814787.1	46102	46175	-	73
NW_015814787.1	46091	46164	-	73

NW_015814787.1	46086	46184	-	98
NW_015815456.1	12297	12456	-	159
NW_015815476.1	80395	80508	-	113
NW_015815476.1	80358	80509	-	151
NW_015815515.1	27565	27681	-	116
NW_015816087.1	26615	26689	-	74
NW_015816087.1	26557	26683	-	126
NW_015816087.1	26877	27100	+	223
NW_015816487.1	17706	17910	-	204
NW_015816700.1	14565	14686	-	121
NW_015816849.1	23869	23949	+	80
NW_015816896.1	23618	23782	-	164
NW_015817414.1	10567	10609	+	33
NW_015817414.1	10477	10623	+	98
NW_015818172.1	16694	16740	+	46
NW_015818840.1	56145	56197	+	52
NW_015819657.1	4831	5030	+	199
NW_015819883.1	16977	17007	-	30
NW_015820244.1	17811	18046	+	235
NW_015820647.1	13763	13777	-	14
NW_015821239.1	322733	322982	-	249
NW_015821418.1	15227	15270	+	43
NW_015822259.1	37585	37781	-	196
NW_015822259.1	37666	37804	-	138
NW_015822259.1	37659	37805	-	146

NW_015823840.1	81767	81940	-	173
NW_015824007.1	35391	35510	-	119
NW_015824270.1	54218	54298	+	80
NW_015824399.1	111571	111684	+	113
NW_015824421.1	71730	71808	-	78
NW_015824421.1	71923	71980	-	57
NW_015824421.1	71729	71992	-	263
NW_015824421.1	71735	71977	-	242
NW_015824475.1	115424	115504	-	80
NW_015824514.1	6219	6418	+	199
NW_015824538.1	12389	12499	-	110
NW_015824765.1	4916	5140	-	224
NW_015824877.1	40217	40269	+	52
NW_015825712.1	31366	31567	-	201
NW_015826290.1	25348	25536	-	188
NW_015826290.1	25367	25535	-	168
NW_015826290.1	25339	25526	-	187
NW_015826295.1	12353	12446	+	93
NW_015826406.1	97242	97367	+	125
NW_015826406.1	97169	97377	+	208
NW_015826561.1	1344	1778	-	281
NW_015826758.1	23780	23936	+	156
NW_015827184.1	102328	102486	+	158
NW_015827184.1	102322	102469	+	147
NW_015827540.1	69230	69365	+	135

NW_015828032.1	55655	55750	-	95
NW_015828863.1	11865	11917	-	52
NW_015829361.1	25670	25740	-	70
NW_015830225.1	11070	11280	+	210
NW_015830388.1	53647	53742	-	95
NW_015830579.1	14044	14213	+	169
NW_015831798.1	60079	60213	-	134
NW_015831798.1	60073	60216	-	143
NW_015832017.1	48878	48985	+	107
NW_015832645.1	38352	38490	-	138
NW_015832748.1	27591	27760	-	169
NW_015832833.1	16604	16816	+	212
NW_015832916.1	59719	59794	+	75
NW_015833681.1	106439	106500	+	61
NW_015834645.1	16535	16673	-	138
NW_015835254.1	63398	63664	-	266
NW_015835713.1	232	379	-	147
NW_015835713.1	231	377	-	146
NW_015835713.1	234	384	-	150
NW_015836303.1	13987	14125	+	138
NW_015836353.1	32858	32898	+	40
NW_015836783.1	340716	340901	+	185
NW_015837315.1	81590	81755	+	165
NW_015837463.1	3394	3451	-	57
NW_015837463.1	3385	3462	-	77

NW_015838004.1	261024	261224	-	200
NW_015838891.1	16539	16595	+	56
NW_015838991.1	45696	45953	+	161
NW_015839770.1	11384	11478	-	94
NW_015840034.1	12485	12564	+	79
NW_015840034.1	12261	12786	+	525
NW_015840234.1	41970	42097	-	127
NW_015840275.1	4782	4842	-	60
NW_015840275.1	4774	5010	-	236
NW_015840282.1	112672	112793	+	121
NW_015840657.1	13445	13505	+	60
NW_015840758.1	142736	142975	+	239
NW_015840790.1	24926	25184	+	258
NW_015840790.1	26251	26299	+	48
NW_015840790.1	26454	26478	+	24
NW_015840790.1	24948	25187	+	239
NW_015840790.1	26262	26321	+	59
NW_015840790.1	24932	25194	+	262
NW_015840790.1	26225	26336	+	111
NW_015840908.1	52349	52467	-	118
NW_015840931.1	56270	56312	+	42
NW_015841106.1	57219	57331	-	112
NW_015841117.1	8732	8931	-	199
NW_015841117.1	8681	8938	-	257
NW_015841921.1	435	460	+	25

NW_015842217.1	49748	49841	+	93
NW_015842217.1	49739	49847	+	108
NW_015842290.1	28405	28760	-	355
NW_015842290.1	28473	28764	-	291
NW_015842290.1	28467	28774	-	307
NW_015843537.1	34298	34364	-	66
NW_015843537.1	34304	34359	-	55
NW_015844660.1	15507	15519	+	12
NW_015846045.1	5826	5922	+	96
NW_015846045.1	6036	6194	+	158
NW_015846305.1	4425	4491	-	66
NW_015846305.1	4606	4660	-	54
NW_015846331.1	52576	52613	+	37
NW_015847145.1	49466	49576	+	110
NW_015847145.1	49426	49588	+	162
NW_015847259.1	21435	21791	+	356
NW_015847894.1	116157	116201	-	44
NW_015848121.1	50263	50371	+	108
NW_015848816.1	56956	57014	+	58
NW_015849252.1	62490	62612	+	122
NW_015849520.1	7070	7176	+	42
NW_015849664.1	59718	59775	+	13
NW_015850380.1	6405	6462	+	57
NW_015850448.1	180946	180991	+	45
NW_015850766.1	17183	17347	-	164

NW_015851210.1	23323	23491	+	168
NW_015851210.1	23172	23507	+	335
NW_015851625.1	19830	19912	+	82
NW_015851661.1	14026	14152	-	126
NW_015852649.1	10749	10812	+	63
NW_015852649.1	10732	10846	+	114
NW_015853108.1	31983	32163	-	180
NW_015853298.1	44899	45060	-	161
NW_015853527.1	69778	69830	-	52
NW_015853580.1	1950	2201	-	251
NW_015853692.1	28373	29146	-	773
NW_015853692.1	28361	29143	-	782
NW_015853874.1	40508	40584	+	76
NW_015854112.1	33595	33706	+	111
NW_015854284.1	132432	132481	-	49
NW_015854334.1	6219	6241	+	22
NW_015854679.1	1700	2008	-	308
NW_015854679.1	1705	1999	-	294
NW_015854679.1	1691	2012	-	321
NW_015855565.1	87094	87268	-	174
NW_015855617.1	23813	24003	-	188
NW_015856308.1	10071	10257	-	186
NW_015856419.1	4073	4236	+	163
NW_015857996.1	20895	21044	+	149
NW_015857996.1	20875	21046	+	171

NW_015858043.1	79442	79621	+	179
NW_015858103.1	26063	26141	-	78
NW_015858103.1	26066	26144	-	78
NW_015858294.1	18101	18272	+	171
NW_015858593.1	2938	3051	-	113
NW_015858696.1	77135	77245	-	110
NW_015859129.1	5488	5559	-	71
NW_015859307.1	368	411	-	43
NW_015860220.1	10151	10246	-	95
NW_015860220.1	10054	10275	-	221
NW_015860800.1	5117	5151	-	34
NW_015860800.1	5120	5190	-	70
NW_015860976.1	1943	2193	+	250
NW_015860976.1	1937	2191	+	254
NW_015860976.1	1925	2202	+	277
NW_015861076.1	15088	15133	-	45
NW_015861798.1	13535	13575	+	40
NW_015861840.1	1900	2023	+	123
NW_015861840.1	1770	2048	+	212
NW_015861909.1	10556	10656	+	100
NW_015861909.1	10533	10700	+	167
NW_015862209.1	190784	190875	+	91
NW_015862391.1	24349	24370	-	21
NW_015862824.1	3279	3428	+	149
NW_015862830.1	153748	153835	+	87

NW_015863298.1	37362	37756	+	394
NW_015863431.1	3722	3885	-	163
NW_015863832.1	12404	12479	+	75
NW_015863832.1	12396	12486	+	90
NW_015864975.1	43301	43518	+	217
NW_015865914.1	21581	21744	-	114
NW_015865933.1	108381	108459	+	78
NW_015865933.1	108371	108474	+	103
NW_015866069.1	48480	48669	-	187
NW_015866173.1	128537	128622	-	85
NW_015866492.1	48675	48823	-	148
NW_015866508.1	251219	251484	-	265
NW_015866547.1	5393	5585	+	192
NW_015866916.1	1753	1887	-	134
NW_015867252.1	20723	20824	+	101
NW_015867252.1	20735	20817	+	82
NW_015867266.1	56639	56878	-	239
NW_015867266.1	56599	56883	-	284
NW_015867465.1	26106	26192	-	86
NW_015867465.1	26100	26196	-	96
NW_015867717.1	134698	134784	-	50
NW_015868068.1	40721	40868	+	147
NW_015868073.1	13484	13524	-	40
NW_015868079.1	30573	30654	-	81
NW_015868090.1	63452	63500	-	48

NW_015868090.1	63455	63495	-	40
NW_015868471.1	33344	33395	-	51
NW_015868471.1	33352	33406	-	54
NW_015868471.1	33347	33412	-	65
NW_015868569.1	22210	22379	-	169
NW_015868612.1	225402	225567	-	165
NW_015869325.1	46423	46487	-	64
NW_015869579.1	19137	19256	+	119
NW_015870461.1	49706	49859	+	153
NW_015870635.1	7302	7329	-	27
NW_015871750.1	36496	36653	+	157
NW_015871750.1	36466	36656	+	190
NW_015873125.1	23333	23425	+	92
NW_015875187.1	30581	30745	-	164
NW_015875913.1	95058	95163	+	105
NW_015877516.1	24234	24339	+	105
NW_015877845.1	4417	4552	+	135
NW_015878008.1	52667	52810	+	143
NW_015878239.1	96453	96569	-	116
NW_015878239.1	96464	96641	-	177
NW_015878239.1	96456	96652	-	196
NW_015878289.1	85034	85227	+	193
NW_015878484.1	11271	11500	+	229
NW_015879256.1	20580	20767	-	187
NW_015879684.1	23104	23211	-	107

NW_015879810.1	11873	11951	-	78
NW_015880178.1	20086	20168	-	82
NW_015880230.1	64823	64948	-	125
NW_015880571.1	64395	64580	-	185
NW_015880571.1	64407	64581	-	174
NW_015880571.1	64393	64605	-	212
NW_015881916.1	117525	117566	-	41
NW_015882221.1	22584	22704	+	120
NW_015882221.1	22570	22702	+	132
NW_015884356.1	2305	2371	+	66
NW_015884356.1	2270	2357	+	87
NW_015884635.1	47072	47212	-	140
NW_015884635.1	46824	47234	-	410
NW_015884980.1	4145	4351	-	100
NW_015885356.1	84408	84518	-	110
NW_015885378.1	44954	45128	-	174
NW_015885604.1	6067	6518	+	451
NW_015885604.1	5849	6527	+	678
NW_015885681.1	3900	3959	+	59
NW_015885681.1	3592	3956	+	364
NW_015885750.1	162502	162643	-	113
NW_015885827.1	9666	9701	+	35
NW_015886247.1	31488	31583	+	95
NW_015886285.1	27386	27446	+	60
NW_015886487.1	43812	43940	+	128

NW_015886487.1	48675	48906	-	231
NW_015886670.1	173640	173713	+	73
NW_015886670.1	173589	173724	+	135
NW_015886706.1	67936	68046	+	110
NW_015886756.1	71945	72137	-	192
NW_015886756.1	71951	72305	-	354
NW_015886798.1	75211	75294	+	83
NW_015886799.1	108958	109146	+	188
NW_015886799.1	109269	109375	+	106
NW_015886799.1	109249	109368	+	119
NW_015886799.1	108726	109386	+	660
NW_015886821.1	14425	14490	+	65
NW_015886840.1	21896	22097	-	201
NW_015886840.1	21929	22090	-	161
NW_015887066.1	13337	13531	-	194
NW_015887133.1	217983	218277	-	294
NW_015887133.1	217970	218689	-	719
NW_015887133.1	82415	82551	-	106
NW_015887232.1	66038	66150	+	112
NW_015887604.1	368178	368411	+	233
NW_015887604.1	368169	368265	+	96
NW_015887604.1	368151	368274	+	123
NW_015887651.1	121483	121553	+	70
NW_015887651.1	121486	121543	+	57
NW_015887671.1	118465	118601	+	136

NW_015887671.1	118209	118551	+	342
NW_015887671.1	118185	118610	+	425
NW_015887734.1	4645	4998	+	258
NW_015887752.1	107806	107856	+	50
NW_015888020.1	3631	3850	-	219
NW_015888020.1	3709	3843	-	134
NW_015888340.1	232694	232745	-	51
NW_015888346.1	135269	135396	+	127
NW_015888361.1	2066	2195	-	129
NW_015888361.1	2045	2201	-	156
NW_015888419.1	7874	8038	-	164
NW_015888419.1	7870	8029	-	159
NW_015888421.1	7860	8002	-	95
NW_015888425.1	30688	30807	-	102
NW_015888425.1	57761	58151	-	390
NW_015888447.1	141654	141794	-	140
NW_015888531.1	11591	11663	-	72
NW_015888572.1	29781	29885	+	104
NW_015888581.1	39682	39773	+	91
NW_015888581.1	39951	40258	+	307
NW_015888581.1	39676	39774	+	98
NW_015888581.1	39472	39824	+	352
NW_015888894.1	16619	16735	+	116
NW_015888894.1	16613	16718	+	105
NW_015888977.1	36831	36871	+	40

NW_015889053.1	18783	18979	-	196
NW_015889184.1	25131	25338	-	207
NW_015889258.1	68225	68302	-	77
NW_015889258.1	68166	68376	-	210
NW_015889302.1	28149	28229	+	80
NW_015889779.1	13106	13204	+	98
NW_015889779.1	13105	13223	+	118
NW_015889800.1	41794	41840	-	46
NW_015889819.1	14454	14583	-	129
NW_015889819.1	14492	14567	-	75
NW_015889819.1	14456	14609	-	153
NW_015889858.1	37497	37656	+	116
NW_015890018.1	620	664	-	44
NW_015890221.1	13435	13500	-	65
NW_015890221.1	21512	21583	-	71
NW_015890322.1	30907	31056	-	94
NW_015890470.1	25317	25422	-	105
NW_015890885.1	21767	21845	-	78
NW_015890885.1	21954	22109	-	155
NW_015890885.1	21736	22120	-	384
NW_015891054.1	1768	1848	-	80
NW_015891256.1	18578	18749	-	97
NW_015891257.1	18548	18575	+	27
NW_015891356.1	13631	14176	+	545
NW_015891490.1	34948	35033	-	85

NW_015891636.1	60021	60262	+	241
NW_015891952.1	10464	10604	+	140
NW_015891966.1	35420	35476	-	56
NW_015892102.1	45278	45354	-	76
NW_015892123.1	26226	26341	+	115
NW_015892253.1	91239	91285	+	46
NW_015892253.1	91168	91301	+	133
NW_015892254.1	26981	27136	-	155
NW_015892309.1	96055	96215	+	160
NW_015892309.1	96024	96219	+	195
NW_015893570.1	55	90	+	35
NW_015894087.1	15116	15245	+	129
NW_015894675.1	17320	17693	-	373
NW_015894860.1	41602	41755	+	153
NW_015894954.1	19	147	-	128
NW_015895371.1	58337	58537	-	200
NW_015895787.1	62628	62761	+	133
NW_015895787.1	62632	62788	+	156
NW_015897470.1	115	267	-	152
NW_015897470.1	81	348	-	267
NW_015898069.1	450	615	-	165
NW_015898292.1	1363	1520	-	157
NW_015898709.1	2310	2451	+	141
NW_015898709.1	2833	3012	+	179
NW_015898709.1	2306	2468	+	162

NW_015898709.1	2802	3051	+	249
NW_015898864.1	5129	5316	+	187
NW_015898932.1	7541	7677	+	136
NW_015899054.1	53388	53452	-	64
NW_015899443.1	112384	112470	+	86
NW_015899445.1	161335	161366	-	31
NW_015899650.1	18484	18532	+	48
NW_015899867.1	41206	41279	-	73
NW_015900222.1	671	758	+	87
NW_015900322.1	20807	21003	+	196
NW_015900341.1	62356	62420	-	64
NW_015900506.1	8126	8197	-	71
NW_015900667.1	50981	51071	-	90
NW_015900681.1	71051	71136	+	85
NW_015900681.1	71037	71126	+	89
NW_015900923.1	48397	48533	+	136
NW_015900935.1	32246	32327	+	81
NW_015900939.1	24264	24554	+	290
NW_015900939.1	24211	24564	+	353
NW_015901044.1	100532	100684	-	152
NW_015901153.1	157702	157907	-	75
NW_015901237.1	6941	6999	-	58
NW_015901493.1	12459	12565	+	106
NW_015901537.1	22812	22975	+	163
NW_015901537.1	22829	22978	+	149

NW_015901537.1	22806	22985	+	179
NW_015901544.1	16510	16553	-	43
NW_015901544.1	15884	15959	-	75
NW_015901544.1	16495	16580	-	85
NW_015901586.1	77594	77729	-	135
NW_015901586.1	77602	77759	-	157
NW_015901586.1	34589	34695	-	106
NW_015901933.1	154649	154695	-	46
NW_015902311.1	16385	16488	+	103
NW_015902493.1	112585	112664	-	79
NW_015902614.1	15210	15325	-	115
NW_015902670.1	39171	39370	-	199
NW_015902801.1	15302	15428	+	126
NW_015902804.1	33149	33227	+	78
NW_015902872.1	11093	11228	-	135
NW_015902993.1	21641	21654	+	13
NW_015902993.1	21556	21655	+	99
NW_015902993.1	21553	21639	+	86
NW_015903090.1	14848	14881	-	33
NW_015903281.1	63620	63752	+	132
NW_015903294.1	8723	8804	+	81
NW_015903342.1	14342	14484	-	142
NW_015903342.1	14330	14581	-	251
NW_015903380.1	45260	45371	+	111
NW_015903497.1	29323	29504	-	181

NW_015903564.1	91071	91105	-	34
NW_015903630.1	28741	28858	-	117
NW_015903797.1	126747	126907	+	160
NW_015904042.1	2035	2182	+	147
NW_015904042.1	2076	2177	+	101
NW_015904284.1	12194	12251	-	57
NW_015904370.1	55349	55439	-	90
NW_015904444.1	31944	32211	+	267
NW_015904444.1	31903	32205	+	302
NW_015904445.1	117220	117372	-	152
NW_015904445.1	117209	117401	-	192
NW_015904462.1	101012	101105	-	93
NW_015904478.1	44172	44280	-	108
NW_015904481.1	5484	5525	+	41
NW_015904724.1	42010	42142	-	132
NW_015904724.1	42009	42135	-	126
NW_015904735.1	13630	14016	+	364
NW_015905254.1	18146	18293	-	147
NW_015905309.1	65498	65587	-	89
NW_015905309.1	65472	65584	-	112
NW_015905344.1	21057	21405	+	348
NW_015905476.1	48831	48904	+	73
NW_015905954.1	50625	50739	+	114
NW_015905954.1	50436	50746	+	310
NW_015905982.1	24035	24191	-	156

NW_015906190.1	107560	107775	-	215
NW_015906221.1	43825	43905	+	80
NW_015906418.1	172215	172357	-	142
NW_015906752.1	131488	131577	+	89
NW_015906788.1	39636	40063	+	427
NW_015906932.1	14140	14276	-	90
NW_015906973.1	38771	38985	+	214
NW_015907125.1	78073	78235	+	84
NW_015907237.1	71708	71983	+	275
NW_015907237.1	71704	72204	+	500
NW_015907331.1	25432	25487	+	55
NW_015907489.1	26829	26920	+	91
NW_015907636.1	126937	127015	-	78
NW_015907655.1	29663	29772	-	109
NW_015907655.1	29632	29773	-	141
NW_015907765.1	85338	85627	+	289
NW_015907765.1	85457	85544	+	87
NW_015907838.1	71063	71168	+	105
NW_015907838.1	71083	71304	+	221
NW_015908411.1	129059	129258	+	199
NW_015908598.1	45473	45565	+	92
NW_015908643.1	119759	119959	-	200
NW_015908944.1	8829	9035	-	206
NW_015908944.1	8824	9025	-	201
NW_015908995.1	240971	241166	+	195

NW_015909023.1	87474	87533	-	59
NW_015909339.1	6163	6215	+	52
NW_015909339.1	6123	6274	+	151
NW_015909339.1	6119	6291	+	172
NW_015909430.1	24571	24634	-	63
NW_015909533.1	32193	32573	+	380
NW_015909533.1	32164	32506	+	342
NW_015909536.1	41702	41799	+	97
NW_015909536.1	41438	41793	+	355
NW_015909633.1	70998	71126	+	128
NW_015909837.1	47580	47621	-	41
NW_015909837.1	47585	47638	-	53
NW_015909862.1	15508	15592	+	84
NW_015910019.1	70908	71012	+	104
NW_015910130.1	3114	3289	+	175
NW_015910200.1	26233	26321	+	88
NW_015910200.1	26218	26332	+	114
NW_015910288.1	43104	43168	-	64
NW_015910409.1	32423	32573	+	150
NW_015910429.1	131154	131286	+	132
NW_015910518.1	52239	52406	-	167
NW_015910957.1	47174	47459	-	285
NW_015911029.1	6473	6590	-	117
NW_015911271.1	45364	45524	+	160
NW_015911271.1	45358	45518	+	160

NW_015911274.1	10954	11177	-	223
NW_015911274.1	11402	11508	-	106
NW_015911444.1	17552	17625	+	73
NW_015911502.1	2737	2947	-	210
NW_015911585.1	75415	75467	-	52
NW_015911585.1	75413	75490	-	77
NW_015911612.1	22371	22625	+	254
NW_015911652.1	40777	40932	-	155
NW_015912263.1	7627	7822	-	195
NW_015912288.1	55948	56201	-	253
NW_015912382.1	12625	12672	-	47
NW_015912564.1	82325	82392	+	67
NW_015912564.1	82326	82385	+	59
NW_015912564.1	82336	82398	+	62
NW_015912607.1	51472	51689	+	217
NW_015912648.1	69946	70077	+	131
NW_015912724.1	139802	139932	+	130
NW_015912753.1	7115	7191	+	76
NW_015912753.1	7089	7200	+	111
NW_015912758.1	167568	167656	+	53
NW_015912773.1	79957	80050	-	93
NW_015912817.1	55729	55766	+	37
NW_015912962.1	133246	133338	+	92
NW_015912962.1	133245	133341	+	96
NW_015912962.1	133248	133344	+	96

NW_015912989.1	3401	3514	+	113
NW_015913653.1	43257	43535	-	278
NW_015913653.1	43268	43538	-	270
NW_015913779.1	21572	21724	+	152
NW_015914011.1	42698	42927	-	229
NW_015914227.1	7873	7962	+	89
NW_015914227.1	7740	8005	+	265
NW_015914235.1	50909	51008	-	99
NW_015914650.1	44077	44134	-	57
NW_015914776.1	21717	21743	+	26
NW_015914908.1	91268	91490	-	222
NW_015914974.1	7698	7817	-	119
NW_015915082.1	136198	136338	+	114
NW_015915509.1	20395	20638	+	243
NW_015915509.1	31996	32168	-	172
NW_015915553.1	42658	42666	+	8
NW_015915562.1	21237	21443	+	206
NW_015915687.1	44629	44772	+	143
NW_015915705.1	22582	22614	+	32
NW_015915722.1	90905	91135	+	230
NW_015915768.1	5137	5269	+	132
NW_015916189.1	66309	66391	-	82
NW_015916238.1	38477	38692	-	215
NW_015916238.1	38467	38681	-	214
NW_015916664.1	2080	2268	+	188

NW_015916664.1	2119	2246	+	127
NW_015916664.1	2073	2286	+	213
NW_015916678.1	30203	30331	+	128
NW_015916827.1	25004	25032	+	28
NW_015917091.1	29290	29485	-	191
NW_015917104.1	53019	53092	+	73
NW_015917104.1	52964	53090	+	126
NW_015917424.1	46954	47004	+	50
NW_015917489.1	7320	7396	+	76
NW_015917701.1	15365	15586	-	221
NW_015917774.1	1548	1695	-	147
NW_015917774.1	1527	1699	-	172
NW_015917816.1	24205	24244	-	39
NW_015917816.1	24147	24257	-	110
NW_015918107.1	9535	9624	-	89
NW_015918248.1	46840	46969	-	129
NW_015918388.1	67976	68150	-	174
NW_015918495.1	13991	14119	-	128
NW_015918551.1	40757	40874	+	117
NW_015918760.1	32443	32533	-	90
NW_015918936.1	44580	44710	+	71
NW_015919061.1	77809	77987	+	178
NW_015919061.1	78202	78326	+	124
NW_015919061.1	77456	77953	+	497
NW_015919061.1	78157	78319	+	162

NW_015919160.1	2538	2662	-	124
NW_015919290.1	4492	4584	+	92
NW_015919290.1	4488	4579	+	91
NW_015919290.1	20762	20807	+	45
NW_015919343.1	13686	13784	+	98
NW_015919343.1	13683	13791	+	108
NW_015919395.1	29136	29265	+	129
NW_015919603.1	92037	92220	-	183
NW_015919812.1	40721	40828	-	107
NW_015919812.1	40710	40958	-	248
NW_015920043.1	24965	25039	-	74
NW_015920043.1	24830	25049	-	219
NW_015920181.1	168862	168946	+	84
NW_015920181.1	168835	168943	+	108
NW_015920181.1	168840	168953	+	113
NW_015920380.1	17517	17609	-	92
NW_015920494.1	36497	36627	+	85
NW_015920676.1	70304	70545	+	241
NW_015920854.1	81855	81918	+	63
NW_015920997.1	58063	58115	-	52
NW_015921105.1	88464	88607	-	143
NW_015921262.1	14025	14094	-	69
NW_015921466.1	79882	80024	-	142
NW_015921570.1	23820	23866	+	46
NW_015921570.1	23828	23939	+	111

NW_015921570.1	23822	24029	+	207
NW_015921832.1	49379	49483	-	104
NW_015921874.1	14439	14629	-	190
NW_015922020.1	6342	6417	-	75
NW_015922140.1	77742	77897	-	155
NW_015922466.1	24616	24721	-	105
NW_015922501.1	93757	93902	-	145
NW_015922501.1	115916	115935	-	19
NW_015922873.1	25017	25204	-	187
NW_015923011.1	25964	26148	-	184
NW_015923011.1	25967	26160	-	193
NW_015923032.1	10782	10849	-	67
NW_015923094.1	25996	26076	+	80
NW_015923094.1	34957	35013	+	56
NW_015923095.1	42563	42648	+	85
NW_015923095.1	42516	42629	+	113
NW_015923106.1	44558	44627	-	69
NW_015923366.1	3476	3768	+	292
NW_015923366.1	3485	3747	+	262
NW_015923366.1	3449	3772	+	323
NW_015923454.1	43609	43719	+	110
NW_015923475.1	16282	16413	-	131
NW_015923475.1	16273	16457	-	184
NW_015923524.1	49303	49385	-	82
NW_015923544.1	8195	8358	-	163

NW_015923902.1	17201	17318	+	117
NW_015923902.1	17174	17323	+	149
NW_015923998.1	11662	11700	-	38
NW_015924300.1	5949	5999	+	50
NW_015924300.1	5956	6014	+	58
NW_015924743.1	69545	69675	-	130
NW_015924743.1	69540	69760	-	220
NW_015924798.1	3734	3859	-	125
NW_015924847.1	31102	31137	+	35
NW_015925049.1	49219	49353	+	134
NW_015925049.1	49111	49341	+	230
NW_015925470.1	13300	13535	+	235
NW_015925470.1	13302	13564	+	262
NW_015925470.1	13267	13943	+	676
NW_015925684.1	49512	49554	-	42
NW_015925772.1	114229	114352	+	123
NW_015926003.1	119697	119750	-	53
NW_015926401.1	35639	35784	-	145
NW_015926401.1	35618	35763	-	145
NW_015926401.1	32681	32741	-	60
NW_015926401.1	35614	35787	-	173
NW_015926652.1	19311	19451	-	140
NW_015926675.1	41765	41818	-	53
NW_015926811.1	73385	73497	+	112
NW_015926811.1	73371	73517	+	146

NW_015926811.1	73350	73529	+	179
NW_015926815.1	51351	51458	-	107
NW_015926839.1	223303	223356	-	53
NW_015926966.1	9258	9391	-	133
NW_015927007.1	41707	41753	-	46
NW_015927105.1	51148	51240	-	92
NW_015927105.1	50942	50974	-	27
NW_015927105.1	51106	51408	-	302
NW_015927141.1	31987	32288	-	180
NW_015927141.1	31993	32290	-	182
NW_015927141.1	32075	32274	-	166
NW_015927329.1	27054	27195	-	141
NW_015927329.1	27049	27182	-	133
NW_015927633.1	14409	14473	-	64
NW_015927718.1	7834	7905	-	71
NW_015927718.1	7844	7900	-	56
NW_015927837.1	65245	65328	+	83
NW_015928006.1	35749	35860	+	111
NW_015928100.1	57361	57521	+	160
NW_015928200.1	10504	10597	+	93
NW_015928200.1	11031	11173	+	142
NW_015928373.1	4637	5003	-	330
NW_015928392.1	33652	33722	+	70
NW_015928592.1	56102	56237	+	135
NW_015928664.1	2154	2215	+	61

NW_015928664.1	2167	2224	+	57
NW_015928664.1	2157	2221	+	64
NW_015928743.1	34350	34425	-	75
NW_015929565.1	11758	11946	-	188
NW_015929565.1	11722	11947	-	225
NW_015929865.1	15153	15389	-	236
NW_015929870.1	10738	10815	-	77
NW_015929896.1	132619	132850	+	231
NW_015930022.1	108329	108737	+	408
NW_015930022.1	99342	99371	-	29
NW_015930097.1	46180	46280	+	100
NW_015930184.1	25250	25397	-	147
NW_015930350.1	5291	5697	-	406
NW_015930350.1	5062	5465	-	268
NW_015930350.1	5049	5705	-	508
NW_015930723.1	29261	29337	+	76
NW_015930915.1	48302	48373	+	71
NW_015930988.1	8788	8909	-	121
NW_015931444.1	22671	22874	-	203
NW_015931550.1	16436	16496	+	60
NW_015931581.1	36861	37020	+	159
NW_015931806.1	16338	16440	-	102
NW_015931819.1	7174	7402	+	228
NW_015931819.1	7145	7400	+	255
NW_015931819.1	7144	7437	+	293

NW_015931842.1	5206	5455	-	249
NW_015931897.1	26104	26248	+	144
NW_015932141.1	34261	34588	+	327
NW_015932141.1	34266	34783	+	517
NW_015932222.1	13799	13931	-	132
NW_015932673.1	37120	37171	-	51
NW_015932901.1	5377	5467	+	90
NW_015933085.1	3378	3459	-	81
NW_015933265.1	9628	9729	+	101
NW_015933278.1	43692	43766	-	39
NW_015933313.1	3218	3314	+	96
NW_015933313.1	3227	3311	+	84
NW_015934100.1	104225	104342	-	117
NW_015934159.1	83740	83797	-	57
NW_015934159.1	83739	83803	-	64
NW_015934187.1	152970	153015	+	45
NW_015934222.1	1114	1182	+	68
NW_015934352.1	4402	4500	+	98
NW_015934383.1	49064	49087	-	23
NW_015934436.1	38810	38966	+	156
NW_015934455.1	9821	9862	+	41
NW_015934627.1	27832	27960	-	128
NW_015934627.1	3886	3978	+	92
NW_015934705.1	97116	97164	+	48
NW_015934705.1	97090	97162	+	72

NW_015934705.1	97088	97155	+	67
NW_015934918.1	26408	26501	-	93
NW_015935386.1	122941	123002	+	61
NW_015935386.1	122899	123061	+	162
NW_015935599.1	28060	28167	-	78
NW_015935759.1	18520	18596	-	76
NW_015935759.1	18476	18639	-	163
NW_015935759.1	18436	18636	-	200
NW_015935841.1	69351	69520	-	89
NW_015935988.1	21315	21654	+	339
NW_015936305.1	4027	4092	+	65
NW_015936305.1	4033	4088	+	55
NW_015936352.1	24399	24426	-	27
NW_015936381.1	29318	29393	+	75
NW_015936596.1	60426	60558	-	132
NW_015937086.1	14677	14738	-	61
NW_015937119.1	25211	25361	-	150
NW_015937234.1	317674	317776	+	78
NW_015937241.1	41139	41241	-	102
NW_015937468.1	48603	48846	-	243
NW_015937468.1	48605	48885	-	280
NW_015937468.1	48614	48879	-	265
NW_015937765.1	25904	25930	-	26
NW_015938167.1	29	124	-	95
NW_015938284.1	27843	27884	+	41

NW_015938555.1	28842	28915	-	73
NW_015938902.1	15449	15531	+	82
NW_015938902.1	15050	15266	+	216
NW_015938902.1	15408	15546	+	138
NW_015938964.1	11349	11464	-	115
NW_015939165.1	7130	7227	-	97
NW_015939165.1	7128	7225	-	97
NW_015939391.1	62865	63029	-	164
NW_015939773.1	33484	33696	-	212
NW_015940228.1	11275	11394	-	79
NW_015940333.1	76332	76445	+	113
NW_015940333.1	76226	76452	+	226
NW_015940347.1	37600	37680	-	80
NW_015940574.1	30186	30402	+	216
NW_015940596.1	28762	28912	-	150
NW_015940596.1	28784	28911	-	127
NW_015940596.1	28768	28869	-	101
NW_015940635.1	7623	7758	-	135
NW_015940635.1	7609	7742	-	133
NW_015940635.1	7608	7777	-	169
NW_015940740.1	7916	8049	-	133
NW_015941003.1	51635	51776	-	141
NW_015941031.1	9361	9478	-	117
NW_015941516.1	32057	32168	+	111
NW_015941547.1	8371	8516	-	145

NW_015941552.1	44016	44050	-	34
NW_015941661.1	78888	79009	-	121
NW_015941661.1	78865	79002	-	137
NW_015941661.1	93247	93541	+	164
NW_015941739.1	12877	12978	+	19
NW_015941847.1	7477	7538	+	61
NW_015941917.1	86931	86996	-	65
NW_015942054.1	20201	20256	-	55
NW_015942228.1	94374	94537	-	163
NW_015942326.1	13770	13927	+	157
NW_015942336.1	38773	38881	-	108
NW_015942583.1	5686	5784	+	98
NW_015943099.1	34684	34781	+	97
NW_015943233.1	17129	17169	-	40
NW_015943245.1	35145	35301	+	156
NW_015943297.1	14599	14687	-	88
NW_015943297.1	14523	14649	-	126
NW_015943297.1	14516	14690	-	174
NW_015943607.1	36752	36765	-	13
NW_015943817.1	28101	28241	+	78
NW_015943817.1	28091	28257	+	88
NW_015943827.1	87323	87530	-	207
NW_015943860.1	338	389	+	51
NW_015944005.1	18240	18377	-	137
NW_015944040.1	55123	55411	+	288

NW_015944148.1	98509	98664	-	155
NW_015944424.1	2547	2580	-	33
NW_015944489.1	8720	8763	+	43
NW_015944541.1	1144	1288	-	17
NW_015944541.1	1187	1282	-	11
NW_015944541.1	1155	1286	-	15
NW_015944732.1	67970	68048	+	78
NW_015945258.1	26484	26560	-	76
NW_015945258.1	26349	26606	-	257
NW_015945275.1	18737	18773	-	36
NW_015945275.1	19116	19280	-	164
NW_015945275.1	19104	19281	-	177
NW_015945275.1	18714	18793	-	79
NW_015945478.1	6192	6370	-	178
NW_015945761.1	20594	20724	+	130
NW_015945791.1	36090	36490	-	400
NW_015945791.1	36097	36468	-	371
NW_015945791.1	36079	36526	-	447
NW_015945827.1	14759	14863	-	104
NW_015945875.1	40311	40441	-	130
NW_015945972.1	24477	24591	+	114
NW_015946040.1	69069	69111	-	42
NW_015946068.1	55759	55912	-	78
NW_015946068.1	55676	55908	-	158
NW_015946639.1	30587	30620	-	33

NW_015946812.1	73237	73378	-	141
NW_015946913.1	24170	24391	-	221
NW_015946913.1	24173	24648	-	475
NW_015946945.1	11882	11947	-	65
NW_015946945.1	11822	11975	-	153
NW_015946945.1	11819	11972	-	153
NW_015946956.1	108921	109074	+	153
NW_015947026.1	39588	39753	-	165
NW_015947333.1	113540	113614	-	74
NW_015947391.1	23487	23687	+	200
NW_015948154.1	63324	63426	-	102
NW_015948186.1	15731	16023	-	292
NW_015948276.1	34512	34587	+	75
NW_015948286.1	18048	18133	-	85
NW_015948766.1	55863	56024	+	161
NW_015948940.1	33195	33357	-	162
NW_015948940.1	33197	33344	-	147
NW_015948940.1	33170	33385	-	215
NW_015948976.1	172322	172363	+	41
NW_015949014.1	32983	33056	-	73
NW_015949014.1	32982	33074	-	92
NW_015949014.1	32989	33073	-	84
NW_015949612.1	147058	147211	+	153
NW_015949806.1	31799	31991	-	192
NW_015949806.1	4141	4446	-	244

NW_015949806.1	31806	31997	-	191
NW_015949825.1	79883	79947	+	63
NW_015949825.1	79851	79944	+	60
NW_015950147.1	8906	8994	-	88
NW_015950147.1	8871	9000	-	129
NW_015950258.1	99534	99559	+	25
NW_015950258.1	99515	99551	+	36
NW_015950258.1	83962	84050	+	88
NW_015950258.1	84579	84655	+	76
NW_015950258.1	99495	99574	+	79
NW_015950377.1	27302	27381	+	79
NW_015950562.1	6382	6506	+	124
NW_015950798.1	21726	21845	+	119
NW_015951571.1	4389	4448	-	59
NW_015951862.1	23867	23966	+	84
NW_015951974.1	17288	17499	-	211
NW_015951974.1	17291	17507	-	216
NW_015952009.1	16295	16427	-	129
NW_015952105.1	12853	12970	+	117
NW_015952120.1	32556	32743	+	187
NW_015952120.1	32555	32744	+	189
NW_015952120.1	32551	32722	+	171
NW_015952521.1	136259	136399	+	140
NW_015952983.1	12568	12710	-	142
NW_015953170.1	5855	5950	+	95

NW_015953211.1	71427	71449	+	22
NW_015953320.1	12682	13017	+	335
NW_015953320.1	12811	13054	+	243
NW_015953320.1	12677	13048	+	371
NW_015953492.1	12104	12368	+	264
NW_015953507.1	10200	10290	+	90
NW_015953507.1	10177	10326	+	149
NW_015953580.1	39343	39621	-	278
NW_015953580.1	39408	39603	-	195
NW_015953580.1	39333	39532	-	199
NW_015953599.1	15451	15589	-	138
NW_015953731.1	23514	23597	-	42
NW_015953774.1	68326	68389	+	63
NW_015953774.1	68328	68409	+	81
NW_015954099.1	45984	46097	+	113
NW_015954102.1	34075	34213	-	138
NW_015954636.1	17276	17425	-	149
NW_015954636.1	8729	8843	+	114
NW_015954636.1	17306	17415	+	109
NW_015954636.1	8656	8895	+	239
NW_015954636.1	17264	17412	+	148
NW_015955219.1	67530	67601	-	71

16days post inoculation (Hypo-DMRs)

Chr	DMR_Start	DMR_End	RegionStart	OverlappedSite
NW_015787321.1	11238	11298	-	60
NW_015787340.1	4235	4291	+	56
NW_015787340.1	4198	4299	+	101
NW_015787375.1	17919	18061	-	142
NW_015787444.1	27469	27713	-	244
NW_015787463.1	21997	22103	-	106
NW_015787655.1	25044	25132	+	88
NW_015787679.1	23068	23131	-	63
NW_015787703.1	14839	15053	+	214
NW_015787753.1	2696	2768	+	72
NW_015787775.1	33801	33918	+	117
NW_015787854.1	27742	27830	-	88
NW_015788138.1	67168	67212	+	44
NW_015788158.1	8879	9164	-	285
NW_015788210.1	10965	11088	-	73
NW_015788314.1	21357	21510	-	153
NW_015788314.1	21880	22015	-	135
NW_015788317.1	27663	27697	-	34
NW_015788476.1	34624	34813	-	98
NW_015788513.1	3823	3987	+	164
NW_015788528.1	12621	12655	-	34
NW_015788570.1	63332	63474	-	142
NW_015788570.1	59931	60157	-	226
NW_015788608.1	29233	29373	-	140

NW_015788700.1	8016	8154	-	45
NW_015788706.1	264186	264316	-	130
NW_015788719.1	36282	36340	-	58
NW_015788757.1	16421	16526	+	105
NW_015788759.1	24700	24779	+	79
NW_015788908.1	22831	22893	+	62
NW_015788943.1	13553	13609	-	56
NW_015789118.1	3968	4061	+	93
NW_015789259.1	18760	18884	+	124
NW_015789314.1	13388	13480	-	92
NW_015789404.1	3180	3270	+	90
NW_015789405.1	4782	4863	-	81
NW_015789405.1	4656	4870	-	214
NW_015789585.1	7496	7602	+	106
NW_015789620.1	81358	81447	-	89
NW_015789624.1	62002	62152	-	150
NW_015789752.1	27853	27875	-	22
NW_015789944.1	6670	6961	-	136
NW_015790084.1	10918	11097	+	179
NW_015790111.1	17036	17224	-	188
NW_015790118.1	88439	88574	+	135
NW_015790311.1	40835	41011	+	100
NW_015790311.1	40604	41029	+	275
NW_015790311.1	50641	50789	-	148
NW_015790413.1	7538	7707	+	169

NW_015790534.1	8114	8382	+	268
NW_015790612.1	117383	117478	+	95
NW_015790638.1	3265	3383	+	118
NW_015790638.1	12900	12930	-	30
NW_015790646.1	22571	22703	+	132
NW_015790868.1	35848	36051	+	203
NW_015790989.1	11762	11864	-	102
NW_015791028.1	26822	27056	+	132
NW_015791242.1	72536	72688	+	152
NW_015791267.1	88673	88726	+	53
NW_015791267.1	88662	88739	+	77
NW_015791293.1	53709	53783	-	74
NW_015791433.1	29367	29491	-	124
NW_015791444.1	14965	15038	+	73
NW_015791444.1	14919	15043	+	124
NW_015791560.1	4353	4452	+	95
NW_015791560.1	4344	4459	+	102
NW_015791567.1	22418	22542	-	124
NW_015791861.1	26374	26560	+	73
NW_015791955.1	18319	18403	+	84
NW_015791955.1	18288	18404	+	116
NW_015791958.1	22588	22744	-	156
NW_015792039.1	9109	9205	-	96
NW_015792055.1	122838	122956	-	118
NW_015792158.1	22423	22546	-	123

NW_015792185.1	12650	12810	-	160
NW_015792204.1	7414	7563	+	149
NW_015792333.1	82337	82582	-	245
NW_015792335.1	47204	47335	-	131
NW_015792376.1	37058	37403	-	345
NW_015792376.1	6288	6383	+	95
NW_015792483.1	37767	38099	+	332
NW_015792500.1	14570	14646	+	76
NW_015792520.1	11234	11282	+	48
NW_015792589.1	8562	8879	+	317
NW_015792642.1	36252	36386	+	134
NW_015792710.1	51439	51554	+	115
NW_015792740.1	31910	31951	-	41
NW_015792849.1	68447	68573	+	126
NW_015792948.1	146450	146507	+	57
NW_015793021.1	25609	25694	+	85
NW_015793035.1	101355	101383	-	28
NW_015793066.1	36011	36119	-	108
NW_015793152.1	25362	25437	+	75
NW_015793231.1	66055	66093	-	38
NW_015793234.1	7948	8048	-	100
NW_015793238.1	22079	22228	-	149
NW_015793434.1	11974	12014	+	40
NW_015793434.1	11966	12009	+	43
NW_015793469.1	70952	71007	-	29

NW_015793573.1	73483	73651	+	168
NW_015793598.1	5346	5462	-	116
NW_015793615.1	17569	17700	-	131
NW_015793650.1	39149	39200	-	51
NW_015793978.1	13186	13231	-	45
NW_015794002.1	92188	92330	-	142
NW_015794132.1	199383	199451	-	68
NW_015794132.1	171072	171126	-	54
NW_015794132.1	171041	171114	-	73
NW_015794158.1	27261	27288	-	27
NW_015794193.1	43111	43213	+	102
NW_015794196.1	5734	5810	+	76
NW_015794332.1	6945	7069	+	124
NW_015794475.1	149402	149474	-	47
NW_015794503.1	61912	61962	+	50
NW_015794511.1	38160	38247	+	87
NW_015794620.1	8762	8822	-	60
NW_015794640.1	35718	35910	+	192
NW_015794811.1	43162	43276	-	114
NW_015794825.1	58099	58256	+	157
NW_015794850.1	8650	8766	-	97
NW_015795024.1	22729	22754	+	25
NW_015795033.1	13249	13604	-	243
NW_015795042.1	12026	12207	+	181
NW_015795193.1	3213	3328	+	115

NW_015795225.1	7134	7164	+	30
NW_015795290.1	50549	50733	+	184
NW_015795426.1	24773	25063	-	290
NW_015795512.1	97351	97452	+	101
NW_015795619.1	106626	106698	-	72
NW_015795628.1	19089	19265	-	175
NW_015795654.1	7021	7171	+	88
NW_015795689.1	6039	6156	+	67
NW_015795724.1	17595	17723	-	128
NW_015795724.1	17588	17716	-	128
NW_015795761.1	135477	135567	+	90
NW_015795779.1	1145	1257	-	112
NW_015795818.1	159555	159673	+	67
NW_015795840.1	97173	97198	+	25
NW_015795913.1	6410	6465	+	55
NW_015795960.1	120073	120531	-	458
NW_015796009.1	5456	5513	-	57
NW_015796036.1	7361	7518	-	37
NW_015796104.1	46042	46309	+	99
NW_015796104.1	46037	46326	+	116
NW_015796127.1	58290	58723	-	433
NW_015796173.1	5158	5262	+	104
NW_015796287.1	17753	17785	-	32
NW_015796305.1	16909	17080	-	171
NW_015796366.1	6157	6275	+	118

NW_015796367.1	14543	14824	+	281
NW_015796504.1	65064	65284	-	220
NW_015796504.1	33057	33205	+	94
NW_015796526.1	59054	59218	+	164
NW_015796699.1	19417	19500	+	83
NW_015796876.1	21619	21821	+	152
NW_015796936.1	2217	2282	+	65
NW_015796940.1	16596	16647	-	51
NW_015796958.1	203327	203457	+	97
NW_015797084.1	2808	2831	+	23
NW_015797183.1	106299	106526	-	227
NW_015797221.1	99439	99725	+	286
NW_015797403.1	4563	4654	-	91
NW_015797403.1	4586	4678	-	92
NW_015797438.1	1775	1851	+	76
NW_015797438.1	1735	1853	+	118
NW_015797568.1	51198	51278	-	80
NW_015797899.1	57595	57660	+	65
NW_015797903.1	7918	8061	+	143
NW_015798051.1	4758	4926	+	168
NW_015798165.1	12709	12913	+	204
NW_015798305.1	18520	18643	-	123
NW_015798380.1	35260	35390	+	130
NW_015798528.1	18426	18507	-	81
NW_015798807.1	13443	13527	-	84

NW_015798807.1	13434	13535	-	101
NW_015798878.1	6419	6631	+	212
NW_015798909.1	8652	8785	+	133
NW_015798921.1	15456	15516	+	60
NW_015798989.1	6459	6578	+	119
NW_015798995.1	14588	14725	+	137
NW_015799259.1	24193	24275	-	82
NW_015799259.1	98656	98812	+	156
NW_015799321.1	12427	12896	-	469
NW_015799362.1	85106	85279	+	173
NW_015799376.1	13535	13542	+	7
NW_015799669.1	22014	22169	-	155
NW_015799707.1	13274	13400	-	126
NW_015799868.1	12795	12929	-	134
NW_015800001.1	32186	32401	+	215
NW_015800029.1	24523	24563	+	40
NW_015800091.1	33692	33870	+	178
NW_015800126.1	6009	6194	+	94
NW_015800142.1	59556	59598	+	42
NW_015800150.1	115900	115961	-	61
NW_015800263.1	10680	10865	-	115
NW_015800329.1	18048	18151	+	103
NW_015800391.1	2718	2952	+	234
NW_015800391.1	2670	2949	+	240
NW_015800462.1	13870	14038	-	168

NW_015800467.1	3241	3359	+	118
NW_015800604.1	32218	32460	-	242
NW_015800633.1	40987	41027	+	40
NW_015800693.1	7810	7867	+	57
NW_015800713.1	22006	22082	+	76
NW_015800760.1	19721	19777	+	56
NW_015800782.1	50417	50619	+	202
NW_015801051.1	6607	6766	-	159
NW_015801051.1	6604	6818	-	214
NW_015801185.1	2481	2612	-	77
NW_015801233.1	51977	52073	+	96
NW_015801241.1	54992	55123	+	131
NW_015801269.1	5018	5125	+	107
NW_015801275.1	37718	37834	+	116
NW_015801281.1	80858	80968	-	110
NW_015801296.1	24532	24688	+	156
NW_015801297.1	46382	46554	+	172
NW_015801567.1	22861	22937	+	76
NW_015801678.1	11099	11171	+	72
NW_015801726.1	75947	76107	+	160
NW_015801759.1	15259	15482	-	223
NW_015801843.1	15724	15789	-	65
NW_015801978.1	51278	51463	-	185
NW_015802068.1	27956	28003	+	47
NW_015802103.1	49689	49826	-	137

NW_015802354.1	17259	17324	+	65
NW_015802410.1	23448	23510	+	62
NW_015802451.1	28669	28815	+	146
NW_015802646.1	105590	105681	+	91
NW_015802666.1	87566	87792	-	226
NW_015802682.1	14178	14401	+	223
NW_015802882.1	19046	19155	+	109
NW_015802882.1	157773	157900	+	127
NW_015802882.1	144235	144275	+	40
NW_015802919.1	3053	3254	+	201
NW_015802919.1	3077	3265	+	188
NW_015802928.1	32859	32942	+	83
NW_015802928.1	32854	33080	+	226
NW_015802956.1	86106	86233	-	127
NW_015803008.1	8498	8719	-	25
NW_015803015.1	3414	3559	-	145
NW_015803111.1	28128	28432	+	304
NW_015803120.1	32166	32220	+	54
NW_015803187.1	20147	20373	+	226
NW_015803333.1	13595	13739	-	135
NW_015803439.1	19835	19925	-	90
NW_015803472.1	28674	28735	+	61
NW_015803648.1	15016	15067	+	51
NW_015803671.1	1088	1315	-	227
NW_015803680.1	56834	57266	-	432

NW_015803680.1	56914	57358	-	444
NW_015803686.1	14577	14644	-	67
NW_015803742.1	16984	17080	-	96
NW_015803772.1	2426	2538	-	112
NW_015803794.1	65103	65341	-	238
NW_015803895.1	4038	4157	-	83
NW_015804033.1	14309	14523	+	214
NW_015804073.1	57144	57290	+	146
NW_015804073.1	63010	63110	+	79
NW_015804131.1	151174	151307	-	133
NW_015804318.1	9337	9393	-	56
NW_015804409.1	59427	59567	+	140
NW_015804499.1	55595	55675	+	80
NW_015804525.1	12766	13125	-	359
NW_015804590.1	32412	32595	-	144
NW_015804590.1	32395	32593	-	161
NW_015804847.1	1859	1883	-	24
NW_015804904.1	40133	40401	+	268
NW_015804948.1	5931	6049	-	118
NW_015805006.1	196851	196960	+	109
NW_015805110.1	21296	21365	-	69
NW_015805116.1	25136	25328	+	192
NW_015805125.1	5593	5733	+	140
NW_015805344.1	17778	17951	-	173
NW_015805413.1	18612	18799	+	187

NW_015805636.1	27929	28086	-	83
NW_015805681.1	385812	385888	+	76
NW_015805860.1	32776	32884	-	108
NW_015805980.1	32326	32456	+	130
NW_015806020.1	5482	5572	+	90
NW_015806140.1	4827	4923	-	96
NW_015806190.1	40637	40844	-	207
NW_015806190.1	40536	40823	-	287
NW_015806426.1	33033	33166	-	133
NW_015806450.1	21110	21217	-	107
NW_015806476.1	30900	30952	-	52
NW_015806485.1	21527	21595	-	68
NW_015806518.1	131533	131610	-	77
NW_015806601.1	16166	16535	+	241
NW_015806601.1	110297	110375	+	78
NW_015806621.1	175	329	-	154
NW_015806709.1	26302	26424	+	122
NW_015806749.1	5041	5247	-	206
NW_015806933.1	58498	58521	-	23
NW_015807019.1	23798	23895	+	97
NW_015807570.1	2297	2364	+	67
NW_015807602.1	17526	17644	-	118
NW_015807629.1	164402	164453	+	51
NW_015807646.1	40878	41024	-	146
NW_015807712.1	9594	9737	+	143

NW_015807757.1	55568	55670	-	102
NW_015807931.1	27049	27137	-	88
NW_015807950.1	125317	125392	+	75
NW_015808028.1	43503	43531	+	28
NW_015808065.1	8675	9052	-	377
NW_015808069.1	86621	86900	-	279
NW_015808103.1	20593	20705	-	112
NW_015808104.1	51368	51471	-	70
NW_015808218.1	247	306	+	59
NW_015808451.1	11936	12087	-	151
NW_015808465.1	13047	13340	-	190
NW_015808541.1	304926	304981	-	55
NW_015808587.1	20832	20976	+	144
NW_015808778.1	99179	99293	-	114
NW_015808796.1	17825	17920	-	95
NW_015808904.1	998	1196	-	103
NW_015808973.1	12083	12132	-	49
NW_015808973.1	11901	12148	-	247
NW_015808999.1	66856	66971	-	115
NW_015809059.1	23145	23301	-	156
NW_015809059.1	23135	23289	-	154
NW_015809094.1	16913	17007	-	68
NW_015809247.1	25389	25575	-	186
NW_015809346.1	93140	93254	+	108
NW_015809359.1	27774	27825	+	51

NW_015809440.1	6470	6528	-	58
NW_015809596.1	2847	3141	-	294
NW_015809613.1	11692	11784	-	92
NW_015809613.1	11685	11785	-	100
NW_015809759.1	49658	49799	-	141
NW_015809922.1	252688	252775	-	87
NW_015809922.1	258977	259134	-	157
NW_015809989.1	27591	27701	-	110
NW_015809996.1	42997	43047	+	50
NW_015810105.1	3843	3881	+	38
NW_015810109.1	3839	3956	-	117
NW_015810183.1	9125	9246	-	121
NW_015810194.1	43106	43214	+	104
NW_015810241.1	1454	1499	+	45
NW_015810395.1	5798	5983	-	108
NW_015810449.1	2249	2394	-	145
NW_015810452.1	2773	2873	+	100
NW_015810781.1	13602	14049	+	447
NW_015810795.1	1974	2085	+	111
NW_015810839.1	52008	52035	+	27
NW_015810839.1	51999	52043	+	44
NW_015810861.1	99394	99522	+	128
NW_015810861.1	254896	255014	-	118
NW_015810917.1	43009	43056	-	47
NW_015810983.1	19232	19358	-	126

NW_015811024.1	15501	15607	-	106
NW_015811033.1	275747	275801	+	54
NW_015811046.1	12524	12648	-	124
NW_015811093.1	30725	30810	-	85
NW_015811149.1	13304	13426	+	84
NW_015811149.1	13296	13386	+	90
NW_015811149.1	14014	14216	+	202
NW_015811158.1	136449	136557	+	108
NW_015811231.1	141664	141807	-	143
NW_015811280.1	28278	28425	-	147
NW_015811418.1	6185	6269	+	84
NW_015811527.1	24562	24758	+	196
NW_015811571.1	46808	46925	-	117
NW_015811571.1	41227	41316	-	89
NW_015811571.1	46858	46937	-	79
NW_015811591.1	41977	42090	+	113
NW_015811605.1	9837	9938	-	74
NW_015811843.1	15467	15570	-	103
NW_015811867.1	70204	70309	+	105
NW_015811883.1	109744	109892	+	148
NW_015812026.1	13953	14051	-	98
NW_015812182.1	32957	33110	+	153
NW_015812256.1	15446	15737	-	291
NW_015812279.1	7641	7675	+	34
NW_015812377.1	34966	35075	-	109

NW_015812421.1	20655	20710	-	55
NW_015812421.1	35585	35719	-	134
NW_015812421.1	20654	20711	+	57
NW_015812492.1	56628	57052	-	424
NW_015812500.1	21168	21277	-	109
NW_015812517.1	35229	35336	-	107
NW_015812517.1	35234	35345	-	111
NW_015812562.1	14784	14887	-	103
NW_015812570.1	1927	2131	+	204
NW_015812707.1	40602	40659	-	57
NW_015812716.1	13897	13992	+	67
NW_015812797.1	4273	4393	-	120
NW_015812800.1	37222	37399	-	177
NW_015812873.1	5298	5358	+	60
NW_015812947.1	14730	14796	-	66
NW_015813061.1	96943	97027	-	84
NW_015813085.1	20440	20526	-	86
NW_015813218.1	35306	35494	-	188
NW_015813263.1	12419	12500	-	81
NW_015813270.1	58746	58828	-	82
NW_015813270.1	11203	11410	-	207
NW_015813270.1	59041	59125	-	84
NW_015813464.1	5775	5909	-	134
NW_015813464.1	5786	5881	-	95
NW_015813595.1	6986	7190	+	204

NW_015813683.1	4835	4916	-	81
NW_015813924.1	1591	1777	+	104
NW_015813936.1	25009	25086	-	77
NW_015813936.1	25018	25216	-	198
NW_015813946.1	294	328	+	34
NW_015814015.1	18975	19086	-	111
NW_015814130.1	53695	53759	-	64
NW_015814212.1	30932	31077	-	145
NW_015814225.1	5812	5969	-	102
NW_015814451.1	31127	31317	-	190
NW_015814572.1	18049	18167	-	78
NW_015814572.1	18076	18189	-	28
NW_015814595.1	23473	23515	+	42
NW_015814765.1	12485	12541	-	56
NW_015814766.1	8275	8409	-	134
NW_015814814.1	68742	68882	+	140
NW_015814937.1	53944	53952	-	8
NW_015814964.1	103142	103245	-	103
NW_015814964.1	207450	207502	-	52
NW_015815030.1	72530	72723	-	193
NW_015815099.1	29582	29700	+	118
NW_015815149.1	6889	6995	-	106
NW_015815186.1	55401	55645	-	244
NW_015815189.1	15882	15923	+	41
NW_015815430.1	122798	123486	-	688

NW_015815513.1	33749	33826	-	77
NW_015815682.1	15734	15776	+	42
NW_015815718.1	91734	91759	-	25
NW_015815745.1	7551	7701	-	150
NW_015815772.1	8247	8377	-	130
NW_015815772.1	8306	8361	-	55
NW_015815911.1	18921	19012	+	91
NW_015815911.1	18840	19036	+	196
NW_015815959.1	93003	93103	-	100
NW_015815959.1	92966	93111	-	145
NW_015816232.1	119295	119375	-	80
NW_015816359.1	186026	186203	-	177
NW_015816450.1	17465	17554	+	55
NW_015816626.1	3557	3640	-	83
NW_015816773.1	19117	19265	+	148
NW_015816773.1	19169	19347	+	178
NW_015816904.1	89192	89326	-	134
NW_015816928.1	16818	16944	+	126
NW_015816976.1	11892	12054	+	162
NW_015817148.1	17157	17256	+	99
NW_015817164.1	26626	26680	-	54
NW_015817186.1	36108	36301	-	193
NW_015817388.1	4244	4416	-	172
NW_015817641.1	36586	36656	+	70
NW_015817657.1	7893	8607	-	714

NW_015817715.1	18469	18499	-	30
NW_015818032.1	38632	38895	-	263
NW_015818104.1	32190	32359	-	169
NW_015818104.1	7336	7379	+	43
NW_015818214.1	45326	45442	-	116
NW_015818386.1	139657	139749	-	92
NW_015818386.1	139675	139807	-	132
NW_015818422.1	3438	3626	+	188
NW_015818474.1	1574	1694	-	120
NW_015818529.1	5250	5378	-	47
NW_015818566.1	15861	16226	+	365
NW_015818635.1	18278	18453	-	175
NW_015818713.1	10504	10528	+	24
NW_015818755.1	17356	17406	-	50
NW_015818789.1	16051	16260	-	209
NW_015818917.1	47685	47801	-	116
NW_015819137.1	21035	21161	+	126
NW_015819179.1	87564	87719	-	155
NW_015819179.1	196857	197018	+	93
NW_015819268.1	106115	106221	+	104
NW_015819357.1	2103	2220	+	117
NW_015819643.1	25090	25137	-	47
NW_015819643.1	25096	25135	-	39
NW_015819698.1	16309	16459	+	123
NW_015819897.1	56838	56897	-	59

NW_015820059.1	27397	27523	+	126
NW_015820118.1	42010	42090	-	80
NW_015820118.1	20948	21009	+	61
NW_015820176.1	27904	27997	-	93
NW_015820259.1	74689	74961	+	272
NW_015820416.1	15813	15841	-	28
NW_015820454.1	21308	21463	-	155
NW_015820494.1	4677	5202	+	525
NW_015820820.1	44223	44366	+	143
NW_015820840.1	13085	13143	-	58
NW_015820923.1	18030	18173	-	143
NW_015820924.1	29020	29141	-	121
NW_015821066.1	59264	59441	+	177
NW_015821092.1	27	322	+	194
NW_015821092.1	18	327	+	203
NW_015821122.1	11364	11505	-	128
NW_015821240.1	17257	17338	-	81
NW_015821299.1	54142	54234	+	92
NW_015821418.1	9414	9541	+	127
NW_015821688.1	64416	64502	-	86
NW_015821854.1	220689	220762	-	73
NW_015821934.1	4415	4494	-	1
NW_015821994.1	80395	80445	-	50
NW_015822028.1	23163	23235	-	72
NW_015822052.1	46156	46250	-	94

NW_015822203.1	38579	38646	+	67
NW_015822307.1	12432	12597	+	165
NW_015822502.1	18120	18234	+	114
NW_015822561.1	135323	135489	+	166
NW_015822674.1	52779	52965	-	186
NW_015822755.1	4630	4793	-	163
NW_015822824.1	213867	214008	-	141
NW_015822915.1	50798	50836	+	38
NW_015822915.1	50487	50828	+	341
NW_015823041.1	52869	52931	-	62
NW_015823203.1	12921	13025	+	104
NW_015823374.1	17012	17054	+	42
NW_015823637.1	46762	46825	-	63
NW_015823752.1	6430	6512	-	82
NW_015823772.1	8466	8596	+	130
NW_015823838.1	3395	3549	-	154
NW_015823838.1	12851	13020	-	169
NW_015823840.1	75310	75451	-	141
NW_015823886.1	4515	4569	-	54
NW_015824029.1	29067	29129	-	36
NW_015824250.1	6958	7055	-	97
NW_015824555.1	13262	13379	-	117
NW_015824723.1	77236	77268	+	32
NW_015824779.1	16221	16327	-	106
NW_015825212.1	32481	32615	-	134

NW_015825357.1	90124	90290	-	166
NW_015825368.1	115451	115624	+	173
NW_015825368.1	222313	222424	+	111
NW_015825418.1	76428	76521	-	93
NW_015825432.1	1710	1808	+	98
NW_015825541.1	10500	10558	+	50
NW_015825567.1	4239	4362	+	123
NW_015825634.1	6696	6753	+	49
NW_015825712.1	31681	31933	-	252
NW_015825823.1	17828	17959	+	131
NW_015825953.1	23863	24073	-	210
NW_015826083.1	3963	4105	-	142
NW_015826102.1	18926	19005	+	79
NW_015826233.1	17834	18025	+	191
NW_015826233.1	17821	18007	+	186
NW_015826303.1	48415	48557	+	142
NW_015826434.1	47313	47449	-	136
NW_015826799.1	69621	69758	+	137
NW_015826879.1	6770	6815	+	45
NW_015826961.1	32644	32753	-	109
NW_015827070.1	124702	124771	+	69
NW_015827436.1	12114	12172	+	58
NW_015827687.1	186	283	-	97
NW_015827801.1	307007	307113	+	106
NW_015827801.1	116321	116411	-	70

NW_015827801.1	175842	176029	-	115
NW_015827850.1	9040	9144	-	104
NW_015828272.1	28558	28602	-	44
NW_015828272.1	28333	28594	-	261
NW_015828298.1	20716	21153	-	437
NW_015828379.1	2031	2146	+	115
NW_015828413.1	9145	9332	+	187
NW_015828413.1	9128	9360	+	232
NW_015828505.1	33695	33777	-	82
NW_015828505.1	43492	43568	+	76
NW_015828882.1	36175	36338	-	117
NW_015828972.1	16364	16472	+	108
NW_015829102.1	17497	17558	+	61
NW_015829262.1	44090	44426	-	336
NW_015829266.1	4644	4817	-	171
NW_015829266.1	4631	4961	-	184
NW_015829300.1	8279	8321	+	42
NW_015829454.1	175510	175757	+	247
NW_015829611.1	22139	22177	-	38
NW_015829643.1	11946	12069	+	123
NW_015829661.1	83951	84162	+	211
NW_015829661.1	107410	107474	-	64
NW_015829671.1	27010	27077	-	67
NW_015829679.1	19228	19344	-	116
NW_015829783.1	15649	15929	-	280

NW_015829824.1	7679	7830	+	151
NW_015829824.1	7558	7850	+	292
NW_015829975.1	5067	5190	-	123
NW_015829975.1	5303	5366	-	63
NW_015830246.1	8966	9081	+	115
NW_015830272.1	39726	39820	+	94
NW_015830339.1	46924	47057	+	133
NW_015830448.1	59406	59532	+	126
NW_015830473.1	121343	121513	+	170
NW_015830513.1	22348	22524	-	51
NW_015830873.1	24798	24945	+	124
NW_015830949.1	9491	9651	-	160
NW_015830966.1	3721	3835	+	114
NW_015831077.1	3782	3959	+	177
NW_015831152.1	35969	36129	+	160
NW_015831365.1	4719	4880	+	161
NW_015831538.1	33418	33515	-	97
NW_015831606.1	22240	22323	-	83
NW_015831630.1	16104	16231	-	127
NW_015831798.1	59010	59219	-	172
NW_015831967.1	5170	5211	+	41
NW_015832227.1	27589	27737	+	148
NW_015832275.1	49256	49399	+	143
NW_015832748.1	27283	27762	-	479
NW_015832748.1	31798	31827	-	29

NW_015832874.1	26610	26712	+	102
NW_015833075.1	17706	17870	+	55
NW_015833075.1	17751	17955	+	72
NW_015833169.1	4372	4580	-	208
NW_015833195.1	3507	3597	+	90
NW_015833229.1	7809	8009	+	200
NW_015833460.1	36870	37198	+	328
NW_015833563.1	12836	12943	-	107
NW_015833585.1	26513	26622	-	109
NW_015833620.1	1959	2119	+	160
NW_015833943.1	6068	6129	-	61
NW_015833943.1	6050	6128	-	78
NW_015833943.1	6056	6127	-	71
NW_015834229.1	15542	15625	-	83
NW_015834252.1	29394	29738	+	321
NW_015834373.1	3118	3325	-	139
NW_015834423.1	29953	30092	+	139
NW_015834423.1	29952	30088	+	136
NW_015834480.1	66162	66266	-	104
NW_015834625.1	5907	5996	+	89
NW_015834632.1	82277	82337	+	60
NW_015834901.1	222387	222650	-	263
NW_015835185.1	2566	2658	+	92
NW_015835205.1	82006	82293	-	287
NW_015835205.1	92886	92991	-	105

NW_015835299.1	11054	11280	-	226
NW_015835530.1	17554	17689	-	129
NW_015835620.1	187529	187646	-	117
NW_015835668.1	125867	125996	-	129
NW_015835668.1	125701	125945	-	161
NW_015835809.1	23961	24007	-	46
NW_015835922.1	51616	51765	-	149
NW_015836007.1	7413	7473	-	60
NW_015836007.1	7670	7980	-	310
NW_015836014.1	36039	36322	-	283
NW_015836154.1	57970	58025	-	55
NW_015836160.1	64369	64530	+	90
NW_015836300.1	6521	6576	-	55
NW_015836416.1	20938	20993	+	55
NW_015836416.1	20936	21028	+	92
NW_015836566.1	26328	26430	+	102
NW_015836620.1	8686	8762	+	76
NW_015836620.1	8656	8764	+	102
NW_015836672.1	4969	5066	-	97
NW_015836718.1	16296	16422	+	126
NW_015836967.1	1721	1789	-	68
NW_015836983.1	15985	16074	+	89
NW_015836983.1	16029	16095	+	66
NW_015836983.1	27433	27494	-	61
NW_015837020.1	1492	1706	+	214

NW_015837151.1	79687	80094	+	261
NW_015837205.1	37375	37474	+	99
NW_015837461.1	4503	4602	-	99
NW_015837861.1	14626	14686	-	60
NW_015837861.1	14610	14684	-	74
NW_015837861.1	8565	8611	+	46
NW_015837991.1	108742	108790	+	48
NW_015837991.1	108712	108830	+	118
NW_015838024.1	21753	21822	-	69
NW_015838084.1	3899	3954	-	55
NW_015838276.1	12611	12685	+	74
NW_015838488.1	7857	7989	-	132
NW_015838618.1	17505	17624	+	119
NW_015838630.1	22893	23016	-	123
NW_015838911.1	24275	24332	-	57
NW_015839040.1	19103	19176	+	55
NW_015839053.1	11024	11198	+	174
NW_015839194.1	38727	38803	+	76
NW_015839267.1	29122	29234	-	112
NW_015839452.1	21010	21145	+	135
NW_015839479.1	85480	85537	-	57
NW_015840082.1	23257	23390	-	133
NW_015840099.1	2449	2625	-	176
NW_015840179.1	13881	13930	+	49
NW_015840179.1	12643	12732	+	89

NW_015840200.1	17722	17829	+	107
NW_015840231.1	65020	65149	+	125
NW_015840344.1	24914	24992	-	76
NW_015840644.1	55150	55322	+	172
NW_015840769.1	15431	15641	-	210
NW_015840790.1	47783	47991	+	208
NW_015840790.1	49303	49445	+	142
NW_015840953.1	29675	29818	+	75
NW_015840967.1	33806	33914	+	108
NW_015840967.1	33828	33908	+	80
NW_015840967.1	33804	33927	+	123
NW_015841216.1	9148	9254	-	106
NW_015841230.1	102972	103023	+	51
NW_015841244.1	20540	20623	-	83
NW_015841254.1	31207	31236	+	29
NW_015841390.1	10179	10270	+	78
NW_015841746.1	89987	90137	-	150
NW_015841918.1	75571	75837	+	266
NW_015841952.1	11011	11037	+	26
NW_015842213.1	117143	117199	-	56
NW_015842213.1	102864	102954	-	58
NW_015842236.1	19140	19204	+	64
NW_015842291.1	16232	16303	-	71
NW_015842344.1	52992	53326	-	334
NW_015842452.1	64506	64637	+	131

NW_015842452.1	62676	62736	+	60
NW_015842647.1	1463	1567	+	104
NW_015842694.1	14533	14664	+	131
NW_015842735.1	6631	6712	+	81
NW_015842749.1	81202	81306	+	104
NW_015842764.1	193757	194027	+	270
NW_015843003.1	106549	106776	-	227
NW_015843003.1	106505	106743	-	238
NW_015843003.1	106495	106806	-	311
NW_015843045.1	17765	17846	+	81
NW_015843060.1	313	385	-	72
NW_015843060.1	278	401	-	123
NW_015843090.1	4171	4307	-	136
NW_015843249.1	93730	94010	+	280
NW_015843481.1	3405	3628	+	223
NW_015843749.1	70311	70451	-	140
NW_015843805.1	8425	8702	+	277
NW_015843929.1	6737	6758	+	21
NW_015843929.1	7337	7406	+	69
NW_015843980.1	27552	27638	-	86
NW_015843980.1	27550	27678	-	128
NW_015844092.1	130955	131146	+	191
NW_015844286.1	51070	51162	+	92
NW_015844639.1	21073	21168	+	95
NW_015844678.1	42651	42815	+	164

NW_015844808.1	3881	4020	-	139
NW_015844855.1	17123	17201	-	78
NW_015844978.1	8825	8905	-	80
NW_015845277.1	61375	61479	+	104
NW_015845477.1	2940	3326	+	386
NW_015845800.1	28872	28986	-	114
NW_015845989.1	44681	44725	+	44
NW_015846222.1	77610	77914	-	304
NW_015846301.1	204	435	+	231
NW_015846331.1	15748	16109	-	361
NW_015846425.1	253432	253578	-	146
NW_015846758.1	2010	2155	-	145
NW_015847361.1	40505	40538	-	33
NW_015847589.1	5058	5265	+	205
NW_015847597.1	89971	90006	-	35
NW_015847604.1	15093	15185	-	92
NW_015847612.1	13732	13881	+	96
NW_015847674.1	46124	46357	-	233
NW_015847674.1	46143	46323	-	180
NW_015847674.1	46130	46325	-	195
NW_015847674.1	76245	76319	+	74
NW_015847728.1	205404	205467	+	63
NW_015847989.1	27850	27957	+	84
NW_015848148.1	10686	10760	-	74
NW_015848535.1	48914	49058	-	144

NW_015848601.1	52666	52824	-	158
NW_015848816.1	57434	57559	+	125
NW_015848846.1	75313	75505	-	192
NW_015849015.1	7635	7678	+	43
NW_015849056.1	8352	8631	+	279
NW_015849252.1	55634	55928	+	294
NW_015849632.1	27810	28002	+	192
NW_015849664.1	59700	59807	+	45
NW_015849778.1	49652	49721	+	69
NW_015849778.1	49630	49745	+	115
NW_015849966.1	53694	53808	-	114
NW_015850069.1	23226	23296	-	70
NW_015850078.1	48271	48429	+	158
NW_015850182.1	27452	27511	-	59
NW_015850182.1	70743	70933	-	190
NW_015850224.1	7947	8009	+	62
NW_015850436.1	91404	91490	+	86
NW_015850497.1	35895	35989	-	94
NW_015850520.1	4433	4506	-	73
NW_015850570.1	4389	4662	+	273
NW_015850570.1	4330	4657	+	327
NW_015850791.1	5327	5479	+	152
NW_015850846.1	2932	2989	-	57
NW_015850890.1	2938	3104	-	166
NW_015850966.1	11827	11899	+	72

NW_015850992.1	12356	12418	-	62
NW_015851005.1	32760	32848	-	88
NW_015851005.1	32691	32838	-	147
NW_015851058.1	1315	1604	+	124
NW_015851058.1	1325	1590	+	114
NW_015851097.1	80399	80531	-	132
NW_015851138.1	36464	36623	+	159
NW_015851248.1	5537	5692	+	155
NW_015851313.1	47308	47500	+	192
NW_015851465.1	47777	47839	-	62
NW_015851465.1	47768	47840	-	72
NW_015851534.1	58807	58915	+	108
NW_015851558.1	72312	72394	+	82
NW_015851789.1	13323	13416	-	93
NW_015851810.1	22403	22445	-	42
NW_015851891.1	180856	180980	-	124
NW_015852066.1	8179	8345	-	166
NW_015852154.1	5535	5625	-	90
NW_015852163.1	73028	73225	+	89
NW_015852189.1	53808	53987	+	179
NW_015852473.1	16026	16107	+	81
NW_015852649.1	37456	37600	-	25
NW_015852803.1	152428	152480	-	52
NW_015852828.1	3651	3739	+	88
NW_015853176.1	27260	27303	-	43

NW_015853209.1	302217	302334	-	117
NW_015853270.1	17352	17574	-	155
NW_015853270.1	99360	99473	+	113
NW_015853347.1	93831	93928	-	97
NW_015853527.1	67423	67538	-	115
NW_015853696.1	7114	7203	+	74
NW_015853801.1	59513	59562	-	49
NW_015853978.1	28834	28966	+	132
NW_015854093.1	8060	8203	+	143
NW_015854099.1	36444	36465	+	21
NW_015854122.1	15447	15536	-	89
NW_015854260.1	5685	5780	+	95
NW_015854284.1	132671	133254	-	583
NW_015854478.1	17488	17626	-	138
NW_015854552.1	51045	51212	+	167
NW_015854573.1	38476	38581	-	68
NW_015854614.1	39221	39362	+	141
NW_015854664.1	5194	5327	-	133
NW_015854679.1	2490	2577	-	87
NW_015854682.1	38221	38411	-	131
NW_015854738.1	4265	4319	+	54
NW_015854870.1	3326	3466	-	140
NW_015854875.1	4242	4398	-	156
NW_015855024.1	26155	26296	+	141
NW_015855029.1	86089	86249	-	160

NW_015855108.1	206090	206189	+	99
NW_015855210.1	13705	13745	+	40
NW_015855210.1	44152	44189	+	37
NW_015855882.1	47683	47771	+	88
NW_015856130.1	35833	35905	-	72
NW_015856222.1	98100	98220	-	97
NW_015856591.1	28825	28908	+	83
NW_015856591.1	28805	28914	+	109
NW_015856591.1	51387	51540	+	153
NW_015856680.1	17142	17401	-	259
NW_015857129.1	8139	8240	+	101
NW_015857473.1	13252	13430	+	134
NW_015857518.1	73589	73632	-	43
NW_015857980.1	3119	3262	-	107
NW_015857996.1	12531	12675	+	144
NW_015858043.1	84492	84632	+	140
NW_015858073.1	3966	4139	+	173
NW_015858089.1	20229	20283	-	54
NW_015858629.1	70609	70842	-	233
NW_015858865.1	8857	9105	+	248
NW_015858865.1	13829	13892	+	63
NW_015858865.1	15823	15894	+	71
NW_015858944.1	46133	46215	+	57
NW_015858944.1	49587	49721	-	134
NW_015859072.1	86151	86314	+	163

NW_015859072.1	86120	86321	+	201
NW_015859199.1	42012	42088	-	76
NW_015859277.1	2854	2990	+	136
NW_015859277.1	3744	3851	+	107
NW_015859285.1	12059	12084	+	25
NW_015859544.1	17151	17738	-	587
NW_015859602.1	38640	38861	-	221
NW_015859602.1	38380	38904	-	524
NW_015859645.1	35212	35425	+	154
NW_015859709.1	25179	25378	+	199
NW_015859720.1	25738	25908	+	170
NW_015859920.1	17295	17384	-	89
NW_015859926.1	4440	4633	+	193
NW_015859926.1	4431	4663	+	213
NW_015860027.1	33143	33192	-	49
NW_015860134.1	12195	12325	-	122
NW_015860202.1	49484	49708	-	224
NW_015860202.1	193020	193145	+	125
NW_015860202.1	257601	257722	+	121
NW_015860375.1	37991	38030	-	39
NW_015860815.1	3208	3267	-	59
NW_015860891.1	114657	114807	+	150
NW_015860931.1	3402	3504	-	102
NW_015861087.1	38264	38305	+	41
NW_015861237.1	5253	5323	+	39

NW_015861375.1	86710	86794	-	84
NW_015861485.1	7969	8087	+	118
NW_015861531.1	13060	13226	+	86
NW_015861812.1	96442	96544	-	79
NW_015861812.1	7193	7332	+	139
NW_015861954.1	25527	25598	+	71
NW_015861972.1	71390	71498	-	108
NW_015862002.1	64098	64156	-	58
NW_015862325.1	1932	2098	+	166
NW_015862329.1	35545	35694	-	149
NW_015862380.1	66220	66309	-	89
NW_015862386.1	29067	29241	+	174
NW_015862391.1	24338	24486	-	148
NW_015862590.1	4582	4772	+	190
NW_015862657.1	13344	13412	+	68
NW_015862657.1	13342	13583	+	241
NW_015862677.1	13155	13335	+	180
NW_015862803.1	204036	204161	-	105
NW_015862827.1	24469	24634	-	124
NW_015863090.1	9241	9378	-	137
NW_015863115.1	117974	118108	+	134
NW_015863248.1	29196	29222	-	26
NW_015863429.1	25240	25374	+	134
NW_015863452.1	37755	37894	-	139
NW_015863514.1	18855	18907	+	52

NW_015863580.1	3045	3098	-	53
NW_015863628.1	69906	70050	-	144
NW_015863661.1	1440	1581	-	141
NW_015863722.1	22231	22349	+	118
NW_015863803.1	57848	57993	+	145
NW_015863999.1	70596	70695	+	99
NW_015864017.1	130677	130786	+	104
NW_015864136.1	29928	30278	+	350
NW_015864256.1	11482	11546	-	64
NW_015864327.1	55816	55982	-	166
NW_015864371.1	29651	29801	-	150
NW_015864383.1	39799	40094	+	295
NW_015864497.1	25805	25880	+	75
NW_015864532.1	133899	134062	-	163
NW_015864612.1	25994	26217	+	223
NW_015864719.1	48312	48387	+	75
NW_015864776.1	33868	33996	+	128
NW_015864801.1	41813	41939	-	70
NW_015864814.1	2502	2706	+	204
NW_015864846.1	36209	36519	+	310
NW_015864888.1	65534	65704	+	170
NW_015864951.1	12944	12996	-	52
NW_015865063.1	16609	16891	-	282
NW_015865095.1	53601	53732	+	131
NW_015865095.1	27790	27867	+	77

NW_015865174.1	62975	63178	-	162
NW_015865234.1	30732	30824	-	92
NW_015865234.1	30716	30844	-	128
NW_015865282.1	166820	166916	-	96
NW_015865436.1	47210	47248	-	38
NW_015865486.1	43	280	+	168
NW_015865642.1	49329	49394	-	65
NW_015865814.1	9408	9478	-	70
NW_015865988.1	21720	22012	-	224
NW_015866064.1	24605	24755	-	150
NW_015866246.1	18358	18599	+	181
NW_015866417.1	6240	6438	+	198
NW_015866492.1	44678	44711	-	33
NW_015866573.1	99411	99556	-	145
NW_015866642.1	90271	90457	+	186
NW_015866701.1	4774	4865	+	91
NW_015866788.1	77199	77359	-	160
NW_015866792.1	15974	16033	+	59
NW_015866883.1	11894	12024	+	130
NW_015867070.1	16162	16306	-	144
NW_015867277.1	15857	15916	+	59
NW_015867302.1	10203	10395	+	192
NW_015867332.1	52014	52106	+	92
NW_015867373.1	2899	2935	-	36
NW_015867385.1	114906	115173	-	267

NW_015867942.1	37312	37464	-	77
NW_015868019.1	117707	117848	-	141
NW_015868129.1	4344	4439	+	95
NW_015868313.1	27247	27271	+	24
NW_015868481.1	10694	10718	+	24
NW_015868801.1	21903	21985	-	82
NW_015868903.1	34211	34342	-	131
NW_015869052.1	108676	108735	-	59
NW_015869084.1	12091	12241	+	125
NW_015869203.1	19553	19718	+	165
NW_015869314.1	104261	104834	-	573
NW_015869399.1	15680	15739	-	59
NW_015869440.1	28066	28222	+	156
NW_015869440.1	28070	28118	+	48
NW_015869440.1	28076	28302	+	226
NW_015869475.1	21624	21712	-	88
NW_015869647.1	60256	60315	-	59
NW_015869858.1	128048	128184	+	136
NW_015869887.1	10911	11199	+	288
NW_015870068.1	40275	40454	-	179
NW_015870078.1	22488	22501	-	13
NW_015870188.1	9428	9479	-	51
NW_015870340.1	80515	80607	-	92
NW_015870502.1	13761	13974	-	97
NW_015870555.1	60725	60881	-	156

NW_015870571.1	72892	72926	+	34
NW_015870571.1	24527	24950	-	423
NW_015870571.1	72512	72784	+	272
NW_015870571.1	133718	133793	+	75
NW_015870571.1	149514	149571	+	57
NW_015870645.1	42915	43015	+	100
NW_015870747.1	126559	126663	-	104
NW_015870775.1	37556	37645	+	89
NW_015870919.1	11974	12108	+	134
NW_015870921.1	115878	115964	-	86
NW_015870921.1	161975	162101	+	126
NW_015871407.1	2714	2782	+	68
NW_015871411.1	7376	7568	+	192
NW_015871473.1	28698	28841	+	50
NW_015871536.1	28647	28844	+	110
NW_015871653.1	46544	46627	-	83
NW_015871737.1	24050	24189	-	139
NW_015871737.1	24056	24178	-	122
NW_015871856.1	6070	6106	+	36
NW_015871936.1	27585	27669	-	84
NW_015871965.1	10681	10759	+	78
NW_015871972.1	64575	64641	-	66
NW_015871972.1	69893	70070	-	121
NW_015872001.1	32349	32431	-	82
NW_015872033.1	57131	57229	-	98

NW_015872232.1	13010	13109	-	52
NW_015872723.1	7831	8114	+	274
NW_015872969.1	174741	174795	+	54
NW_015872981.1	128036	128311	+	275
NW_015873181.1	72704	72853	-	149
NW_015873382.1	6499	6557	-	58
NW_015873401.1	2233	2279	-	46
NW_015873493.1	69094	69194	-	100
NW_015873709.1	117778	117929	+	151
NW_015874034.1	55303	55360	+	57
NW_015874034.1	7317	7401	+	84
NW_015874093.1	55367	55562	+	195
NW_015874183.1	9717	9777	+	60
NW_015874389.1	24054	24160	-	104
NW_015874397.1	37275	37360	-	82
NW_015874484.1	17979	18196	+	210
NW_015874560.1	4150	4332	+	182
NW_015874560.1	4144	4329	+	185
NW_015874579.1	70579	70643	+	64
NW_015874598.1	1292	1479	+	187
NW_015874616.1	56707	56779	-	72
NW_015874831.1	79849	79943	+	94
NW_015874831.1	79842	79997	+	155
NW_015875056.1	104749	104809	-	60
NW_015875147.1	166	184	+	18

NW_015875291.1	31794	31908	+	114
NW_015875383.1	60206	60304	-	66
NW_015875415.1	33249	33382	-	133
NW_015875415.1	30967	30996	-	29
NW_015875526.1	27391	27475	-	84
NW_015875645.1	55196	55277	-	81
NW_015875805.1	12284	12438	-	76
NW_015875958.1	12492	12589	-	97
NW_015876058.1	2407	2484	+	77
NW_015876256.1	1331	1517	+	15
NW_015876339.1	60708	60800	+	92
NW_015876339.1	55665	55888	+	223
NW_015876465.1	51343	51397	+	54
NW_015876640.1	58278	58746	+	252
NW_015876645.1	4212	4474	+	262
NW_015876689.1	65466	65636	-	170
NW_015876772.1	8708	8821	+	113
NW_015876940.1	12181	12309	-	128
NW_015877118.1	20965	21037	+	72
NW_015877120.1	43686	43723	+	37
NW_015877132.1	71615	71656	+	41
NW_015877132.1	69257	69504	+	80
NW_015877220.1	125124	125180	-	48
NW_015877332.1	14272	14401	-	129
NW_015877345.1	66019	66160	-	141

NW_015877540.1	79325	79563	+	238
NW_015878004.1	13545	13789	-	244
NW_015878008.1	31961	32094	+	133
NW_015878092.1	47377	47513	-	136
NW_015878239.1	85587	85672	+	85
NW_015878320.1	72198	72466	-	268
NW_015878320.1	73772	74043	-	271
NW_015878553.1	8725	8841	-	116
NW_015878644.1	152089	152175	-	86
NW_015878668.1	96071	96278	-	75
NW_015878834.1	37136	37253	+	117
NW_015878853.1	15304	15462	-	158
NW_015879146.1	66250	66368	-	118
NW_015879312.1	67651	67771	-	120
NW_015879420.1	64929	65095	-	166
NW_015879465.1	3919	4092	+	173
NW_015879524.1	61309	61528	-	219
NW_015879524.1	67487	67613	-	126
NW_015880240.1	17770	17834	-	64
NW_015880286.1	15975	16065	+	90
NW_015880286.1	15969	16088	+	119
NW_015880302.1	42064	42133	-	69
NW_015880302.1	42023	42135	-	112
NW_015880450.1	99338	99465	+	127
NW_015880531.1	25562	25650	+	88

NW_015880531.1	25133	25174	+	41
NW_015880582.1	12236	12330	+	94
NW_015881121.1	112970	113087	+	117
NW_015881156.1	57155	57283	+	101
NW_015881211.1	12019	12357	+	338
NW_015881225.1	14130	14224	+	94
NW_015881441.1	24149	24265	-	116
NW_015881641.1	24935	25057	-	122
NW_015881706.1	6641	6760	-	119
NW_015881909.1	70035	70193	-	51
NW_015882161.1	5534	5675	+	141
NW_015882230.1	50546	50722	-	127
NW_015882343.1	59447	59513	-	66
NW_015882452.1	48817	48858	+	41
NW_015882749.1	6312	6446	-	134
NW_015882749.1	6314	6454	-	140
NW_015883260.1	11865	11984	+	119
NW_015883405.1	2514	2553	-	39
NW_015883454.1	28521	28631	+	110
NW_015883564.1	1635	1710	-	75
NW_015883696.1	19092	19641	+	549
NW_015883696.1	19077	19673	+	596
NW_015883933.1	434	547	-	113
NW_015883944.1	34941	34984	-	43
NW_015884381.1	89697	89788	-	91

NW_015884425.1	3898	3960	+	62
NW_015884425.1	3914	3951	+	37
NW_015884475.1	12105	12274	+	169
NW_015884615.1	6509	6526	-	17
NW_015884786.1	4017	4114	+	97
NW_015885042.1	7831	7900	-	69
NW_015885098.1	161728	161843	-	115
NW_015885149.1	43452	43580	+	128
NW_015885244.1	35796	35866	+	70
NW_015885262.1	39617	39737	-	120
NW_015885307.1	21500	21516	-	16
NW_015885320.1	79080	79223	-	143
NW_015885320.1	53636	53714	+	78
NW_015885396.1	122841	122893	-	52
NW_015885413.1	18449	18618	+	144
NW_015885435.1	26945	27007	-	62
NW_015885456.1	140373	140516	+	143
NW_015885516.1	21255	21347	-	92
NW_015885545.1	6954	7113	+	159
NW_015885571.1	21990	22128	-	138
NW_015885572.1	15151	15273	-	122
NW_015885572.1	15153	15311	-	156
NW_015885606.1	96703	96859	+	156
NW_015885618.1	52840	52909	-	69
NW_015885638.1	52853	53004	-	151

NW_015885730.1	30983	31044	+	61
NW_015885731.1	65679	65751	-	72
NW_015885731.1	43276	43396	+	120
NW_015885740.1	24848	24981	+	133
NW_015885797.1	21599	21688	+	89
NW_015885797.1	21614	21695	+	81
NW_015885814.1	24231	24336	-	105
NW_015885851.1	38576	38727	+	151
NW_015885869.1	31241	31276	+	35
NW_015885918.1	62694	62756	-	62
NW_015885938.1	86294	86381	+	87
NW_015886024.1	39679	39763	+	84
NW_015886055.1	36811	36893	+	82
NW_015886073.1	24340	24397	-	57
NW_015886146.1	18148	18386	+	127
NW_015886146.1	18128	18365	+	118
NW_015886150.1	15335	15436	-	101
NW_015886221.1	5814	5951	-	105
NW_015886224.1	8621	8802	-	181
NW_015886241.1	20577	20745	+	168
NW_015886265.1	62590	62637	+	47
NW_015886265.1	62589	62613	+	24
NW_015886316.1	8060	8238	+	178
NW_015886361.1	79699	79802	+	103
NW_015886472.1	9866	10054	+	66

NW_015886487.1	94008	94195	+	187
NW_015886530.1	65622	65766	+	144
NW_015886547.1	43820	43859	+	39
NW_015886690.1	10239	10343	+	16
NW_015886717.1	17273	17406	+	133
NW_015886730.1	29867	30005	-	138
NW_015886753.1	57827	58045	-	218
NW_015886798.1	69528	69613	+	85
NW_015886839.1	21032	21159	+	127
NW_015886839.1	46107	46160	-	53
NW_015886886.1	55049	55120	-	71
NW_015886917.1	29964	30149	-	185
NW_015886973.1	6314	6414	-	100
NW_015886977.1	192240	192381	+	141
NW_015886988.1	126699	126729	+	30
NW_015886993.1	6738	6803	+	65
NW_015887001.1	15057	15145	+	88
NW_015887012.1	53161	53363	-	202
NW_015887012.1	73843	73904	-	61
NW_015887108.1	4303	4411	+	108
NW_015887128.1	116942	117080	-	138
NW_015887163.1	18840	18947	+	107
NW_015887170.1	113885	113998	+	113
NW_015887232.1	79945	80026	-	81
NW_015887273.1	74010	74143	-	128

NW_015887275.1	5062	5111	+	49
NW_015887291.1	236927	237046	-	119
NW_015887346.1	56554	56674	+	120
NW_015887346.1	172433	172536	-	103
NW_015887350.1	3063	3674	+	611
NW_015887373.1	35089	35148	-	59
NW_015887373.1	176072	176171	+	99
NW_015887512.1	123419	123504	+	63
NW_015887512.1	116077	116323	+	246
NW_015887642.1	46724	46872	+	148
NW_015887684.1	78886	79101	-	213
NW_015887687.1	6054	6362	-	308
NW_015887730.1	14969	15003	+	34
NW_015887745.1	34551	34704	+	99
NW_015887761.1	41305	41372	+	67
NW_015887819.1	20861	21111	-	250
NW_015887828.1	46102	46213	-	64
NW_015887838.1	61386	61452	+	66
NW_015887841.1	82797	82868	+	71
NW_015887845.1	5829	5969	-	140
NW_015887883.1	35581	35629	-	48
NW_015887897.1	115757	115816	+	59
NW_015887908.1	53834	53915	+	81
NW_015887941.1	23156	23296	-	140
NW_015887950.1	18140	18289	+	149

NW_015887972.1	19743	19814	-	71
NW_015887976.1	78275	78338	+	63
NW_015887976.1	78714	78877	+	163
NW_015888000.1	41726	41806	-	80
NW_015888002.1	95158	95439	-	281
NW_015888035.1	12260	12501	+	241
NW_015888035.1	12634	12727	+	93
NW_015888051.1	37287	37400	-	73
NW_015888076.1	91359	91488	+	129
NW_015888119.1	29555	29821	+	266
NW_015888119.1	29509	30253	+	744
NW_015888133.1	13165	13263	-	98
NW_015888145.1	44870	44947	-	77
NW_015888209.1	133766	133873	+	107
NW_015888217.1	15140	15245	+	105
NW_015888225.1	18891	19016	+	125
NW_015888234.1	52016	52188	+	172
NW_015888271.1	58496	58556	-	51
NW_015888285.1	23463	23554	+	91
NW_015888345.1	62701	62805	-	104
NW_015888399.1	83338	83498	-	160
NW_015888421.1	9376	9452	-	10
NW_015888510.1	13928	14019	-	91
NW_015888536.1	49056	49137	+	81
NW_015888542.1	17160	17237	-	77

NW_015888555.1	41321	41402	+	81
NW_015888571.1	15178	15227	+	49
NW_015888575.1	56144	56272	-	128
NW_015888581.1	39488	39572	+	84
NW_015888593.1	10558	10583	-	25
NW_015888594.1	46405	46503	+	65
NW_015888634.1	7316	7449	-	133
NW_015888639.1	87934	88171	+	237
NW_015888640.1	86144	86222	-	78
NW_015888664.1	115042	115142	-	100
NW_015888701.1	21144	21333	+	175
NW_015888701.1	54015	54121	+	84
NW_015888785.1	161500	161617	-	117
NW_015888863.1	8320	8404	+	84
NW_015888866.1	7869	8049	+	180
NW_015888930.1	7321	7476	+	155
NW_015888938.1	16105	16208	+	103
NW_015889038.1	21322	21489	-	167
NW_015889070.1	9176	9271	-	66
NW_015889091.1	34819	34896	-	77
NW_015889108.1	10397	10493	-	96
NW_015889108.1	10404	10501	-	97
NW_015889136.1	104013	104132	+	119
NW_015889159.1	12008	12129	-	81
NW_015889159.1	11988	12101	-	101

NW_015889183.1	12224	12373	+	113
NW_015889206.1	62654	63112	+	416
NW_015889244.1	70326	70427	+	101
NW_015889250.1	37073	37283	+	120
NW_015889282.1	18521	18673	+	152
NW_015889306.1	22491	22522	-	31
NW_015889423.1	30168	30392	+	224
NW_015889469.1	12175	12242	+	67
NW_015889491.1	116626	116693	-	67
NW_015889493.1	13424	13518	-	94
NW_015889544.1	35798	35870	+	72
NW_015889571.1	35201	35494	-	150
NW_015889739.1	6118	6255	+	119
NW_015889739.1	6112	6266	+	130
NW_015889805.1	4332	4435	-	103
NW_015889806.1	39632	39736	+	104
NW_015889806.1	39641	39989	+	179
NW_015889821.1	8562	8691	-	71
NW_015889839.1	40014	40143	-	129
NW_015889858.1	146582	146712	+	130
NW_015889868.1	102429	102610	+	181
NW_015889929.1	15077	15266	-	189
NW_015889938.1	131980	132097	+	117
NW_015889985.1	22980	23058	-	78
NW_015890011.1	145179	145223	-	44

NW_015890018.1	25654	25722	-	68
NW_015890023.1	21245	21341	+	96
NW_015890026.1	55372	55560	+	19
NW_015890058.1	31916	32174	-	258
NW_015890340.1	19592	19652	-	60
NW_015890356.1	51300	51427	+	127
NW_015890435.1	32920	32971	+	51
NW_015890453.1	27069	27164	+	95
NW_015890453.1	40343	40624	-	261
NW_015890485.1	41063	41153	+	90
NW_015890530.1	18703	18847	-	144
NW_015890557.1	72561	72647	+	86
NW_015890589.1	91777	91924	+	105
NW_015890619.1	47845	47949	+	104
NW_015890639.1	84105	84287	+	182
NW_015890776.1	24141	24268	-	127
NW_015890836.1	23607	23670	-	63
NW_015890836.1	6520	6663	+	127
NW_015890856.1	36697	36786	+	89
NW_015890876.1	47283	47451	+	168
NW_015890884.1	60620	60801	-	177
NW_015890913.1	25459	25510	+	51
NW_015890936.1	63849	63994	-	145
NW_015890942.1	37354	37734	-	380
NW_015890994.1	116366	116503	-	137

NW_015891021.1	103482	103580	-	98
NW_015891058.1	31495	31655	+	160
NW_015891103.1	66728	66806	-	78
NW_015891104.1	193335	193466	-	73
NW_015891132.1	9017	9120	-	103
NW_015891143.1	12634	13068	-	434
NW_015891143.1	12625	13066	-	441
NW_015891161.1	25359	25417	+	58
NW_015891230.1	40154	40351	+	107
NW_015891245.1	16820	16926	-	106
NW_015891273.1	60231	60296	-	65
NW_015891307.1	10715	10836	+	121
NW_015891443.1	195667	196015	+	242
NW_015891445.1	24348	24450	+	102
NW_015891461.1	90914	91049	+	135
NW_015891515.1	10954	11120	-	166
NW_015891517.1	27737	27865	+	128
NW_015891551.1	18984	19162	+	178
NW_015891641.1	34957	35009	-	52
NW_015891655.1	1565	1938	+	344
NW_015891675.1	37837	37982	-	145
NW_015891723.1	77953	78053	+	100
NW_015891723.1	79815	79892	+	77
NW_015891727.1	46212	46277	+	65
NW_015891738.1	39668	39717	-	35

NW_015891793.1	16595	16711	+	116
NW_015891796.1	63864	63955	-	91
NW_015891867.1	280291	280436	+	145
NW_015892128.1	26842	26960	+	86
NW_015892151.1	16057	16165	+	108
NW_015892219.1	16217	16308	-	91
NW_015892249.1	28079	28280	+	201
NW_015892304.1	31592	31741	+	149
NW_015892338.1	11887	12051	+	164
NW_015892342.1	178206	178321	+	115
NW_015892427.1	97941	98092	-	151
NW_015892642.1	46554	46776	+	222
NW_015893144.1	782	851	+	69
NW_015893144.1	746	985	+	155
NW_015893145.1	56592	56646	-	54
NW_015893245.1	28501	28593	+	92
NW_015893661.1	24552	24633	-	81
NW_015893945.1	15870	15909	+	39
NW_015894107.1	820	877	+	57
NW_015894251.1	607	786	+	156
NW_015894633.1	23579	23790	+	211
NW_015894875.1	334	642	+	308
NW_015895023.1	142	425	+	237
NW_015895536.1	28741	28882	-	141
NW_015895536.1	28730	28888	-	158

NW_015895657.1	79531	79674	-	143
NW_015896246.1	1423	1508	-	85
NW_015896586.1	12474	12664	-	190
NW_015896652.1	3482	3572	+	90
NW_015896893.1	20089	20143	+	54
NW_015896999.1	1438	1522	+	84
NW_015897342.1	23030	23090	+	60
NW_015897495.1	4090	4210	+	120
NW_015897495.1	3972	4178	+	206
NW_015897571.1	1956	2047	-	91
NW_015897571.1	1961	2037	-	76
NW_015897627.1	94408	94518	-	110
NW_015897767.1	2179	2441	+	262
NW_015897824.1	8613	8686	+	73
NW_015897840.1	537	727	-	102
NW_015897856.1	39665	39693	-	28
NW_015897880.1	2384	2604	+	111
NW_015897911.1	44435	44776	+	341
NW_015898218.1	95277	95339	-	62
NW_015898292.1	2329	2389	-	60
NW_015898468.1	343	421	+	50
NW_015898584.1	4483	4535	-	52
NW_015898590.1	1125	1238	+	48
NW_015898644.1	229	307	+	78
NW_015898644.1	228	326	+	98

NW_015898674.1	2587	2812	+	225
NW_015898700.1	1719	1806	-	87
NW_015898700.1	4289	4503	-	36
NW_015898776.1	2557	2740	+	123
NW_015898845.1	2379	2516	+	137
NW_015898861.1	5318	5571	-	253
NW_015898877.1	4117	4259	-	142
NW_015898877.1	6266	6435	-	169
NW_015898921.1	3915	4063	+	148
NW_015898945.1	7386	7514	-	128
NW_015898954.1	2717	2908	+	191
NW_015898962.1	2425	2538	-	113
NW_015898966.1	73	151	+	2
NW_015898986.1	392	501	+	109
NW_015899006.1	6135	6454	-	131
NW_015899067.1	8489	8551	+	62
NW_015899071.1	17782	17813	-	31
NW_015899071.1	17730	17814	-	84
NW_015899120.1	71646	71775	-	129
NW_015899134.1	41271	41379	-	108
NW_015899136.1	32216	32415	-	199
NW_015899221.1	20723	20901	-	178
NW_015899232.1	46538	46822	-	283
NW_015899245.1	37617	37703	+	86
NW_015899255.1	26406	26469	+	63

NW_015899301.1	4019	4069	-	50
NW_015899321.1	17286	17366	-	80
NW_015899399.1	9036	9209	+	173
NW_015899443.1	107370	107438	+	68
NW_015899445.1	152393	152547	+	154
NW_015899445.1	234231	234304	+	73
NW_015899445.1	257108	257178	-	70
NW_015899449.1	43155	43411	-	107
NW_015899513.1	77969	78057	-	88
NW_015899518.1	63397	63701	-	212
NW_015899519.1	23164	23204	-	40
NW_015899519.1	43183	43250	+	67
NW_015899519.1	23176	23228	-	52
NW_015899562.1	18569	18709	-	140
NW_015899566.1	43004	43185	+	181
NW_015899574.1	37563	37636	+	73
NW_015899637.1	104222	104287	-	65
NW_015899637.1	123594	123759	+	165
NW_015899664.1	16966	17102	-	136
NW_015899720.1	21630	21733	-	103
NW_015899772.1	8784	8969	-	185
NW_015899800.1	21374	21688	-	174
NW_015899802.1	22825	22960	+	135
NW_015899847.1	61109	61206	+	97
NW_015899852.1	53546	53631	-	85

NW_015899892.1	59623	59773	-	107
NW_015899892.1	59609	59834	-	121
NW_015899892.1	59604	59826	-	126
NW_015899912.1	27302	27484	-	182
NW_015899943.1	33339	33499	+	160
NW_015899959.1	51759	51999	-	240
NW_015899985.1	19739	19810	+	71
NW_015900085.1	56780	56869	+	89
NW_015900104.1	23752	23947	+	166
NW_015900116.1	5376	5532	+	156
NW_015900242.1	15624	15745	+	121
NW_015900331.1	45820	45970	+	150
NW_015900433.1	102957	103032	+	75
NW_015900434.1	18852	18916	-	64
NW_015900434.1	18850	18942	-	92
NW_015900449.1	78166	78239	+	73
NW_015900449.1	78146	78265	+	119
NW_015900541.1	16281	16338	-	57
NW_015900541.1	16266	16349	-	83
NW_015900580.1	5321	5540	-	219
NW_015900580.1	5669	5934	-	149
NW_015900631.1	38763	38808	+	45
NW_015900673.1	61431	61577	-	146
NW_015900676.1	22488	22579	+	75
NW_015900711.1	39528	39685	+	157

NW_015900711.1	85647	85752	-	105
NW_015900807.1	70121	70263	-	142
NW_015900807.1	70109	70268	-	159
NW_015900944.1	16388	16440	-	52
NW_015900953.1	192869	193021	+	99
NW_015901067.1	8047	8208	-	161
NW_015901126.1	84255	84464	-	209
NW_015901130.1	27303	27389	-	86
NW_015901231.1	19640	19717	-	77
NW_015901249.1	48783	48868	-	85
NW_015901257.1	45106	45147	-	41
NW_015901301.1	29044	29139	+	95
NW_015901333.1	57375	57480	+	105
NW_015901390.1	198699	198806	+	107
NW_015901442.1	3869	3923	+	54
NW_015901464.1	6087	6130	+	43
NW_015901507.1	17257	17336	-	79
NW_015901597.1	18399	18555	+	102
NW_015901739.1	54995	55087	-	92
NW_015901798.1	14445	14487	+	42
NW_015901834.1	49909	50004	+	95
NW_015901933.1	13158	13307	-	149
NW_015902105.1	30268	30458	+	190
NW_015902130.1	3291	3373	+	82
NW_015902156.1	17448	17627	+	84

NW_015902213.1	50661	50936	+	275
NW_015902217.1	41766	41889	-	123
NW_015902239.1	24220	24357	+	137
NW_015902293.1	35857	36002	+	145
NW_015902300.1	186706	186729	+	23
NW_015902311.1	16007	16290	+	283
NW_015902330.1	108553	108665	-	112
NW_015902347.1	18526	18595	-	69
NW_015902379.1	97183	97294	-	111
NW_015902389.1	34999	35082	+	83
NW_015902436.1	65465	65613	+	125
NW_015902457.1	20542	20678	+	136
NW_015902507.1	13884	14385	-	476
NW_015902526.1	23502	23618	+	116
NW_015902530.1	21491	21594	-	103
NW_015902628.1	34444	34479	-	35
NW_015902638.1	69956	70226	+	270
NW_015902845.1	2806	3101	+	295
NW_015902877.1	52062	52147	-	85
NW_015902891.1	26099	26208	+	109
NW_015902905.1	77863	78082	+	219
NW_015902918.1	31480	31842	+	362
NW_015902918.1	31433	31835	+	402
NW_015903036.1	8231	8312	+	81
NW_015903099.1	32652	32736	-	84

NW_015903115.1	84869	85003	-	80
NW_015903184.1	6108	6231	+	123
NW_015903239.1	9590	9670	+	80
NW_015903270.1	26215	26310	-	95
NW_015903281.1	59442	59549	+	107
NW_015903294.1	9638	9680	+	42
NW_015903340.1	3488	3594	-	87
NW_015903347.1	92317	92553	+	236
NW_015903404.1	37307	37471	-	164
NW_015903409.1	117156	117289	+	133
NW_015903420.1	33193	33363	+	170
NW_015903438.1	30799	30948	+	149
NW_015903464.1	25979	26153	+	174
NW_015903536.1	50235	50504	-	269
NW_015903570.1	62667	62775	-	70
NW_015903570.1	20306	20518	+	212
NW_015903624.1	13880	13956	-	76
NW_015903641.1	66165	66403	+	238
NW_015903716.1	57865	58168	+	303
NW_015903722.1	167251	167404	-	153
NW_015903864.1	13478	13529	+	51
NW_015903924.1	21607	21649	-	42
NW_015903924.1	21599	21643	-	44
NW_015903962.1	12543	12635	+	50
NW_015903962.1	12530	12642	+	63

NW_015903976.1	36617	36723	-	106
NW_015903984.1	143633	143708	+	75
NW_015904002.1	45438	45569	+	131
NW_015904094.1	29555	29688	-	133
NW_015904213.1	7255	7441	-	186
NW_015904216.1	166617	166838	-	221
NW_015904216.1	166442	166850	-	408
NW_015904250.1	189421	189574	+	153
NW_015904261.1	120808	120952	-	144
NW_015904282.1	191	259	-	68
NW_015904349.1	26893	26979	+	86
NW_015904370.1	149574	149735	-	161
NW_015904383.1	25998	26055	-	57
NW_015904437.1	16716	17205	-	489
NW_015904444.1	31333	31489	+	156
NW_015904444.1	31300	31480	+	180
NW_015904459.1	11804	11897	+	83
NW_015904478.1	44489	44662	-	173
NW_015904478.1	44623	44656	-	33
NW_015904532.1	75237	75398	-	161
NW_015904552.1	24831	25122	-	202
NW_015904568.1	23478	23581	-	103
NW_015904592.1	8449	8506	-	57
NW_015904622.1	4414	4452	+	38
NW_015904671.1	13818	13974	-	156

NW_015904709.1	43832	43894	-	62
NW_015904717.1	69379	69528	-	149
NW_015904717.1	69264	69593	-	329
NW_015904717.1	36004	36070	+	66
NW_015904737.1	197846	198002	+	123
NW_015904798.1	234555	234584	-	29
NW_015904798.1	234470	234661	-	191
NW_015904876.1	23201	23297	-	96
NW_015904943.1	85746	85907	+	161
NW_015905071.1	36361	36634	+	273
NW_015905085.1	40087	40302	+	112
NW_015905140.1	28229	28423	+	194
NW_015905170.1	189625	189675	+	50
NW_015905175.1	17746	18086	-	340
NW_015905192.1	108783	108988	+	205
NW_015905204.1	39600	39647	+	47
NW_015905204.1	39592	39651	+	59
NW_015905228.1	48583	48678	-	95
NW_015905264.1	55899	56013	-	114
NW_015905338.1	69256	69401	+	145
NW_015905354.1	61978	62180	+	202
NW_015905382.1	8425	8444	+	19
NW_015905415.1	29321	29421	+	62
NW_015905711.1	8248	8421	+	173
NW_015905759.1	21150	21204	+	54

NW_015905783.1	92183	92490	+	307
NW_015905785.1	2923	2996	+	73
NW_015905802.1	56760	56858	+	98
NW_015905877.1	55021	55111	-	90
NW_015905884.1	104677	104802	-	125
NW_015905901.1	30350	30427	-	77
NW_015905941.1	41161	41279	-	118
NW_015906117.1	5262	5348	+	86
NW_015906117.1	5727	5769	-	42
NW_015906215.1	42232	42274	-	42
NW_015906218.1	19331	19373	+	42
NW_015906218.1	21333	21498	+	144
NW_015906221.1	44856	44924	+	68
NW_015906221.1	46336	46441	-	105
NW_015906340.1	50255	50347	+	92
NW_015906340.1	50278	50358	+	80
NW_015906375.1	95084	95336	+	252
NW_015906375.1	95121	95386	+	265
NW_015906375.1	95090	95377	+	287
NW_015906389.1	38401	38483	-	82
NW_015906401.1	45531	45965	-	434
NW_015906464.1	14268	14468	+	200
NW_015906465.1	31001	31112	-	111
NW_015906512.1	69892	69952	-	60
NW_015906548.1	124449	124523	-	74

NW_015906584.1	14814	15032	-	218
NW_015906621.1	116528	116569	+	41
NW_015906643.1	15960	16047	-	62
NW_015906659.1	80979	81042	-	63
NW_015906751.1	18252	18397	+	145
NW_015906829.1	33005	33473	-	468
NW_015906998.1	14279	14359	+	80
NW_015907009.1	2521	2558	-	37
NW_015907068.1	37029	37167	+	138
NW_015907210.1	50078	50105	+	27
NW_015907228.1	17818	17864	-	46
NW_015907265.1	37134	37239	+	105
NW_015907265.1	37139	37252	+	113
NW_015907331.1	41345	41532	+	187
NW_015907331.1	112062	112237	+	175
NW_015907349.1	146723	146881	+	158
NW_015907369.1	61159	61379	-	220
NW_015907430.1	27752	27814	+	62
NW_015907486.1	28719	28756	+	37
NW_015907494.1	10201	10311	+	110
NW_015907531.1	2196	2554	-	358
NW_015907600.1	14168	14237	+	69
NW_015907646.1	71933	72139	-	206
NW_015907664.1	21251	21292	+	41
NW_015907678.1	4858	4996	+	138

NW_015907696.1	23097	23205	-	108
NW_015907711.1	4696	4721	-	25
NW_015907716.1	82778	82928	-	150
NW_015907731.1	11130	11304	-	174
NW_015907781.1	7072	7284	-	212
NW_015907904.1	19549	19685	-	136
NW_015907940.1	82442	82513	+	71
NW_015907986.1	26062	26207	-	145
NW_015907987.1	8120	8409	+	289
NW_015908027.1	8239	8314	-	75
NW_015908170.1	14531	14811	-	280
NW_015908206.1	33539	33619	+	80
NW_015908240.1	690	717	+	27
NW_015908246.1	15178	15282	-	104
NW_015908270.1	68458	68625	-	94
NW_015908286.1	5518	5579	-	61
NW_015908345.1	34143	34183	+	40
NW_015908351.1	29577	29642	+	65
NW_015908367.1	13505	13610	+	105
NW_015908394.1	45229	45317	+	88
NW_015908411.1	107029	107118	+	89
NW_015908472.1	65119	65332	+	213
NW_015908474.1	29209	29311	+	102
NW_015908492.1	22912	22959	-	47
NW_015908550.1	37841	37949	-	108

NW_015908554.1	7861	7908	+	47
NW_015908580.1	29548	29633	+	43
NW_015908580.1	29419	29623	+	170
NW_015908584.1	32474	32845	-	371
NW_015908633.1	55236	55794	+	558
NW_015908648.1	25616	25847	-	231
NW_015908687.1	5801	5979	-	178
NW_015908689.1	60077	60176	+	99
NW_015908700.1	76637	76761	+	124
NW_015908714.1	97433	97572	-	139
NW_015908714.1	97447	97675	-	228
NW_015908758.1	26483	26621	-	138
NW_015908758.1	26980	27067	-	87
NW_015908769.1	40845	40946	+	101
NW_015908769.1	82941	83072	+	131
NW_015908799.1	7365	7530	+	165
NW_015908843.1	25901	26237	+	336
NW_015908848.1	88574	88818	+	244
NW_015908886.1	17024	17212	+	188
NW_015908918.1	54103	54227	+	124
NW_015908939.1	32034	32163	+	129
NW_015908946.1	19901	20091	+	114
NW_015908950.1	22418	22461	-	43
NW_015908950.1	33013	33055	+	42
NW_015908986.1	14678	14777	-	99

NW_015909015.1	87435	87534	-	99
NW_015909017.1	19594	19681	+	87
NW_015909022.1	6329	6421	-	67
NW_015909023.1	84884	85036	-	152
NW_015909108.1	10727	10803	-	76
NW_015909198.1	23072	23212	-	140
NW_015909244.1	8282	8465	+	97
NW_015909256.1	25522	25693	-	171
NW_015909272.1	28449	28564	-	115
NW_015909306.1	121993	122091	-	98
NW_015909421.1	57686	57811	-	125
NW_015909533.1	32963	32991	+	28
NW_015909536.1	43583	43634	+	51
NW_015909536.1	43587	43657	+	70
NW_015909562.1	28997	29129	-	132
NW_015909565.1	28668	28766	+	98
NW_015909610.1	13629	13842	+	213
NW_015909610.1	13655	13835	+	180
NW_015909627.1	49408	49449	-	41
NW_015909633.1	15959	16050	+	91
NW_015909681.1	15302	15470	-	168
NW_015909848.1	52041	52260	-	219
NW_015909858.1	32158	32198	-	40
NW_015909909.1	106722	107034	+	312
NW_015909951.1	28946	28995	-	49

NW_015909984.1	72766	72999	-	233
NW_015909995.1	71505	71582	+	77
NW_015910036.1	16815	17214	-	399
NW_015910106.1	54843	55020	-	177
NW_015910124.1	18449	18631	-	182
NW_015910130.1	3081	3348	+	267
NW_015910399.1	11802	11854	-	52
NW_015910399.1	11806	11870	-	64
NW_015910487.1	22947	23094	+	147
NW_015910523.1	35072	35237	+	165
NW_015910523.1	35042	35468	+	426
NW_015910536.1	13833	14084	-	251
NW_015910566.1	122416	122518	-	102
NW_015910566.1	122419	122554	-	135
NW_015910619.1	35354	35413	-	59
NW_015910658.1	229900	230043	+	143
NW_015910710.1	31605	31670	-	65
NW_015910719.1	20070	20194	+	124
NW_015910734.1	18570	18748	+	178
NW_015910734.1	18895	18943	+	48
NW_015910755.1	111675	111889	+	214
NW_015910764.1	40800	40884	-	79
NW_015910837.1	19663	19785	-	122
NW_015910852.1	11692	11992	+	300
NW_015910887.1	89269	89324	-	55

NW_015910898.1	16331	16581	+	250
NW_015910921.1	14958	15024	-	66
NW_015910924.1	21189	21295	-	106
NW_015910939.1	159871	160005	+	77
NW_015910959.1	74610	74833	-	223
NW_015910990.1	8913	9077	+	164
NW_015911153.1	161941	162192	+	251
NW_015911158.1	5793	5848	-	55
NW_015911170.1	32809	32920	-	111
NW_015911212.1	61985	62077	+	92
NW_015911271.1	70007	70167	+	160
NW_015911293.1	28758	28893	-	135
NW_015911403.1	23945	24005	-	60
NW_015911410.1	129511	129742	+	231
NW_015911468.1	24001	24084	+	83
NW_015911561.1	51809	52016	-	207
NW_015911649.1	25674	25791	-	117
NW_015911663.1	69511	69630	-	76
NW_015911677.1	44341	44462	-	121
NW_015911706.1	125827	125932	-	105
NW_015911743.1	126337	126440	+	103
NW_015911774.1	32448	32572	+	124
NW_015911790.1	26695	26854	-	159
NW_015911817.1	16294	16419	-	75
NW_015911857.1	32434	32573	-	139

NW_015911876.1	23618	23792	-	174
NW_015911940.1	4416	4473	-	57
NW_015911963.1	43440	43574	-	134
NW_015912003.1	41339	41491	+	152
NW_015912018.1	15284	15375	+	90
NW_015912040.1	7587	7686	+	99
NW_015912041.1	2048	2092	-	44
NW_015912050.1	37001	37293	+	292
NW_015912095.1	74918	75049	-	131
NW_015912105.1	170750	170887	+	137
NW_015912131.1	56861	56967	+	106
NW_015912224.1	7732	7906	+	174
NW_015912261.1	27600	27858	+	258
NW_015912299.1	95774	95932	+	132
NW_015912307.1	90419	90761	-	342
NW_015912317.1	3867	3926	+	59
NW_015912317.1	17928	18058	-	130
NW_015912323.1	25934	25991	+	57
NW_015912333.1	25881	25986	+	105
NW_015912402.1	23540	23669	-	129
NW_015912433.1	3502	3743	+	193
NW_015912591.1	4807	5089	-	282
NW_015912591.1	20889	21105	+	216
NW_015912835.1	47805	47931	+	126
NW_015912861.1	15639	15717	-	78

NW_015912908.1	6826	6922	+	96
NW_015912909.1	26887	26903	-	16
NW_015912913.1	5613	5751	-	138
NW_015913018.1	42301	42398	+	97
NW_015913181.1	19496	19596	-	100
NW_015913300.1	1929	1990	+	61
NW_015913302.1	21802	21857	+	46
NW_015913333.1	64872	64985	+	113
NW_015913333.1	13081	13221	-	140
NW_015913361.1	47008	47105	-	97
NW_015913397.1	257040	257134	-	94
NW_015913441.1	28883	29024	+	141
NW_015913495.1	41618	41825	-	104
NW_015913538.1	22732	22996	-	264
NW_015913548.1	18194	18241	-	47
NW_015913592.1	24432	24533	-	80
NW_015913593.1	36659	36726	-	67
NW_015913653.1	44086	44168	-	82
NW_015913686.1	35758	35795	-	37
NW_015913690.1	135134	135189	-	55
NW_015913702.1	88126	88334	+	208
NW_015913720.1	47036	47329	+	195
NW_015913724.1	17878	18090	+	212
NW_015913725.1	38175	38522	+	347
NW_015913733.1	24834	24932	-	98

NW_015913743.1	153092	153191	-	99
NW_015913743.1	144064	144148	+	84
NW_015913757.1	24588	24735	+	147
NW_015913770.1	57015	57071	-	56
NW_015913845.1	44636	44732	-	96
NW_015913856.1	60120	60226	+	106
NW_015913856.1	60111	60253	+	142
NW_015913909.1	60040	60139	+	99
NW_015913926.1	93001	93107	-	106
NW_015913926.1	92992	93101	-	109
NW_015913926.1	92986	93104	-	118
NW_015913946.1	25634	25745	-	111
NW_015914009.1	4745	4928	-	183
NW_015914025.1	37162	37245	+	83
NW_015914045.1	14060	14202	-	142
NW_015914055.1	149211	149538	-	235
NW_015914088.1	10657	10723	-	66
NW_015914106.1	2097	2189	+	92
NW_015914121.1	75052	75261	+	165
NW_015914148.1	92815	92906	+	91
NW_015914152.1	35392	35558	+	166
NW_015914241.1	39569	39699	-	86
NW_015914336.1	8910	8964	-	54
NW_015914468.1	10097	10211	+	114
NW_015914492.1	34833	35058	+	217

NW_015914508.1	15293	15377	+	84
NW_015914528.1	99129	99286	-	142
NW_015914562.1	6095	6178	+	83
NW_015914675.1	45493	45607	+	114
NW_015914701.1	29720	29779	+	59
NW_015914861.1	1473	1545	+	72
NW_015914908.1	108041	108150	-	109
NW_015914929.1	52278	52417	-	139
NW_015914974.1	7944	8019	-	75
NW_015914999.1	25098	25174	-	76
NW_015915042.1	22869	22978	+	109
NW_015915051.1	175892	176529	+	562
NW_015915055.1	49487	49709	+	222
NW_015915163.1	10411	10498	-	87
NW_015915247.1	14077	14176	+	99
NW_015915248.1	13870	13906	+	36
NW_015915293.1	65267	65513	-	246
NW_015915412.1	11149	11210	-	61
NW_015915476.1	35381	35440	+	59
NW_015915503.1	5948	6011	+	63
NW_015915503.1	5951	6013	+	62
NW_015915612.1	3787	3847	-	60
NW_015915657.1	48208	48343	-	135
NW_015915699.1	43805	43875	+	70
NW_015915700.1	19405	19551	+	146

NW_015915700.1	19657	19717	+	60
NW_015915705.1	96570	96761	+	113
NW_015915722.1	85490	85517	+	27
NW_015915722.1	68177	68301	-	124
NW_015915834.1	186685	186834	-	113
NW_015915893.1	37173	37345	+	172
NW_015915893.1	14549	14715	+	166
NW_015915980.1	59639	59718	-	79
NW_015915983.1	42839	42897	+	58
NW_015916011.1	86972	87056	-	84
NW_015916059.1	56469	56809	+	340
NW_015916075.1	126518	126709	-	191
NW_015916098.1	12624	12711	+	87
NW_015916098.1	21118	21303	+	185
NW_015916119.1	7448	7624	-	176
NW_015916130.1	32867	32969	-	102
NW_015916168.1	2927	2982	+	55
NW_015916170.1	21223	21367	-	144
NW_015916178.1	26251	26337	+	86
NW_015916259.1	32218	32270	-	52
NW_015916273.1	32080	32243	+	163
NW_015916311.1	35711	35777	+	66
NW_015916319.1	55839	55989	+	150
NW_015916372.1	71639	71847	+	208
NW_015916373.1	124123	124224	-	101

NW_015916405.1	20335	20421	-	86
NW_015916418.1	38165	38326	-	161
NW_015916474.1	11088	11417	-	292
NW_015916480.1	152254	152370	+	116
NW_015916498.1	56591	56727	-	136
NW_015916525.1	67906	67979	-	73
NW_015916526.1	109070	109384	-	314
NW_015916540.1	5107	5162	-	55
NW_015916602.1	29602	29814	-	212
NW_015916630.1	39580	39643	-	63
NW_015916670.1	20071	20122	+	51
NW_015916713.1	32428	32527	-	99
NW_015916753.1	16472	16713	-	146
NW_015916964.1	23039	23107	+	68
NW_015916967.1	64974	65072	+	98
NW_015917019.1	8427	8655	+	228
NW_015917019.1	33321	33496	-	175
NW_015917026.1	31750	31825	-	75
NW_015917044.1	12010	12133	+	123
NW_015917079.1	23417	23515	+	98
NW_015917081.1	38052	38167	+	115
NW_015917211.1	90968	91042	+	74
NW_015917302.1	7267	7355	+	88
NW_015917302.1	7053	7454	+	401
NW_015917319.1	26985	27054	+	69

NW_015917413.1	116887	117039	+	152
NW_015917434.1	46165	46306	+	141
NW_015917434.1	64980	65105	-	125
NW_015917434.1	46159	46331	+	172
NW_015917456.1	1690	1766	-	76
NW_015917575.1	37629	37707	-	78
NW_015917694.1	64103	64284	-	181
NW_015917721.1	154270	154435	-	165
NW_015917737.1	18040	18189	-	149
NW_015917737.1	23801	24031	-	230
NW_015917758.1	78078	78134	+	56
NW_015917758.1	78086	78156	+	70
NW_015917798.1	47659	47713	-	54
NW_015917798.1	47638	47808	-	92
NW_015917959.1	7151	7278	-	127
NW_015918015.1	33140	33228	+	88
NW_015918033.1	20012	20171	+	159
NW_015918215.1	45370	45501	-	131
NW_015918218.1	6408	6473	-	65
NW_015918243.1	27403	27544	+	141
NW_015918253.1	86855	86951	-	96
NW_015918253.1	89295	89373	-	78
NW_015918332.1	72342	72680	+	276
NW_015918392.1	12371	12467	+	96
NW_015918400.1	7549	7656	+	107

NW_015918461.1	1354	1503	+	149
NW_015918466.1	19511	19599	+	88
NW_015918523.1	16141	16787	-	646
NW_015918553.1	8728	8827	-	99
NW_015918638.1	14765	14864	-	99
NW_015918920.1	46189	46342	-	153
NW_015918920.1	15962	16131	-	169
NW_015918970.1	39091	39166	+	75
NW_015918993.1	52547	52675	+	128
NW_015918995.1	7092	7222	+	130
NW_015919008.1	24390	24512	-	122
NW_015919010.1	115400	115515	+	115
NW_015919083.1	11519	11620	+	101
NW_015919083.1	11532	11622	+	90
NW_015919176.1	23609	23680	-	71
NW_015919185.1	160049	160235	-	186
NW_015919185.1	174499	174552	+	53
NW_015919220.1	66670	66749	-	79
NW_015919243.1	79632	79701	-	69
NW_015919243.1	79595	79724	-	129
NW_015919284.1	22230	22356	+	86
NW_015919314.1	14299	14447	+	148
NW_015919368.1	87757	88272	+	444
NW_015919412.1	47683	47775	+	92
NW_015919444.1	22528	22595	+	67

NW_015919522.1	92665	92790	-	73
NW_015919548.1	45199	45240	-	41
NW_015919566.1	36262	36404	+	142
NW_015919598.1	20666	20751	-	85
NW_015919648.1	61166	61326	+	160
NW_015919660.1	48947	49060	-	113
NW_015919748.1	15956	16067	-	111
NW_015919779.1	10574	10686	+	112
NW_015919794.1	5956	6052	+	96
NW_015919874.1	43019	43055	-	36
NW_015919885.1	7243	7430	-	187
NW_015919934.1	125816	125861	+	45
NW_015919953.1	30109	30148	+	39
NW_015919982.1	32318	32412	-	94
NW_015920003.1	59508	59622	-	114
NW_015920065.1	5118	5289	+	171
NW_015920065.1	2830	2953	+	123
NW_015920103.1	41190	41335	-	145
NW_015920115.1	64437	64675	-	238
NW_015920168.1	19421	19460	-	39
NW_015920281.1	89418	89473	-	51
NW_015920298.1	62412	62630	+	218
NW_015920320.1	11947	12055	-	108
NW_015920442.1	49678	49724	-	46
NW_015920546.1	62652	62716	-	64

NW_015920546.1	62501	62731	-	230
NW_015920636.1	585	670	+	85
NW_015920784.1	25349	25453	-	104
NW_015920784.1	35420	35515	-	95
NW_015920834.1	63409	63495	+	86
NW_015920867.1	197421	197521	+	100
NW_015920870.1	8694	8748	+	47
NW_015920870.1	22532	22710	+	178
NW_015920889.1	4078	4206	+	128
NW_015920893.1	2442	2525	-	83
NW_015920910.1	23804	23984	+	180
NW_015921032.1	11823	11964	-	141
NW_015921142.1	26032	26124	+	92
NW_015921262.1	15295	15371	-	76
NW_015921297.1	17443	17482	-	39
NW_015921331.1	56347	56492	+	145
NW_015921331.1	926	976	+	50
NW_015921424.1	43436	43739	-	303
NW_015921424.1	43567	43775	-	208
NW_015921443.1	15265	15344	+	79
NW_015921443.1	15260	15414	+	154
NW_015921455.1	44746	44789	+	43
NW_015921455.1	44765	44896	+	105
NW_015921455.1	41177	41253	+	76
NW_015921476.1	20827	20897	-	70

NW_015921485.1	86290	86317	+	27
NW_015921499.1	17671	17764	+	93
NW_015921505.1	15741	15764	+	23
NW_015921526.1	29576	29661	-	59
NW_015921526.1	29513	29677	-	75
NW_015921570.1	23059	23155	+	96
NW_015921645.1	29383	29836	-	453
NW_015921646.1	71555	71585	+	30
NW_015921656.1	19901	20008	-	61
NW_015921660.1	52535	52623	-	88
NW_015921739.1	3103	3162	+	59
NW_015921743.1	177730	177800	+	70
NW_015921743.1	140405	140494	+	89
NW_015921757.1	39863	39961	+	98
NW_015921875.1	25341	25504	-	163
NW_015921940.1	8707	8772	-	65
NW_015921985.1	86293	86428	-	135
NW_015922001.1	32844	32961	-	117
NW_015922044.1	11129	11214	-	85
NW_015922079.1	11413	11501	+	88
NW_015922099.1	5490	5608	-	118
NW_015922140.1	15813	15898	-	57
NW_015922165.1	8071	8164	-	93
NW_015922166.1	27969	28059	-	90
NW_015922234.1	6095	6444	-	349

NW_015922253.1	14147	14209	+	62
NW_015922253.1	4354	4732	+	378
NW_015922253.1	37156	37315	+	159
NW_015922253.1	38404	38484	+	80
NW_015922346.1	17044	17084	+	40
NW_015922346.1	17042	17093	+	51
NW_015922398.1	24875	25063	-	188
NW_015922412.1	34109	34182	+	73
NW_015922419.1	36704	36838	+	49
NW_015922419.1	40626	40755	+	129
NW_015922419.1	51347	51439	-	92
NW_015922419.1	36698	36842	+	80
NW_015922458.1	47475	47508	-	33
NW_015922480.1	18402	18455	-	53
NW_015922590.1	66641	66773	-	132
NW_015922598.1	5177	5223	-	46
NW_015922598.1	5188	5285	-	97
NW_015922627.1	12902	13088	+	186
NW_015922631.1	6280	6306	+	26
NW_015922631.1	6259	6337	+	78
NW_015922638.1	6887	7061	-	174
NW_015922665.1	47573	47712	+	139
NW_015922775.1	103896	103990	+	94
NW_015922800.1	12744	13036	-	292
NW_015922835.1	74738	74908	-	170

NW_015922866.1	30825	30922	+	97
NW_015922866.1	30766	30927	+	161
NW_015922992.1	14004	14132	-	128
NW_015922992.1	13968	14128	-	160
NW_015923181.1	13744	13894	-	150
NW_015923197.1	48417	48513	-	96
NW_015923273.1	4907	4996	-	89
NW_015923293.1	28983	29183	-	200
NW_015923297.1	8503	8657	-	154
NW_015923358.1	39628	39716	+	88
NW_015923456.1	24305	24368	+	63
NW_015923456.1	7464	7595	+	131
NW_015923483.1	12639	12789	-	150
NW_015923496.1	1612	1730	-	118
NW_015923562.1	27263	27332	-	69
NW_015923598.1	17258	17387	-	129
NW_015923717.1	45465	45667	-	202
NW_015923842.1	48010	48181	+	125
NW_015923849.1	22791	22865	-	74
NW_015923882.1	22344	22440	-	96
NW_015923906.1	9362	9403	-	41
NW_015923920.1	4336	4539	+	203
NW_015923998.1	5548	5721	+	173
NW_015924019.1	9451	9558	-	71
NW_015924026.1	5479	5602	-	123

NW_015924067.1	90134	90338	-	204
NW_015924091.1	16781	16873	+	92
NW_015924246.1	19937	20058	+	121
NW_015924266.1	32975	33163	+	188
NW_015924283.1	136792	136906	+	114
NW_015924319.1	18027	18059	-	32
NW_015924383.1	97323	97388	+	65
NW_015924442.1	123302	123517	-	215
NW_015924521.1	8038	8159	+	121
NW_015924557.1	7025	7215	-	99
NW_015924609.1	279	400	+	121
NW_015924647.1	70398	70612	+	214
NW_015924692.1	4449	4532	+	83
NW_015924703.1	53303	53353	-	50
NW_015924749.1	62561	62736	-	175
NW_015924749.1	41430	41500	+	70
NW_015924776.1	15767	15896	+	129
NW_015924776.1	19748	19839	+	91
NW_015924789.1	60043	60202	-	159
NW_015924798.1	29826	29901	-	75
NW_015924819.1	30895	31086	+	191
NW_015924824.1	62821	62913	+	92
NW_015924910.1	36207	36396	+	189
NW_015924957.1	35090	35150	+	60
NW_015924967.1	219718	219840	-	122

NW_015924977.1	16233	16397	+	164
NW_015924983.1	14842	15027	+	185
NW_015925131.1	22579	22692	-	113
NW_015925145.1	73710	73884	-	174
NW_015925192.1	6241	6624	+	383
NW_015925224.1	21581	21639	+	58
NW_015925272.1	9509	9607	-	98
NW_015925353.1	4673	4747	+	74
NW_015925463.1	217135	217258	-	92
NW_015925547.1	42215	42305	-	90
NW_015925547.1	190992	191120	-	128
NW_015925614.1	119326	119493	+	167
NW_015925690.1	10642	10718	+	76
NW_015925709.1	9488	9537	+	49
NW_015925709.1	9449	9518	+	69
NW_015925709.1	9374	9551	+	177
NW_015925760.1	18569	18909	+	340
NW_015925891.1	46718	46779	-	61
NW_015925982.1	20610	20783	-	173
NW_015926060.1	12388	12516	-	110
NW_015926076.1	39836	39891	+	55
NW_015926091.1	22542	22748	+	206
NW_015926121.1	2664	2731	-	67
NW_015926153.1	53096	53245	-	149
NW_015926199.1	86337	86396	-	59

NW_015926258.1	10845	11007	+	162
NW_015926277.1	20074	20243	-	169
NW_015926317.1	11770	11878	-	108
NW_015926589.1	12651	12802	-	151
NW_015926596.1	66403	66506	-	103
NW_015926689.1	2374	2445	+	71
NW_015926798.1	71778	71979	+	201
NW_015926845.1	6031	6234	-	203
NW_015926850.1	58605	58764	+	113
NW_015926850.1	58611	58759	+	107
NW_015926911.1	26231	26415	-	184
NW_015926944.1	64684	64951	+	267
NW_015926962.1	46697	46722	+	25
NW_015927091.1	34225	34323	+	98
NW_015927121.1	42174	42309	-	135
NW_015927176.1	2989	3177	+	188
NW_015927253.1	23133	23273	+	140
NW_015927326.1	16977	17069	-	92
NW_015927376.1	42934	43052	+	118
NW_015927404.1	91016	91103	+	87
NW_015927459.1	50993	51085	-	92
NW_015927568.1	4613	4683	+	70
NW_015927569.1	15189	15539	-	350
NW_015927625.1	28018	28192	-	174
NW_015927711.1	24581	24884	+	303

NW_015927837.1	63363	63549	+	186
NW_015927838.1	3838	3889	+	51
NW_015927838.1	1470	1545	+	65
NW_015927960.1	38880	39095	-	215
NW_015927982.1	14676	15092	-	416
NW_015928055.1	36402	36540	-	138
NW_015928080.1	40388	40572	-	184
NW_015928124.1	10955	11376	+	371
NW_015928128.1	56403	56502	+	99
NW_015928145.1	16735	16802	+	67
NW_015928153.1	33107	33127	-	20
NW_015928153.1	33080	33138	-	58
NW_015928200.1	5244	5379	-	135
NW_015928282.1	41525	41660	+	94
NW_015928433.1	11641	11798	+	157
NW_015928548.1	44205	44319	-	114
NW_015928560.1	103853	103961	-	108
NW_015928592.1	50731	50873	+	97
NW_015928592.1	50819	50907	+	88
NW_015928713.1	4020	4052	-	32
NW_015928739.1	26586	26750	-	164
NW_015928803.1	123000	123100	+	100
NW_015928907.1	4715	4801	-	76
NW_015928918.1	6416	6474	-	58
NW_015928936.1	66832	66974	+	142

NW_015929049.1	2712	2788	+	76
NW_015929051.1	120482	120564	-	82
NW_015929212.1	61990	62153	+	163
NW_015929228.1	81168	81319	+	151
NW_015929283.1	8115	8243	+	86
NW_015929377.1	246870	246937	+	67
NW_015929419.1	13036	13252	-	216
NW_015929427.1	27646	27778	-	132
NW_015929432.1	5876	5918	+	42
NW_015929489.1	22604	22683	-	79
NW_015929530.1	11029	11145	-	76
NW_015929586.1	589	644	+	55
NW_015929622.1	106029	106078	-	49
NW_015929751.1	14792	14904	-	112
NW_015929848.1	65595	65698	-	103
NW_015929871.1	23750	23817	+	67
NW_015929922.1	29396	29598	-	202
NW_015929922.1	29393	29605	-	212
NW_015929922.1	29386	29608	-	222
NW_015929960.1	15451	15503	-	52
NW_015929960.1	15437	15521	-	84
NW_015929967.1	12814	12969	+	155
NW_015930042.1	32614	32768	+	154
NW_015930045.1	69434	69585	+	151
NW_015930170.1	52524	52904	+	380

NW_015930190.1	4806	4876	-	70
NW_015930330.1	2812	2889	-	77
NW_015930395.1	6347	6454	+	107
NW_015930447.1	14225	14480	+	255
NW_015930455.1	2545	2606	+	61
NW_015930485.1	204	269	+	65
NW_015930491.1	40438	40566	-	128
NW_015930588.1	62907	62949	+	42
NW_015930828.1	7642	7710	-	68
NW_015930843.1	21169	21260	+	91
NW_015930843.1	55935	56083	+	148
NW_015930886.1	57721	57853	+	132
NW_015930915.1	25911	26001	+	90
NW_015930965.1	21789	22004	+	215
NW_015931076.1	13190	13250	-	60
NW_015931221.1	36002	36219	+	217
NW_015931294.1	22516	22972	-	456
NW_015931325.1	6057	6157	-	100
NW_015931336.1	114910	115302	+	392
NW_015931389.1	86166	86371	-	205
NW_015931526.1	36671	36763	+	92
NW_015931547.1	32728	32836	-	55
NW_015931583.1	49233	49362	+	79
NW_015931631.1	32532	32838	+	306
NW_015931690.1	7793	7818	+	25

NW_015931859.1	42484	42589	+	105
NW_015931892.1	26337	26474	-	137
NW_015931929.1	2636	2843	+	207
NW_015932029.1	18431	18576	-	145
NW_015932046.1	8861	8985	+	124
NW_015932120.1	72377	72453	-	76
NW_015932131.1	45801	45905	-	104
NW_015932174.1	14833	14928	+	95
NW_015932185.1	24954	25207	+	63
NW_015932187.1	28828	28880	+	52
NW_015932264.1	11272	11423	+	151
NW_015932287.1	48192	48330	+	138
NW_015932287.1	48128	48352	+	224
NW_015932296.1	116069	116255	+	186
NW_015932399.1	5232	5446	-	214
NW_015932410.1	168372	168481	+	109
NW_015932412.1	33628	33728	+	100
NW_015932470.1	30629	30759	-	130
NW_015932557.1	24640	24745	+	105
NW_015932609.1	26678	26824	-	146
NW_015932618.1	20340	20429	-	89
NW_015932637.1	19625	19955	+	330
NW_015932637.1	21566	21598	-	32
NW_015932673.1	133891	134069	-	178
NW_015932691.1	46581	46728	+	147

NW_015932735.1	26252	26305	-	53
NW_015932816.1	64559	64614	+	55
NW_015932864.1	22930	23105	+	175
NW_015932864.1	39945	40103	-	158
NW_015932885.1	89626	89703	-	77
NW_015933163.1	100546	100586	+	40
NW_015933189.1	18955	19091	+	91
NW_015933232.1	18520	18767	-	247
NW_015933252.1	15608	15809	-	201
NW_015933253.1	2181	2328	+	147
NW_015933253.1	25601	25636	-	35
NW_015933256.1	48905	49056	+	97
NW_015933444.1	8318	8677	+	359
NW_015933631.1	27965	28091	-	126
NW_015933680.1	9236	9265	+	29
NW_015933796.1	8757	8907	+	150
NW_015933796.1	8738	8917	+	179
NW_015933808.1	31121	31187	+	60
NW_015933818.1	13989	14158	+	148
NW_015933840.1	114654	114780	-	126
NW_015933858.1	53648	53772	-	124
NW_015933980.1	116644	116783	-	139
NW_015934010.1	15460	15487	-	27
NW_015934071.1	28134	28326	+	192
NW_015934082.1	76786	76928	+	142

NW_015934119.1	105572	105604	+	32
NW_015934184.1	165787	165957	+	170
NW_015934203.1	8798	8901	-	103
NW_015934220.1	9251	9438	-	187
NW_015934231.1	57524	57635	+	111
NW_015934242.1	38212	38312	+	60
NW_015934301.1	6117	6212	+	95
NW_015934332.1	1798	1955	-	157
NW_015934363.1	70915	70959	+	44
NW_015934384.1	14470	14600	+	130
NW_015934407.1	1117	1179	+	62
NW_015934501.1	45587	45645	+	58
NW_015934544.1	12118	12181	+	63
NW_015934564.1	85424	85529	-	105
NW_015934637.1	15364	15469	-	105
NW_015934651.1	12445	12523	-	78
NW_015934705.1	55004	55119	+	115
NW_015934726.1	126583	126700	+	117
NW_015934728.1	45531	45816	-	285
NW_015934794.1	45444	45554	+	71
NW_015934794.1	44995	45161	+	78
NW_015934924.1	4132	4614	-	482
NW_015934933.1	121485	121568	+	83
NW_015934999.1	1123	1192	-	69
NW_015935048.1	58886	59010	-	124

NW_015935085.1	48408	48491	+	83
NW_015935085.1	54356	54476	+	120
NW_015935209.1	70200	70258	+	58
NW_015935215.1	34081	34263	-	182
NW_015935321.1	17460	17609	+	149
NW_015935321.1	17461	17612	+	151
NW_015935321.1	69706	69867	-	161
NW_015935426.1	3830	3988	-	158
NW_015935452.1	10370	10447	-	77
NW_015935507.1	37802	37838	+	36
NW_015935532.1	166754	166798	+	44
NW_015935532.1	166740	166808	+	68
NW_015935554.1	15329	15468	+	76
NW_015935561.1	136308	136367	+	55
NW_015935578.1	12334	12530	+	196
NW_015935659.1	4681	4937	-	256
NW_015935691.1	80294	80401	+	107
NW_015935698.1	2604	2762	-	158
NW_015935745.1	6604	6659	+	55
NW_015935769.1	36641	36871	-	159
NW_015935900.1	3652	3787	+	135
NW_015935920.1	21690	21801	+	111
NW_015935920.1	21676	21792	+	116
NW_015935958.1	9799	9974	+	175
NW_015936080.1	31781	31869	+	88

NW_015936082.1	136026	136174	-	148
NW_015936136.1	16883	16980	+	97
NW_015936146.1	38110	38164	+	54
NW_015936163.1	88138	88183	-	45
NW_015936275.1	159587	159705	-	118
NW_015936282.1	5153	5212	+	59
NW_015936418.1	40777	40823	+	46
NW_015936527.1	10715	10775	-	60
NW_015936531.1	4261	4358	+	97
NW_015936549.1	37866	37912	-	46
NW_015936593.1	27076	27315	+	239
NW_015936598.1	37918	38096	+	178
NW_015936654.1	11521	11654	-	133
NW_015936654.1	11502	11677	-	175
NW_015936671.1	123233	123382	+	149
NW_015936770.1	62575	62642	+	67
NW_015936776.1	44141	44253	+	112
NW_015936873.1	10013	10113	+	100
NW_015936898.1	78712	78806	+	94
NW_015936970.1	59158	59261	-	103
NW_015936986.1	29432	29589	-	157
NW_015936986.1	29436	29568	-	132
NW_015937016.1	16980	17085	-	105
NW_015937047.1	14122	14254	+	132
NW_015937113.1	61846	61957	+	111

NW_015937130.1	10472	10515	+	43
NW_015937130.1	10475	10516	+	41
NW_015937176.1	95022	95117	+	95
NW_015937234.1	325924	326092	-	168
NW_015937333.1	81357	81397	-	40
NW_015937369.1	8447	8491	+	44
NW_015937440.1	4291	4483	-	192
NW_015937464.1	39789	39885	-	96
NW_015937585.1	13949	14028	-	79
NW_015937677.1	8292	8457	-	74
NW_015937723.1	14291	14472	-	181
NW_015937748.1	22698	22728	+	30
NW_015937776.1	40730	40915	-	185
NW_015937850.1	2616	2904	-	288
NW_015937850.1	20424	20685	-	261
NW_015937880.1	33530	33599	+	69
NW_015937916.1	44045	44181	-	94
NW_015937946.1	11604	11800	+	196
NW_015938041.1	12779	12869	-	90
NW_015938041.1	5441	5476	-	35
NW_015938054.1	42958	43056	-	98
NW_015938063.1	8766	8829	-	63
NW_015938092.1	29889	30247	+	358
NW_015938092.1	29907	30285	+	378
NW_015938092.1	29880	30283	+	403

NW_015938439.1	2710	2835	+	125
NW_015938523.1	61209	61327	+	118
NW_015938557.1	38510	38698	-	188
NW_015938557.1	38501	38733	-	232
NW_015938570.1	177465	177669	-	149
NW_015938719.1	11191	11360	-	147
NW_015938729.1	10738	10883	+	145
NW_015938751.1	62647	62923	-	276
NW_015938751.1	62613	62931	-	318
NW_015938930.1	83080	83183	+	103
NW_015939000.1	7298	7453	+	155
NW_015939057.1	38665	38758	-	93
NW_015939093.1	15755	15821	-	66
NW_015939169.1	17188	17341	-	153
NW_015939409.1	17442	17584	+	142
NW_015939450.1	11823	11890	+	67
NW_015939577.1	19927	19994	+	67
NW_015939676.1	18475	18551	-	41
NW_015939729.1	7468	7812	-	344
NW_015939830.1	4656	4793	-	137
NW_015939894.1	2358	2394	-	36
NW_015939915.1	27932	28043	-	111
NW_015939970.1	92155	92162	+	7
NW_015940113.1	51535	51832	+	297
NW_015940146.1	37347	37531	-	184

NW_015940157.1	19516	19567	+	51
NW_015940333.1	155356	155436	-	80
NW_015940347.1	22373	22511	-	138
NW_015940397.1	10566	10594	-	28
NW_015940444.1	4564	4746	+	120
NW_015940481.1	24965	25241	+	276
NW_015940540.1	13988	14095	+	107
NW_015940548.1	30255	30417	+	162
NW_015940653.1	22344	22487	-	143
NW_015940684.1	13444	13599	-	79
NW_015940712.1	42353	42463	-	110
NW_015940752.1	11754	11898	-	144
NW_015940826.1	32637	32708	-	71
NW_015940858.1	30191	30317	+	126
NW_015940961.1	6679	6817	-	138
NW_015940996.1	38892	39111	+	219
NW_015941027.1	2450	2518	-	68
NW_015941028.1	17029	17144	+	115
NW_015941028.1	17870	18046	+	176
NW_015941077.1	32583	32677	-	94
NW_015941143.1	23016	23123	-	107
NW_015941377.1	5336	5395	+	59
NW_015941377.1	5227	5410	+	183
NW_015941455.1	9537	9554	-	17
NW_015941591.1	5552	5726	+	174

NW_015941628.1	1667	1757	+	90
NW_015941750.1	113144	113254	+	110
NW_015941766.1	16330	16443	+	113
NW_015941832.1	21254	21393	+	78
NW_015941915.1	16649	16834	+	163
NW_015941917.1	78409	78610	-	201
NW_015941949.1	6803	6913	-	110
NW_015942170.1	16214	16278	+	64
NW_015942170.1	73223	73323	-	100
NW_015942212.1	17516	17620	-	104
NW_015942243.1	6180	6300	+	120
NW_015942316.1	181767	181801	+	34
NW_015942395.1	65893	66036	-	143
NW_015942512.1	32572	32643	+	71
NW_015942759.1	22395	22491	+	96
NW_015942786.1	12383	12450	-	67
NW_015942820.1	32228	32326	-	98
NW_015942850.1	6574	6686	+	112
NW_015942850.1	18395	18547	-	101
NW_015942910.1	37541	37616	+	75
NW_015942954.1	42148	42309	-	161
NW_015942979.1	4411	4478	+	59
NW_015943061.1	200333	200430	+	97
NW_015943089.1	25697	25739	+	42
NW_015943089.1	26549	26686	-	137

NW_015943089.1	26523	26667	-	144
NW_015943152.1	57284	57319	-	35
NW_015943161.1	38021	38080	-	59
NW_015943162.1	39419	39485	+	66
NW_015943230.1	72397	72551	-	154
NW_015943319.1	3496	3560	-	64
NW_015943319.1	22612	22855	+	243
NW_015943368.1	36951	37016	-	65
NW_015943370.1	43838	44013	+	175
NW_015943447.1	3550	3669	-	119
NW_015943534.1	48552	48620	-	68
NW_015943573.1	9224	9339	-	115
NW_015943586.1	36458	36615	+	157
NW_015943606.1	13333	13422	-	89
NW_015943790.1	5872	5998	-	126
NW_015943808.1	66423	66537	-	114
NW_015943812.1	2713	3066	+	353
NW_015943877.1	15959	16099	+	140
NW_015943899.1	38774	39019	+	245
NW_015943915.1	88774	88828	+	54
NW_015944054.1	4087	4284	+	197
NW_015944096.1	27982	28273	-	291
NW_015944250.1	29547	29682	+	135
NW_015944264.1	44957	45003	+	46
NW_015944327.1	33868	33963	-	95

NW_015944340.1	5778	5832	-	54
NW_015944425.1	8343	8417	+	74
NW_015944521.1	18018	18134	+	112
NW_015944565.1	59523	59643	-	120
NW_015944632.1	12141	12285	-	109
NW_015944696.1	145794	145938	-	144
NW_015944917.1	56170	56307	+	137
NW_015944917.1	57282	57413	+	131
NW_015945086.1	50556	50626	+	70
NW_015945117.1	20993	21193	+	187
NW_015945158.1	188670	188821	-	151
NW_015945158.1	188652	188837	-	185
NW_015945193.1	6598	6714	+	116
NW_015945193.1	6573	6727	+	154
NW_015945193.1	13156	13358	+	202
NW_015945209.1	29954	30030	-	76
NW_015945235.1	20262	20328	-	66
NW_015945235.1	20244	20313	-	69
NW_015945335.1	2521	2875	+	351
NW_015945414.1	32547	32632	-	85
NW_015945827.1	6925	7034	+	109
NW_015945875.1	36994	37137	-	143
NW_015945941.1	45653	46010	+	289
NW_015945958.1	75518	75626	-	100
NW_015946040.1	75318	75378	-	60

NW_015946040.1	115952	116065	-	113
NW_015946110.1	31519	31739	+	220
NW_015946357.1	2200	2457	-	257
NW_015946496.1	2306	2462	-	156
NW_015946508.1	63988	64210	+	222
NW_015946521.1	6126	6220	+	94
NW_015946604.1	2367	2527	-	110
NW_015946624.1	13909	14077	-	168
NW_015946636.1	53328	53428	-	100
NW_015946706.1	65665	65740	+	75
NW_015946810.1	6397	6540	-	143
NW_015946810.1	6404	6515	-	111
NW_015946810.1	6389	6552	-	163
NW_015946934.1	41151	41175	+	24
NW_015946985.1	28280	28454	+	174
NW_015947005.1	25600	25662	-	62
NW_015947005.1	26128	26394	-	266
NW_015947193.1	78231	78286	+	55
NW_015947391.1	23510	23681	+	171
NW_015947423.1	14249	14333	+	84
NW_015947507.1	5378	5493	+	115
NW_015947637.1	21274	21402	+	128
NW_015947669.1	29586	29690	+	104
NW_015947703.1	83165	83225	+	60
NW_015947703.1	83157	83236	+	79

NW_015947764.1	29995	30124	-	88
NW_015947772.1	38244	38331	+	87
NW_015947801.1	24778	24859	-	81
NW_015947834.1	75513	75616	-	103
NW_015947850.1	7442	7495	-	53
NW_015947872.1	3651	3708	-	57
NW_015948033.1	50429	50469	+	40
NW_015948033.1	50383	50521	+	138
NW_015948079.1	62763	62808	+	45
NW_015948079.1	34308	34464	-	156
NW_015948084.1	14803	14920	-	117
NW_015948152.1	19258	19318	+	60
NW_015948154.1	61094	61192	-	98
NW_015948154.1	85723	85814	+	91
NW_015948182.1	27301	27361	+	60
NW_015948395.1	16839	16886	+	47
NW_015948504.1	7278	7374	+	96
NW_015948504.1	39086	39177	-	87
NW_015948505.1	34719	35006	-	287
NW_015948524.1	13611	13721	-	91
NW_015948576.1	4624	4749	-	125
NW_015948672.1	23860	23994	+	134
NW_015948690.1	3525	3641	-	116
NW_015948700.1	4167	4278	-	111
NW_015948713.1	19411	19513	-	102

NW_015948723.1	15671	15732	+	61
NW_015948828.1	25788	25861	+	73
NW_015948969.1	195361	195449	-	88
NW_015949053.1	8066	8373	+	294
NW_015949055.1	64281	64434	+	22
NW_015949087.1	46369	46464	+	95
NW_015949127.1	20150	20202	-	52
NW_015949412.1	19007	19030	+	23
NW_015949414.1	14712	14835	-	123
NW_015949438.1	141682	141734	-	52
NW_015949578.1	4404	4535	-	131
NW_015949587.1	46681	46881	+	200
NW_015949601.1	14944	14990	+	46
NW_015949603.1	15810	15844	+	34
NW_015949618.1	37935	37974	+	39
NW_015949622.1	137810	137915	-	105
NW_015949622.1	45022	45188	+	143
NW_015949634.1	65597	65765	+	168
NW_015949666.1	16041	16129	+	88
NW_015949695.1	5862	5941	-	79
NW_015949923.1	9159	9298	+	139
NW_015949979.1	15274	15402	+	128
NW_015950202.1	68493	68614	-	81
NW_015950220.1	12880	13016	+	136
NW_015950368.1	54535	54596	-	61

NW_015950466.1	36597	36771	-	174
NW_015950480.1	31283	31366	-	83
NW_015950514.1	27287	27380	-	93
NW_015950627.1	53069	53217	-	148
NW_015950798.1	25394	25486	+	92
NW_015951003.1	33978	34068	+	90
NW_015951006.1	45229	45407	+	178
NW_015951006.1	5391	5623	+	232
NW_015951026.1	51729	51987	-	258
NW_015951046.1	25690	25790	+	62
NW_015951085.1	16582	16678	-	80
NW_015951185.1	5936	6074	+	138
NW_015951196.1	154964	155062	+	98
NW_015951222.1	170332	170373	-	41
NW_015951222.1	170228	170361	-	133
NW_015951222.1	174149	174197	-	48
NW_015951294.1	37597	37770	+	136
NW_015951358.1	6258	6437	-	97
NW_015951514.1	15662	15738	+	76
NW_015951735.1	40750	40892	-	142
NW_015951933.1	69324	69384	-	60
NW_015951974.1	8691	8894	+	203
NW_015952228.1	7768	7880	+	112
NW_015952433.1	35826	35948	+	122
NW_015952523.1	14773	14844	-	71

NW_015952580.1	42796	42977	+	181
NW_015952592.1	4360	4498	-	138
NW_015952863.1	19145	19483	+	250
NW_015952863.1	19156	19492	+	259
NW_015952940.1	1933	2004	-	71
NW_015953096.1	7554	7783	+	229
NW_015953504.1	89187	89344	-	157
NW_015953554.1	56102	56256	+	154
NW_015953728.1	13093	13575	-	482
NW_015953731.1	14738	14848	-	110
NW_015953746.1	19706	20162	-	456
NW_015953746.1	19698	20062	-	364
NW_015953755.1	7914	8080	-	166
NW_015953774.1	21674	21751	-	77
NW_015953833.1	30786	30980	+	98
NW_015953888.1	51673	51871	-	198
NW_015953989.1	17683	17715	+	32
NW_015954036.1	23760	23939	-	179
NW_015954166.1	74189	74260	+	71
NW_015954188.1	7277	7309	-	32
NW_015954189.1	88790	88832	-	42
NW_015954264.1	60716	60906	+	190
NW_015954267.1	41314	41401	-	87
NW_015954359.1	327058	327164	+	55
NW_015954445.1	16449	16531	-	82

NW_015954473.1	42900	42928	+	28
NW_015954522.1	18013	18174	-	161
NW_015954584.1	13352	13537	+	185
NW_015954637.1	52866	52979	-	113
NW_015954642.1	11813	12013	+	200
NW_015954653.1	36441	36521	-	80
NW_015954668.1	14249	14305	-	56
NW_015954695.1	63899	64025	-	126
NW_015954748.1	4679	4753	+	74
NW_015954776.1	9666	9764	+	98
NW_015954809.1	48150	48210	-	60
NW_015954809.1	48145	48207	-	62
NW_015954833.1	8774	8858	-	84
NW_015954893.1	42364	42548	-	184
NW_015954893.1	35505	35608	-	64
NW_015954901.1	21786	21978	-	71
NW_015954940.1	16100	16242	-	142
NW_015955055.1	38122	38258	+	136
NW_015955156.1	145299	145437	-	138
NW_015955219.1	68232	68383	-	151
NW_015955427.1	12563	12676	-	113
